# Improved Skill of Rotaxanes to Recognize Cations: A Theoretical Perspective

**DOI:** 10.1021/acsphyschemau.4c00090

**Published:** 2025-01-06

**Authors:** Renato Pereira Orenha, Alvaro Muñoz-Castro, Maurício Jeomar Piotrowski, Giovanni F. Caramori, Renato Gonçalves Rocha, Renato Luis Tame Parreira

**Affiliations:** † Núcleo de Pesquisas em Ciências Exatas e Tecnológicas, Universidade de Franca, Av. Dr. Armando de Sáles Oliveira 201, Franca, São Paulo 14404-600, Brazil; ‡ Facultad de Ingeniería, Arquitectura y Diseño, Universidad San Sebastián, Bellavista 7, Santiago 8420524, Chile; § Department of Physics, Federal University of Pelotas, Pelotas, Rio Grande do Sul 96010-900, Brazil; ∥ Departamento de Química, Universidade Federal de Santa Catarina, Campus Universitário Trindade, CP 476, Florianópolis, Santa Catarina 88040-900, Brazil

**Keywords:** MIMs, transition metal, alkali metal, cation, EDA, NOCV, VDD

## Abstract

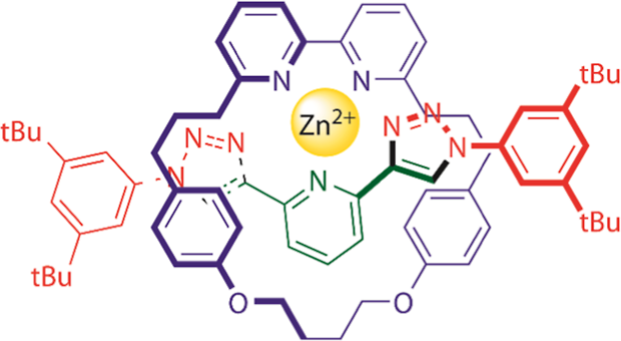

Cations have significant applications in fields such as medicinal inorganic chemistry and catalysis. Rotaxanes are composed of a macrocyclic structure that is mechanically interlocked with a linear molecule. These mechanically interlocked molecules (MIMs) provide a potential chemical environment that allows for the interaction with cations. In this study, the bonding situations between rotaxanes or their acyclic/cyclic molecular derivatives and: (i) transition metal (Zn^2+^ and Cd^2+^); or (ii) alkali metal (Li^+^, Na^+^, and K^+^), cations have been studied. It is notable that among the MIMs structures, the rotaxanes demonstrate enhanced interactions with cations in comparison to the cyclic and, notably, the acyclic derivative molecules. The modification of rotaxane structures through structural changes and chemical reduction represents an intriguing approach to enhance cationic recognition, which is supported by the formation of more favorable electrostatic and/or orbital interaction energies in comparison with Pauli repulsive energies. The findings of this investigation can be employed in the synthesis of compounds with enhanced cation recognition capabilities.

## Introduction

A significant challenge in chemical sciences is the regulation of the coordination number and geometry of transition metal ions. It has a number of applications, most notably in medicinal inorganic chemistry,^1^ photochemistry,^2^ and catalysis.^3,4^ Furthermore, alkali metal ions are involved in several enzymatic processes.^5,6^ The transport of cations, such as Na^+^ and K^+^, across phospholipid bilayers appears as a critical process to life.^7^ The potassium ion is highly accumulated in diverse types of living cells and shows to be indispensable for numerous physiological purposes.^8^ Conversely, the sodium ion is toxic at higher concentrations and can be lethal.^8^ Over the years, lithium has been shown to be the most used alkali metal in organometallic chemistry.^9^ More recently, sodium and potassium have also been demonstrated to be capable of mediating organic reactions, such as polymerization^10^ main-group homogeneous catalysis,^11^ and stoichiometric organic synthesis.^12^

Mechanically interlocked molecules (MIMs) are composed of entangled molecular units that are constrained in space. These units cannot be separated without causing breaks or distortions in the chemical bonds that connect them.^13^ In this system, the molecular units are joined by mechanical bonds.^13^ The mechanical bonds show vital functions in proteins and nucleic acids, promoting a precise spatial organization and providing additional stability to molecular components.^14−16^ Fascinatingly, artificial molecular machines, which can realize complex tasks, as so as biological molecular machines, have been experimentally synthesized.^17−19^

A rotaxane can be defined as a MIM comprising (i) a dumbbell-shaped molecular entity threaded through a (ii) macrocyclic ring. It is characterized by a kinetic trapping of the two components, due to the larger diameter of the dumbbell stoppers relative to the internal diameter of the ring, which inhibits dissociation.^20^ The delimited environment proportioned by mechanical bonds allows the stabilization of bound metal ions.^21,22^ An interesting strategy applied to recognize ions involves the use of isolated or a combination of diverse noncovalent interactions, as, for example, anion–π interactions,^23^ coordination to metal ions,^24^ hydrophobicity,^25^ electrostatic interactions,^26^ and halogen and hydrogen bonds.^27^ Other structures appear as potential candidates for recognizing cations mainly via cation–π interaction.^28^ Further, theoretical investigation systematically explores cation affinities across the periodic table, providing critical insights into the fundamental principles that govern cation–ligand interactions, such as trends in binding strength and selectivity.^29^ The ability of MIMs to recognize ions is typically evaluated in relation to the non-interlocked derivative molecules.^30,31^ A recent investigation has compared the ability of catenanes (a distinct class of MIMs) to recognize ions. This comparison has been conducted on acyclic and cyclic molecular derivatives that contain an identical number of chemical groups available to interact with cations and anions.^32^ It is of particular significance that this study has demonstrated that acyclic derivative compounds are more likely to interact with ions in the context of catenanes. However, the degree of preference for interaction with cations or anions is contingent upon the chemical environment that is constituted by the catenanes or cyclic derivative molecules. Accordingly, further research is required to assess the ability of MIMs to recognize ions.

**Figure 1:**
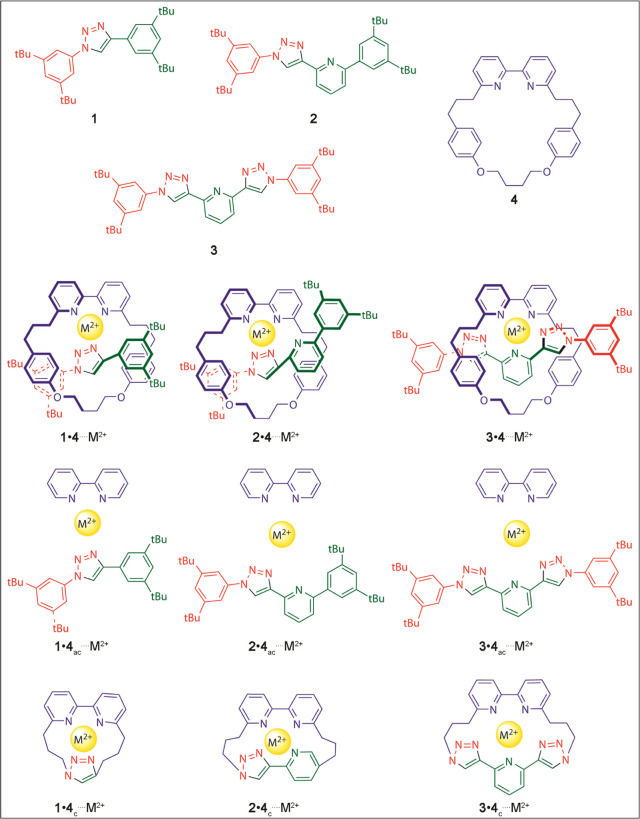
Rotaxanes (**1**•**4**, **2**•**4,** and **3**•**4**, composed of non-interlocked molecular structures **1**–**4**) and acyclic (**1**•**4**_ac_, **2**•**4**_ac_, and **3**•**4**_ac_) or cyclic (**1**•**4**_c_, **2**•**4**_c_ and **3**•**4**_c_) molecular derivatives for the recognition of cations (M^2+^ = Zn^2+^ or Cd^2+^).

**Figure 2:**
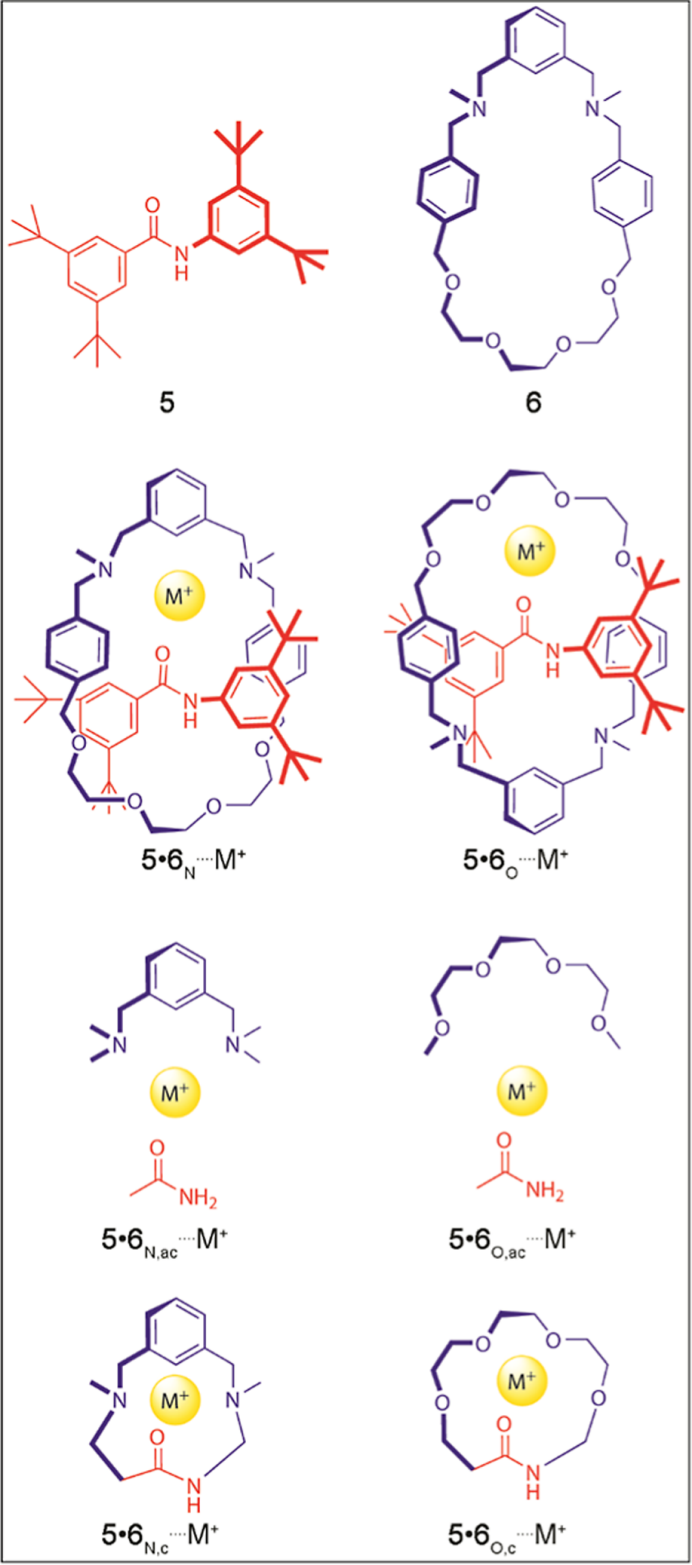
Rotaxanes (**5**•**6**_N_ and **5**•**6**_O_, composed of non-interlocked molecular structures **5** and **6**) and acyclic (**5**•**6**_N,ac_ and **5**•**6**_O,ac_) or cyclic (**5**•**6**_N,c_ and **5**•**6**_O,c_) molecular derivatives for the recognition of cations (M^+^ = Li^+^, Na^+^ or K^+^).

**Figure 3:**
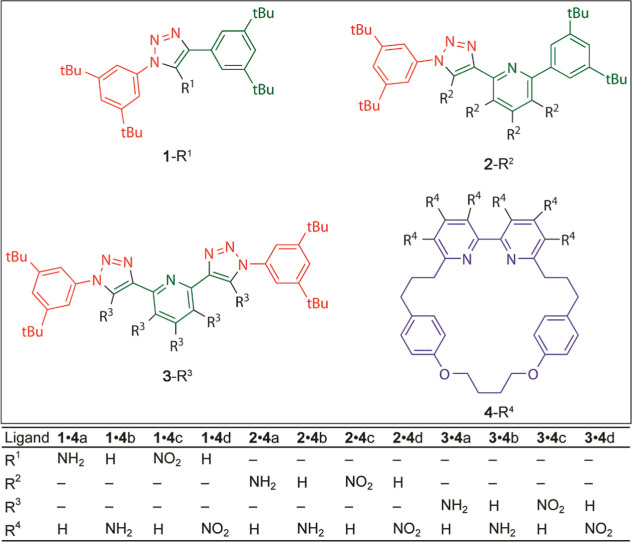
Proposed structural modifications for the molecular components of the **1**•**4**, **2**•**4**, and **3**•**4** rotaxanes.

**Figure 4:**
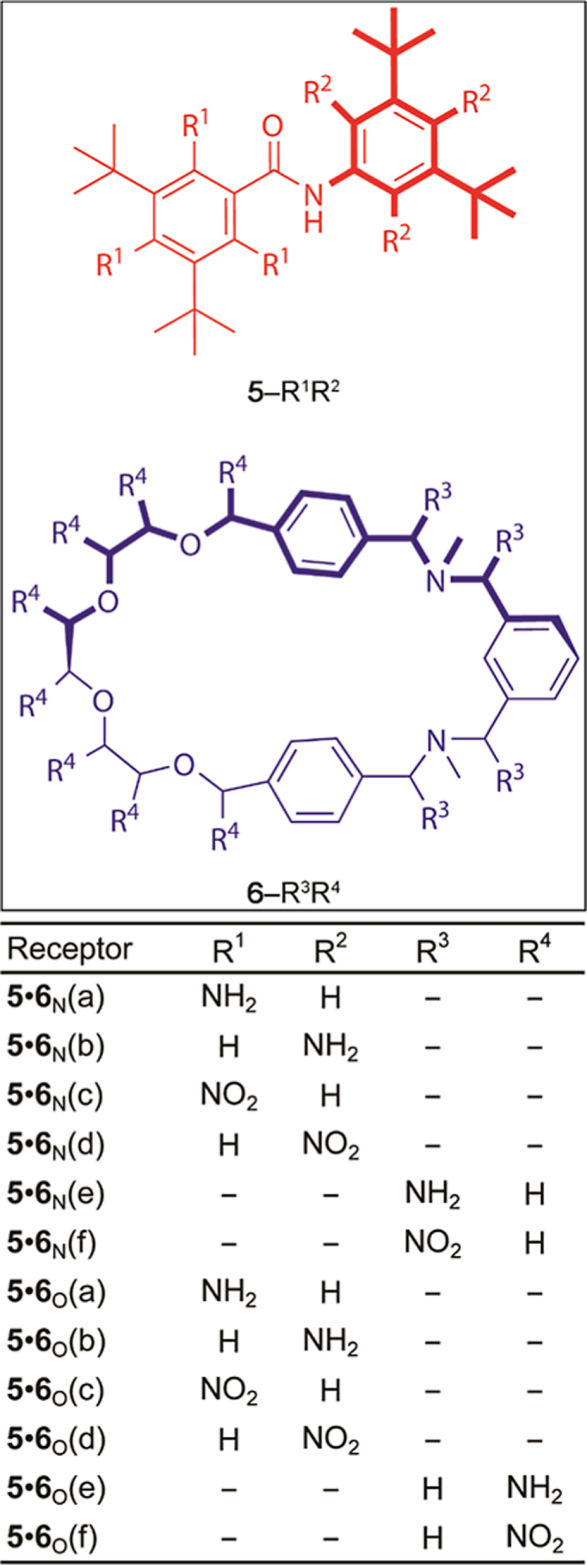
Proposed structural modifications for the molecular components of the **5**•**6**_N_ and **5**•**6**_O_ rotaxanes.

In this context, the nature of the chemical bonds present between the Zn^2+^ or Cd^2+^ transition metal cation and (i) rotaxanes (experimentally investigated by Cirulli et al.)^30^ and (ii) acyclic/cyclic molecular derivatives will be initially elucidated (Figure 1). Subsequently, the bonding situation between the Li^+^, Na^+^, or K^+^ alkali metal cation and (i) rotaxanes (experimentally investigated by Lee et al.)^33^ and (ii) acyclic/cyclic molecular derivatives also will be investigated (Figure 2). It is of particular importance to evaluate the influence of the mechanical bonds concerning ionic recognition by means of a comparison of the interactions, namely: (i) rotaxane····ion and (ii) acyclic/cyclic molecular derivative····ion. The influence of the cation nature will be studied through the analysis of the receptor····(Zn^2+^ or Cd^2+^) and receptor····(Li^+^, Na^+^, or K^+^) interactions. Furthermore, the substitution of hydrogen atoms with electron donor (−NH_2_) or acceptor (−NO_2_) groups will be employed to modify the rotaxanes, with the aim of regulating the strength of the rotaxane····cation bond (Figures 3 and 4). Finally, the ability of the reduced form of the rotaxanes to interact with cations (Zn^2+^ or Na^+^) will be evaluated. The bonding situations will be investigated through the use of energy decomposition analysis (EDA) in conjunction with the natural orbitals for chemical valence (NOCV) methodology. The charge distribution was calculated using the Voronoi deformation density (VDD) method. The topological analysis of the electron density will be performed using the quantum theory of atoms in the molecules (QTAIM) method. The QTAIM results are discussed in the Supporting Information and, in general, are in agreement with the NOCV and VDD data.

## Results and Discussion

### Relevance of Mechanical Bonds in Rotaxanes for the Recognition of Transition Metal Cations

In order to gain insight into the significance of mechanical bonds in rotaxanes with regard to the recognition of transition metal cations, the rotaxanes····Zn^2+^ chemical bonds will be compared with the acyclic or cyclic molecular derivatives····Zn^2+^ interactions. The initial step will be the application of EDA, in which the interaction energy, Δ*E*_int_, between, for example, **1**•**4** and Zn^2+^, is comprised of four main terms^34^
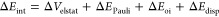
1

The electrostatic term Δ*V*_elstat_ represents the classic electrostatic interaction between the charge distributions of the unperturbed and deformed fragments. The Pauli repulsion, Δ*E*_Pauli_, contains destabilizing interactions between the occupied orbitals. It is related to steric effects. The orbital interaction component, Δ*E*_oi_, means the charge transfer (interaction between the occupied orbitals in a fragment with the nonoccupied orbitals of another moiety) and polarization (mixture of the occupied orbitals with the nonoccupied orbitals in a fragment due the presence of another moiety). The Δ*E*_disp_ energy considers the dispersion corrections, as suggested by Grimme et al.^35,36^

The EDA results related to the bonding situation between the receptors (**1**•**4**_ac_, **2**•**4**_ac_, **3**•**4**_ac_, **1**•**4**_c_, **2**•**4**_c_, **3**•**4**_c_, **1**•**4**, **2**•**4**, or **3**•**4**) and cation (Zn^2+^) have been organized in Table 1. All interactions are found to be predominantly of a polar covalent nature. The interactions between the MIMs (**1**•**4**, **2**•**4**, or **3**•**4**) and cation (Zn^2+^) appear more attractive compared to bonds between the acyclic (**1**•**4**_ac_, **2**•**4**_ac_, or **3**•**4**_ac_) or cyclic (**1**•**4**_c_, **2**•**4**_c_, or **3**•**4**_c_) molecules and cation (Zn^2+^) due to (i) more favorable electrostatic, orbital, and dispersion interactions and/or (ii) less repulsive chemical environment (lower Pauli repulsion). The main orbital interactions will be rationalized through the NOCV methodology, while the charge distribution involved in the chemical bond formation: receptor····cation, will be evaluated via the VDD method.

**Table 1:** Analysis of the Bonding Situation Between the Receptors (**1**•**4**_ac_, **2**•**4**_ac_, **3**•**4**_ac_, **1**•**4**_c_, **2**•**4**_c_, **3**•**4**_c_, **1**•**4**, **2**•**4, 3**•**4**, **1**•**4**^–^, **2**•**4**^–^, or **3**•**4**^–^) and Transition Metal Cations (Zn^2+^ or Cd^2+^) Through the EDA–NOCV Methodology and the Study of Charge Distribution Proportioned from the VDD Method^a^

complex	Δ*E*_int_	Δ*V*_elstat_	Δ*E*_Pauli_	Δ*E*_oi_	Δ*E*_disp_	Δ*E*_oi,1_	Δ*E*_oi,2_	Δ*E*_oi,3_	Δ*E*_oi,4_	Δ*E*_oi,5_	Δ*E*_oi,6_	*q*_Cation_^VDD^
**1**•**4**_ac_····Zn^2+^	–424.1	–264.5 (47)	137.5	–284.0 (51)	–13.1 (2)	–70.9	–30.5	–27.8	–30.8	–19.0	–14.7	–0.643
**1**•**4**_c_····Zn^2+^	–380.1	–244.4 (46)	148.5	–271.4 (51)	–12.8 (2)	–73.4	–36.0	–24.2	–31.2	–15.5	–13.5	–0.623
**1**•**4**····Zn^2+^	–433.8	–250.4 (44)	133.4	–299.9 (53)	–16.9 (3)	–70.9	–30.3	–29.2	–28.9	–19.0	–16.1	–0.590
**2**•**4**_ac_····Zn^2+^	–457.8	–293.3 (49)	139.7	–290.3 (49)	–13.9 (2)	–65.9	–25.4	–28.3	–26.8	–19.2	–15.7	–0.626
**2**•**4**_c_····Zn^2+^	–364.0	–180.6 (39)	101.7	–271.5 (58)	–13.6 (3)	–93.8	–37.6	–25.9	–24.6	–14.3	–8.5	–0.733
**2**•**4**····Zn^2+^	–474.2	–299.4 (48)	144.1	–302.1 (49)	–16.8 (3)	–65.8	–27.7	–26.3	–28.9	–16.7	–17.2	–0.610
**3**•**4**_ac_····Zn^2+^	–482.5	–304.6 (50)	123.0	–288.2 (48)	–12.8 (2)	–64.4	–23.9	–25.4	–27.7	–13.6	–13.4	–0.612
**3**•**4**_c_····Zn^2+^	–402.9	–237.4 (45)	125.5	–275.9 (52)	–15.1 (3)	–73.6	–25.9	–27.9	–29.7	–16.2	–13.5	–0.652
**3**•**4**····Zn^2+^	–497.0	–307.4 (49)	125.2	–299.2 (48)	–15.6 (3)	–64.9	–25.1	–25.5	–27.6	–13.6	–12.9	–0.594
**1**•**4**····Cd^2+^	–365.5	–217.9 (44)	125.8	–252.7 (51)	–20.7 (4)	–69.6	–27.2	–22.4	–19.1	–12.9	–10.9	–0.500
**2**•**4**····Cd^2+^	–399.8	–278.0 (50)	154.0	–253.8 (46)	–22.0 (4)	–60.3	–20.0	–21.9	–22.2	–12.9	–11.0	–0.480
**3**•**4**····Cd^2+^	–421.6	–290.1 (52)	138.7	–250.7 (45)	–19.4 (3)	–58.1	–20.6	–19.1	–21.2	–11.3	–10.6	–0.455
**1**•**4**^–^····Zn^2+^	–574.3	–346.4 (52)	93.3	–307.3 (46)	–13.9 (2)	–158.1	–17.7	–24.0	–22.1	–10.1	–10.9	–0.957
**2**•**4**^–^····Zn^2+^	–612.7	–429.3 (56)	149.0	–315.5 (41)	–16.8 (2)	–53.1	–65.1	–28.6	–24.0	–22.4	–14.4	–0.617
**3**•**4**^–^····Zn^2+^	–626.5	–423.6 (56)	130.9	–318.2 (42)	–15.6 (2)	–60.5	–63.5	–24.0	–28.2	–20.7	–13.5	–0.598

a Δ*E*_int_ = Δ*V*_elstat_ + Δ*E*_Pauli_ + Δ*E*_oi_ + Δ*E*_disp_. Values in parentheses correspond to the percentage of each stabilizing contribution (Δ*V*_elstat_ + Δ*E*_oi_ + Δ*E*_disp_ = 100%). The Δ*E*_oi,1–6_ energies are associated with Δρ_1–6_ density deformation channels. The units of energy and charge are kcal mol^–1^ and a.u., respectively.

The NOCV methodology allows the orbital interactions between, for example, **1**•**4** and Zn^2+^ to be decomposed into pairwise contributions of the most relevant molecular orbitals. The pairwise orbital interaction of a specific bond can be visualized by examining the shape of the deformation density channels, Δρ_k_(r), where the red and blue regions indicate electron density outflow and inflow, respectively. The NOCV method also enables the measurement of the energy (Δ*E*_oi,k_) associated with each density deformation channel (Δρ_k_) to Δ*E*_oi_.^37,38^

**Figure 5:**
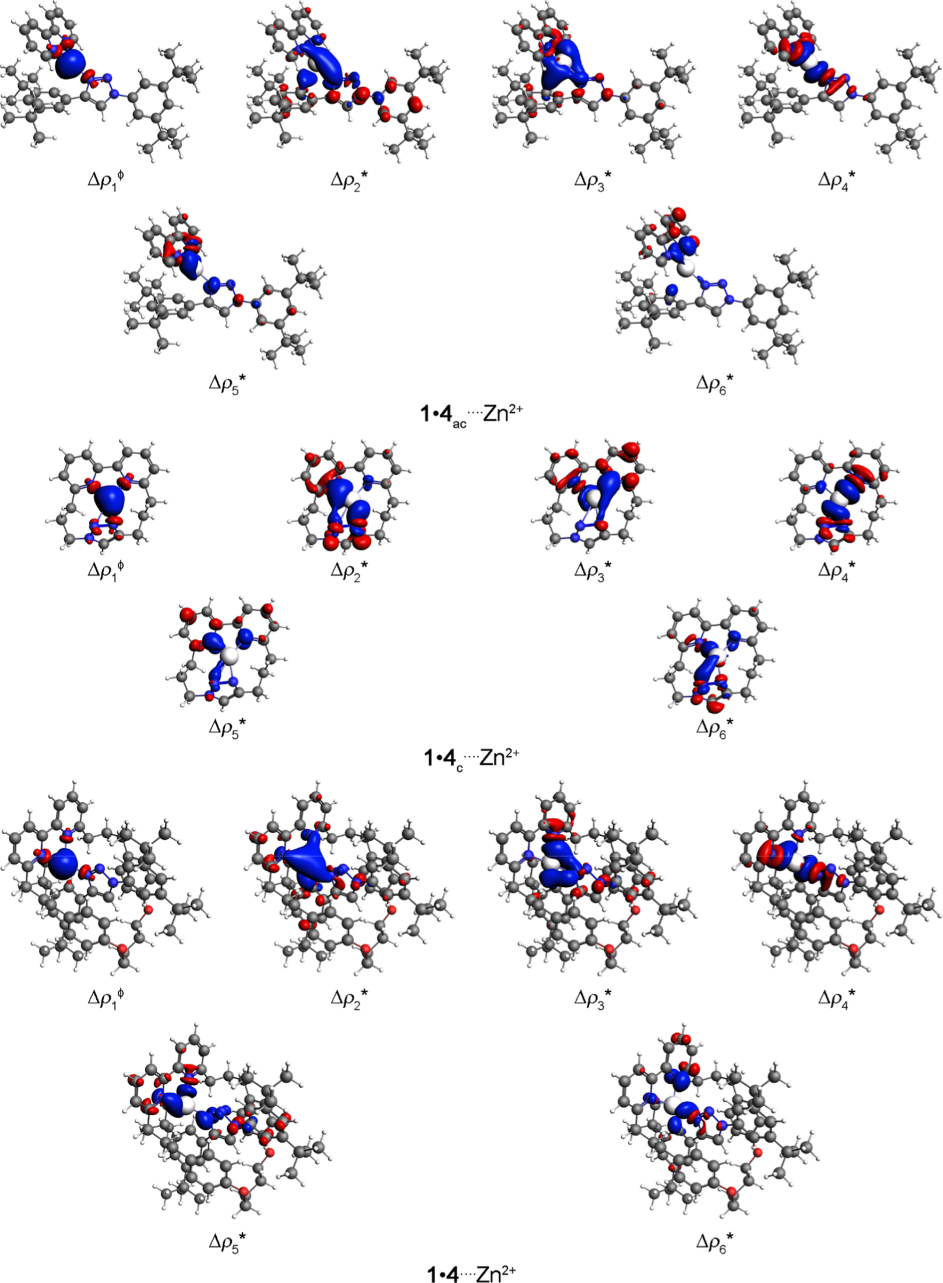
Main density deformation channel surface plots with isovalues = ϕ 0.005 and * 0.001 au, where the red and blue regions indicate the electron density outflow and inflow, respectively, for (**1**•**4**_ac_, **1**•**4**_c_, or **1**•**4**)····Zn^2+^ complexes. Color code for atoms: H = white; C = gray; N = blue; O = red; and Zn = ice.

The main density deformation channel surface plots for the (**1**•**4**_ac_, **2**•**4**_ac_, **3**•**4**_ac_, **1**•**4**_c_, **2**•**4**_c_, **3**•**4**_c_, **1**•**4**, **2**•**4,** or **3**•**4**)····Zn^2+^ complexes are presented in Figures 5, S1 and S2, while the values of each energetic contribution are organized in Table 1. The primary orbital contributions in the (**1**•**4**_ac_, **2**•**4**_ac_, **3**•**4**_ac_, **1**•**4**_c_, **2**•**4**_c_, **3**•**4**_c_, **1**•**4**, **2**•**4,** or **3**•**4**)····Zn^2+^ bonding situations stem from the π and, mainly, from σ (C and, chiefly, N)····Zn^2+^ interactions. Furthermore, the NOCV results demonstrate that, in general, the more favorable MIM····cation interactions are supported by more attractive σ or π (C or N)····Zn^2+^ bonds concerning the acyclic/cyclic molecule····cation interactions. The QTAIM method indicates, through bond critical points (BCPs),^39^ the presence of (C, C–H or, mainly, N)····Zn^2+^ interactions with a partially covalent character^40^ in the (**1**•**4**_ac_, **2**•**4**_ac_, **3**•**4**_ac_, **1**•**4**_c_, **2**•**4**_c_, **3**•**4**_c_, **1**•**4**, **2**•**4,** or **3**•**4**)····Zn^2+^ complexes (Figure S12 and Table S2). In addition, the larger values of the sum of the electron density, ρ_b_, at these BCPs, Δρ_b_, corroborate with the more attractive Δ*E*_oi_ energy in the (**1**•**4**, **2**•**4**, or **3**•**4**)····Zn^2+^ complexes in relation to (**1**•**4**_ac_, **2**•**4**_ac_, or **3**•**4**_ac_)····Zn^2+^ and (**1**•**4**_c_, **2**•**4**_c_, or **3**•**4**_c_)····Zn^2+^ molecules (Tables S2 and 1).

The VDD method indicates that the Zn^2+^ cation is receiving more charge than donating to receptor structures (**1**•**4**_ac_, **2**•**4**_ac_, **3**•**4**_ac_, **1**•**4**_c_, **2**•**4**_c_, **3**•**4**_c_, **1**•**4**, **2**•**4,** or **3**•**4**) (Table 1). In addition, this ion is donating more charge to MIMs (**1**•**4**, **2**•**4,** or **3**•**4**) than to acyclic (**1**•**4**_ac_, **2**•**4**_ac,_ or **3**•**4**_ac_) or cyclic (**1**•**4**_c_, **2**•**4**_c_, or **3**•**4**_c_) molecules in the chemical bond formation process: isolated receptor + isolated cation → receptor····cation complex, justifying the more favorable orbital interactions in the (**1**•**4**, **2**•**4**, or **3**•**4**)····Zn^2+^ complexes in relation to (**1**•**4**_ac_, **2**•**4**_ac_, or **3**•**4**_ac_)····Zn^2+^ and (**1**•**4**_c_, **2**•**4**_c_, or **3**•**4**_c_)····Zn^2+^ compounds. Relevantly, between the atoms present in the **1**•**4**_ac_, **2**•**4**_ac_, **3**•**4**_ac_, **1**•**4**_c_, **2**•**4**_c_, **3**•**4**_c_, **1**•**4**, **2**•**4**, or **3**•**4** compounds, the nitrogen atoms show the most negative VDD charges (Table S11). It indicates that these atoms are the most involved in the charge transfer with the Zn^2+^ cation (showing an average distance N····Zn^2+^ equal to 2.040 Å).

### Influence of the Transition Metal Cation Nature in the Rotaxane····Cation Interaction

In order to evaluate the importance of cation nature in receptor····cation interactions, it is first necessary to examine the EDA results related to the following bonding situations: (i) (**1**•**4**, **2**•**4**, or **3**•**4**)····Zn^2+^ and (ii) (**1**•**4**, **2**•**4**, or **3**•**4**)····Cd^2+^, which are compared (Table 1). So as the (**1**•**4**, **2**•**4**, or **3**•**4**)····Zn^2+^ interactions, the (**1**•**4**, **2**•**4**, or **3**•**4**)····Cd^2+^ bonds also have a chiefly polar covalent character. The (**1**•**4**, **2**•**4**, or **3**•**4**)····Zn^2+^ interactions are more attractive than the (**1**•**4**, **2**•**4**, or **3**•**4**)····Cd^2+^ bonds because of (i) more favorable electrostatic and orbital interactions and/or (ii) a less Pauli repulsive chemical environment. The NOCV analysis indicates that the main orbital interactions present between the receptor structures (**1**•**4**, **2**•**4**, or **3**•**4**) and Cd^2+^ cation involve π and, chiefly, σ (C and, mainly, N)····Cd^2+^ interactions (Figures S3 and S4). Moreover, the NOCV data indicate that the more favorable receptor····Zn^2+^ bonds are maintained by more beneficial σ and π (C or N)····cation interactions in comparison to the receptor····Cd^2+^ bonds. The QTAIM method shows the presence of (i) N····Cd^2+^ interactions with a partially covalent character;^40^ and (ii) (C or C–H)····Cd^2+^ bonds with a predominantly non-covalent nature^40^ (Figure S12 and Table S3). The larger Δρ_b_ values agree with the more favorable Δ*E*_oi_ in the (**1**•**4**, **2**•**4**, or **3**•**4**)····Zn^2+^ compounds in relation to (**1**•**4**, **2**•**4**, or **3**•**4**)····Cd^2+^ molecules (Tables S2, S3, and 1). Overall, the VDD method shows that the receptor compounds (**1**•**4**, **2**•**4**, or **3**•**4**) are donating less charge to Cd^2+^ than to Zn^2+^ in agreement with the less attractive orbital interactions present in the (**1**•**4**, **2**•**4**, or **3**•**4**)····Cd^2+^ structures regarding the (**1**•**4**, **2**•**4**, or **3**•**4**)····Zn^2+^ complexes. In the (**1**•**4**, **2**•**4**, or **3**•**4**)····Cd^2+^ complexes, the nitrogen atoms of the rotaxanes are the most involved in the charge transfer with the Cd^2+^ cation (having an average distance N····Cd^2+^ equal to 2.302 Å).

### Tuning the Rotaxane····Transition Metal Cation Interaction using Structural Changes

The substitution of hydrogen atoms by electron donor (−NH_2_) or acceptor (−NO_2_) groups has been proposed in the receptor structures: (i) **1**•**4**; (ii) **2**•**4**; and (iii) **3**•**4**, aiming to tune the receptor····cation interaction (Figures 1 and 3). As a whole, the EDA results show that the interactions between (i) **1**•**4**a–d, **2**•**4**a–d, or **3**•**4**a–d and (ii) Zn^2+^ show a mainly polar covalent nature (Table S1).

The substitutions that favor the Zn^2+^ recognition are (1) −H → −NH_2_ or −NO_2_ in the −R^1^ position of **1** in **1**•**4** (producing **1**•**4**a and **1**•**4**c, respectively); (2) −H → −NH_2_ in the −R^4^ position of **4** in **1**•**4** (making **1**•**4**b); (3) −H → −NH_2_ in the position: (i) −R^2^ of **2**; (ii) −R^3^ of **3**; and (iii) −R^4^ of **4**, in **2**•**4** or **3**•**4** (creating **2**•**4**a or **3**•**4**a and **2**•**4**b or **3**•**4**b, respectively). It is rationalized from more attractive electrostatic and orbital interactions in the substituted receptor····cation complexes regarding the non-substituted receptor····cation structures.

The NOCV methodology indicates that the primary orbital interactions in the (**1**•**4**, **2**•**4**, or **3**•**4**)(a–d)····Zn^2+^ compounds, similarly to (**1**•**4**, **2**•**4**, or **3**•**4**)····Zn^2+^ molecules, are π and, mainly, σ (C and, chiefly, N)····Zn^2+^ (Figures S5–S10). Besides, all substitutions proposed, on the whole, favor the σ and π (C or N)····Zn^2+^ interactions. The QTAIM method points to the fact that the (**1**•**4**, **2**•**4**, or **3**•**4**)(a–d)····Zn^2+^ complexes are supported by partially covalent (C, C–H, and, mainly, N)····Zn^2+^ interactions (Figure S13 and Table S4). In general, there are larger Δρ_b_ values associated to N····Zn^2+^ BCPs, which are in agreement with the more favorable Δ*E*_oi_ energy in the (**1**•**4**, **2**•**4**, and **3**•**4**)(a–d)····Zn^2+^ interactions compared to (**1**•**4**, **2**•**4**, and **3**•**4**)····Zn^2+^ bonds (Tables S2, S4, 1 and S1).

The VDD method shows, in general, that the substituted compounds: (**1**•**4**, **2**•**4**, or **3**•**4**)(a–d), are donating more charge to Zn^2+^ cation compared to non-substituted molecules: **1**•**4**, **2**•**4**, or **3**•**4**, which agrees with the more favorable orbital interactions present in the (**1**•**4**, **2**•**4**, or **3**•**4**)(a–d)····Zn^2+^ compared to (**1**•**4**, **2**•**4**, or **3**•**4**)····Zn^2+^. Importantly, the nitrogen atoms of the rotaxanes are largely related to charge transfer with the Zn^2+^ cation in the (**1**•**4**, **2**•**4**, or **3**•**4**)(a–d)····Zn^2+^ molecules (appear with an average distance N····Zn^2+^ equal to 2.048 Å).

### Improving the Rotaxane····Transition Metal Cation Interaction via Chemical Reduction

In order to evaluate the influence of the receptor charge on the receptor–transition metal cation interaction, the bond between the chemical reduced receptors (**1**•**4**^–^, **2**•**4**^–^, or **3**•**4**^–^) and cation (Zn^2+^) is compared regarding the interaction between the neutral receptors (**1**•**4**, **2**•**4**, or **3**•**4**) and cation (Zn^2+^). The (**1**•**4**^–^, **2**•**4**^–^, or **3**•**4**^–^)····Zn^2+^ interactions have a predominantly non-covalent character. It is of particular significance that the experimental outcomes demonstrate that the interlocked metal complexes exhibit enhanced robustness with regard to electrochemical manipulation, attributable to the mechanical bond.^30^ The chemical reduction of the receptor (**1**•**4**, **2**•**4**, or **3**•**4** → **1**•**4**^–^, **2**•**4**^–^, or **3**•**4**^–^, respectively) facilitates interaction with the Zn^2+^ cation due to two primary factors: (i) enhanced electrostatic and orbital interactions and/or (ii) a reduced repulsive environment (decreased Pauli repulsion).

The NOCV method shows that the main orbital interactions present in the (**1**•**4**^–^, **2**•**4**^–^, or **3**•**4**^–^)····Zn^2+^ complexes, in the same way as (**1**•**4**, **2**•**4**, or **3**•**4**)····Zn^2+^ compounds are σ and π (C and N)····Zn^2+^ (Figure S11). Additionally, the NOCV results indicate that the chemical reduction of the receptor structures improves the σ and π (C or N)····Zn^2+^ orbital interactions. The QTAIM method shows that the (**1**•**4**^–^, **2**•**4**^–^, or **3**•**4**^–^)····Zn^2+^ compounds are maintained by N····Zn^2+^ interactions with a partially covalent nature (Figure S14 and Table S3). Besides, the similar or lower Δρ_b_ values associated with N····Zn^2+^ BCPs of (**1**•**4**^–^, **2**•**4**^–^, or **3**•**4**^–^)····Zn^2+^ concerning to (**1**•**4**, **2**•**4**, or **3**•**4**)····Zn^2+^ complexes show that the more attractive Δ*E*_oi_ energy in the (**1**•**4**^–^, **2**•**4**^–^, or **3**•**4**^–^)····Zn^2+^ interactions regarding the (**1**•**4**, **2**•**4**, or **3**•**4**)····Zn^2+^ bonds are supported by C····Zn^2+^ orbital interactions (Tables S2, S3, and 1). The VDD method designates that the reduced molecules (**1**•**4**^–^, **2**•**4**^–^, or **3**•**4**^–^) are donating more charge to Zn^2+^ cation compared to neutral compounds **1**•**4**, **2**•**4**, or **3**•**4**, respectively, in agreement with the more favorable orbital interactions in (**1**•**4**^–^, **2**•**4**^–^, or **3**•**4**^–^)····Zn^2+^ regarding (**1**•**4**, **2**•**4**, or **3**•**4**)····Zn^2+^. The VDD data also show that, as in the neutral complexes, in (**1**•**4**^–^, **2**•**4**^–^, or **3**•**4**^–^)····Zn^2+^ structures, the nitrogen atoms of the rotaxanes are the most important atoms associated to charge transfer with the Zn^2+^ ion (in an average distance N····Zn^2+^ equal to 2.085 Å).

### Importance of Mechanical Bonds in Rotaxanes for the Recognition of Alkali Metal Cations

In order to understand the relevance of the mechanical bonds in rotaxanes with respect to the recognition of alkali metal cations, the rotaxanes····Na^+^ interactions were compared to acyclic or cyclic molecular compounds····Na^+^ bonds. The EDA data associated with chemical interactions between the compounds (**5**•**6**_N,ac_, **5**•**6**_N,c_, **5**•**6**_N_, **5**•**6**_O,ac_, **5**•**6**_O,c_, or **5**•**6**_O_) and cation (Na^+^) have been presented in Table 2. All interactions (**5**•**6**_N,ac_, **5**•**6**_N,c_, **5**•**6**_N_, **5**•**6**_O,ac_, **5**•**6**_O,c_, or **5**•**6**_O_)····Na^+^ have a mostly non-covalent character, in which the electrostatic contribution dominates, followed by the orbital contribution and finally the dispersion component. The **5**•**6**_N_ and **5**•**6**_O_ MIMs preferably interact with the Na^+^ cation than its acyclic (**5**•**6**_N,ac_ and **5**•**6**_O,ac_, respectively) or cyclic (**5**•**6**_N,c_ and **5**•**6**_O,c_, respectively) molecular derivatives because of (i) more attractive orbital and dispersion interactions and (ii) less Pauli repulsive chemical environment (only to **5**•**6**_N_····Na^+^ and **5**•**6**_O_····Na^+^ regarding **5**•**6**_N,ac_····Na^+^ and **5**•**6**_O,ac_····Na^+^, respectively) and more favorable electrostatic interactions (only to **5**•**6**_N_····Na^+^ and **5**•**6**_O_····Na^+^ regarding **5**•**6**_N,c_····Na^+^ and **5**•**6**_O,c_····Na^+^, respectively). It is a very interesting result since, in a recent investigation, catenane MIMs demonstrate a lower ability to recognize alkali metal cations (Na^+^) with respect to acyclic or cyclic molecular derivatives.^32^

**Table 2:** Analysis of the Bonding Situation Between the Receptors (**5**•**6**_N,ac_, **5**•**6**_N,c_, **5**•**6**_N_, **5**•**6**_O,ac_, **5**•**6**_O,c_, **5**•**6**_O_, **5**•**6**_N_^–^, or **5**•**6**_O_^–^) and Alkali Metal Cations (Li^+^, Na^+^ or K^+^) Through the EDA–NOCV Methodology and the Study of Charge Distribution Proportioned from the VDD Method^a^

complex	Δ*E*_int_	Δ*V*_elstat_	Δ*E*_Pauli_	Δ*E*_oi_	Δ*E*_disp_	Δ*E*_oi,1_	Δ*E*_oi,2_	Δ*E*_oi,3_	*q*_Cation_^VDD^
**5**•**6**_N,ac_····Na^+^	–87.4	–65.1 (60)	21.6	–33.6 (31)	–10.4 (9)	–5.9	–4.1	–3.5	–0.100
**5**•**6**_N,c_····Na^+^	–60.1	–43.4 (57)	15.4	–25.7 (34)	–6.4 (8)	–5.6	–4.5	–2.7	–0.088
**5**•**6**_N_····Na^+^	–90.8	–51.2 (48)	15.6	–39.5 (37)	–15.8 (15)	–5.3	–4.0	–2.6	–0.109
**5**•**6**_O,ac_····Na^+^	–103.1	–84.9 (66)	25.4	–33.0 (26)	–10.6 (8)	–4.3	–3.2	–2.8	–0.083
**5**•**6**_O,c_····Na^+^	–86.2	–67.0 (62)	22.2	–31.4 (29)	–10.0 (9)	–4.3	–3.1	–2.9	–0.082
**5**•**6**_O_····Na^+^	–110.9	–79.7 (59)	24.7	–40.3 (30)	–15.6 (12)	–4.3	–3.6	–3.1	–0.090
**5**•**6**_N_····K^+^	–74.3	–51.8 (50)	29.4	–35.0 (34)	–16.8 (16)	–4.8	–3.5	–3.4	–0.066
**5**•**6**_N_····Li^+^	–114.9	–72.4 (51)	27.8	–59.4 (42)	–11.0 (8)	–12.2	–10.0	–5.2	–0.174
**5**•**6**_O_····K^+^	–76.0	–56.2 (56)	23.8	–31.8 (32)	–11.9 (12)	–4.1	–3.0	–2.6	–0.073
**5**•**6**_O_····Li^+^	–140.4	–94.2 (57)	24.4	–59.2 (36)	–11.3 (7)	–9.9	–8.8	–5.4	–0.161
**5**•**6**_N_^–^····Na^+^	–155.7	–119.9 (68)	21.5	–42.6 (24)	–14.6 (8)	–8.9	–2.6	–4.7	–0.110
**5**•**6**_O_^–^····Na^+^	–167.2	–134.5 (70)	25.6	–42.8 (22)	–15.4 (8)	–10.9	–4.1		–0.095

a Δ*E*_int_ = Δ*V*_elstat_ + Δ*E*_Pauli_ + Δ*E*_oi_ + Δ*E*_disp_. Values in parentheses correspond to the percentage of each stabilizing contribution (Δ*V*_elstat_ + Δ*E*_oi_ + Δ*E*_disp_ = 100%). The Δ*E*_oi,1–6_ energies are associated with Δρ_1–6_ density deformation channels. The units of energy and charge are kcal mol^–1^ and a.u., respectively.

**Figure 6:**
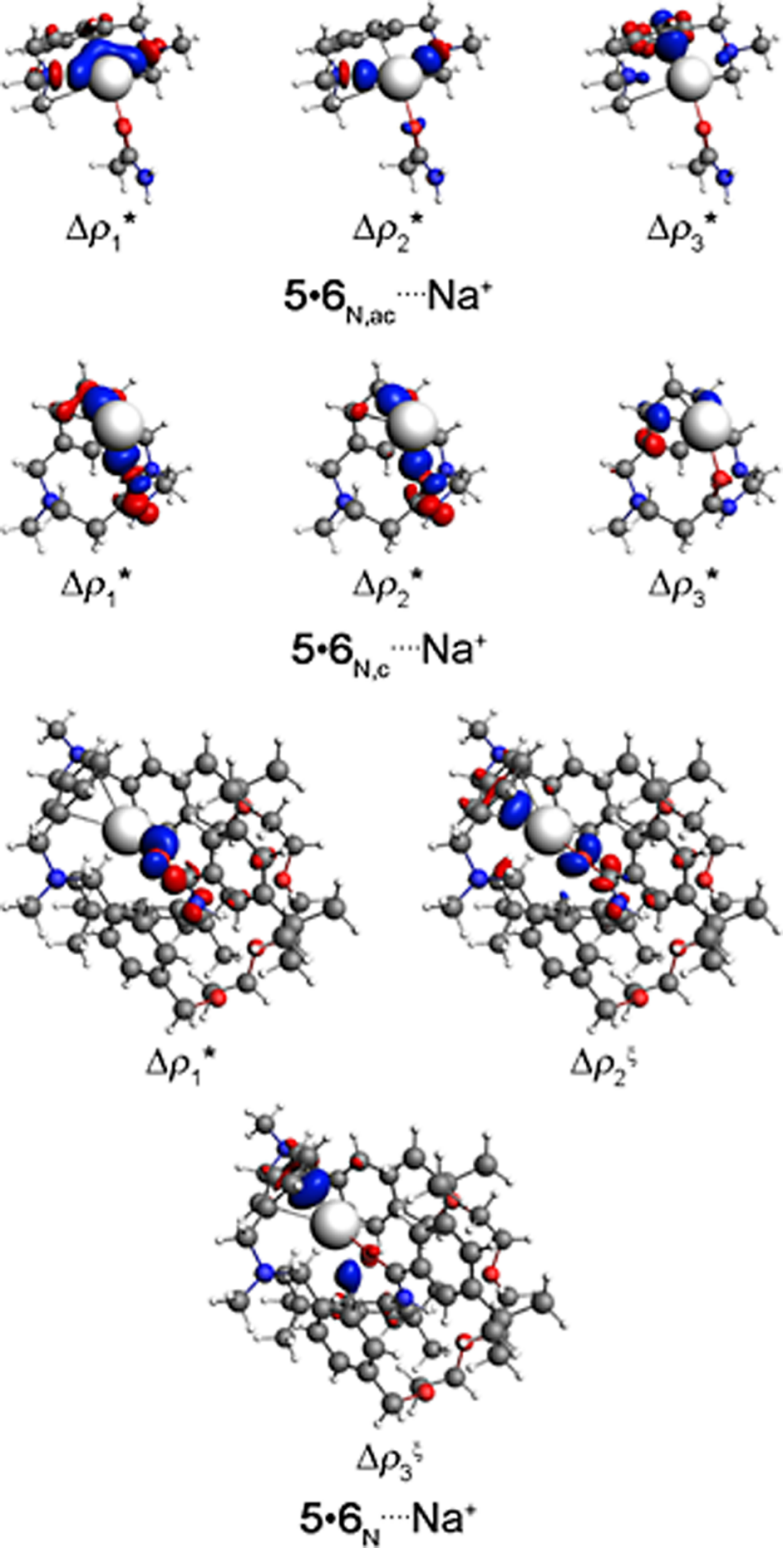
Main density deformation channel surface plots with isovalues = ξ 0.005 and * 0.001 au, where the red and blue regions indicate the electron density outflow and inflow, respectively, for (**5**•**6**_N,ac_, **5**•**6**_N,c_, or **5**•**6**_N_)····Na^+^ complexes. Color code for atoms: H = white; C = gray; N = blue; O = red; and Na = ice.

**Figure 7:**
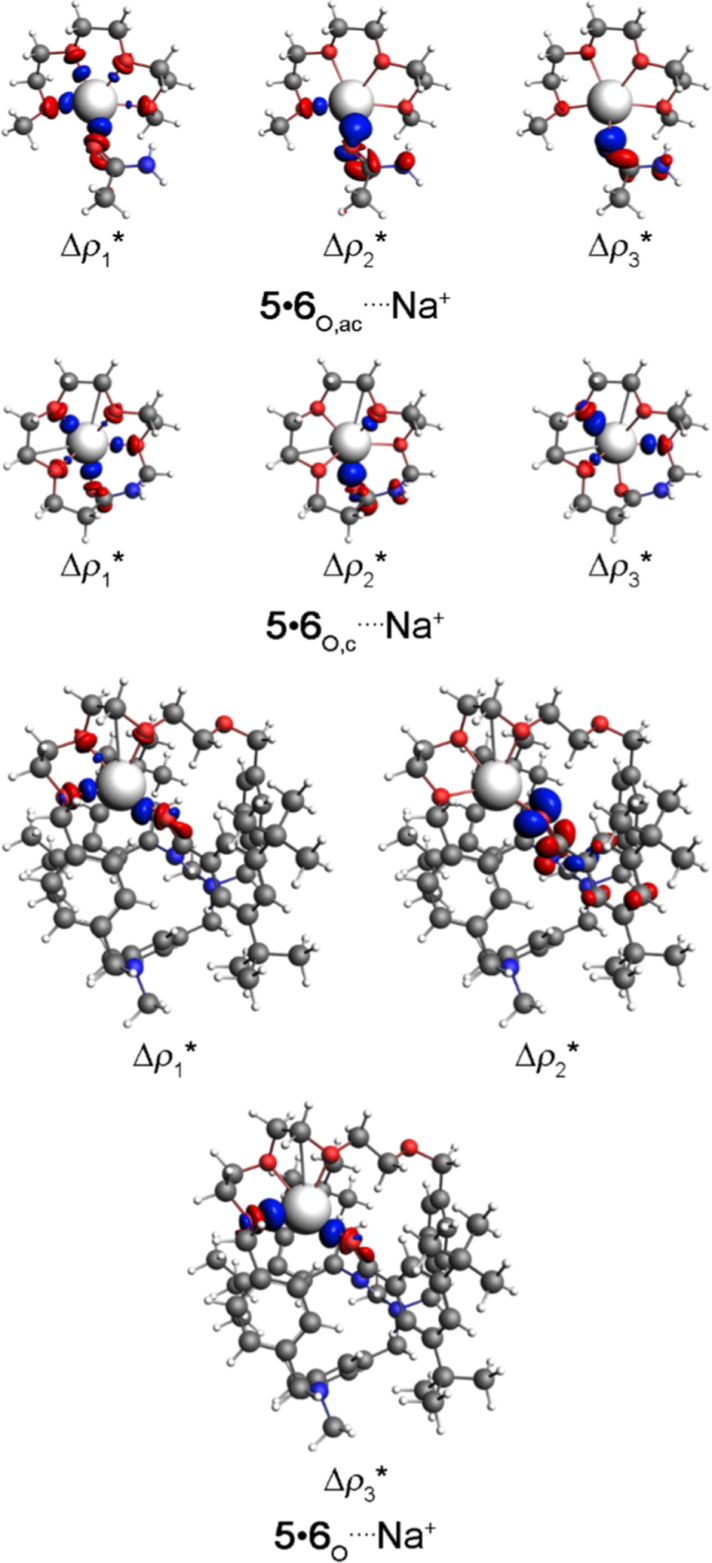
Main density deformation channel surface plots with isovalue = * 0.001 au, where the red and blue regions indicate the electron density outflow and inflow, respectively, for (**5**•**6**_O,ac_, **5**•**6**_O,c_, or **5**•**6**_O_)····Na^+^ complexes. Color code for atoms: H = white; C = gray; N = blue; O = red; and Na = ice.

The NOCV methodology indicates that the **5**•**6**_N,ac_····Na^+^ complex is, mainly, supported by the σ (N or C)····Na^+^ and π C····Na^+^ orbital interactions (Figure 6). On the other hand, the (**5**•**6**_N,c_ or **5**•**6**_N_)····Na^+^ compounds are, chiefly, maintained by the π (C or O)····Na^+^ orbital interactions (Figure 6). Interestingly, experimental results also indicated that the Na^+^ ion is coordinated to the **5**•**6**_N_ receptor through the C=O group present in the **5** component (Figure 2).^33^ The NOCV data suggest that the orbital interactions with less significant energy are decisive to point out the Δ*E*_oi_ energy order in the (**5**•**6**_N,ac_, **5**•**6**_N,c_, or **5**•**6**_N_)····Na^+^ complexes. To (**5**•**6**_O,ac_, **5**•**6**_O,c_, or **5**•**6**_O_)····Na^+^ molecules is possible to visualize the presence of the σ and π O····Na^+^ orbital interactions (Figure 7). In this case, the more favorable σ and π O····Na^+^ orbital interactions in the **5**•**6**_O_····Na^+^ structure concerning the (**5**•**6**_O,ac_, or **5**•**6**_O,c_)····Na^+^ compounds explain the Δ*E*_oi_ energy order (Table 2). The QTAIM method shows that the (**5**•**6**_N,ac_, **5**•**6**_N,c_, **5**•**6**_N_, **5**•**6**_O,ac_, **5**•**6**_O,c_, or **5**•**6**_O_)····Na^+^ complexes are supported by predominantly non-covalent (H, C, N, or, mainly, O)····Na^+^ interactions (Figure S21 and Table S6). The Δρ_b_ values at (C or O)····Na^+^ BCPs are similar or larger (in agreement with the more attractive Δ*E*_oi_ energy) in the **5**•**6**_N_····Na^+^ and **5**•**6**_O_····Na^+^ complexes concerning the (**5**•**6**_N,ac_, or **5**•**6**_N,c_)····Na^+^ and (**5**•**6**_O,ac_, or **5**•**6**_O,c_)····Na^+^ structures, respectively (Tables S6 and 2). The VDD method demonstrates that the receptor structure exhibits a greater charge donation to the Na^+^ cation, which is consistent with the presence of more attractive orbital interactions in the **5**•**6**_N_····Na^+^ and **5**•**6**_O_····Na^+^ molecules compared to the (**5**•**6**_N,ac_ or **5**•**6**_N,c_)····Na^+^ and (**5**•**6**_O,ac_ or **5**•**6**_O,c_)····Na^+^ compounds, respectively. The VDD results also show relevantly negative VDD charges to oxygen atoms present in the receptor structures (**5**•**6**_N,ac_, **5**•**6**_N,c_, **5**•**6**_N_, **5**•**6**_O,ac_, **5**•**6**_O,c_, or **5**•**6**_O_) in the (**5**•**6**_N,ac_, **5**•**6**_N,c_, **5**•**6**_N_, **5**•**6**_O,ac_, **5**•**6**_O,c_, or **5**•**6**_O_)····Na^+^ compounds. It indicates that these oxygen atoms are the most relevant atoms of the receptor molecules involved with the charge transfer with the Na^+^ cation. The average distance O····Na^+^ is equal to 2.149 and 2.737 Å for the (**5**•**6**_N,ac_, **5**•**6**_N,c_, or **5**•**6**_N_)····Na^+^ and (**5**•**6**_O,ac_, **5**•**6**_O,c_, or **5**•**6**_O_)····Na^+^ molecules, respectively.

### Relevance of the Alkali Metal Cation Nature in the Rotaxane····Cation Bond

The attractive interactions between rotaxanes (**5**•**6**_N_ or **5**•**6**_O_) and other alkali metal cations (Li^+^ or K^+^) show a largely non-covalent nature, as do the (**5**•**6**_N_ or **5**•**6**_O_)····Na^+^ interactions. The decrease in the alkali metal cation size (K^+^ → Na^+^ → Li^+^) favors the interaction with rotaxanes (**5**•**6**_N_ or **5**•**6**_O_) due to more favorable electrostatic and orbital interactions. The NOCV methodology indicates that the **5**•**6**_N_ receptor recognizes the Li^+^ and K^+^ cations through the σ N····(Li^+^ or K^+^) and σ and π (C or O)····(Li^+^ or K^+^) orbital interactions (Figure S15). However, the **5**•**6**_O_ molecule recognizes the Li^+^ and K^+^ cations by the σ and π (C or O)····(Li^+^ or K^+^) orbital interactions (Figure S15). The decrease of the cation size (K^+^ → Na^+^ → Li^+^) promotes more attractive σ and π orbital interactions between the C/N/O atoms of the **5**•**6**_N_ or **5**•**6**_O_ structures and the ion (Table 2). The QTAIM method indicates that (**5**•**6**_N_ or **5**•**6**_O_)····(Li^+^ or K^+^) structures are kept by chiefly non-covalent (H, C, N, or O)····(Li^+^ or K^+^) interactions (Figure S22 and Table S7). The ρ_b_ values at C=O····cation BCPs increase (in agreement with more attractive Δ*E*_oi_ energy, see Tables S7 and 2) with the decrease of the cation size.

The VDD method points out that the **5**•**6**_N_ or **5**•**6**_O_ receptor structures are donating more charge to ions with the decrease of the cation size (K^+^ → Na^+^ → Li^+^). It agrees with the increasingly favorable orbital interactions present in the following complexes trend (**5**•**6**_N_ or **5**•**6**_O_)····K^+^ → (**5**•**6**_N_ or **5**•**6**_O_)····Na^+^ → (**5**•**6**_N_ or **5**•**6**_O_)····Li^+^ (Table 2). Similar to (**5**•**6**_N_ or **5**•**6**_O_)····Na^+^ interactions, in the (**5**•**6**_N_ or **5**•**6**_O_)····(Li^+^ or K^+^) bonds, oxygen atoms appear as the most relevant atoms of the rotaxanes that are associated with charge transfer for the Li^+^ or K^+^ cations. The cation K^+^ shows O····ion average distances in the (**5**•**6**_N_ or **5**•**6**_O_)····K^+^ complexes equal to 2.787 and 3.219 Å, respectively, while the Li^+^ ion in the (**5**•**6**_N_ or **5**•**6**_O_)····Li^+^ structures have O····ion average distances equal to 1.983 and 2.190 Å, respectively.

### Preferential Binding Mode of Rotaxanes Regarding Alkali Metal Cations

The rotaxane **5**•**6** can provide two different conformations for interacting with alkali metal cations (Li^+^, Na^+^, or K^+^). The **5**•**6**_N_ structure furnishes, mainly, nitrogen atoms, while the **5**•**6**_O_ compound provides, chiefly, oxygen atoms to recognize the Li^+^, Na^+^, or K^+^ cations. The **5**•**6**_O_ molecule interacts in a more attractive way with the Li^+^, Na^+^, or K^+^ cations compared to the **5**•**6**_N_ compound supported by (i) more favorable electrostatic interactions and/or (ii) less repulsive chemical environment (lower Pauli repulsion) (Table 2).

### Regulation of the Rotaxane····Alkali Metal Cation Bond through Structural Changes

The substitution of hydrogen atoms by electron donor (−NH_2_) or electron acceptor (−NO_2_) groups in the **5**•**6**_N_ and **5**•**6**_O_ structures has been realized aiming to improve the interaction with the Na^+^ cation (Figures 2 and 4). The EDA results show that interactions between the substituted receptor compounds (**5**•**6**_N_(a–f) and **5**•**6**_O_(a–f)) and Na^+^ cation have a mostly non-covalent character.

The structural changes that favor the Na^+^ recognition are (i) −H → −NH_2_ substitutions in the −R^2^ position of the **5**•**6**_N_ molecule producing the **5**•**6**_N_(b) compound; (ii) −H → −NO_2_ substitutions in the −R^3^ position of the **5**•**6**_N_ structure leading to the **5**•**6**_N_(d) structure; (iii) −H → −NH_2_ substitutions in the −R^1,2^ positions of the **5**•**6**_O_ compound leading to **5**•**6**_O_(a) or **5**•**6**_O_(b) molecules; and (iv) −H → −NH_2_ substitutions in the −R^4^ position of the **5**•**6**_O_ structure creating the **5**•**6**_O_(e) receptor. It occurs due to (i) more attractive electrostatic and/or orbital interactions and/or (ii) less repulsive chemical ambiance (lower Pauli repulsion).

The NOCV method indicates that the main orbital interactions present between the substituted receptors: **5**•**6**_N_(a–f)s and Na^+^ cation are σ and π (C, N or O)····Na^+^ (Figures S16 and S17). In addition, the main orbital interactions present between the substituted receptors: **5**•**6**_O_(a–f) and Na^+^ cation are σ (C or N)····Na^+^ and σ and π O····Na^+^ (Figures S18 and S19). In general, the similar or more attractive orbital interactions in the **5**•**6**_N_(a–f)····Na^+^ and **5**•**6**_O_(a–f)····Na^+^ complexes compared to **5**•**6**_N_····Na^+^ and **5**•**6**_O_····Na^+^ structures, respectively, are supported by more favorable σ or π (C, N or O)····Na^+^ bonds. The QTAIM method shows that between the substituted receptors (**5**•**6**_N_(a–f) or **5**•**6**_O_(a–f)) and cation (Na^+^) there are largely non-covalent (H, C, N, or O)····Na^+^ bonds (Figure S23 and Table S8). All-proposed substitutions in the **5**•**6**_N_ molecule, **5**•**6**_N_(a–f), favor the increase of the Δρ_b_ values, associated with all these (H, C, N, or O)····Na^+^ interactions, in agreement with the more attractive Δ*E*_oi_ energy in the **5**•**6**_N_(a–f)····Na^+^ complexes compared to **5**•**6**_N_····Na^+^ compound (Tables S6, S8, S5 and 2). In general, the substituted molecules of **5**•**6**_O_, **5**•**6**_O_(a–f), not relevantly change or increase the Δρ_b_ values related to (H, C, N, or O)····Na^+^ bonds, which agree with the similar or more attractive Δ*E*_oi_ energy in the **5**•**6**_O_(a–f)····Na^+^ structures compared to the **5**•**6**_O_····Na^+^ complex (Tables S6, S9, S5 and 2). There is no clear correlation between the NOCV and VDD data in the complexes: (i) **5**•**6**_N_····Na^+^ and **5**•**6**_N_(a–f)····Na^+^ and (ii) **5**•**6**_O_····Na^+^ and **5**•**6**_O_(a–f)····Na^+^. The VDD method indicates that the charge transfer between the substituted receptors: **5**•**6**_N_(a–f) or **5**•**6**_O_(a–f), and the Na^+^ cation occurs mainly via oxygen atoms, so as in the **(5**•**6**_N_ or **5**•**6**_O_)····Na^+^ complexes. The O····Na^+^ average distances in the **5**•**6**_N_(a–f)····Na^+^ and **5**•**6**_O_(a–f)····Na^+^ complexes show values equal to 2.572 and 3.101 Å, respectively.

### Adjusting the Rotaxane····Alkali Metal Cation Bond Supported by Chemical Reduction

To analyze the relevance of the receptor charge to receptor····alkali metal cation bond, the interaction between the chemical-reduced receptors (**5**•**6**_N_^–^ or **5**•**6**_O_^–^) and cation (Na^+^) will be compared concerning the chemical bond between the neutral receptors (**5**•**6**_N_ or **5**•**6**_O_) and cation (Na^+^). The (**5**•**6**_N_^–^ or **5**•**6**_O_^–^)····Na^+^ bonds have a chiefly non-covalent nature. The chemical reduction of the receptors structure (**5**•**6**_N_ or **5**•**6**_O_ → **5**•**6**_N_^–^ or **5**•**6**_O_^–^, respectively) stabilizes the interaction with the Na^+^ cation due to more attractive (i) electrostatic and (ii) orbital interactions. The NOCV methodology indicates that the main orbital interactions present in the (**5**•**6**_N_^–^ or **5**•**6**_O_^–^)····Na^+^ compounds, similar to (**5**•**6**_N_ or **5**•**6**_O_)····Na^+^ molecules, are σ and π (C and O)····Na^+^ (Figure S20). Furthermore, the NOCV data show that the chemical reduction of the receptor structures improves the σ and π (C or O)····Na^+^ orbital interactions.

The QTAIM method shows that between the chemical reduced receptors (**5**•**6**_N_^–^ or **5**•**6**_O_^–^) and cation (Na^+^) there are largely non-covalent (C or, mainly, O)····Na^+^ interactions (Figure S25 and Table S10). The Δρ_b_ values associated with (C or, mainly, O)····Na^+^ bonds in the (**5**•**6**_N_^–^ or **5**•**6**_O_^–^)····Na^+^ structures are larger or close (in agreement with the more attractive Δ*E*_oi_ energy) than in the (**5**•**6**_N_ or **5**•**6**_O_)····Na^+^ complexes (Tables S5, S6 and S10). The VDD method indicates that the reduced compounds (**5**•**6**_N_^–^ or **5**•**6**_O_^–^) are donating more charge to Na^+^ cation in relation to neutral molecules: **5**•**6**_N_ or **5**•**6**_O_, respectively, which agrees with the more attractive orbital interactions in (**5**•**6**_N_^–^ or **5**•**6**_O_^–^)····Na^+^ compared to (**5**•**6**_N_ or **5**•**6**_O_)····Na^+^. In addition, as neutral receptors: **5**•**6**_N_ or **5**•**6**_O_, the reduced rotaxanes: **5**•**6**_N_^–^ or **5**•**6**_O_^–^, promote a charge transfer to Na^+^ cation chiefly through oxygen atoms. The O····Na^+^ average distances in the (**5**•**6**_N_^–^ or **5**•**6**_O_^–^)····Na^+^ compounds are equal to 2.283 and 2.360 Å, respectively.

## Conclusions

The rotaxanes facilitate the formation of more stable bonds with transition metal or alkali cations in comparison with the cyclic derivative compounds. This phenomenon is also observed in the formation of unrestricted chemical bonds in environments in which acyclic derivative molecules are present. The rotaxanes preferably recognize (i) transition metal cations through more attractive electrostatic and σ N····cation bonds and (ii) alkali metal cations due to more favorable dispersion and σ and π (C, N, or O)····cation interactions. It is a noteworthy conclusion as a recent study indicated that another class of MIMs, catenanes, demonstrated a diminished capacity to interact with the alkali metal cation. The interaction of Na^+^ with acyclic or cyclic molecular derivative structures is also worthy of note. The recognition of smaller cations by rotaxanes is facilitated by the formation of more favorable electrostatic and σ and/or π (C, N, or O)····cation bonds.

Importantly, the interaction between rotaxanes and transition metal cation (Zn^2+^) can be enhanced via (i) −H → −NH_2_ substitutions and (ii) chemical reduction of the **1**•**4**, **2**•**4**, and **3**•**4** compounds through more favorable electrostatic and σ or π (C, N, or O)····cation bonds. Structural changes in the **5**•**6**_N_ (−H → −NH_2_ substitutions in the −R^2^ position leading to **5**•**6**_N_(b)) and **5**•**6**_O_ (−H → −NH_2_ substitutions in the −R^4^ position making to **5**•**6**_O_(e)) rotaxanes can favor the interaction with alkali metal cation (Na^+^), chiefly, through more attractive electrostatic (C, N, or O)····cation interactions. The chemical reduction of the **5**•**6**_N_ or **5**•**6**_O_ molecules favors the Na^+^ recognition through more attractive electrostatic and σ and π (C or O)····Na^+^ orbital interactions.

The findings of this study demonstrate that rotaxanes possess a greater capacity to recognize cations in comparison to their acyclic and cyclic molecular derivatives. Furthermore, the present paper offers invaluable insights into the design of receptor structures with enhanced capabilities to recognize cations.

## Computational Methods

The geometry optimization of all compounds was realized without restrictions, and the vibrational frequencies were calculated using the following computational method: BLYP,^41−43^ including the dispersion corrections of Grimme along with the damping functions of Becke–Johnson: D3(BJ)^35^ and the Def2-TZVP basis set.^44^ The RIJCOSX^45,46^ approach was used to speed up the calculations. The Coulomb integral was approximated with the RI–J^45^ method using the auxiliary basis set: Def2/J.^47^ The analysis of the vibrational frequencies was realized to check that all the optimized geometries are in the minimum energy (no imaginary vibrational frequency) with regard to the theoretical model chosen. These calculations were performed using the ORCA software.^48^ The choice of the BLYP-D3(BJ)/triple-ζ basis set approach is suggested in the literature for the geometry optimization of compounds containing non-covalent interactions.^36^ The wave functions (for the QTAIM analysis) were obtained through the BLYP-D3(BJ)/Def2-TZVP theory level using the Gaussian 16 (Revision B.01) software.^49^ The topological analysis of the electron density was realized from the QTAIM^39,40,50^ method using the AIMAll (Version 17.01.25) software.^51^

The bonding situations were obtained through the EDA^34^–NOCV^37,38^ methodology and the VDD^52−56^ method. The VDD method supports the chemical bonding analysis of important studies.^57,58^ These calculations were performed by the Amsterdam density functional (ADF)^59,60^ software using the BLYP-D3(BJ) functional and the TZ2P basis set.^61^ Scalar relativistic corrections were included in a self consistent way through the zero-order regular approximation (ZORA).^62^ The ZORA-BLYP-D3(BJ)/TZ2P computational model demonstrated sufficient efficacy in elucidating the bonding situations involving non-covalent interactions.^63,64^

## Supporting Information Available

EDA–NOCV–VDD and QTAIM complete analysis, EDA–NOCV–VDD results for selected structures, QTAIM results, and optimized Cartesian coordinates of all complexes investigated (PDF)
