# Supplementary material for: Improved Skill of Rotaxanes to Recognize Cations: A Theoretical Perspective
Source: ACS Phys Chem Au. 2025 Jan 6;5(2):183–94. doi: 10.1021/acsphyschemau.4c00090 (PMC11950869; doi:10.1021/acsphyschemau.4c00090)
Supplement: Supplementary file 1 — pg4c00090_si_001.pdf [file pg4c00090_si_001.pdf]

The Improved Skill of Rotaxanes to Recognize Cations: A Theoretical Perspective

Renato Pereira Orenha,[a]* Alvaro Muñoz–Castro,[b] Maurício Jeomar Piotrowski,[c] Giovanni F. Caramori,[d]* Renato Gonçalves Rocha,[a] and Renato Luis Tame Parreira[a]*

[a] Núcleo de Pesquisas em Ciências Exatas e Tecnológicas, Universidade de Franca, Av. Dr. Armando de Sáles Oliveira 201, Franca, SP, 14404–600, Brazil. Email: rpo9@hotmail.com / renato.parreira@unifran.edu.br

[b] Facultad de Ingeniería, Arquitectura y Diseño, Universidad San Sebastián, Bellavista 7, Santiago, 8420524, Chile.

[c] Department of Physics, Federal University of Pelotas, Pelotas, RS, 96010–900, Brazil.

[d] Departamento de Química, Universidade Federal de Santa Catarina, Campus Universitário Trindade, CP 476, Florianópolis, SC, 88040–900, Brazil. Email: giovanni.caramori@ufsc.br

[§] R.P.O., A.M.–C., M.J.P., G.F.C., R.G.R. and R.L.T.P. contributed equally to this work

Supplementary Material

	Page
**EDA–NOCV–VDD Analysis**	4
**QTAIM Analysis**	9
**Figure S1.** The main density deformation channel surface plots with isovalues = ϕ 0.005 and * 0.001 a.u., where the red and blue regions indicate the electron density outflow and inflow, respectively, for (**2•4**$_{ac}$, **2•4**$_{c}$ or **2•4**)···Zn^{2+} complexes. Color code for atoms: H = white; C = gray; N = blue; O = red; and Zn = ice.	12
**Figure S2.** The main density deformation channel surface plots with isovalues = ϕ 0.005 and * 0.001 a.u., where the red and blue regions indicate the electron density outflow and inflow, respectively, for (**3•4**$_{ac}$, **3•4**$_{c}$ or **3•4**)···Zn^{2+} complexes. Color code for atoms: H = white; C = gray; N = blue; O = red; and Zn = ice.	13
**Figure S3.** The main density deformation channel surface plots with isovalues = ϕ 0.005 and * 0.001 a.u., where the red and blue regions indicate the electron density outflow and inflow, respectively, for (**1•4** or **2•4**)···Cd^{2+} complexes. Color code for atoms: H = white; C = gray; N = blue; O = red; and Cd = yellow.	14
**Figure S4.** The main density deformation channel surface plots with isovalues = ϕ 0.005 and * 0.001 a.u., where the red and blue regions indicate the electron density outflow and inflow, respectively, for **3•4**···Cd^{2+} complexes. Color code for atoms: H = white; C = gray; N = blue; O = red; and Cd = yellow.	15
**Table S1.** Analysis of the bonding situation between the substituted receptors (**1•4**a–d, **2•4**a–d or **3•4**a–d) and transition metal cation (Zn^{2+}) through of the EDA–NOCV methodology and study of the charge distribution proportioned from the VDD method. The units of energy and charge are kcal mol^{-1} and a.u., respectively.	16
**Figure S5.** The main density deformation channel surface plots with isovalues = ϕ 0.005 and * 0.001 a.u., where the red and blue regions indicate the electron density outflow and inflow, respectively, for **1•4**(a or b)···Zn^{2+} complexes. Color code for atoms: H = white; C = gray; N = blue; O = red; and Zn = ice.	17
**Figure S6.** The main density deformation channel surface plots with isovalues = ϕ 0.005 and * 0.001 a.u., where the red and blue regions indicate the electron density outflow and inflow, respectively, for **1•4**(c or d)···Zn^{2+} complexes. Color code for atoms: H = white; C = gray; N = blue; O = red; and Zn = ice.	18
**Figure S7.** The main density deformation channel surface plots with isovalues = ϕ 0.005 and * 0.001 a.u., where the red and blue regions indicate the electron density outflow and inflow, respectively, for **2•4**(a or b)···Zn^{2+} complexes. Color code for atoms: H = white; C = gray; N = blue; O = red; and Zn = ice.	19

**Figure S8.** The main density deformation channel surface plots with isovalues = $^{\phi}$ 0.005 and * 0.001 a.u., where the red and blue regions indicate the electron density outflow and inflow, respectively, for **2•4**(c or d)···Zn^{2+} complexes. Color code for atoms: H = white; C = gray; N = blue; O = red; and Zn = ice.	20
**Figure S9.** The main density deformation channel surface plots with isovalues = $^{\phi}$ 0.005 and * 0.001 a.u., where the red and blue regions indicate the electron density outflow and inflow, respectively, for **3•4**(a or b)···Zn^{2+} complexes. Color code for atoms: H = white; C = gray; N = blue; O = red; and Zn = ice.	21
**Figure S10.** The main density deformation channel surface plots with isovalues = $^{\phi}$ 0.005 and * 0.001 a.u., where the red and blue regions indicate the electron density outflow and inflow, respectively, for **3•4**(c or d)···Zn^{2+} complexes. Color code for atoms: H = white; C = gray; N = blue; O = red; and Zn = ice.	22
**Figure S11.** The main density deformation channel surface plots with isovalues = $^{\phi}$ 0.005; ∫ 0.003 and * 0.001 a.u., where the red and blue regions indicate the electron density outflow and inflow, respectively, for (**1•4**$^{-}$, **2•4**$^{-}$ or **3•4**$^{-}$)···Zn^{2+} complexes. Color code for atoms: H = white; C = gray; N = blue; O = red; and Zn = ice.	23
**Figure S12.** Topological map containing the bond paths (continuous or dashed lines connecting the cores) and bond critical points (small green points), for the complexes (**1•4**$_{ac}$, **2•4**$_{ac}$, **3•4**$_{ac}$, **1•4**$_{c}$, **2•4**$_{c}$, **3•4**$_{c}$, **1•4**, **2•4** or **3•4**)···(Zn^{2+} or Cd^{2+}). Atoms color code: H = white; C = gray; N = blue; O = red; Zn = purple; and Cd = yellow.	24
**Figure S13.** Topological map containing the bond paths (continuous or dashed lines connecting the cores) and bond critical points (small green points), for the complexes (**1•4**, **2•4** or **3•4**)a–d···Zn^{2+}. Atoms color code: H = white; C = gray; N = blue; O = red; and Zn = purple.	25
**Figure S14.** Topological map containing the bond paths (continuous or dashed lines connecting the cores) and bond critical points (small green points), for the complexes (**1•4**$^{-}$, **2•4**$^{-}$ or **3•4**$^{-}$)···Zn^{2+}. Atoms color code: H = white; C = gray; N = blue; O = red; and Zn = purple.	26
**Table S2.** Ratio between the kinetic energy density, G_b, and potential energy density, V_b, $-G_b/V_b$, and electron density, ρ_b, at BCPs related to interactions between the receptors (**1•4**$_{ac}$**,** **2•4**$_{ac}$**,** **3•4**$_{ac}$**,** **1•4**$_{c}$**,** **2•4**$_{c}$**,** **3•4**$_{c}$**,** **1•4,** **2•4** or **3•4**) and cation (Zn^{2+}). The values of all the parameters are in a.u.	27
**Table S3.** Ratio between the kinetic energy density, G_b, and potential energy density, V_b, $-G_b/V_b$, and electron density, ρ_b, at BCPs related to interactions between: i) the receptors (**1•4**, **2•4** or **3•4**) and cation (Cd^{2+}); and ii) the receptors (**1•4**$^{-}$, **2•4**$^{-}$ or **3•4**$^{-}$) and cation (Zn^{2+}). The values of all the parameters are in a.u.	28
**Table S4.** Ratio between the kinetic energy density, G_b, and potential energy density, V_b, $-G_b/V_b$, and electron density, ρ_b, at BCPs related to interactions between the receptors (**1•4**, **2•4** or **3•4**)(a–d) and cation (Zn^{2+}). The values of all the parameters are in a.u.	29
**Table S5.** Analysis of the bonding situation between the substituted receptors (**5•6**$_{N}$(a–f) or **5•6**$_{O}$(a–f)) and alkali metal cation (Na^{+}) through of the EDA–NOCV methodology and study of the charge distribution proportioned from the VDD method. The units of energy and charge are kcal mol^{-1} and a.u., respectively.	30
**Figure S15.** The main density deformation channel surface plots with isovalues = $^{\xi}$ 0.005 and * 0.001 a.u., where the red and blue regions indicate the electron density outflow and inflow, respectively, for (**5•6**$_{N}$ or **5•6**$_{O}$)···(Li^{+} or K^{+}) complexes. Color code for atoms: H = white; C = gray; N = blue; O = red; Li = light gray; and K = silver.	31
**Figure S16.** The main density deformation channel surface plots with isovalues = $^{\xi}$ 0.005 and * 0.001 a.u., where the red and blue regions indicate the electron density outflow and inflow, respectively, for **5•6**$_{N}$(a–d)···Na^{+} complexes. Color code for atoms: H = white; C = gray; N = blue; O = red; and Na = ice.	32
**Figure S17.** The main density deformation channel surface plots with isovalues = $^{\xi}$ 0.005 and * 0.001 a.u., where the red and blue regions indicate the electron density outflow and inflow, respectively, for **5•6**$_{N}$(e or f)···Na^{+} complexes. Color code for atoms: H = white; C = gray; N = blue; O = red; and Na = ice.	33
**Figure S18.** The main density deformation channel surface plots with isovalues = $^{\xi}$ 0.005 and * 0.001 a.u., where the red and blue regions indicate the electron density	34

outflow and inflow, respectively, for **5•6**$_{O}$(a–d)···Na^+ complexes. Color code for atoms: H = white; C = gray; N = blue; O = red; and Na = ice.

Figure S19. The main density deformation channel surface plots with isovalues = ξ 0.005 and * 0.001 a.u., where the red and blue regions indicate the electron density outflow and inflow, respectively, for **5•6**$_{O}$(e or f)···Na^+ complexes. Color code for atoms: H = white; C = gray; N = blue; O = red; and Na = ice. 35

Figure S20. The main density deformation channel surface plots with isovalues = ξ 0.005 and * 0.001 a.u., where the red and blue regions indicate the electron density outflow and inflow, respectively, for (**5•6**$_{N}^{-}$ or **5•6**$_{O}^{-}$)···Na^+ complexes. Color code for atoms: H = white; C = gray; N = blue; O = red; Na = ice. 36

Figure S21. Topological map containing the bond paths (continuous or dashed lines connecting the cores) and bond critical points (small green points), for the complexes (**5•6**$_{N,ac}$, **5•6**$_{N,c}$, **5•6**$_{N}$, **5•6**$_{O,ac}$, **5•6**$_{O,c}$ or **5•6**$_{O}$)···Na^+. Atoms color code: H = white; C = gray; N = blue; O = red; and Na = purple. 37

Figure S22. Topological map containing the bond paths (continuous or dashed lines connecting the cores) and bond critical points (small green points), for the complexes (**5•6**$_{N}$ or **5•6**$_{O}$)···(Li^+ or K^+). Atoms color code: H = white; C = gray; N = blue; O = red; Li = pink; and K = dark purple. 38

Figure S23. Topological map containing the bond paths (continuous or dashed lines connecting the cores) and bond critical points (small green points), for the complexes **5•6**$_{N}$(a–f)···Na^+. Atoms color code: H = white; C = gray; N = blue; O = red; and Na = purple. 39

Figure S24. Topological map containing the bond paths (continuous or dashed lines connecting the cores) and bond critical points (small green points), for the complexes **5•6**$_{O}$(a–f)···Na^+. Atoms color code: H = white; C = gray; N = blue; O = red; and Na = purple. 40

Figure S25. Topological map containing the bond paths (continuous or dashed lines connecting the cores) and bond critical points (small green points), for the complexes (**5•6**$_{N}^{-}$ or **5•6**$_{O}^{-}$)···Na^+. Atoms color code: H = white; C = gray; N = blue; O = red; and Na = purple. 41

Table S6. Ratio between the kinetic energy density, G_b, and potential energy density, V_b, $-G_b/V_b$, and electron density, ρ_b, at BCPs related to interactions between the receptors (**5•6**$_{N,ac}$, **5•6**$_{N,c}$, **5•6**$_{N}$, **5•6**$_{O,ac}$, **5•6**$_{O,c}$ or **5•6**$_{O}$) and cation (Na^+). The values of all the parameters are in a.u. 42

Table S7. Ratio between the kinetic energy density, G_b, and potential energy density, V_b, $-G_b/V_b$, and electron density, ρ_b, at BCPs related to interactions between the receptors (**5•6**$_{N}$ or **5•6**$_{O}$) and cations (Li^+ or K^+). The values of all the parameters are in a.u. 43

Table S8. Ratio between the kinetic energy density, G_b, and potential energy density, V_b, $-G_b/V_b$, and electron density, ρ_b, at BCPs related to interactions between the receptors (**5•6**$_{N}$(a–f)) and cation (Na^+). The values of all the parameters are in a.u. 44

Table S9. Ratio between the kinetic energy density, G_b, and potential energy density, V_b, $-G_b/V_b$, and electron density, ρ_b, at BCPs related to interactions between the receptors (**5•6**$_{O}$(a–f)) and cation (Na^+). The values of all the parameters are in a.u. 45

Table S10. Ratio between the kinetic energy density, G_b, and potential energy density, V_b, $-G_b/V_b$, and electron density, ρ_b, at BCPs related to interactions between the receptors (**5•6**$_{N}^{-}$ or **5•6**$_{O}^{-}$) and cation (Na^+). The values of all the parameters are in a.u. 46

Table S11. VDD atomic charges organized in a crescent order for all complexes analyzed in this study. 47

Table S12. Optimized Cartesian coordinates for the compounds investigated in this study using the BLYP–D3(BJ)/Def2–TZVP computational model. 189

EDA–NOCV–VDD Analysis

The Relevance of Mechanical Bonds in Rotaxanes for the Recognition of Transition Metal Cations

All interactions, (**1•4**$_{ac}$, **2•4**$_{ac}$, **3•4**$_{ac}$, **1•4**$_{c}$, **2•4**$_{c}$, **3•4**$_{c}$, **1•4**, **2•4** or **3•4**)···Zn^{2+}, show an attractive nature as indicated from the negative values of ΔE_{int} (Table 1). These chemical bonds have similar contributions of ΔV_{elstat} (39 – 50%) and ΔE_{oi} (48 – 58%), nevertheless a minority weight of ΔE_{disp} (2 – 3%) concerning the sum of all favorable energetic components: ΔV_{elstat} + ΔE_{oi} + ΔE_{disp}.

The interactions between the MIMs (**1•4**, **2•4** or **3•4**) and cation (Zn^{2+}) appear more attractive compared to bonds between the acyclic (**1•4**$_{ac}$, **2•4**$_{ac}$ or **3•4**$_{ac}$) or cyclic (**1•4**$_{c}$, **2•4**$_{c}$ or **3•4**$_{c}$) molecules and cation (Zn^{2+}) because of the, in general, more attractive ΔV_{elstat}, ΔE_{oi} and ΔE_{disp} energies in the (**1•4**, **2•4** or **3•4**)···Zn^{2+} interactions in relation to (**1•4**$_{ac}$, **2•4**$_{ac}$ or **3•4**$_{ac}$)···Zn^{2+} and (**1•4**$_{c}$, **2•4**$_{c}$ or **3•4**$_{c}$)···Zn^{2+} bonds. In addition, it occurs because: i) the **1•4**···Zn^{2+} interaction has a lower Pauli repulsion energy concerning the **1•4**$_{ac}$···Zn^{2+} and **1•4**$_{c}$···Zn^{2+} bonds.

The analysis of the energy sum related to density deformation channels, $\Delta E_{oi,1-6}$, shows, overall, the same trend discussed using the ΔE_{oi} energy. Exceptionally, the (**2•4**$_{c}$ or **3•4**$_{c}$)···Zn^{2+} interactions show more attractive values of the $\Delta E_{oi,1-6}$ energy regarding the (**2•4** or **3•4**)···Zn^{2+} bonds, respectively. It indicates that less relevant orbital interactions contribute to more attractive values of the ΔE_{oi} energy in the bonds (**2•4** or **3•4**)···Zn^{2+} bonds regarding the (**2•4**$_{c}$ or **3•4**$_{c}$)···Zn^{2+} interactions.

The VDD charge related to Zn^{2+} cation, q^{VDD}_{Cation}, show negative values (Table 1). It indicates that the Zn^{2+} cation is receiving more charge than donating to receptor structures (**1•4**$_{ac}$, **2•4**$_{ac}$, **3•4**$_{ac}$, **1•4**$_{c}$, **2•4**$_{c}$, **3•4**$_{c}$, **1•4**, **2•4** or **3•4**). There are less negative values of q^{VDD}_{Cation} in the (**1•4**, **2•4** or **3•4**)···Zn^{2+} complexes regarding to (**1•4**$_{ac}$, **2•4**$_{ac}$ or **3•4**$_{ac}$)···Zn^{2+} and (**1•4**$_{c}$, **2•4**$_{c}$ or **3•4**$_{c}$)···Zn^{2+} structures. Considering that there are more favorable values of ΔE_{oi} in the (**1•4**, **2•4** or **3•4**)···Zn^{2+} complexes in relation to (**1•4**$_{ac}$, **2•4**$_{ac}$ or **3•4**$_{ac}$)···Zn^{2+} and (**1•4**$_{c}$, **2•4**$_{c}$ or **3•4**$_{c}$)···Zn^{2+} compounds, these data indicate that the Zn^{2+} cation is donating more charge to MIMs (**1•4**, **2•4** or **3•4**) than to acyclic (**1•4**$_{ac}$, **2•4**$_{ac}$ or **3•4**$_{ac}$) or cyclic (**1•4**$_{c}$, **2•4**$_{c}$ or **3•4**$_{c}$) molecules in the chemical bond formation process: isolated receptor + isolated cation → receptor···cation complex.

The Influence of the Transition Metal Cation Nature in the Rotaxane···Cation Interaction

So as the (**1•4**, **2•4** or **3•4**)···Zn^{2+} interactions, the (**1•4**, **2•4** or **3•4**)···Cd^{2+} bonds also have close weights of ΔV_{elstat} (44 – 52%) and ΔE_{oi} (45 – 51%), but a smaller contribution of ΔE_{disp} (3 – 4%) regarding ΔV_{elstat} + ΔE_{oi} + ΔE_{disp}. The (**1•4**, **2•4** or **3•4**)···Zn^{2+} interactions are more attractive than the (**1•4**, **2•4** or **3•4**)···Cd^{2+} bonds due the more favorable ΔV_{elstat} and, chiefly, ΔE_{oi} energies. Further, it appears because of the lower Pauli repulsion energy in the (**2•4** or **3•4**)···Zn^{2+} bonds than (**2•4** or **3•4**)···Cd^{2+} interactions. In general, the analysis of the sum of the energy associated to main density deformation channels has the same tendency presented above through the ΔE_{oi} energy. Overall, the VDD method shows that in the (**1•4**, **2•4** or **3•4**)···Cd^{2+} complexes there are less negative values of q^{VDD}_{Cation} compared to (**1•4**, **2•4** or **3•4**)···Zn^{2+} molecules. In view of the less

attractive ΔE_{oi} energy in the (**1•4**, **2•4** or **3•4**)···Cd^{2+} structures regarding the (**1•4**, **2•4** or **3•4**)···Zn^{2+} complexes, these VDD data suggest that the receptor compounds (**1•4**, **2•4** or **3•4**) are donating less charge to Cd^{2+} than to Zn^{2+}.

Tuning the Rotaxane···Transition Metal Cation Interaction using Structural Changes

Initially, the EDA results show that the substituted receptors (**1•4**a–d, **2•4**a–d and **3•4**a–d) interact with the Zn^{2+} cation in an attractive way (Table S1). Overall, the interactions between: i) **1•4**a–d, **2•4**a–d or **3•4**a–d; and ii) Zn^{2+}, have similar weights of ΔV_{elstat} (34 – 51%) and ΔE_{oi} (47 – 62%) regarding to ΔV_{elstat} + ΔE_{oi} + ΔE_{disp}. All interactions (**1•4**a–d, **2•4**a–d or **3•4**a–d)···Zn^{2+} show a minority contribution of ΔE_{disp} (2 – 3%) in ΔV_{elstat} + ΔE_{oi} + ΔE_{disp}.

The substitutions of –H by: i) $–NH_2$ or $–NO_2$ in the $–R^1$ position of **1** in **1•4** (producing **1•4**a and **1•4**c, respectively); and ii) $–NH_2$ in the $–R^4$ position of **4** in **1•4** (making **1•4**b), favor the recognition of the Zn^{2+} cation. It is rationalized from the more attractive ΔV_{elstat} and ΔE_{oi} energies in the **1•4**a–c···Zn^{2+} interactions in relation to **1•4**···Zn^{2+} bond. On the other hand, the –H → $–NO_2$ substitution in the $–R^4$ position of **4** in **1•4** (creating **1•4**d) disfavor the interaction with the Zn^{2+} cation. This result is supported by the less favorable energy ΔV_{elstat} in the **1•4**d···Zn^{2+} interaction compared to **1•4**···Zn^{2+} bond.

The –H → $–NH_2$ substitutions in the position: i) $–R^2$ of **2**; ii) $–R^3$ of **3**; and iii) $–R^4$ of **4**, in **2•4** or **3•4** (producing **2•4**a or **3•4**a and **2•4**b or **3•4**b, respectively) improve the recognition of the Zn^{2+} cation. It is due to more attractive ΔV_{elstat} and ΔE_{oi} energies in the interactions: i) **2•4**a···Zn^{2+} and **2•4**b···Zn^{2+}; and ii) **3•4**a···Zn^{2+} and **3•4**b···Zn^{2+}, regarding: i) **2•4**···Zn^{2+}; and ii) **3•4**···Zn^{2+}, respectively.

The –H → $–NO_2$ substitutions in the position: i) $–R^2$ of **2** in **2•4** (making **2•4**c); ii) $–R^3$ of **3** in **3•4** (leading to **3•4**c); or iii) $–R^4$ of **4** in **2•4** or **3•4** (creating **2•4**d or **3•4**d, respectively), disfavor the interaction with the Zn^{2+} cation. It is because of the less attractive ΔV_{elstat} energy in the **2•4**(c or d)···Zn^{2+} and **3•4**(c or d)···Zn^{2+} bonds in relation to **2•4**···Zn^{2+} and **3•4**···Zn^{2+} interactions, respectively. In addition, it is due the more repulsive ΔE_{Pauli} energy in the **2•4**c···Zn^{2+} and **3•4**c···Zn^{2+} interactions compared to **2•4**···Zn^{2+} and **3•4**···Zn^{2+} bonds, respectively.

As the whole, the analysis of the sum of the energy associated to main density deformation channels shows the same trend from the discussion using the ΔE_{oi} energy. In general, the VDD method shows that in the substituted complexes: (**1•4**, **2•4** or **3•4**)(a–d)···Zn^{2+}, there are more negative values of q^{VDD}_{Cation} concerning the non–substituted structures: (**1•4**, **2•4** or **3•4**)···Zn^{2+}.

Improving the Rotaxane···Transition Metal Cation Interaction via Chemical Reduction

The EDA results show that, similarity to (**1•4**, **2•4** or **3•4**)···Zn^{2+} bonds, the (**1•4**$^–$, **2•4**$^–$ or **3•4**$^–$)···Zn^{2+} interactions have close contributions of the ΔV_{elstat} (52 – 56%) and ΔE_{oi} (41 – 46%) energies, but a minority weight of the ΔE_{disp} (2%) energy to sum of all attractive energy terms: ΔV_{elstat} + ΔE_{oi} + ΔE_{disp} (Table 1).

The chemical reduction of the receptors structure (**1•4**, **2•4** or **3•4** → **1•4**$^–$, **2•4**$^–$ or **3•4**$^–$, respectively) favors the interaction with the Zn^{2+} cation. It occurs because there are more

favorable ΔV_{elstat} and ΔE_{oi} energies in the (**1•4**$^{-}$, **2•4**$^{-}$ or **3•4**$^{-}$)$\cdots$$Zn^{2+}$ bonds concerning the (**1•4**, **2•4** or **3•4**)$\cdots$$Zn^{2+}$ interactions. In addition, the **1•4**$^{-}$$\cdots$$Zn^{2+}$ bond is supported by a less repulsive ΔE_{Pauli} energy compared to **1•4**$\cdots$$Zn^{2+}$ interaction. The values of the sum of the energy associated to main density deformation channels point out to same tendency obtained above in terms of the discussion using the ΔE_{oi} energy (Table 1). The VDD method indicates that in the chemical reduced complexes: (**1•4**$^{-}$, **2•4**$^{-}$ or **3•4**$^{-}$)$\cdots$$Zn^{2+}$, there are close or more negative values of q_{Cation}^{VDD} concerning the neutral compounds: (**1•4**, **2•4** or **3•4**)$\cdots$$Zn^{2+}$.

The Importance of Mechanical Bonds in Rotaxanes for the Recognition of Alkali Metal Cations

All interactions, (**5•6**$_{N,ac}$, **5•6**$_{N,c}$, **5•6**$_{N}$, **5•6**$_{O,ac}$, **5•6**$_{O,c}$ or **5•6**$_{O}$)$\cdots$$Na^{+}$, have a favorable character as shown from the negative values of the ΔE_{int} energy. These chemical bonds have larger contribution of ΔV_{elstat} (48 – 66%) regarding ΔE_{oi} (26 – 37%) and ΔE_{disp} (8 – 15%) regarding the sum of all attractive energy terms: ΔV_{elstat} + ΔE_{oi} + ΔE_{disp}.

The **5•6**$_{N}$ and **5•6**$_{O}$ MIMs preferably interact with the Na^{+} cation than its acyclic (**5•6**$_{N,ac}$ and **5•6**$_{O,ac}$, respectively) or cyclic (**5•6**$_{N,c}$ and **5•6**$_{O,c}$, respectively) molecular derivatives. This trend is supported by more favorable ΔE_{oi} and ΔE_{disp} energies in the (**5•6**$_{N}$ or **5•6**$_{O}$)$\cdots$$Na^{+}$ interactions compared to (**5•6**$_{N,ac}$, **5•6**$_{O,ac}$, **5•6**$_{N,c}$ or **5•6**$_{O,c}$)$\cdots$$Na^{+}$ bonds. Furthermore, the (**5•6**$_{N}$ or **5•6**$_{O}$)$\cdots$$Na^{+}$ bonds have: i) less repulsive ΔE_{Pauli} energy concerning the (**5•6**$_{N,ac}$ or **5•6**$_{O,ac}$)$\cdots$$Na^{+}$ interactions; and ii) more attractive ΔV_{elstat} energy than the (**5•6**$_{N,c}$ or **5•6**$_{O,c}$)$\cdots$$Na^{+}$ interactions.

The sum of the energy values related to main density deformation channels, **5•6**$_{N,ac}$$\cdots$$Na^{+}$ < **5•6**$_{N,c}$$\cdots$$Na^{+}$ < **5•6**$_{N}$$\cdots$$Na^{+}$, not show the same trend visualized above regarding the discussion made from the ΔE_{oi} energy, **5•6**$_{N}$$\cdots$$Na^{+}$ < **5•6**$_{N,c}$$\cdots$$Na^{+}$ < **5•6**$_{N,ac}$$\cdots$$Na^{+}$. The VDD charge of the Na^{+} cation, $q_{Cation}{}^{VDD}$, in the (**5•6**$_{N,ac}$, **5•6**$_{N,c}$, **5•6**$_{N}$, **5•6**$_{O,ac}$, **5•6**$_{O,c}$ or **5•6**$_{O}$)$\cdots$$Na^{+}$ complexes has negative values (Table 2). It shows that the ion is receiving more charge than donating to receptor structure. More negative $q_{Cation}{}^{VDD}$ values (in agreement with more attractive ΔE_{oi} energy) are observed in the **5•6**$_{N}$$\cdots$$Na^{+}$ and **5•6**$_{O}$$\cdots$$Na^{+}$ molecules compared to (**5•6**$_{N,ac}$ or **5•6**$_{N,c}$)$\cdots$$Na^{+}$ and (**5•6**$_{O,ac}$ or **5•6**$_{O,c}$)$\cdots$$Na^{+}$ compounds, respectively.

The Relevance of the Alkali Metal Cation Nature in the Rotaxane$\cdots$Cation Bond

The attractive interactions between rotaxanes (**5•6**$_{N}$ or **5•6**$_{O}$) and other alkali metal cations (Li^{+} or K^{+}) also have a largely weight of ΔV_{elstat} (50 – 57%) in relation to ΔE_{oi} (32 – 42%) and ΔE_{disp} (7 – 16%) in ΔV_{elstat} + ΔE_{oi} + ΔE_{disp}. The decrease of the alkali metal cation size (K^{+} → Na^{+} → Li^{+}) favors the interaction with the rotaxanes (**5•6**$_{N}$ or **5•6**$_{O}$). It occurs due more attractive ΔV_{elstat} and ΔE_{oi} energies in the (**5•6**$_{N}$ or **5•6**$_{O}$)$\cdots$$Li^{+}$ bonds compared to (**5•6**$_{N}$ or **5•6**$_{O}$)$\cdots$(Na^{+} or K^{+}) interactions (Table 2). The VDD charge of the Li^{+} and K^{+} cations show negative values indicating that these ions, so as the Na^{+} cation, are receiving more charge than donating to **5•6**$_{N}$ or **5•6**$_{O}$ receptor structures (Table 2). More negative values of $q_{Cation}{}^{VDD}$ (so as more favorable ΔE_{oi} energy) are observed with the decrease of the cation size (K^{+} → Na^{+} → Li^{+}).

The Preferential Binding Mode of Rotaxanes regarding Alkali Metal Cations

The **5•6**$_O$ molecule interacts in a more attractive way with the Li^+, Na^+ or K^+ cations compared to **5•6**$_N$ compound. It happens mostly because of the more favorable ΔV_{elstat} energy in the **5•6**$_O$···(Li^+, Na^+ or K^+) bonds in relation to **5•6**$_N$···(Li^+, Na^+ or K^+) interactions. Besides, less repulsive ΔE_{Pauli} energy contributes for the more attractive recognition of the Li^+ or K^+ cations by the **5•6**$_O$ receptor than through of the **5•6**$_N$ structure.

Regulation of the Rotaxane···Alkali Metal Cation Bond through of Structural Changes

The EDA results show that interactions between the substituted receptor compounds (**5•6**$_N$(a–f) and **5•6**$_O$(a–f)) and Na^+ cation have a larger contribution of ΔV_{elstat} (44 – 64%) regarding the weight of ΔE_{oi} (27 – 41%) and ΔE_{disp} (10 – 15%) in ΔV_{elstat} + ΔE_{oi} + ΔE_{disp}. It shows that the substituted receptor···Na^+ bonds have a mostly non–covalent character.

The –H → –NH_2 or –NO_2 substitutions in the –R^1 position of the **5•6**$_N$ compound leading to **5•6**$_N$(a) or **5•6**$_N$(c) molecules, respectively, disfavor or not relevantly change the interaction with the Na^+ cation (Table S5). It occurs because of the more repulsive ΔE_{Pauli} energy: i) in the **5•6**$_N$(a)···Na^+ interaction; and ii) that counterbalances the more attractive ΔV_{elstat} and ΔE_{oi} energies in the in the **5•6**$_N$(c)···Na^+ bond, concerning to **5•6**$_N$···Na^+ interaction.

The –H → –NH_2 substitutions in the –R^2 position of the **5•6**$_N$ molecule producing the **5•6**$_N$(b) compound favors the interaction with the Na^+ cation, while the –H → –NO_2 substitutions in the –R^2 position of the **5•6**$_N$ compound leading to **5•6**$_N$(d) molecule produces the opposite effect. It appears due to: i) more attractive ΔV_{elstat} and ΔE_{oi} energies in the **5•6**$_N$(b)···Na^+ bond; and ii) less favorable ΔV_{elstat} energy in the **5•6**$_N$(d)···Na^+ interaction, in relation to **5•6**$_N$···Na^+ bond.

The –H → –NH_2 substitutions in the –R^3 position of the **5•6**$_N$ structure creating the **5•6**$_N$(e) molecule disfavors the interaction with the Na^+ cation, while the –H → –NO_2 substitutions in the –R^3 position of the **5•6**$_N$ structure producing the **5•6**$_N$(d) compound makes the contrary result. It occurs due: i) more repulsive ΔE_{Pauli} energy, and less favorable ΔE_{disp} energy in the **5•6**$_N$(e)···Na^+ interaction; and ii) more attractive ΔV_{elstat} energy in the **5•6**$_N$(f)···Na^+ bond, concerning the **5•6**$_N$···Na^+ interaction.

The –H → –NH_2 substitutions in the –$R^{1,2}$ positions of the **5•6**$_O$ compound leading to **5•6**$_O$(a) or **5•6**$_O$(b) molecules, respectively, favor the interaction with the Na^+ cation supported, mainly, by less repulsive ΔE_{Pauli} energy in the **5•6**$_O$(a or b)···Na^+ interactions regarding the **5•6**$_O$···Na^+ bond. On the other hand, the –H → –NO_2 substitutions in the –$R^{1,2}$ positions of the **5•6**$_O$ molecule making the **5•6**$_O$(c) or **5•6**$_O$(d) compounds, respectively, disfavor the interaction with the Na^+ cation owing to less attractive ΔV_{elstat}, ΔE_{disp} and/or ΔE_{oi} energies in the **5•6**$_O$(c or d)···Na^+ bonds compared to **5•6**$_O$···Na^+ interaction.

The –H → –NH_2 substitutions in the –R^4 position of the **5•6**$_O$ structure creating the **5•6**$_O$(e) molecule produce a more attractive interaction with the Na^+ cation, while the –H → –NO_2 substitutions in the –R^4 position of **5•6**$_O$ leading to **5•6**$_O$(f) ensures the contrary result. It happens because of the: i) more favorable ΔV_{elstat} and ΔE_{oi} energies in the **5•6**$_O$(e)···Na^+ interaction; and ii)

less attractive ΔV_{elstat} and ΔE_{disp} energies in the **5•6**$_O$(f)···Na$^+$ bond, compared to **5•6**$_O$···Na$^+$ interaction.

In general, the sum of the energy values related to main density deformation channels has the same trend concerning the ΔE_{oi} energy order (Tables 2 and S5). There is no clear correlation between the $q_{Cation}{}^{VDD}$ and ΔE_{oi} values in the complexes: i) **5•6**$_N$···Na$^+$ and **5•6**$_N$(a–f)···Na$^+$; and ii) **5•6**$_O$···Na$^+$ and **5•6**$_O$(a–f)···Na$^+$.

Adjusting the Rotaxane···Alkali Metal Cation Bond supported by Chemical Reduction

The EDA data indicate that, similarity to (**5•6**$_N$ or **5•6**$_O$)···Na$^+$ interactions, the (**5•6**$_N{}^-$ or **5•6**$_O{}^-$)···Na$^+$ bonds also have a largely weight of ΔV_{elstat} (68 – 70%) in relation to ΔE_{oi} (22 – 24%) and ΔE_{disp} (8%) in ΔV_{elstat} + ΔE_{oi} + ΔE_{disp} (Table 2).

The chemical reduction of the receptors structure (**5•6**$_N$ or **5•6**$_O$ → **5•6**$_N{}^-$ or **5•6**$_O{}^-$, respectively) favors the interaction with the Na$^+$ cation. It occurs because there are more attractive ΔV_{elstat} and ΔE_{oi} energies in the (**5•6**$_N{}^-$ or **5•6**$_O{}^-$)···Na$^+$ interactions regarding the (**5•6**$_N$ or **5•6**$_O$)···Na$^+$ bonds. The values of the sum of the energy related to main density deformation channels point out to same trend visualized above in terms of the discussion using the ΔE_{oi} energy (Table 2). The VDD method shows that in the chemical reduced complexes: (**5•6**$_N{}^-$ or **5•6**$_O{}^-$)···Na$^+$, there are close or more negative values of q_{Cation}^{VDD} concerning the neutral compounds: (**5•6**$_N$ or **5•6**$_O$)···Na$^+$.

QTAIM Analysis

The Relevance of Mechanical Bonds in Rotaxanes for the Recognition of Transition Metal Cations

The topological analysis of the electron density, performed with the QTAIM method, shows bond critical points (BCPs)[39] between the receptors (**1•4**$_{ac}$, **2•4**$_{ac}$, **3•4**$_{ac}$, **1•4**$_{c}$, **2•4**$_{c}$, **3•4**$_{c}$, **1•4**, **2•4** or **3•4**) and cation (Zn^{2+}): (C, C–H or, mainly, N)···Zn^{2+} (Figure S12 and Table S2). Overall, the ratio between the kinetic energy density, G_b, and potential energy density, V_b, $-G_b/V_b$, at BCPs, related to (C, C–H or, mainly, N)···Zn^{2+} interactions, show values between 0.5 and 1.0, suggesting that these chemical bonds are partially covalent.[40] In general, the sum of the electron density, ρ_b, at these BCPs, $\Delta\rho_b$, increase from (**1•4**$_{ac}$, **2•4**$_{ac}$ or **3•4**$_{ac}$)···Zn^{2+} (0.298 – 0.336 a.u.) and (**1•4**$_{c}$, **2•4**$_{c}$ or **3•4**$_{c}$)···Zn^{2+} (0.226 – 0.290 a.u.) structures to (**1•4**, **2•4** or **3•4**)···Zn^{2+} (0.319 – 0.336 a.u.) complexes. These results agree with the more attractive ΔE_{oi} energy in the (**1•4**, **2•4** or **3•4**)···Zn^{2+} complexes in relation to (**1•4**$_{ac}$, **2•4**$_{ac}$ or **3•4**$_{ac}$)···Zn^{2+} and (**1•4**$_{c}$, **2•4**$_{c}$ or **3•4**$_{c}$)···Zn^{2+} molecules (Table 1).

The Influence of the Transition Metal Cation Nature in the Rotaxane···Cation Interaction

Similarity to (**1•4**, **2•4** or **3•4**)···Zn^{2+} compounds, it is possible visualize in the (**1•4**, **2•4** or **3•4**)···Cd^{2+} molecules the (C, C–H or, mainly, N)···Cd^{2+} BCPs (Figure S12 and Table S3). Values of $-G_b/V_b$ between 0.5 and 1.0 in the N···Cd^{2+} BCPs indicate that these interactions show a partially covalent character.[40] On the other hand, the values of $-G_b/V_b$ larger than 1.0 in the (C or C–H)···Cd^{2+} BCPs show that these bonds have a predominantly non–covalent nature.[40] The QTAIM method shows that there are larger values of $\Delta\rho_b$ in the (**1•4**, **2•4** or **3•4**)···Zn^{2+} (0.319 – 0.336 a.u.) complexes regarding the (**1•4**, **2•4** or **3•4**)···Cd^{2+} (0.278 – 0.317 a.u.) structures. These data agree with the more favorable ΔE_{oi} in the (**1•4**, **2•4** or **3•4**)···Zn^{2+} compounds in relation to (**1•4**, **2•4** or **3•4**)···Cd^{2+} molecules (Table 1).

Tuning the Rotaxane···Transition Metal Cation Interaction using Structural Changes

The QTAIM method shows that between the substituted receptors: (**1•4**, **2•4** or **3•4**)(a–d), and cation (Zn^{2+}), so as in the (**1•4**, **2•4** or **3•4**)···Zn^{2+} complexes, there are BCPs related to interactions: (C, C–H and, mainly, N)···Zn^{2+}, with values of $-G_b/V_b$ between 0.5 and 1.0 (Figure S13 and Table S4), indicating that these chemical bonds are partially covalent.[40] Overall, the values of $\Delta\rho_b$ associated to BCPs N···Zn^{2+} increase from **1•4**···Zn^{2+} (0.261 a.u.), **2•4**···Zn^{2+} (0.326 a.u.) and **3•4**···Zn^{2+} (0.336 a.u.) to **1•4**(a–d)···Zn^{2+} (0.234 – 0.291 a.u.), **2•4**(a–d)···Zn^{2+} (0.326 – 0.347 a.u.) and **3•4**(a–d)···Zn^{2+} (0.328 – 0.353 a.u.), respectively. It is in agreement with the more favorable ΔE_{oi} energy in the (**1•4**, **2•4** and **3•4**)(a–d)···Zn^{2+} interactions compared to (**1•4**, **2•4** and **3•4**)···Zn^{2+} bonds (Table 1).

Improving the Rotaxane···Transition Metal Cation Interaction via Chemical Reduction

There are between the chemical reduced receptors (**1•4**$^-$, **2•4**$^-$ or **3•4**$^-$) and cation (Zn^{2+}) only BCPs associated to N···Zn^{2+} interactions (Figure S14). Values of $–G_b/V_b$ between 0.5 and 1.0 in these BCPs (Table S3) indicated that the N···Zn^{2+} bonds show a partially covalent nature.[40] The values of the sum of ρ_b associated only to N···Zn^{2+} interactions in the (**1•4**$^-$, **2•4**$^-$ or **3•4**$^-$)···Zn^{2+} complexes (0.214 – 0.341 a.u.) are close or lower than in the (**1•4**, **2•4** or **3•4**)···Zn^{2+} structures (0.261 – 0.336 a.u.). These data suggest that the more favorable ΔE_{oi} energy in the (**1•4**$^-$, **2•4**$^-$ or **3•4**$^-$)···Zn^{2+} interactions regarding the (**1•4**, **2•4** or **3•4**)···Zn^{2+} bonds are supported by C···Zn^{2+} orbital interactions.

The Importance of Mechanical Bonds in Rotaxanes for the Recognition of Alkali Metal Cations

The QTAIM method indicates BCPs between the receptors (**5•6**$_{N,ac}$, **5•6**$_{N,c}$, **5•6**$_N$, **5•6**$_{O,ac}$, **5•6**$_{O,c}$ or **5•6**$_O$) and cation (Na^+): (H, C, N or, mainly, O)···Na^+ (Figure S21 and Table S6). The $–G_b/V_b$ values at (H, C, N or, mainly, O)···Na^+ BCPs show values larger than 1.0, signifying that these interactions are predominantly non–covalent.[40] The sum of the electron density at (C or O)···Na^+ BCPs is similar or larger in the **5•6**$_N$···Na^+ (0.043 a.u.) complex concerning the (**5•6**$_{N,ac}$ or **5•6**$_{N,c}$)···Na^+ (0.037 and 0.042 a.u., respectively) compounds. The electron density at C=O···Na^+ BCP of the **5•6**$_O$···Na^+ molecule (0.027 a.u.) is close or larger than in the **5•6**$_{O,ac}$···Na^+ (0.025 a.u.) and **5•6**$_{O,c}$···Na^+ (0.017 a.u.) structures. These data are in agreement with the more favorable ΔE_{oi} energy in the **5•6**$_N$···Na^+ and **5•6**$_O$···Na^+ complexes regarding the (**5•6**$_{N,ac}$ or **5•6**$_{N,c}$)···Na^+ and (**5•6**$_{O,ac}$ or **5•6**$_{O,c}$)···Na^+ compounds, respectively (Table 2).

The Relevance of the Alkali Metal Cation Nature in the Rotaxane···Cation Bond

To (**5•6**$_N$ or **5•6**$_O$)···(Li^+ or K^+) structures, there are BCPs associated to (H, C, N or O)···(Li^+ or K^+) interactions. The $–G_b/V_b$ values at these BCPs show values larger than 1.0, indicating that these interactions are chiefly non–covalent.[40] The ρ_b values at C=O···cation BCPs increase (in agreement with more attractive ΔE_{oi} energy, see Table 2) with the decrease of the cation size: i) **5•6**$_N$···K^+ (0.026 a.u.) → **5•6**$_N$···Na^+ (0.031 a.u.) → **5•6**$_N$···Li^+ (0.045 a.u.); and ii) **5•6**$_O$···K^+ (0.020 a.u.) → **5•6**$_O$···Na^+ (0.027 a.u.) → **5•6**$_O$···Li^+ (0.036 a.u.).

Regulation of the Rotaxane···Alkali Metal Cation Bond through of Structural Changes

There are between the substituted receptors (**5•6**$_N$(a–f) or **5•6**$_O$(a–f)) and cation (Na^+) (H, C, N or O)···Na^+ BCPs with $–G_b/V_b$ values larger than 1.0, which indicates the largely non–covalent nature of these chemical bonds.[40] All–proposed substitutions in the **5•6**$_N$ molecule, **5•6**$_N$(a–f), favor the increase of the $\Delta\rho_b$ values, associated to all these BCPs, in agreement with the more attractive ΔE_{oi} energy in the **5•6**$_N$(a–f)···Na^+ complexes (0.051 – 0.069 a.u. and –43.23 – –40.14 kcal mol^{-1}) compared to **5•6**$_N$···Na^+ compound (0.047 a.u. and –39.48 kcal mol^{-1}). In general, the substituted molecules of **5•6**$_O$, with exception of **5•6**$_O$(c), **5•6**$_O$(a, b and d–f), not relevantly change or increase the $\Delta\rho_b$ values, which agree with the similar or more attractive ΔE_{oi} energy in the

5•6$_O$(a, b and d–f)···Na^+ structures (0.073 – 0.099 a.u. and –42.89 – –40.35 kcal mol^{-1}) compared to **5•6**$_O$···Na^+ complex (0.080 a.u. and –40.27 kcal mol^{-1}). In the **5•6**$_O$(c)···Na^+ complex, there is a lower $\Delta\rho_b$ value (0.066 a.u.) and less favorable ΔE_{oi} energy (–38.26 kcal mol^{-1}) compared to **5•6**$_O$(a)···Na^+ compound.

Adjusting the Rotaxane···Alkali Metal Cation Bond supported by Chemical Reduction

There are between the chemical reduced receptors (**5•6**$_N^-$ or **5•6**$_O^-$) and cation (Na^+) BCPs related to (C or, mainly, O)···Na^+ bonds (Figure S25). Values of $-G_b/V_b$ larger than 1.0 in these BCPs (Table S10) show that the (C or, mainly, O)···Na^+ interactions have an largely non–covalent character.[40] The values of the sum of ρ_b associated only to (C or, mainly, O)···Na^+ bonds in the (**5•6**$_N^-$ or **5•6**$_O^-$)···Na^+ structures (0.054 – 0.080 a.u.) are larger or close than in the (**5•6**$_N$ or **5•6**$_O$)···Na^+ complexes (0.047 – 0.080 a.u.). These data suggest that the more favorable ΔE_{oi} energy in the (**5•6**$_N^-$ or **5•6**$_O^-$)···Na^+ bonds concerning the (**5•6**$_N$ or **5•6**$_O$)···Na^+ interactions are maintained by (C or, mainly, O)···Na^+ orbital interactions.

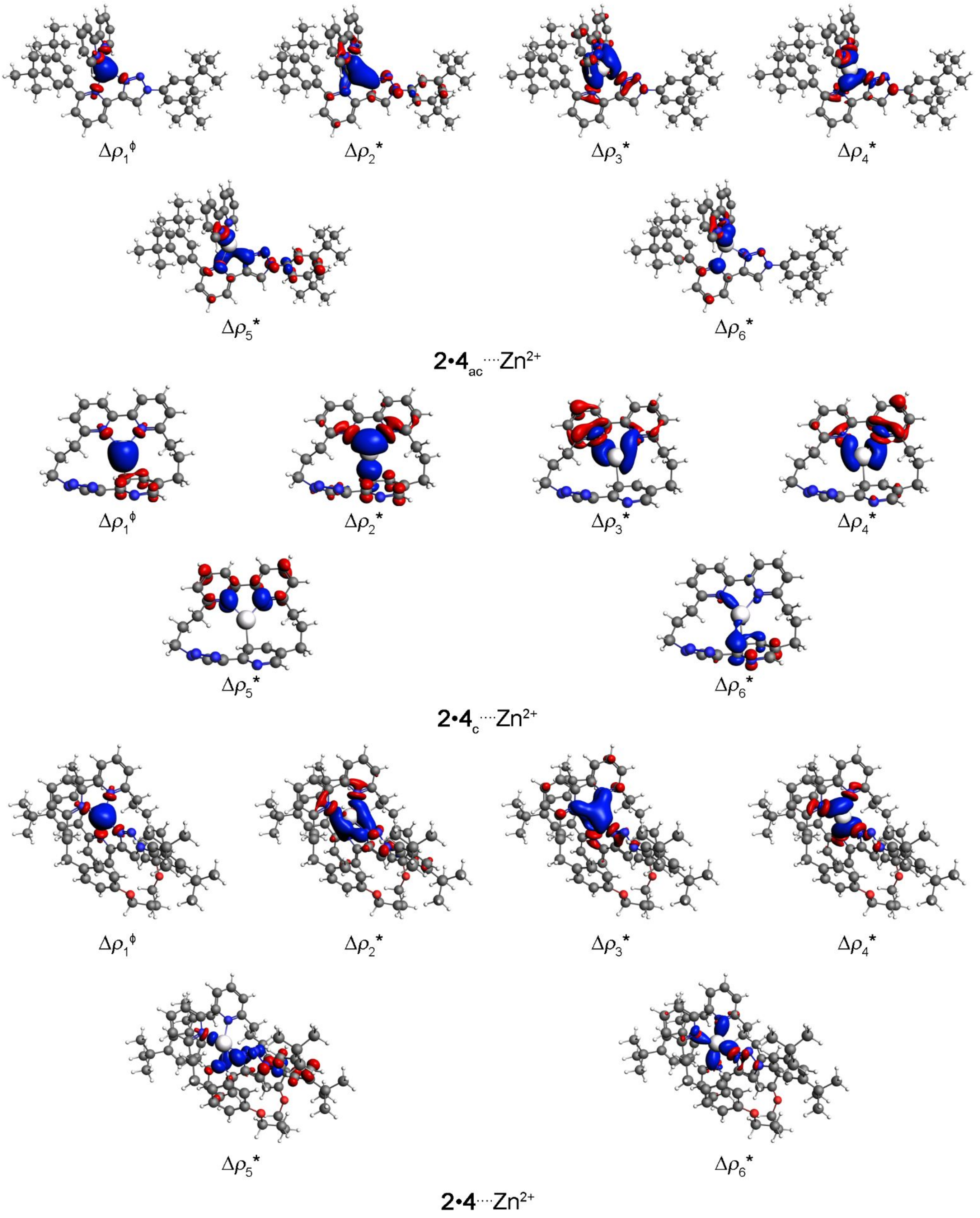

Figure S1. The main density deformation channel surface plots with isovalues = ϕ 0.005 and * 0.001 a.u., where the red and blue regions indicate the electron density outflow and inflow, respectively, for (**2•4**$_{ac}$, **2•4**$_{c}$ or **2•4**)···Zn^{2+} complexes. Color code for atoms: H = white; C = gray; N = blue; O = red; and Zn = ice.

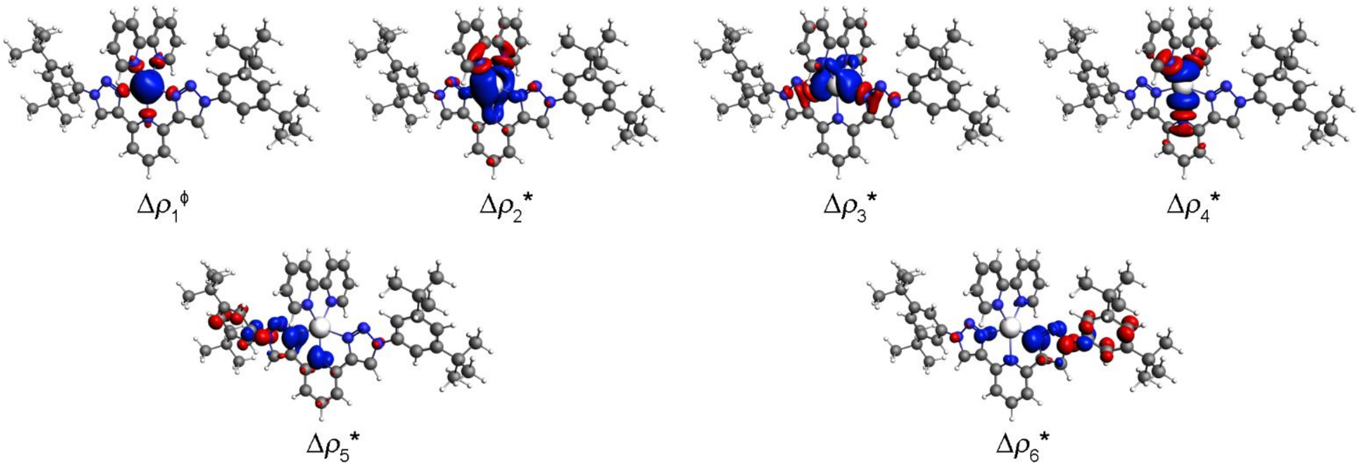

3•4$_{ac}$···Zn^{2+}

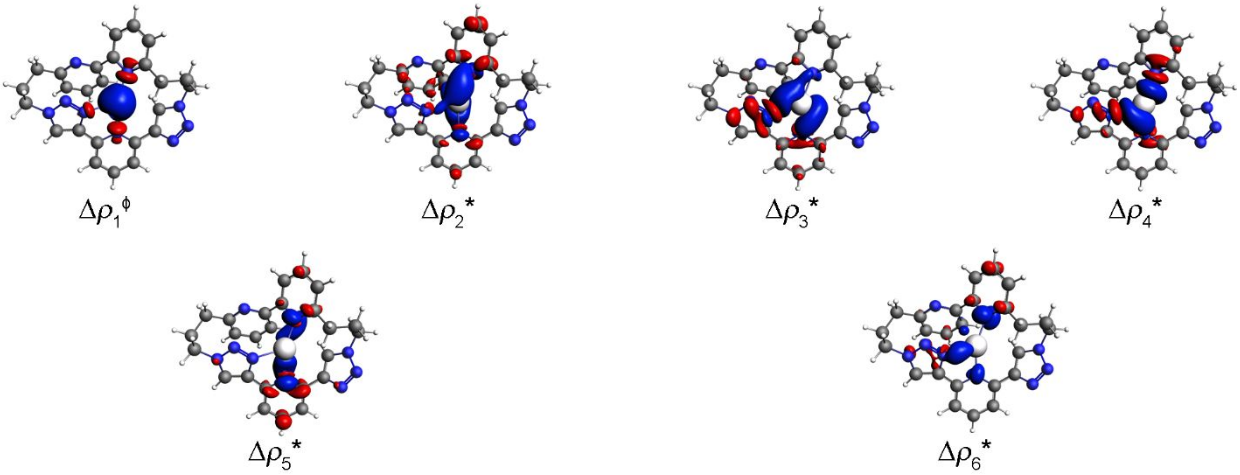

3•4$_{c}$···Zn^{2+}

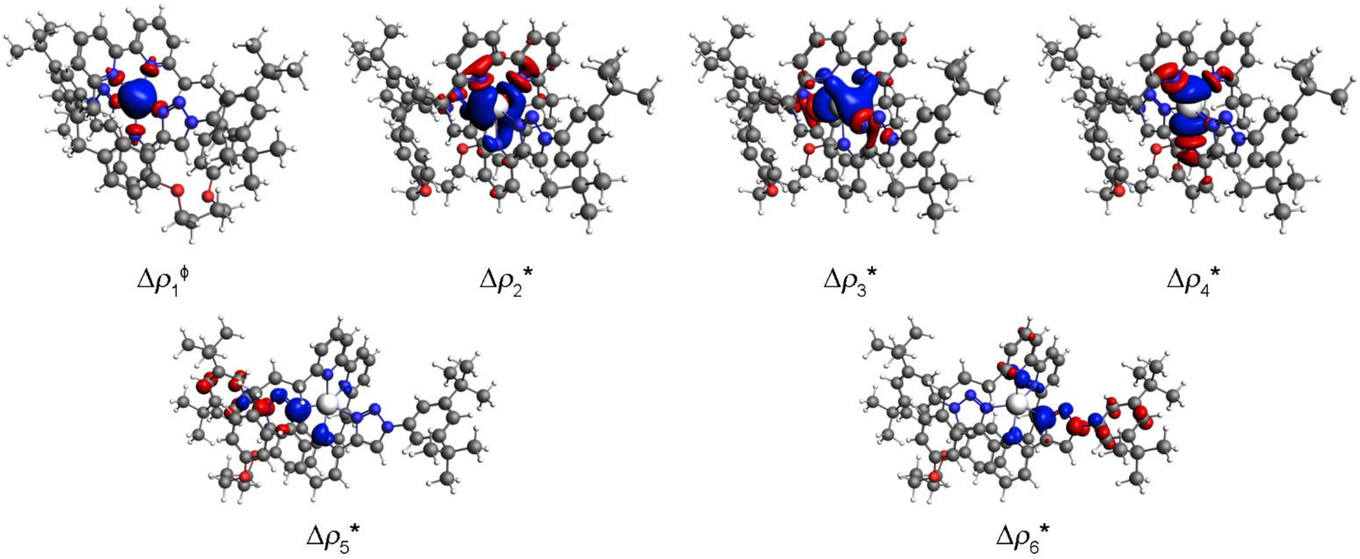

3•4···Zn^{2+}

Figure S2. The main density deformation channel surface plots with isovalues = $^{\phi}$ 0.005 and * 0.001 a.u., where the red and blue regions indicate the electron density outflow and inflow, respectively, for (**3•4**$_{ac}$, **3•4**$_{c}$ or **3•4**)···Zn^{2+} complexes. Color code for atoms: H = white; C = gray; N = blue; O = red; and Zn = ice.

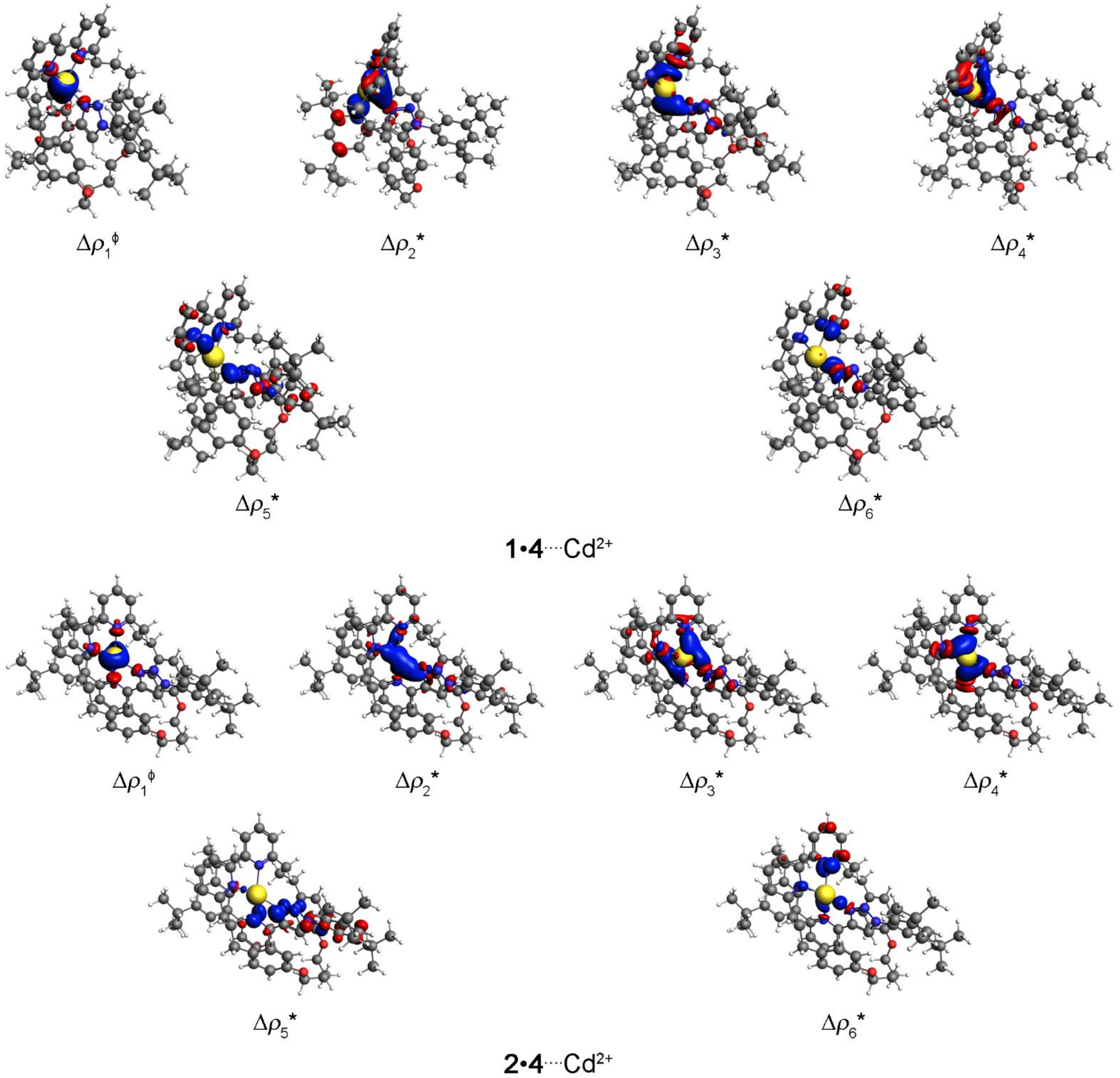

Figure S3. The main density deformation channel surface plots with isovalues = $^{\phi}$ 0.005 and * 0.001 a.u., where the red and blue regions indicate the electron density outflow and inflow, respectively, for (**1•4** or **2•4**)···Cd^{2+} complexes. Color code for atoms: H = white; C = gray; N = blue; O = red; and Cd = yellow.

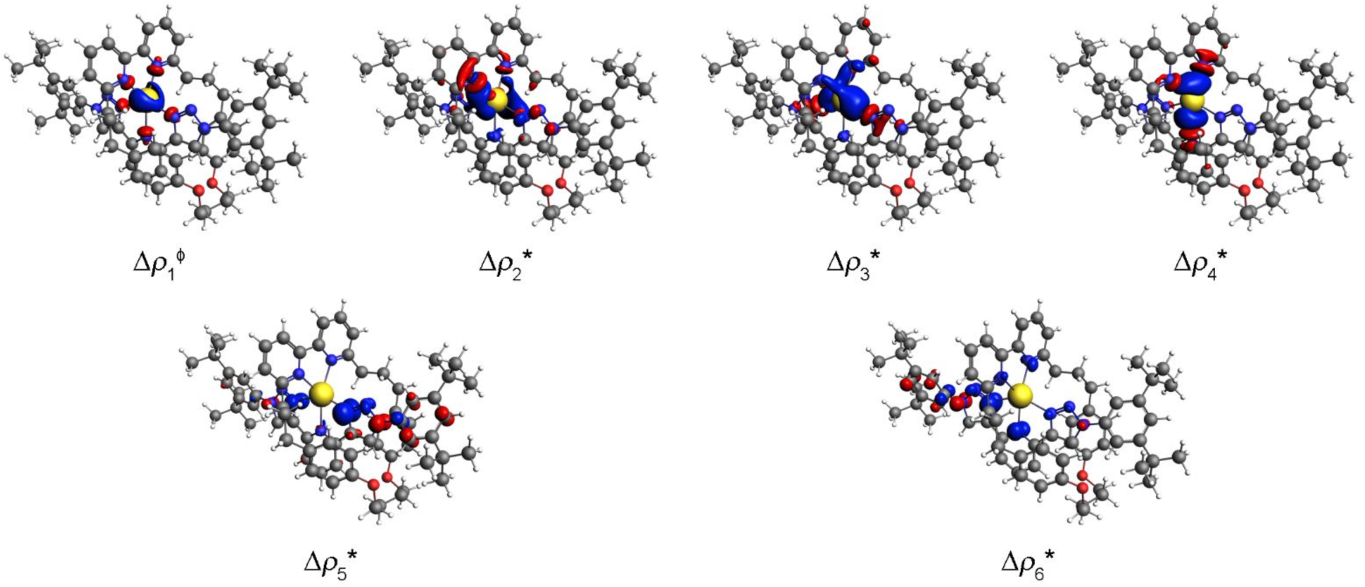

3•4···Cd^{2+}

Figure S4. The main density deformation channel surface plots with isovalues = $^{\phi}$ 0.005 and * 0.001 a.u., where the red and blue regions indicate the electron density outflow and inflow, respectively, for **3•4**···Cd^{2+} complexes. Color code for atoms: H = white; C = gray; N = blue; O = red; and Cd = yellow.

Table S1. Analysis of the bonding situation between the substituted receptors (**1•4**a–d, **2•4**a–d or **3•4**a–d) and transition metal cation (Zn^{2+}) through of the EDA–NOCV methodology and study of the charge distribution proportioned from the VDD method. The units of energy and charge are kcal mol^{-1} and a.u., respectively.[a,b]

Complex	ΔE_{int}	ΔV_{elstat}	ΔE_{Pauli}	ΔE_{oi}	ΔE_{disp}	$\Delta E_{oi,1}$	$\Delta E_{oi,2}$	$\Delta E_{oi,3}$	$\Delta E_{oi,4}$	$\Delta E_{oi,5}$	$\Delta E_{oi,6}$	q_{Cation}^{VDD}
**1•4**a···Zn^{2+}	–446.18	–286.06 (47)	157.46	–301.12 (50)	–16.46 (3)	–69.64	–29.62	–29.27	–31.29	–18.65	–15.13	–0.606
**1•4**b···Zn^{2+}	–464.38	–289.21 (47)	149.00	–307.49 (50)	–16.67 (3)	–71.89	–36.26	–27.61	–31.18	–18.23	–16.69	–0.623
**1•4**c···Zn^{2+}	–434.98	–258.12 (44)	150.25	–310.94 (53)	–16.17 (3)	–74.41	–36.56	–27.51	–28.84	–16.51	–15.53	–0.617
**1•4**d···Zn^{2+}	–386.84	–173.58 (34)	119.54	–315.94 (62)	–16.86 (3)	–83.21	–35.78	–33.75	–28.82	–17.33	–10.48	–0.636
**2•4**a···Zn^{2+}	–488.72	–319.72 (49)	158.40	–310.60 (48)	–16.80 (3)	–65.10	–30.29	–28.34	–30.02	–15.93	–16.42	–0.602
**2•4**b···Zn^{2+}	–494.30	–316.62 (49)	150.87	–311.87 (48)	–16.68 (3)	–66.47	–34.42	–24.67	–28.88	–19.28	–16.76	–0.608
**2•4**c···Zn^{2+}	–437.06	–262.51 (44)	161.45	–318.58 (53)	–17.43 (3)	–57.87	–34.56	–25.83	–27.56	–29.06	–17.42	–0.597
**2•4**d···Zn^{2+}	–421.10	–233.66 (42)	141.47	–312.00 (55)	–16.90 (3)	–66.98	–33.30	–26.11	–30.91	–17.39	–17.11	–0.606
**3•4**a···Zn^{2+}	–518.30	–330.22 (51)	134.45	–306.64 (47)	–15.90 (2)	–64.35	–26.33	–26.95	–28.11	–13.74	–15.61	–0.584
**3•4**b···Zn^{2+}	–512.03	–314.13 (49)	124.74	–306.95 (48)	–15.69 (2)	–67.11	–32.14	–24.23	–27.91	–15.50	–14.34	–0.600
**3•4**c···Zn^{2+}	–466.50	–272.39 (45)	134.09	–312.05 (52)	–16.15 (3)	–61.61	–23.35	–25.78	–26.63	–25.35	–13.26	–0.588
**3•4**d···Zn^{2+}	–433.28	–227.81 (41)	117.48	–306.56 (56)	–16.38 (3)	–68.26	–29.19	–29.85	–26.30	–14.64	–13.60	–0.604

[a] $\Delta E_{int} = \Delta V_{elstat} + \Delta E_{Pauli} + \Delta E_{oi} + \Delta E_{disp}$; [b] Values in parentheses correspond to the percentage of each stabilizing contribution ($\Delta V_{elstat} + \Delta E_{oi} + \Delta E_{disp}$ = 100%).

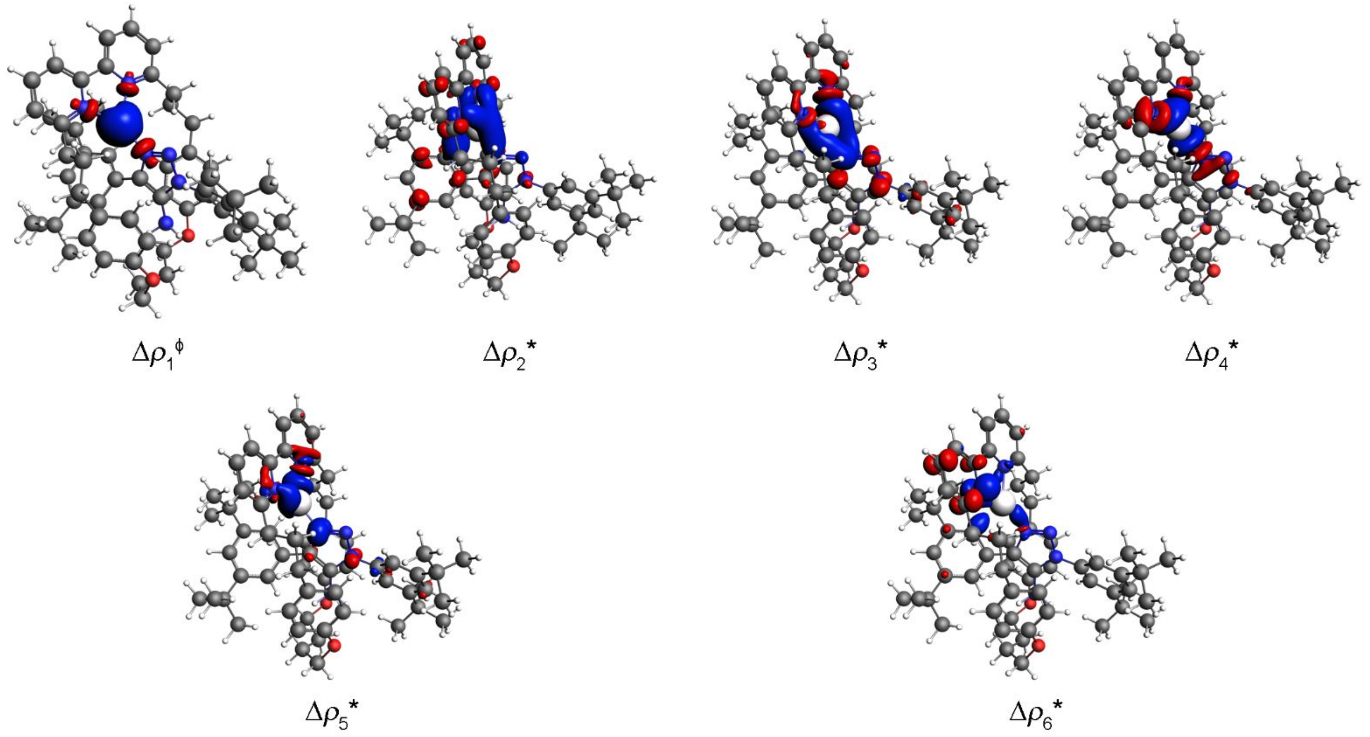

1•4a···Zn^{2+}

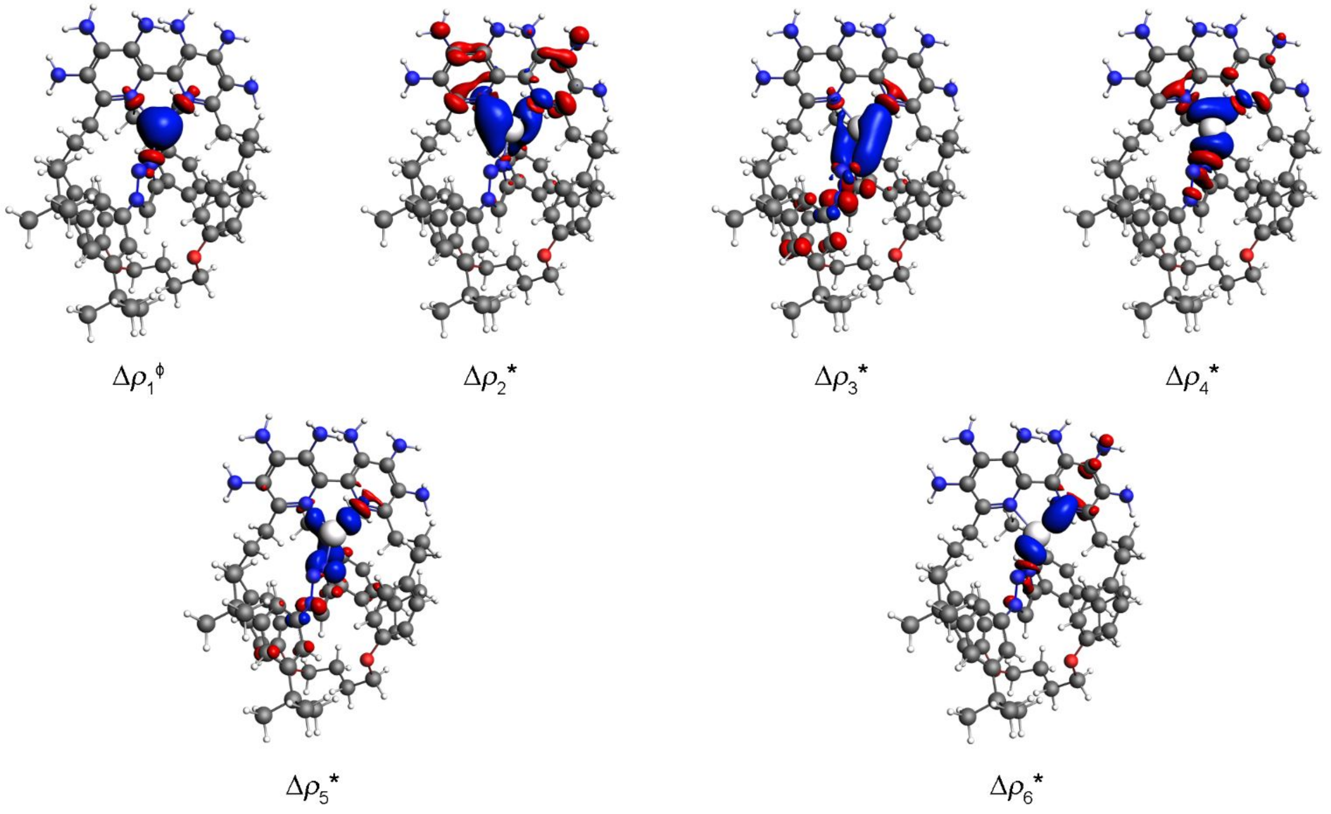

1•4b···Zn^{2+}

Figure S5. The main density deformation channel surface plots with isovalues = $^{\phi}$ 0.005 and * 0.001 a.u., where the red and blue regions indicate the electron density outflow and inflow, respectively, for **1•4**(a or b)···Zn^{2+} complexes. Color code for atoms: H = white; C = gray; N = blue; O = red; and Zn = ice.

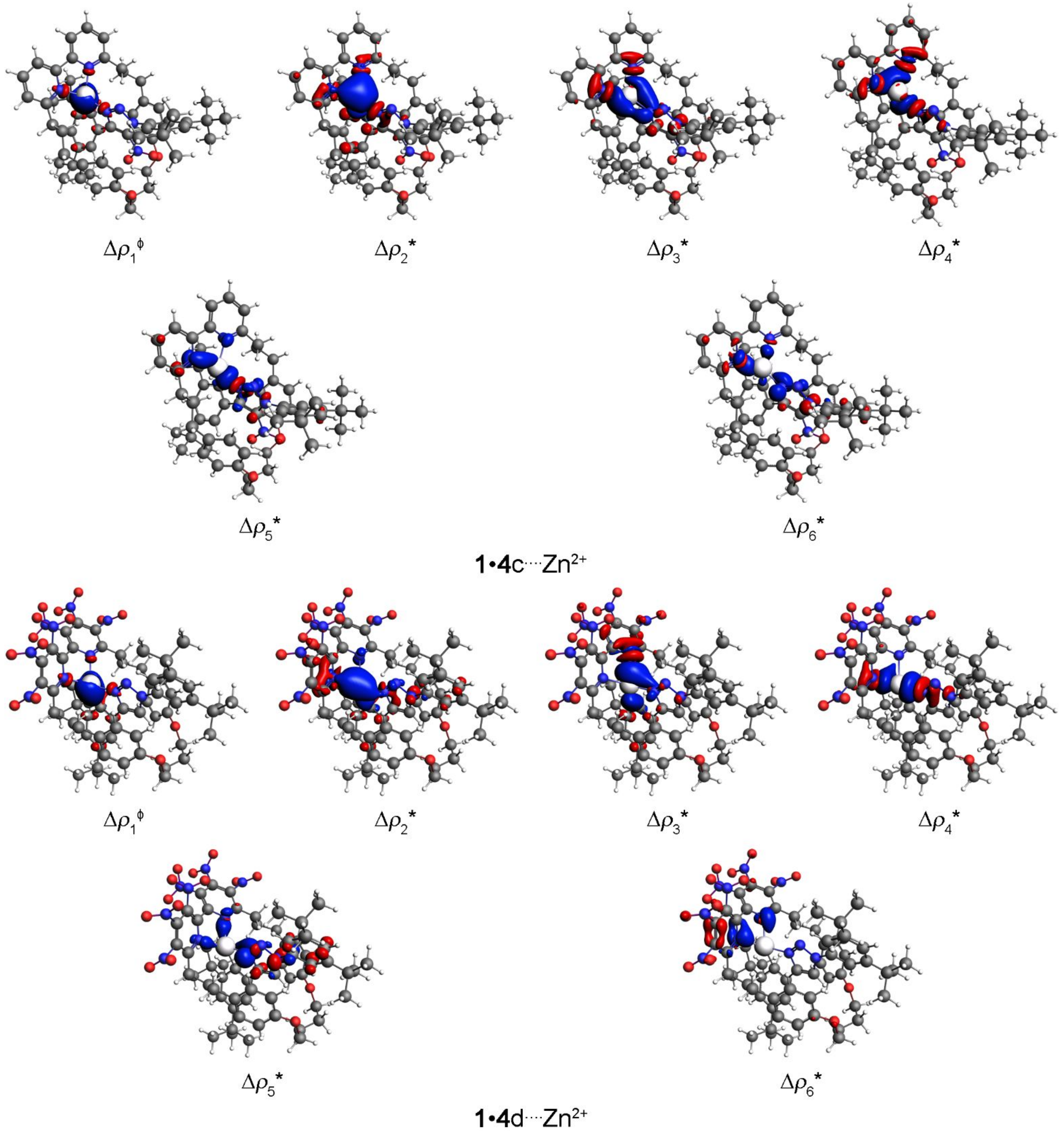

Figure S6. The main density deformation channel surface plots with isovalues = ϕ 0.005 and * 0.001 a.u., where the red and blue regions indicate the electron density outflow and inflow, respectively, for **1•4**(c or d)···Zn^{2+} complexes. Color code for atoms: H = white; C = gray; N = blue; O = red; and Zn = ice.

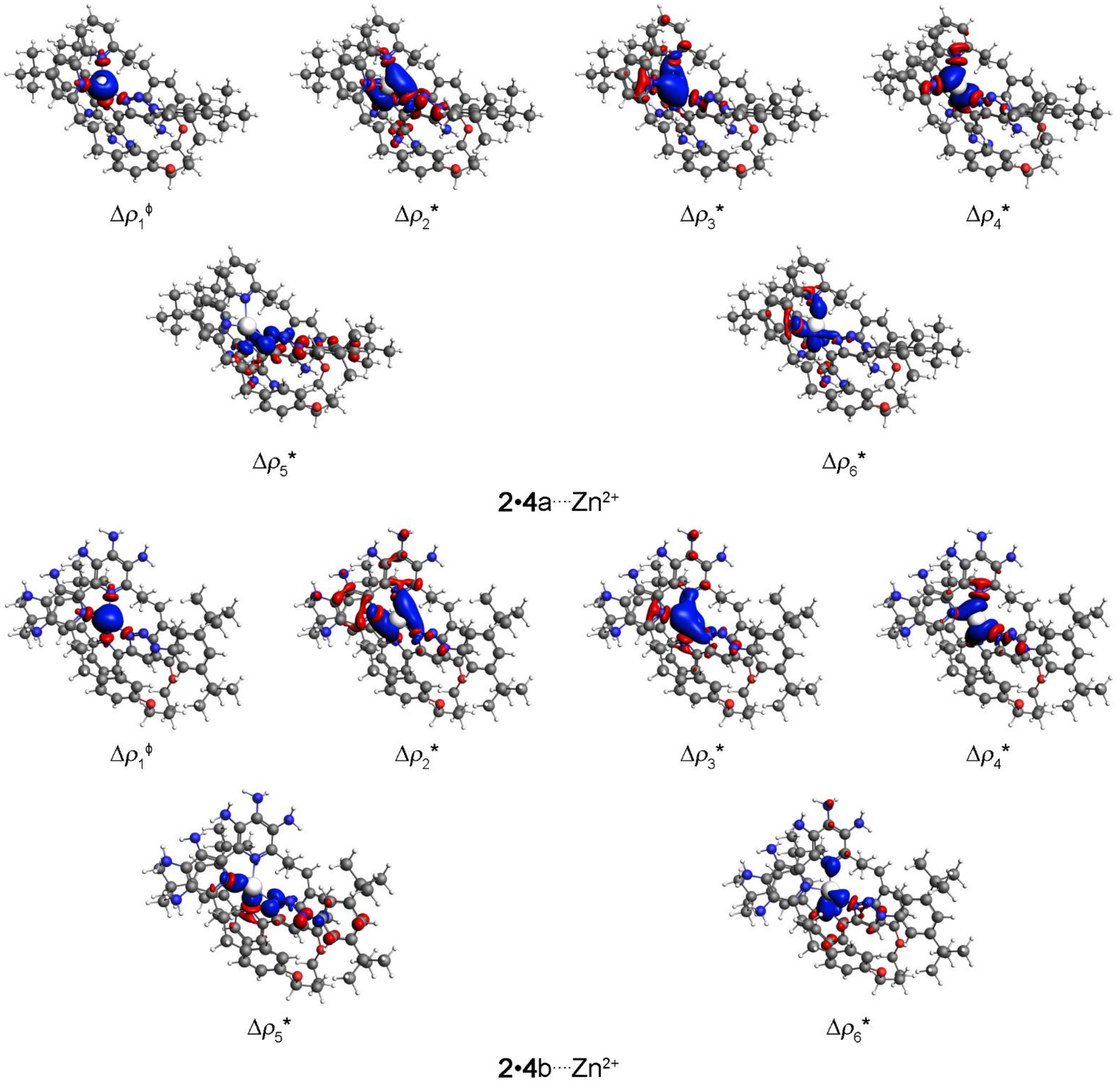

Figure S7. The main density deformation channel surface plots with isovalues = $^{\phi}$ 0.005 and * 0.001 a.u., where the red and blue regions indicate the electron density outflow and inflow, respectively, for **2•4**(a or b)···Zn^{2+} complexes. Color code for atoms: H = white; C = gray; N = blue; O = red; and Zn = ice.

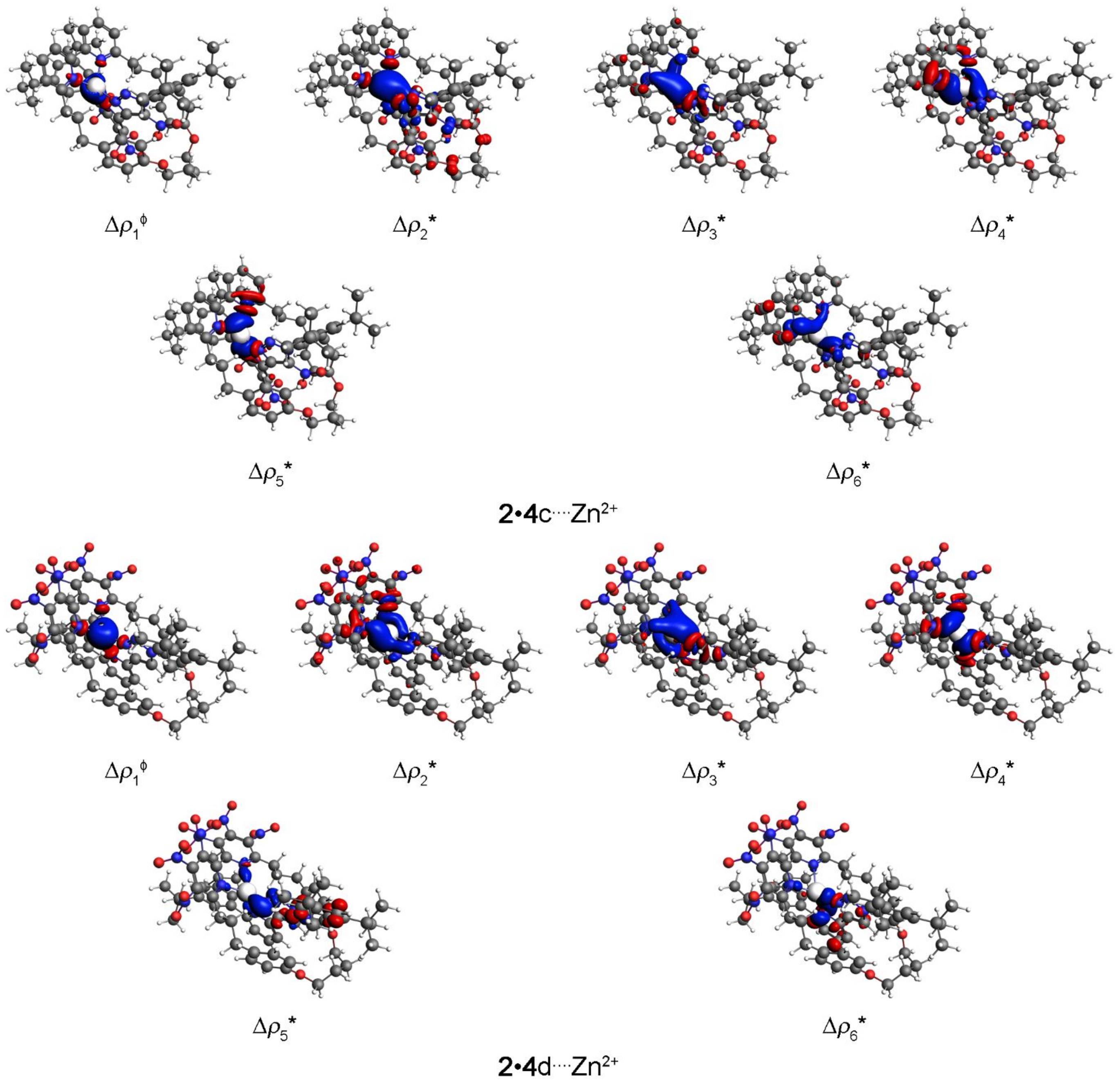

Figure S8. The main density deformation channel surface plots with isovalues = $^{\phi}$ 0.005 and * 0.001 a.u., where the red and blue regions indicate the electron density outflow and inflow, respectively, for **2•4**(c or d)···Zn^{2+} complexes. Color code for atoms: H = white; C = gray; N = blue; O = red; and Zn = ice.

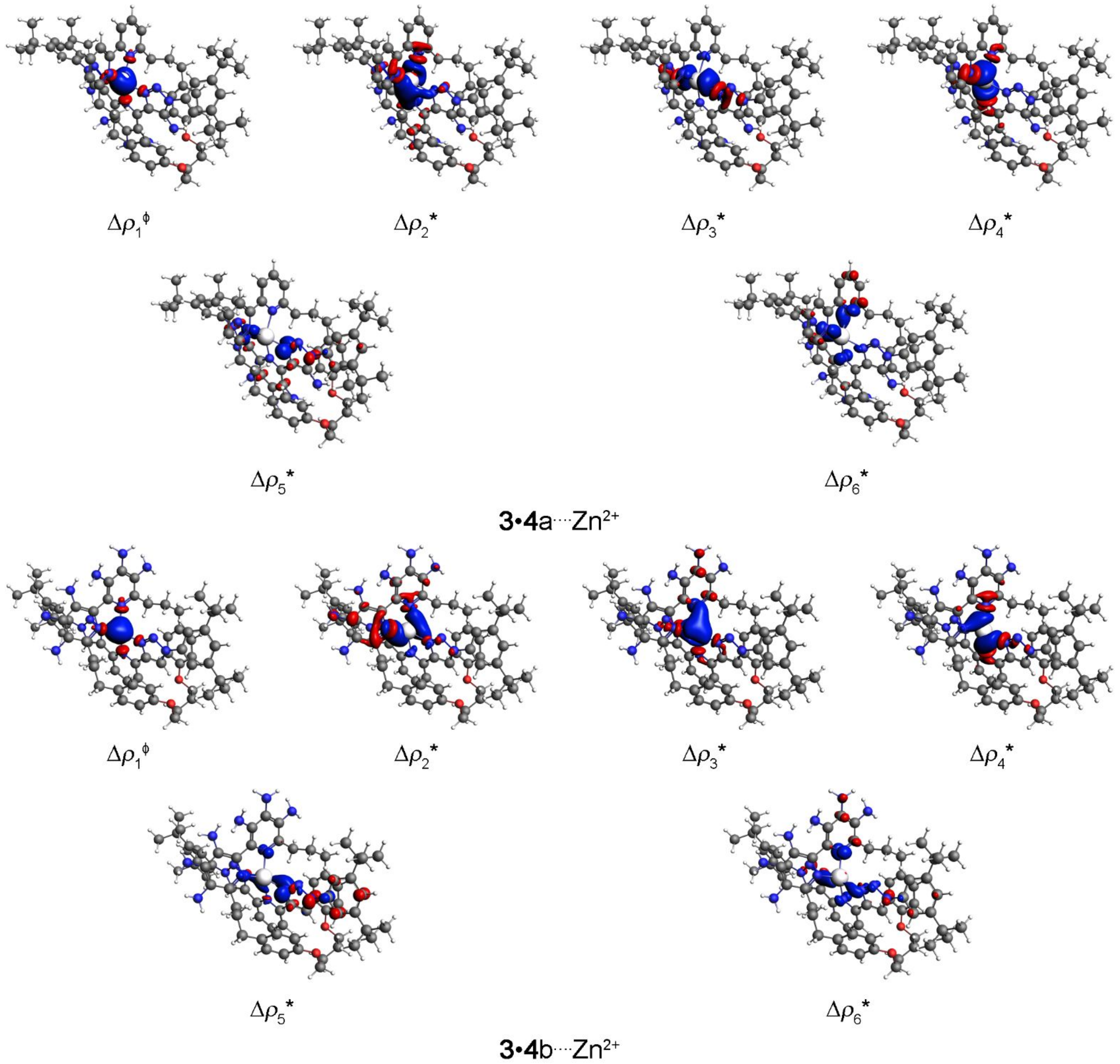

Figure S9. The main density deformation channel surface plots with isovalues = $^{\phi}$ 0.005 and * 0.001 a.u., where the red and blue regions indicate the electron density outflow and inflow, respectively, for **3•4**(a or b)···Zn^{2+} complexes. Color code for atoms: H = white; C = gray; N = blue; O = red; and Zn = ice.

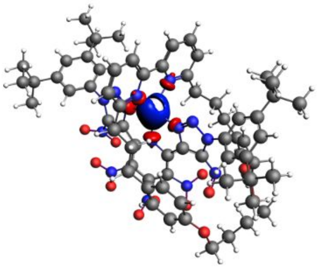

$\Delta\rho_1^{\phi}$

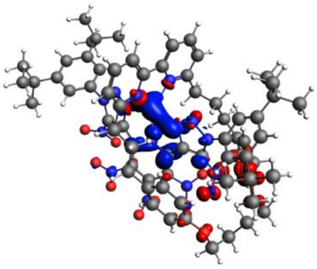

$\Delta\rho_2^{*}$

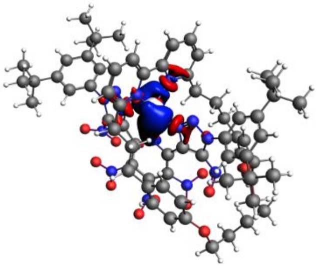

$\Delta\rho_3^{*}$

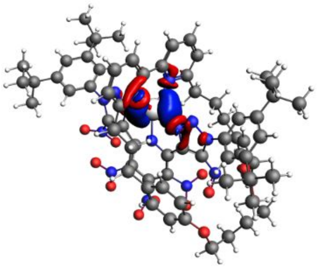

$\Delta\rho_4^{*}$

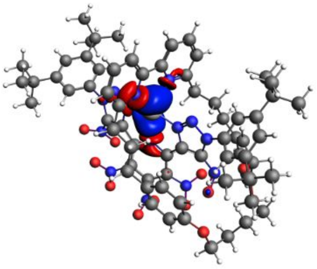

$\Delta\rho_5^{*}$

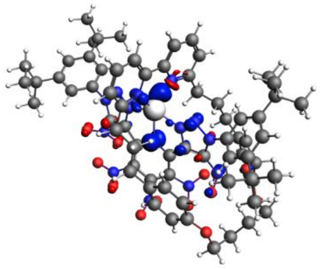

$\Delta\rho_6^{*}$

3•4c···Zn^{2+}

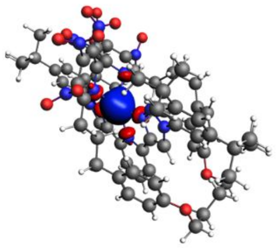

$\Delta\rho_1^{\phi}$

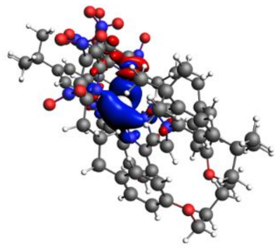

$\Delta\rho_2^{*}$

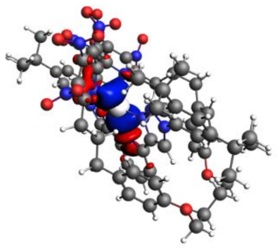

$\Delta\rho_3^{*}$

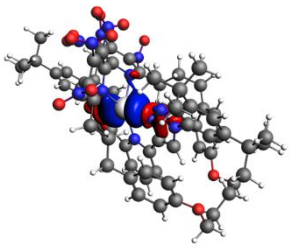

$\Delta\rho_4^{*}$

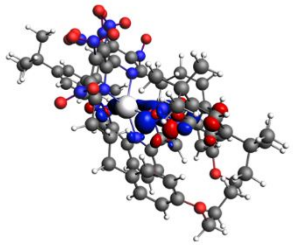

$\Delta\rho_5^{*}$

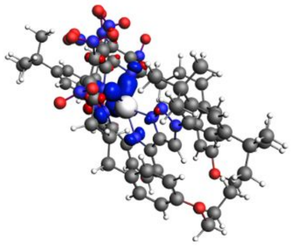

$\Delta\rho_6^{*}$

3•4d···Zn^{2+}

Figure S10. The main density deformation channel surface plots with isovalues = $^{\phi}$ 0.005 and * 0.001 a.u., where the red and blue regions indicate the electron density outflow and inflow, respectively, for **3•4**(c or d)···Zn^{2+} complexes. Color code for atoms: H = white; C = gray; N = blue; O = red; and Zn = ice.

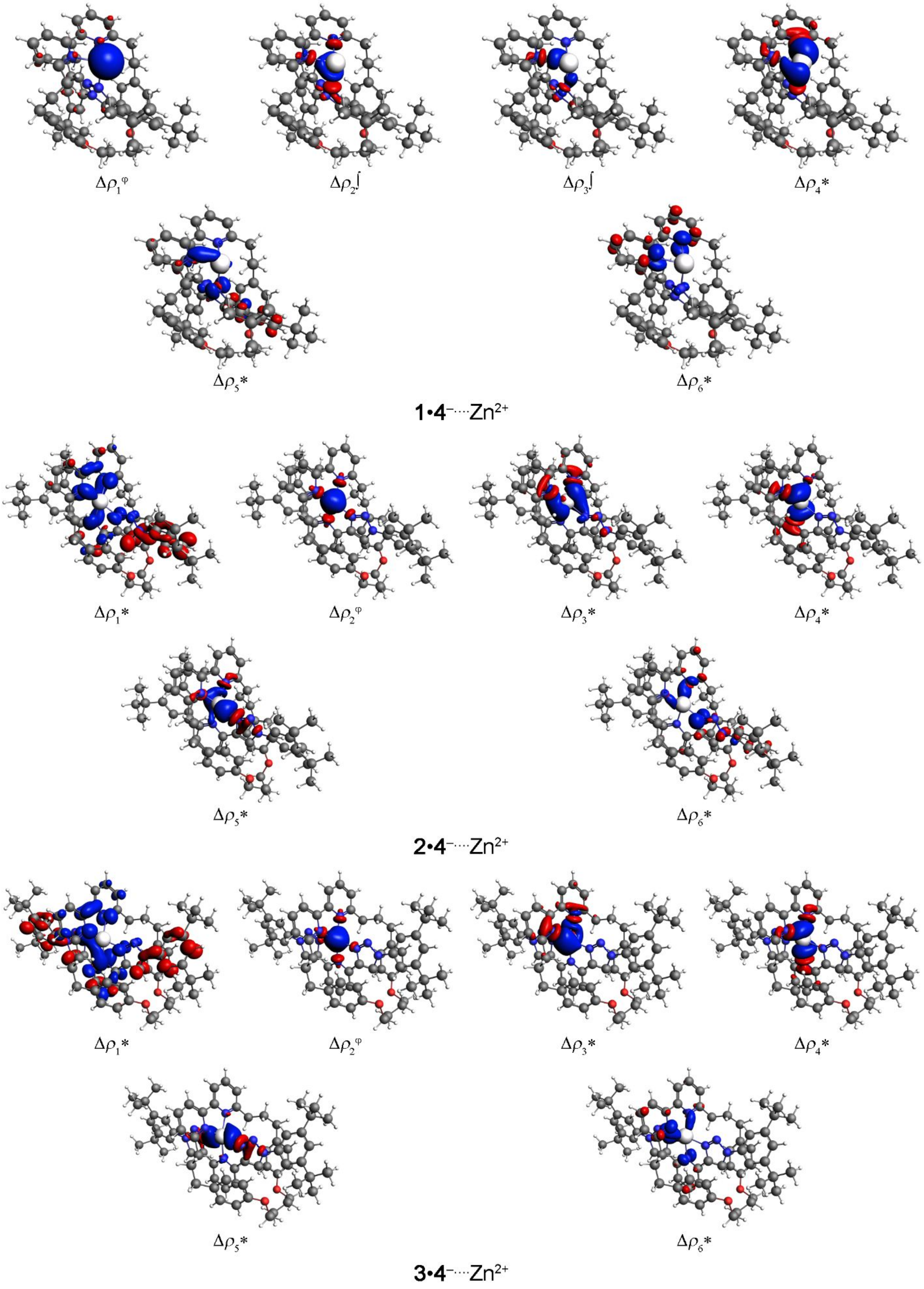

Figure S11. The main density deformation channel surface plots with isovalues = ϕ 0.005; ∫ 0.003 and * 0.001 a.u., where the red and blue regions indicate the electron density outflow and inflow, respectively, for (**1•4**$^{-}$, **2•4**$^{-}$ or **3•4**$^{-}$)····Zn^{2+} complexes. Color code for atoms: H = white; C = gray; N = blue; O = red; and Zn = ice.

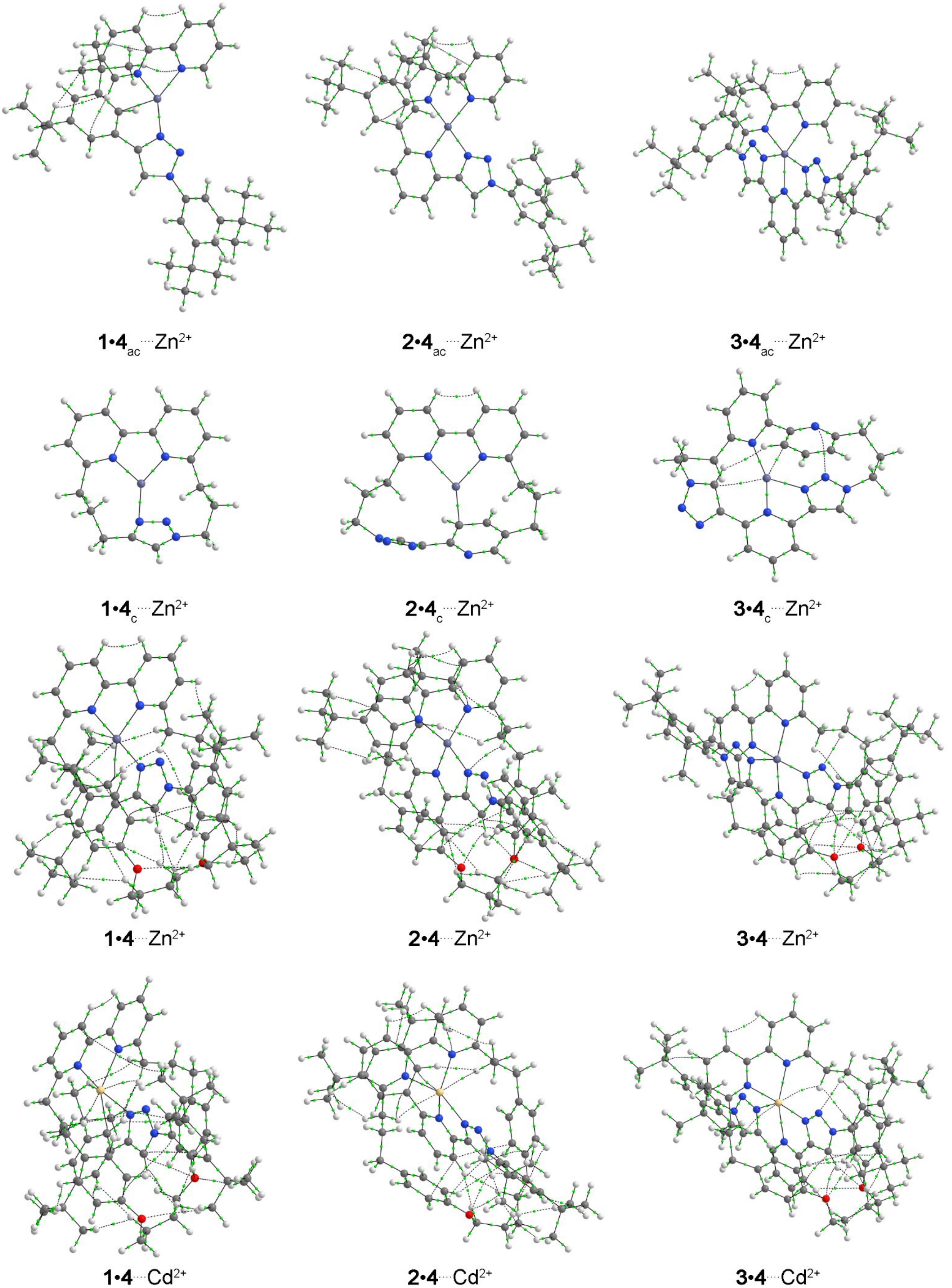

Figure S12. Topological map containing the bond paths (continuous or dashed lines connecting the cores) and bond critical points (small green points), for the complexes (**1•4**$_{ac}$, **2•4**$_{ac}$, **3•4**$_{ac}$, **1•4**$_{c}$, **2•4**$_{c}$, **3•4**$_{c}$, **1•4**, **2•4** or **3•4**)···(Zn^{2+} or Cd^{2+}). Atoms color code: H = white; C = gray; N = blue; O = red; Zn = purple; and Cd = yellow.

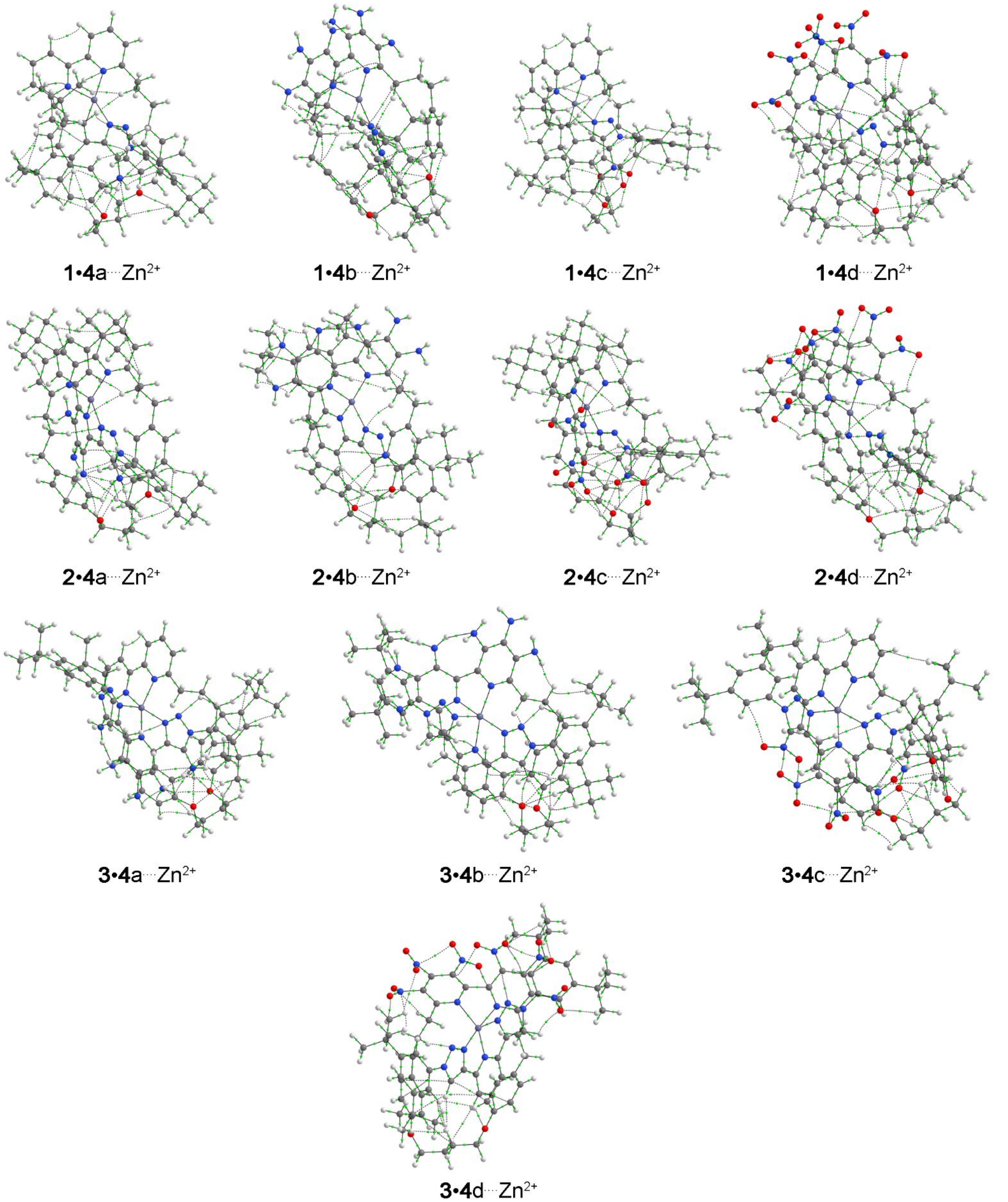

Figure S13. Topological map containing the bond paths (continuous or dashed lines connecting the cores) and bond critical points (small green points), for the complexes (**1•4**, **2•4** or **3•4**)a–d···Zn^{2+}. Atoms color code: H = white; C = gray; N = blue; O = red; and Zn = purple.

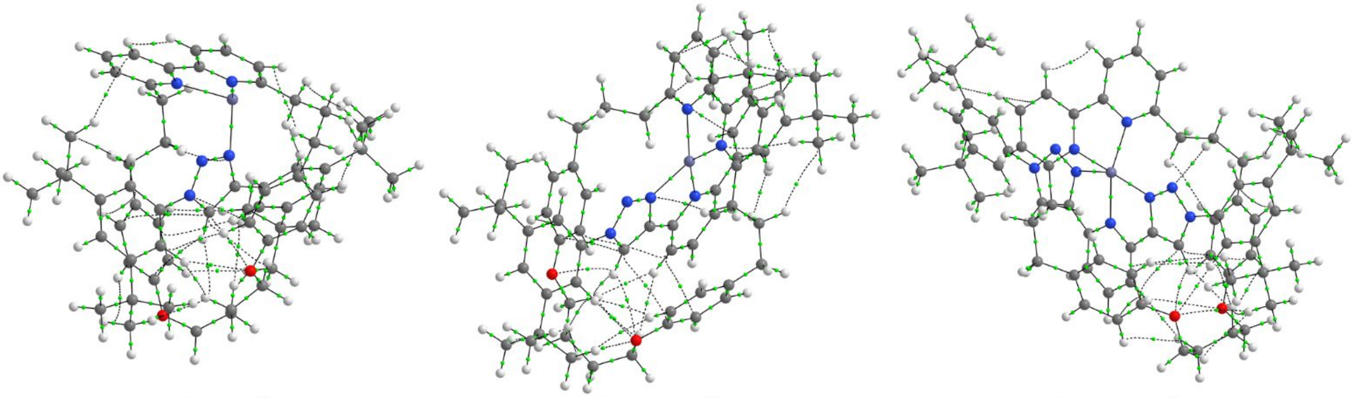

1•4$^{-}\cdots$Zn^{2+} **2•4**$^{-}\cdots$Zn^{2+} **3•4**$^{-}\cdots$Zn^{2+}

Figure S14. Topological map containing the bond paths (continuous or dashed lines connecting the cores) and bond critical points (small green points), for the complexes (**1•4**$^{-}$, **2•4**$^{-}$ or **3•4**$^{-}$)$\cdots$Zn^{2+}. Atoms color code: H = white; C = gray; N = blue; O = red; and Zn = purple.

Table S2. Ratio between the kinetic energy density, G_b, and potential energy density, V_b, $–G_b/V_b$, and electron density, ρ_b, at BCPs related to interactions between the receptors (**1•4**$_{ac}$, **2•4**$_{ac}$, **3•4**$_{ac}$, **1•4**$_{c}$, **2•4**$_{c}$, **3•4**$_{c}$, **1•4**, **2•4** or **3•4**) and cation (Zn^{2+}). The values of all the parameters are in a.u.

Complex	BCP	$–G_b/V_b$	ρ_b
**1•4**$_{ac}$···Zn^{2+}	N···Zn^{2+}	0.832	0.099
	C···Zn^{2+}	0.829	0.028
	N···Zn^{2+}	0.847	0.086
	N···Zn^{2+}	0.845	0.086
**2•4**$_{ac}$···Zn^{2+}	N···Zn^{2+}	0.855	0.081
	N···Zn^{2+}	0.855	0.082
	N···Zn^{2+}	0.868	0.078
	N···Zn^{2+}	0.852	0.081
**3•4**$_{ac}$···Zn^{2+}	N···Zn^{2+}	0.865	0.076
	N···Zn^{2+}	0.866	0.076
	N···Zn^{2+}	0.897	0.059
	N···Zn^{2+}	0.867	0.067
	N···Zn^{2+}	0.897	0.059
**1•4**$_{c}$···Zn^{2+}	N···Zn^{2+}	0.837	0.091
	N···Zn^{2+}	0.822	0.102
	N···Zn^{2+}	0.836	0.092
**2•4**$_{c}$···Zn^{2+}	N···Zn^{2+}	0.847	0.081
	N···Zn^{2+}	0.842	0.084
	C···Zn^{2+}	0.783	0.061
**3•4**$_{c}$···Zn^{2+}	N···Zn^{2+}	0.829	0.092
	N···Zn^{2+}	0.866	0.078
	N···Zn^{2+}	0.840	0.085
	C···Zn^{2+}	0.855	0.021
	C···Zn^{2+}	0.921	0.014
**1•4**···Zn^{2+}	N···Zn^{2+}	0.838	0.095
	C···Zn^{2+}	0.861	0.031
	C–H···Zn^{2+}	0.975	0.010
	N···Zn^{2+}	0.848	0.084
	N···Zn^{2+}	0.851	0.082
	C–H···Zn^{2+}	0.906	0.016
**2•4**···Zn^{2+}	N···Zn^{2+}	0.863	0.081
	N···Zn^{2+}	0.854	0.079
	N···Zn^{2+}	0.851	0.083
	N···Zn^{2+}	0.853	0.082
**3•4**···Zn^{2+}	N···Zn^{2+}	0.888	0.065
	N···Zn^{2+}	0.867	0.068
	N···Zn^{2+}	0.905	0.052
	N···Zn^{2+}	0.863	0.076
	N···Zn^{2+}	0.863	0.076

Table S3. Ratio between the kinetic energy density, G_b, and potential energy density, V_b, $-G_b/V_b$, and electron density, ρ_b, at BCPs related to interactions between: i) the receptors (**1•4**, **2•4** or **3•4**) and cation (Cd^{2+}); and ii) the receptors (**1•4**⁻, **2•4**⁻ or **3•4**⁻) and cation (Zn^{2+}). The values of all the parameters are in a.u.

Complex	BCP	$-G_b/V_b$	ρ_b
**1•4**···Cd^{2+}	N···Cd^{2+}	0.877	0.069
	C···Cd^{2+}	0.845	0.038
	C–H···Cd^{2+}	1.045	0.016
	N···Cd^{2+}	0.867	0.065
	N···Cd^{2+}	0.869	0.066
	C–H···Cd^{2+}	1.112	0.011
	C–H···Cd^{2+}	1.111	0.013
**2•4**···Cd^{2+}	N···Cd^{2+}	0.880	0.067
	N···Cd^{2+}	0.865	0.069
	C···Cd^{2+}	1.098	0.011
	N···Cd^{2+}	0.867	0.071
	N···Cd^{2+}	0.868	0.070
	C–H···Cd^{2+}	1.143	0.012
	C–H···Cd^{2+}	1.216	0.006
**3•4**···Cd^{2+}	N···Cd^{2+}	0.888	0.059
	N···Cd^{2+}	0.874	0.057
	N···Cd^{2+}	0.899	0.048
	N···Cd^{2+}	0.871	0.066
	N···Cd^{2+}	0.875	0.064
	C–H···Cd^{2+}	1.158	0.011
	C–H···Cd^{2+}	1.136	0.012
**1•4**⁻···Zn^{2+}	N···Zn^{2+}	0.871	0.069
	N···Zn^{2+}	0.864	0.075
	N···Zn^{2+}	0.879	0.070
**2•4**⁻···Zn^{2+}	N···Zn^{2+}	0.873	0.077
	N···Zn^{2+}	0.845	0.088
	N···Zn^{2+}	0.846	0.087
	N···Zn^{2+}	0.859	0.078
**3•4**⁻···Zn^{2+}	N···Zn^{2+}	0.858	0.080
	N···Zn^{2+}	0.858	0.080
	N···Zn^{2+}	0.897	0.061
	N···Zn^{2+}	0.861	0.075
	N···Zn^{2+}	0.913	0.045

Table S4. Ratio between the kinetic energy density, G_b, and potential energy density, V_b, $-G_b/V_b$, and electron density, ρ_b, at BCPs related to interactions between the receptors (**1•4**, **2•4** or **3•4**)(a–d) and cation (Zn^{2+}). The values of all the parameters are in a.u.

Complex	BCP	$-G_b/V_b$	ρ_b
**1•4**a···Zn^{2+}	N···Zn^{2+}	0.818	0.108
	C···Zn^{2+}	0.857	0.027
	N···Zn^{2+}	0.837	0.092
	N···Zn^{2+}	0.839	0.091
**1•4**b···Zn^{2+}	N···Zn^{2+}	0.828	0.101
	C···Zn^{2+}	0.852	0.025
	N···Zn^{2+}	0.845	0.087
	N···Zn^{2+}	0.832	0.094
**1•4**c···Zn^{2+}	N···Zn^{2+}	0.845	0.094
	C···Zn^{2+}	0.830	0.049
	N···Zn^{2+}	0.836	0.094
	N···Zn^{2+}	0.847	0.086
**1•4**d···Zn^{2+}	N···Zn^{2+}	0.846	0.090
	C···Zn^{2+}	0.811	0.053
	C···Zn^{2+}	1.062	0.010
	N···Zn^{2+}	0.867	0.072
	N···Zn^{2+}	0.868	0.071
	C–H···Zn^{2+}	0.929	0.015
**2•4**a···Zn^{2+}	N···Zn^{2+}	0.857	0.085
	N···Zn^{2+}	0.843	0.087
	N···Zn^{2+}	0.849	0.087
	N···Zn^{2+}	0.853	0.084
**2•4**b···Zn^{2+}	N···Zn^{2+}	0.865	0.081
	N···Zn^{2+}	0.860	0.077
	N···Zn^{2+}	0.847	0.086
	N···Zn^{2+}	0.841	0.089
**2•4**c···Zn^{2+}	N···Zn^{2+}	0.860	0.085
	N···Zn^{2+}	0.850	0.084
	N···Zn^{2+}	0.849	0.086
	N···Zn^{2+}	0.837	0.093
**2•4**d···Zn^{2+}	N···Zn^{2+}	0.864	0.080
	N···Zn^{2+}	0.844	0.085
	N···Zn^{2+}	0.856	0.081
	N···Zn^{2+}	0.859	0.080
**3•4**a···Zn^{2+}	N···Zn^{2+}	0.881	0.070
	N···Zn^{2+}	0.864	0.069
	N···Zn^{2+}	0.888	0.064
	N···Zn^{2+}	0.867	0.075
	N···Zn^{2+}	0.867	0.074
**3•4**b···Zn^{2+}	N···Zn^{2+}	0.889	0.065
	N···Zn^{2+}	0.870	0.067
	N···Zn^{2+}	0.911	0.045
	N···Zn^{2+}	0.854	0.081
	N···Zn^{2+}	0.863	0.075
**3•4**c···Zn^{2+}	N···Zn^{2+}	0.879	0.071
	N···Zn^{2+}	0.876	0.060
	N···Zn^{2+}	0.891	0.064
	N···Zn^{2+}	0.862	0.076
	N···Zn^{2+}	0.857	0.080
**3•4**d···Zn^{2+}	N···Zn^{2+}	0.873	0.072
	N···Zn^{2+}	0.861	0.070
	N···Zn^{2+}	0.887	0.062
	N···Zn^{2+}	0.885	0.060
	N···Zn^{2+}	0.880	0.064

Table S5. Analysis of the bonding situation between the substituted receptors (**5•6**$_N$(a–f) or **5•6**$_O$(a–f)) and alkali metal cation (Na^+) through of the EDA–NOCV methodology and study of the charge distribution proportioned from the VDD method. The units of energy and charge are kcal mol^{-1} and a.u., respectively.

Complex	ΔE_{int}	ΔV_{elstat}	ΔE_{Pauli}	ΔE_{oi}	ΔE_{disp}	$\Delta E_{oi,1}$	$\Delta E_{oi,2}$	$\Delta E_{oi,3}$	q_{Cation}^{VDD}
**5•6**$_N$(a)···Na^+	–88.88	–50.47 (47)	19.00	–42.49 (39)	–14.92 (14)	–5.68	–5.22	–3.42	–0.118
**5•6**$_N$(b)···Na^+	–98.35	–60.71 (51)	21.25	–43.10 (36)	–15.79 (13)	–5.99	–5.31	–3.34	–0.108
**5•6**$_N$(c)···Na^+	–90.36	–56.60 (49)	24.87	–43.23 (38)	–15.41 (13)	–5.88	–3.94	–4.24	–0.101
**5•6**$_N$(d)···Na^+	–82.15	–42.32 (44)	14.90	–40.14 (41)	–14.59 (15)	–5.45	–4.32	–2.87	–0.099
**5•6**$_N$(e)···Na^+	–88.18	–51.65 (48)	19.02	–40.91 (38)	–14.64 (14)	–5.16	–4.51	–3.17	–0.109
**5•6**$_N$(f)···Na^+	–93.37	–62.15 (53)	23.22	–40.81 (35)	–13.63 (12)	–4.59	–3.86	–3.31	–0.091
**5•6**$_O$(a)···Na^+	–113.30	–80.46 (59)	23.79	–41.04 (30)	–15.60 (11)	–3.93	–4.18	–3.05	–0.094
**5•6**$_O$(b)···Na^+	–112.83	–80.41 (59)	22.50	–40.35 (30)	–14.57 (11)	–4.55	–3.40	–3.04	–0.099
**5•6**$_O$(c)···Na^+	–101.89	–71.42 (58)	21.82	–38.26 (31)	–14.03 (11)	–5.28	–3.43	–2.27	–0.088
**5•6**$_O$(d)···Na^+	–101.38	–72.09 (57)	24.14	–40.59 (32)	–12.84 (10)	–5.12	–4.44	–3.29	–0.085
**5•6**$_O$(e)···Na^+	–128.48	–98.61 (64)	26.73	–41.72 (27)	–14.88 (10)	–4.36	–3.79	–3.08	–0.091
**5•6**$_O$(f)···Na^+	–89.73	–55.68 (50)	22.17	–42.89 (38)	–13.33 (12)	–3.77	–3.60	–2.83	–0.077

[a] $\Delta E_{int} = \Delta V_{elstat} + \Delta E_{Pauli} + \Delta E_{oi} + \Delta E_{disp}$; [b] Values in parentheses correspond to the percentage of each stabilizing contribution ($\Delta V_{elstat} + \Delta E_{oi} + \Delta E_{disp}$ = 100%).

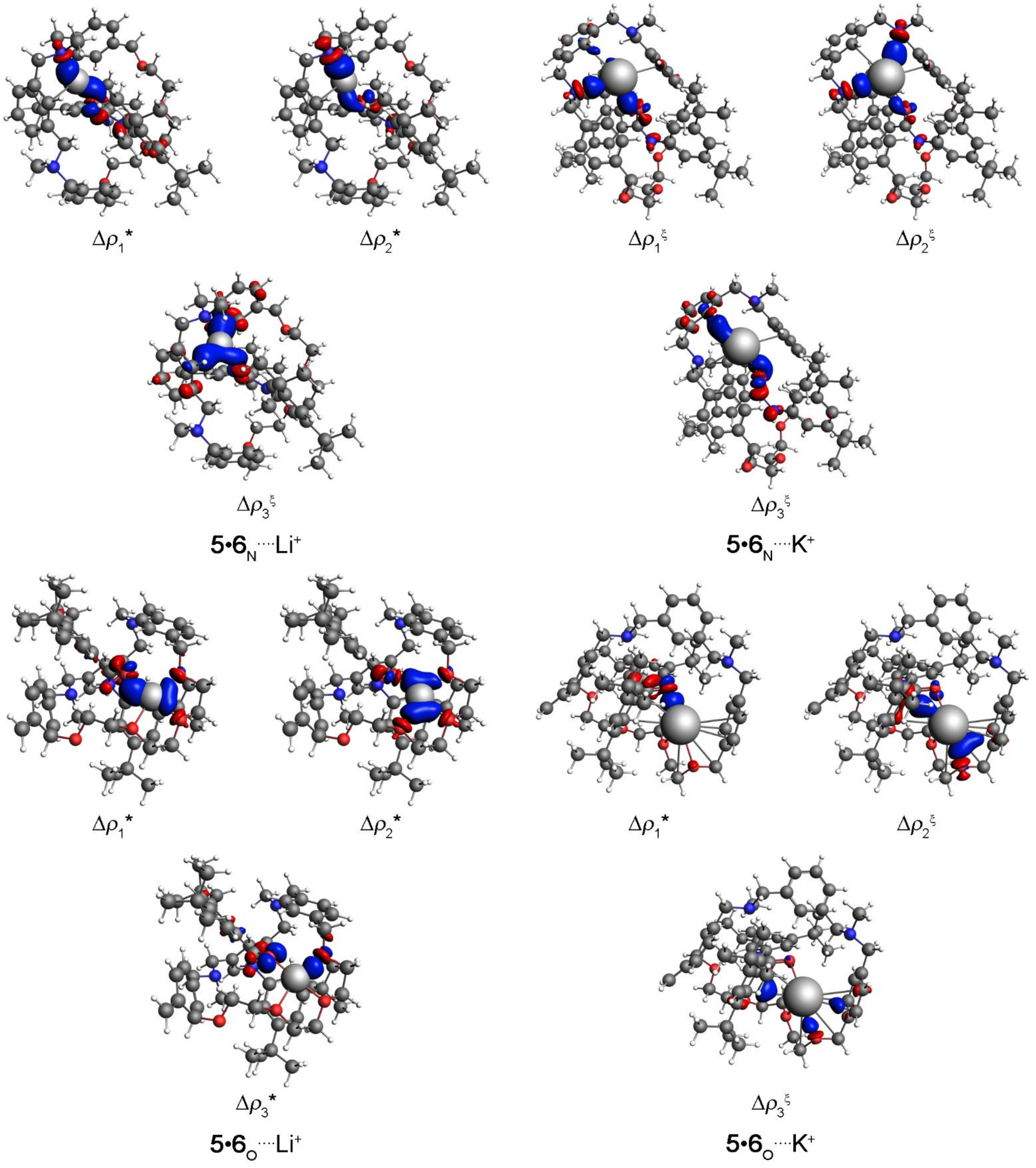

Figure S15. The main density deformation channel surface plots with isovalues = * 0.001 and ξ 0.0005 a.u., where the red and blue regions indicate the electron density outflow and inflow, respectively, for (**5•6**$_N$ or **5•6**$_O$)····(Li$^+$ or K$^+$) complexes. Color code for atoms: H = white; C = gray; N = blue; O = red; Li = light gray; and K = silver.

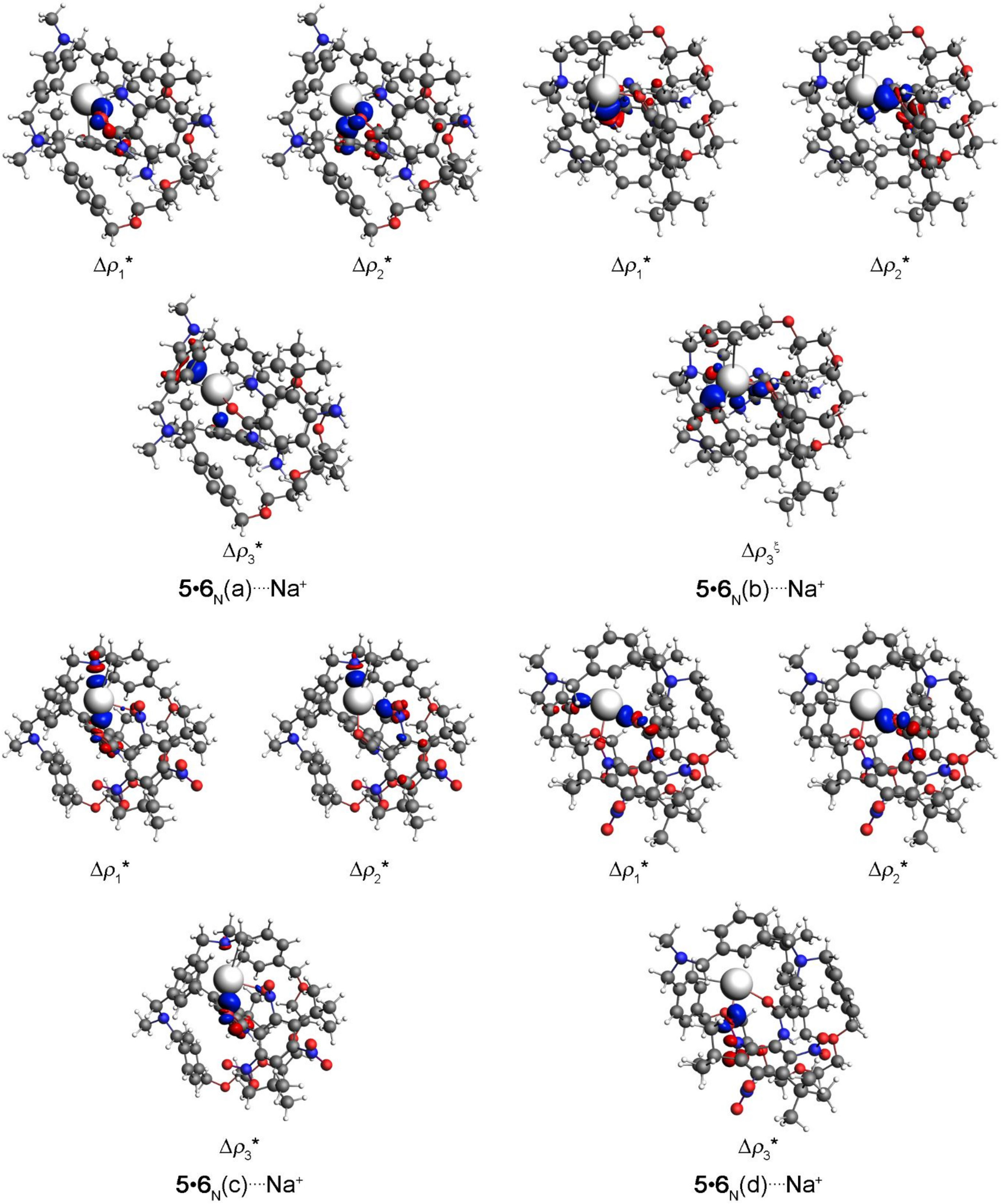

Figure S16. The main density deformation channel surface plots with isovalues = * 0.001 and $^{\xi}$ 0.0005 a.u., where the red and blue regions indicate the electron density outflow and inflow, respectively, for **5•6**$_N$(a–d)···Na$^+$ complexes. Color code for atoms: H = white; C = gray; N = blue; O = red; and Na = ice.

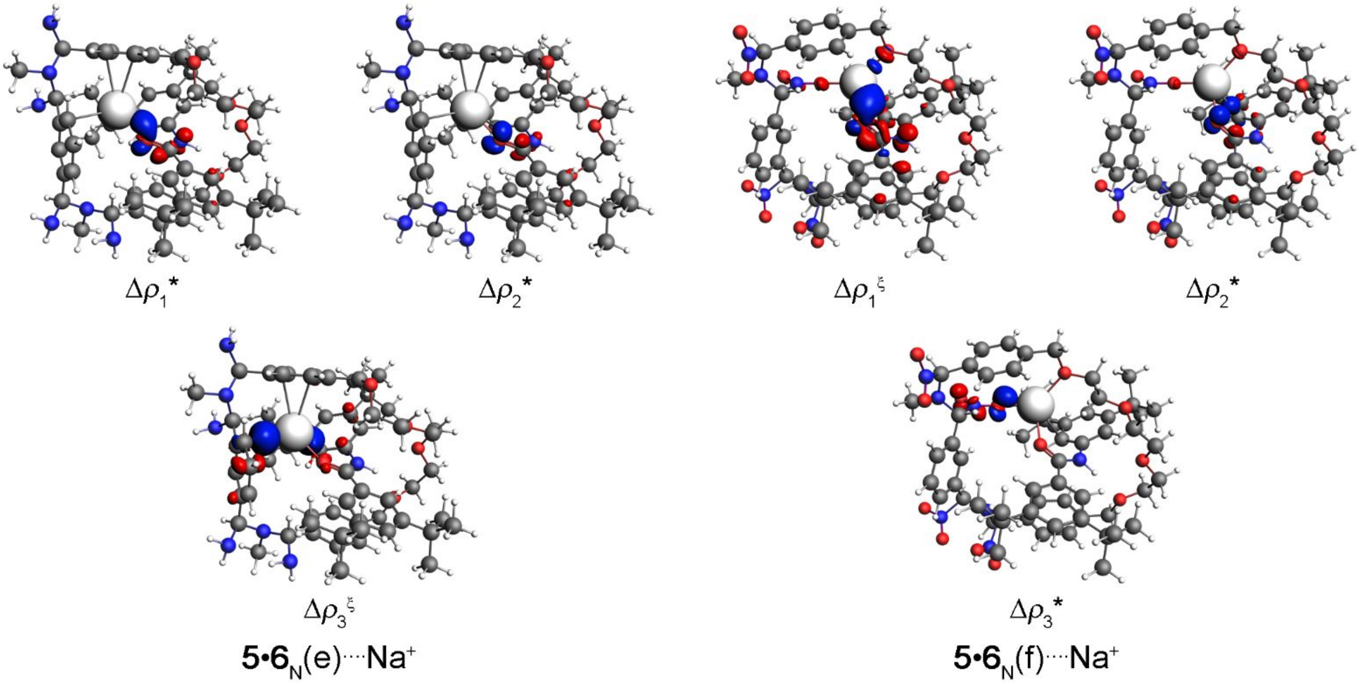

Figure S17. The main density deformation channel surface plots with isovalues = * 0.001 and $^{\xi}$ 0.0005 a.u., where the red and blue regions indicate the electron density outflow and inflow, respectively, for **5•6**$_N$(e or f)···Na^+ complexes. Color code for atoms: H = white; C = gray; N = blue; O = red; and Na = ice.

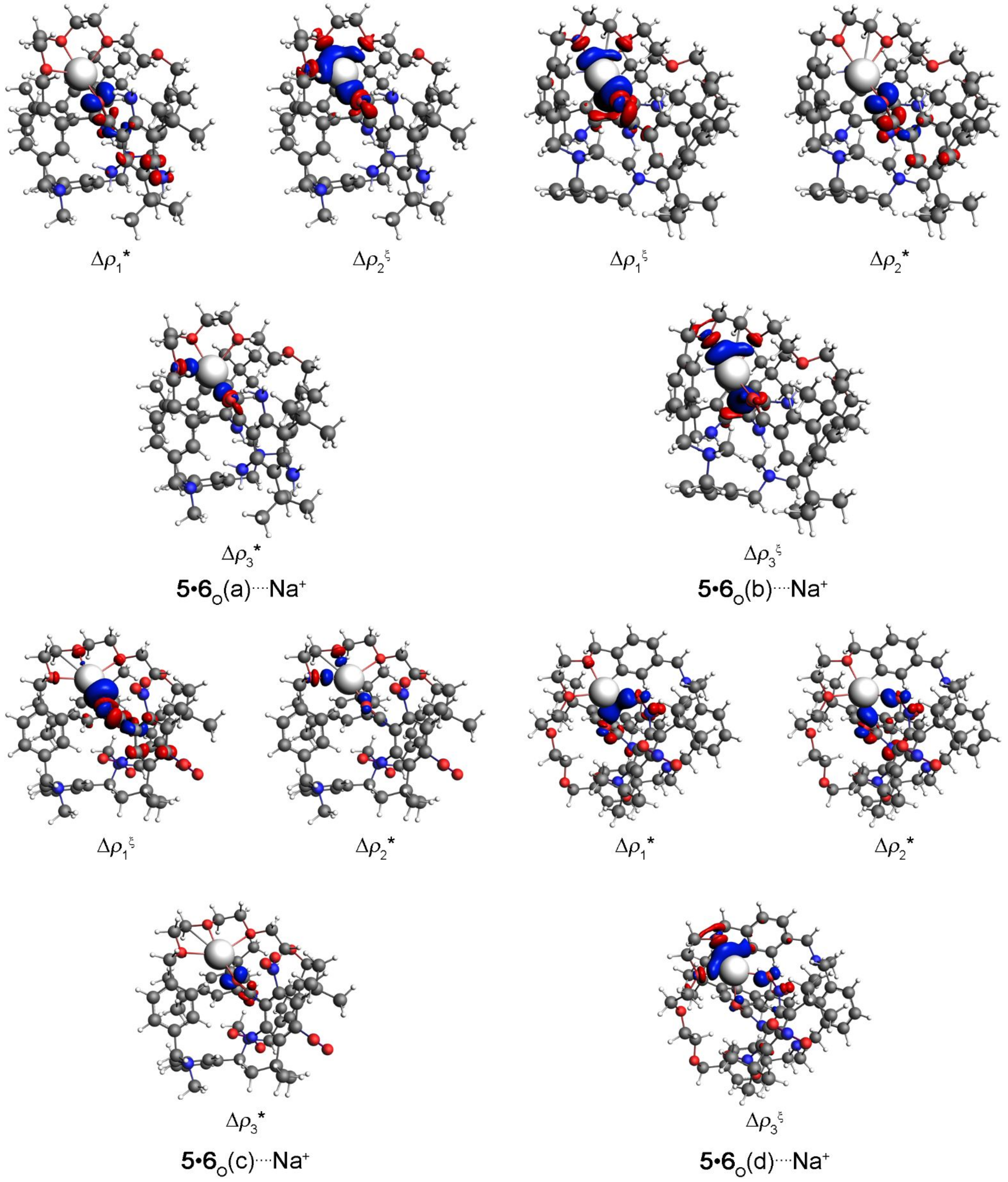

Figure S18. The main density deformation channel surface plots with isovalues = * 0.001 and $^\xi$ 0.0005 a.u., where the red and blue regions indicate the electron density outflow and inflow, respectively, for **5•6**$_O$(a–d)···Na$^+$ complexes. Color code for atoms: H = white; C = gray; N = blue; O = red; and Na = ice.

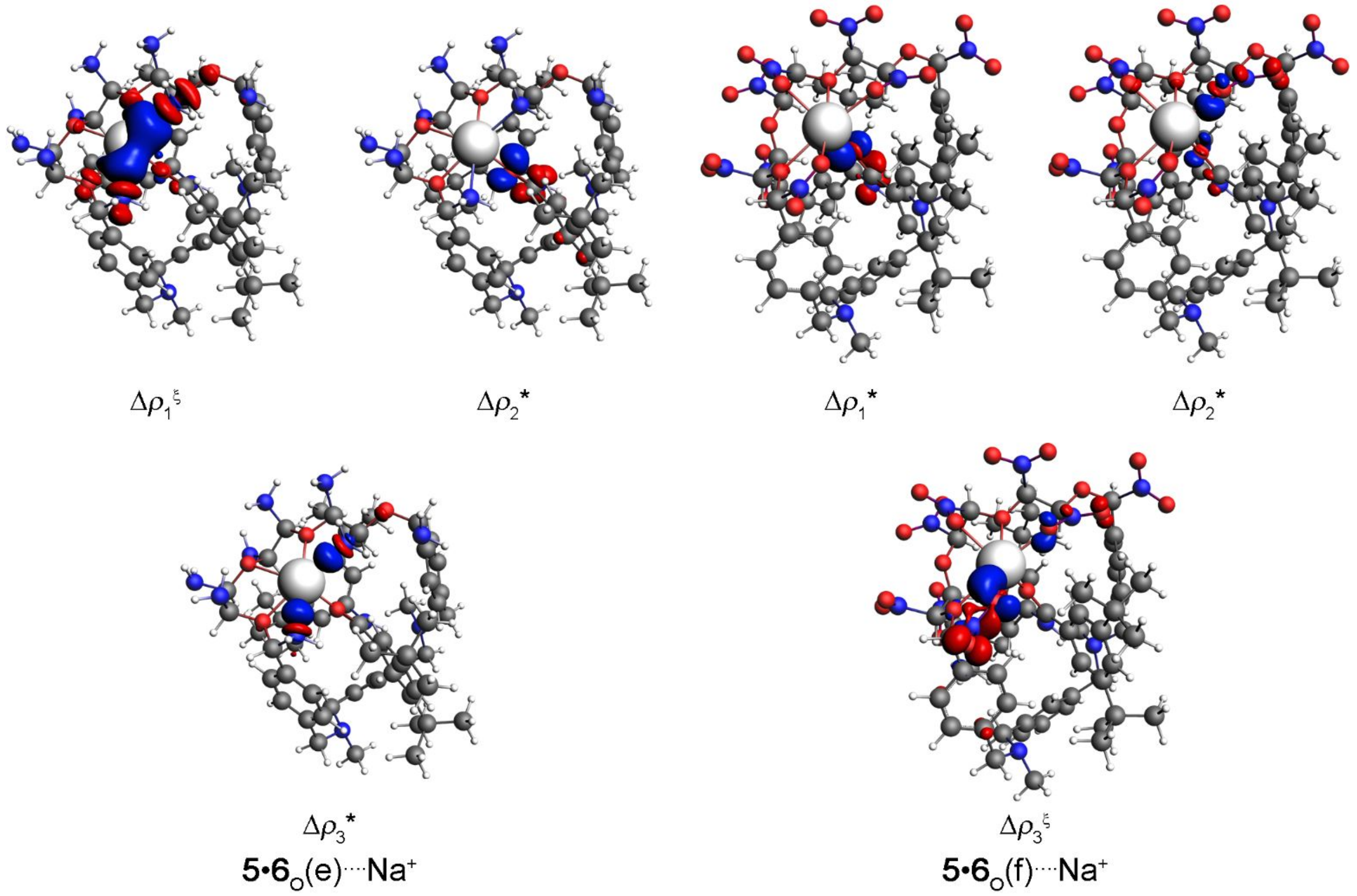

Figure S19. The main density deformation channel surface plots with isovalues = * 0.001 and $^{\xi}$ 0.0005 a.u., where the red and blue regions indicate the electron density outflow and inflow, respectively, for **5•6**$_{O}$(e or f)···Na^{+} complexes. Color code for atoms: H = white; C = gray; N = blue; O = red; and Na = ice.

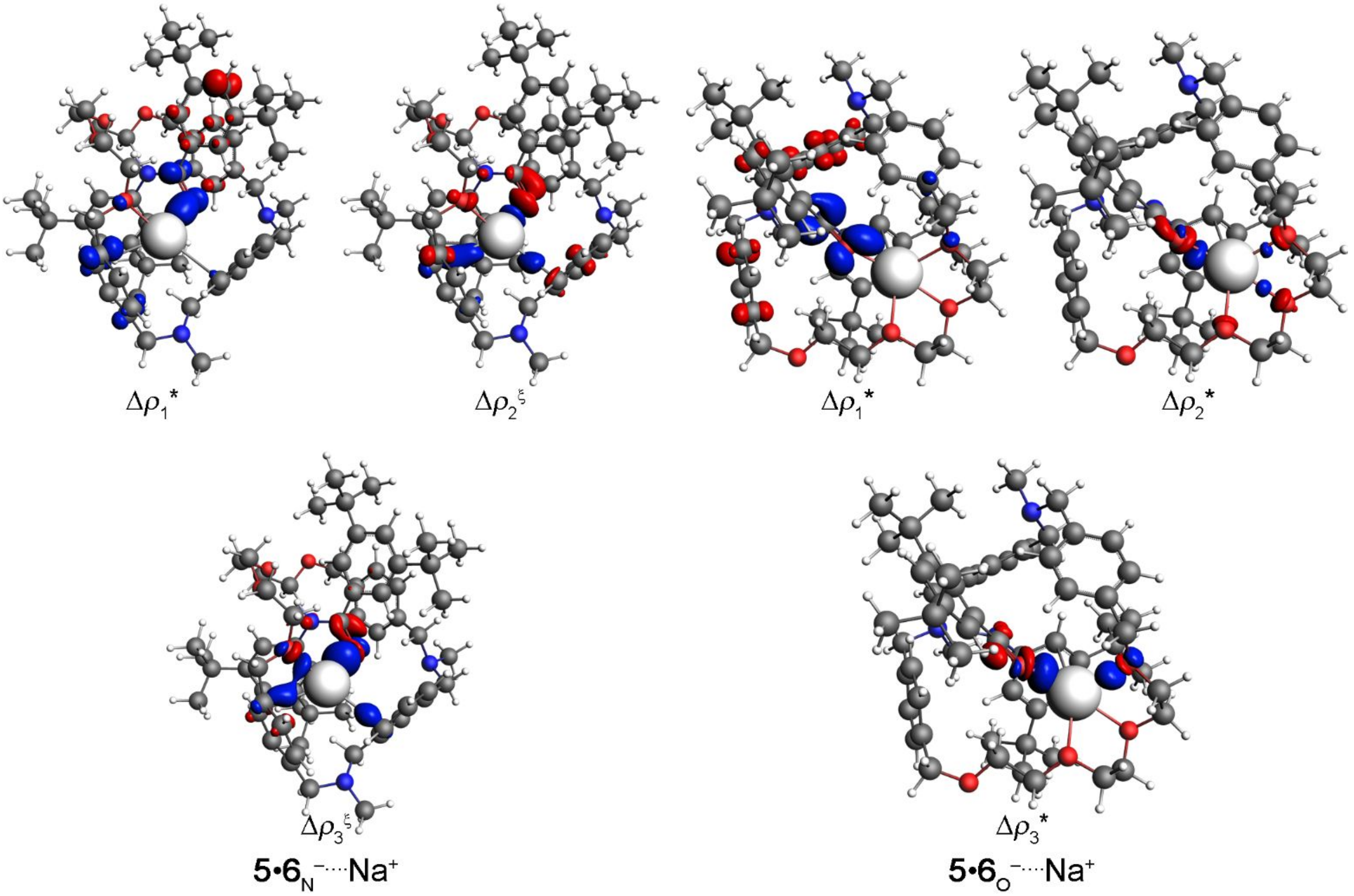

Figure S20. The main density deformation channel surface plots with isovalues = * 0.001 and $^{\xi}$ 0.0005 a.u., where the red and blue regions indicate the electron density outflow and inflow, respectively, for (**5•6**$_N$$^-$ or **5•6**$_O$$^-$)···Na$^+$ complexes. Color code for atoms: H = white; C = gray; N = blue; O = red; Na = ice.

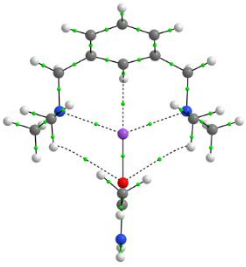

5•6$_{N,ac}$···Na$^+$

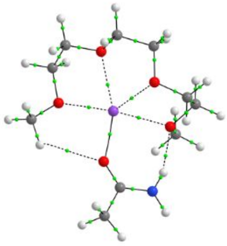

5•6$_{O,ac}$···Na$^+$

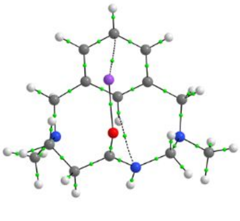

5•6$_{N,c}$···Na$^+$

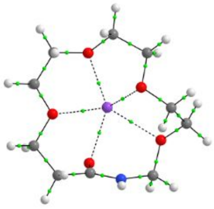

5•6$_{O,c}$···Na$^+$

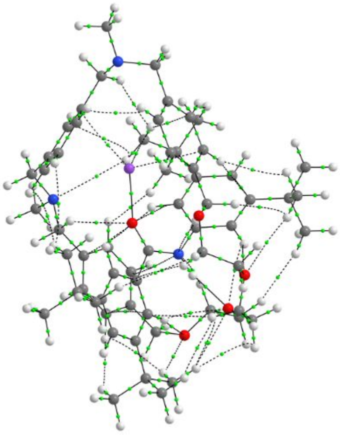

5•6$_{N}$···Na$^+$

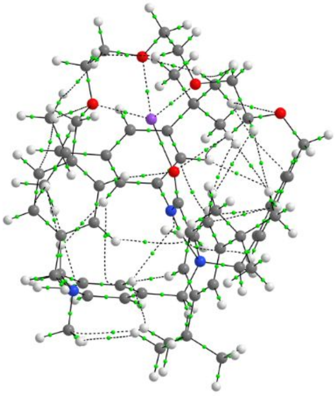

5•6$_{O}$···Na$^+$

Figure S21. Topological map containing the bond paths (continuous or dashed lines connecting the cores) and bond critical points (small green points), for the complexes (**5•6**$_{N,ac}$, **5•6**$_{N,c}$, **5•6**$_{N}$, **5•6**$_{O,ac}$, **5•6**$_{O,c}$ or **5•6**$_{O}$)···Na$^+$. Atoms color code: H = white; C = gray; N = blue; O = red; and Na = purple.

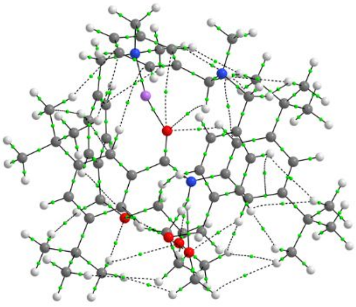

5•6$_{N}$···Li^{+}

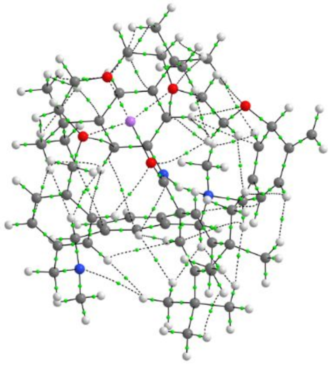

5•6$_{O}$···Li^{+}

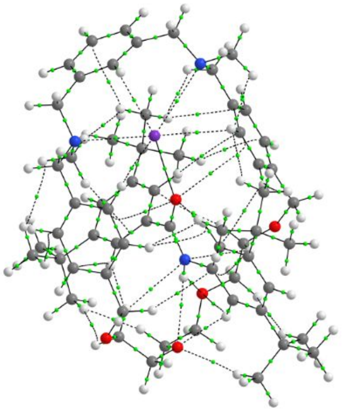

5•6$_{N}$···K^{+}

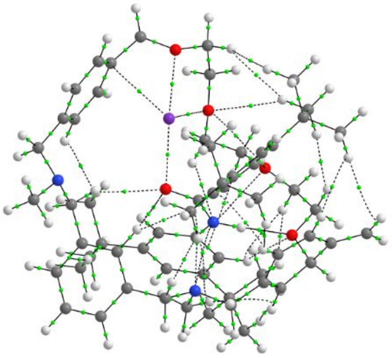

5•6$_{O}$···K^{+}

Figure S22. Topological map containing the bond paths (continuous or dashed lines connecting the cores) and bond critical points (small green points), for the complexes (**5•6**$_{N}$ or **5•6**$_{O}$)···(Li^{+} or K^{+}). Atoms color code: H = white; C = gray; N = blue; O = red; Li = pink; and K = dark purple.

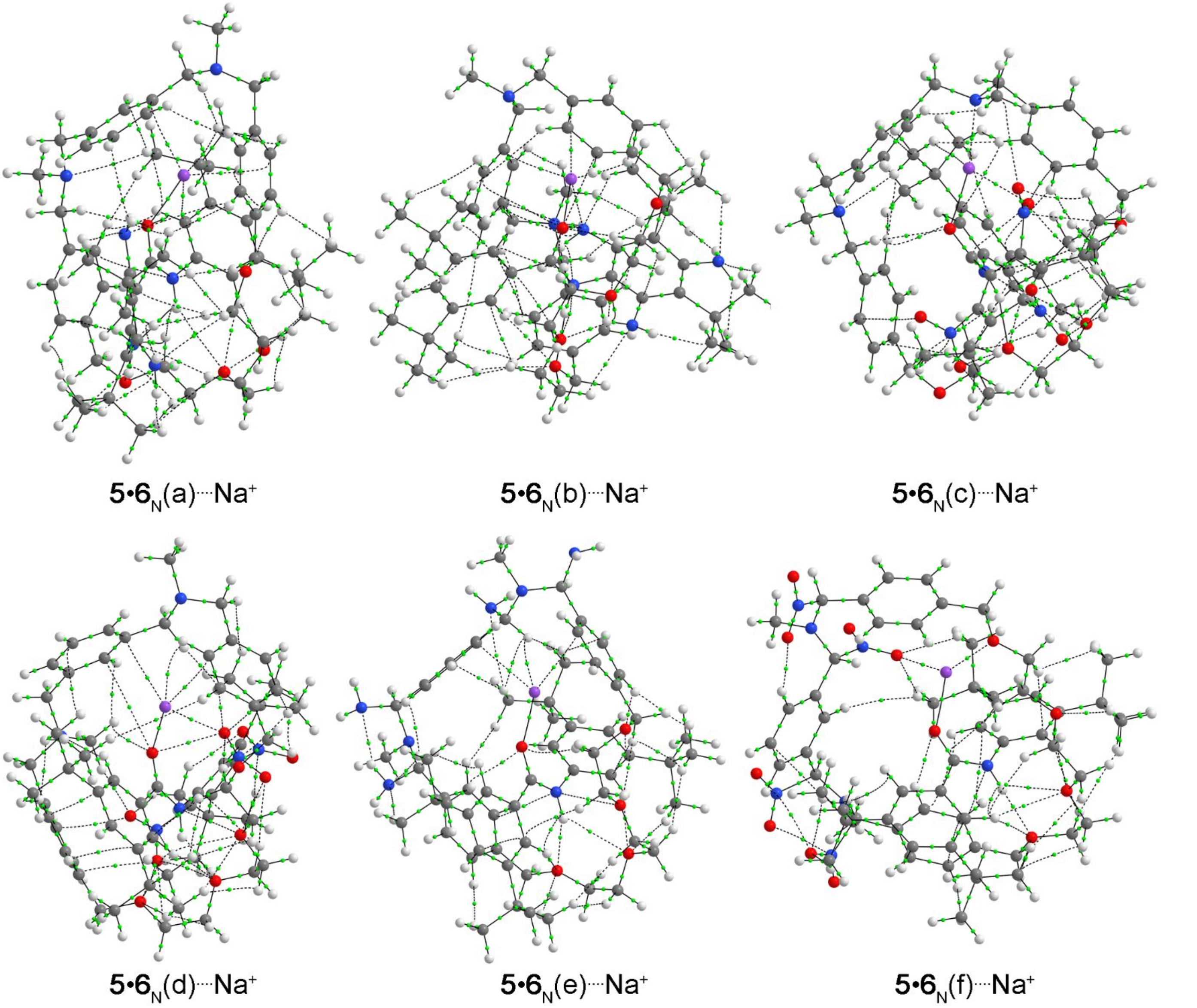

Figure S23. Topological map containing the bond paths (continuous or dashed lines connecting the cores) and bond critical points (small green points), for the complexes **5•6**$_{N}$(a–f)···Na$^+$. Atoms color code: H = white; C = gray; N = blue; O = red; and Na = purple.

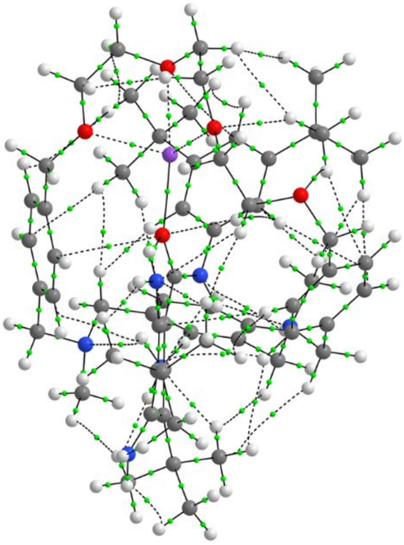

5•6$_O$(a)···Na$^+$

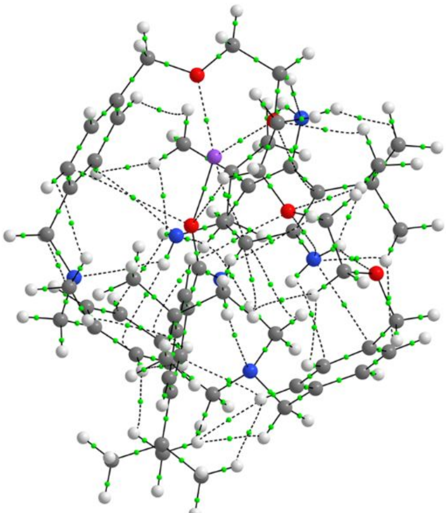

5•6$_O$(b)···Na$^+$

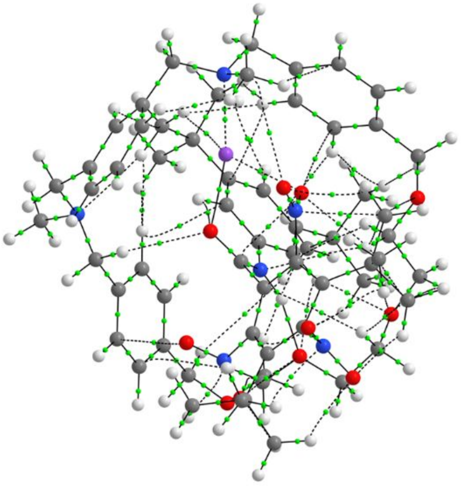

5•6$_O$(c)···Na$^+$

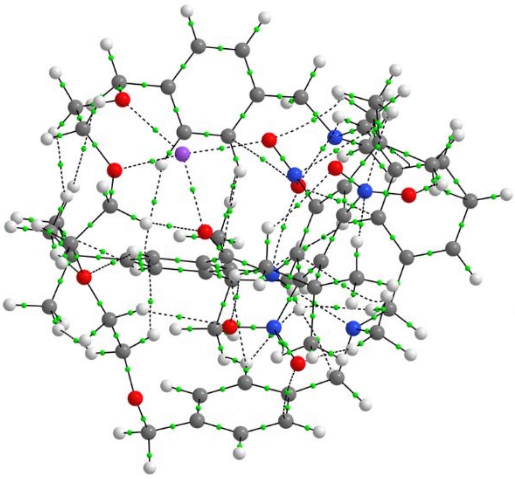

5•6$_O$(d)···Na$^+$

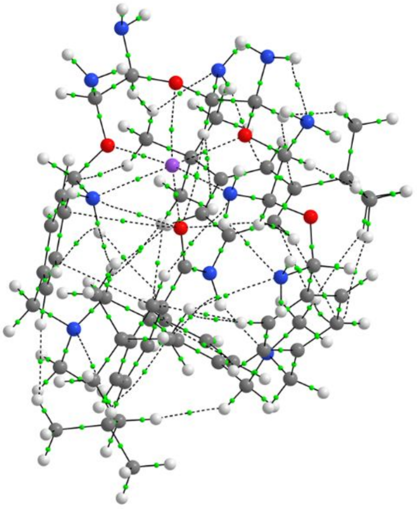

5•6$_O$(e)···Na$^+$

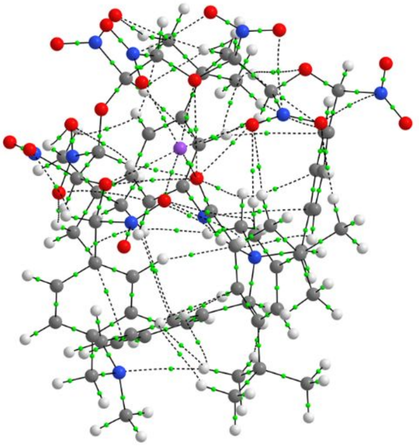

5•6$_O$(f)···Na$^+$

Figure S24. Topological map containing the bond paths (continuous or dashed lines connecting the cores) and bond critical points (small green points), for the complexes **5•6**$_O$(a–f)···Na$^+$. Atoms color code: H = white; C = gray; N = blue; O = red; and Na = purple.

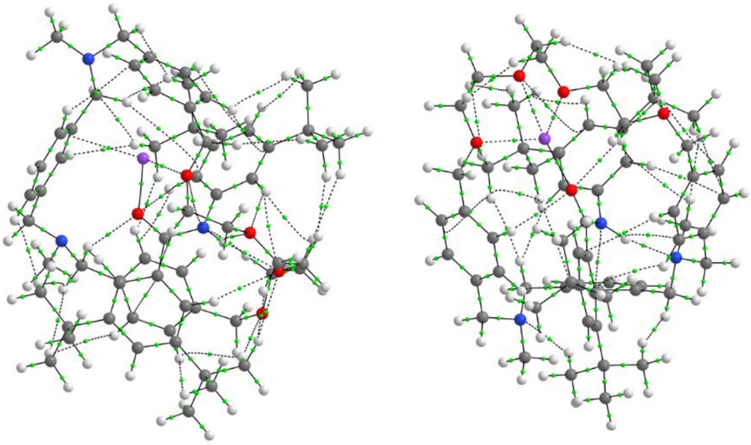

5•6$_N$$^{−}$···Na$^+$ **5•6**$_O$$^{−}$···Na$^+$

Figure S25. Topological map containing the bond paths (continuous or dashed lines connecting the cores) and bond critical points (small green points), for the complexes (**5•6**$_N$$^{−}$ or **5•6**$_O$$^{−}$)···Na$^+$. Atoms color code: H = white; C = gray; N = blue; O = red; and Na = purple.

Table S6. Ratio between the kinetic energy density, G_b, and potential energy density, V_b, $–G_b/V_b$, and electron density, ρ_b, at BCPs related to interactions between the receptors (**5•6**$_{N,ac}$, **5•6**$_{N,c}$, **5•6**$_{N}$, **5•6**$_{O,ac}$, **5•6**$_{O,c}$ or **5•6**$_{O}$) and cation (Na^+). The values of all the parameters are in a.u.

Complex	BCP	$–G_b/V_b$	ρ_b
**5•6**$_{N,ac}$···Na^+	O···Na^+	1.280	0.027
	N···Na^+	1.268	0.016
	N···Na^+	1.272	0.016
	C···Na^+	1.303	0.011
**5•6**$_{N,c}$···Na^+	O···Na^+	1.227	0.032
	C···Na^+	1.289	0.010
**5•6**$_{N}$···Na^+	O···Na^+	1.234	0.031
	C···Na^+	1.327	0.007
	C···Na^+	1.378	0.006
	H···Na^+	1.439	0.004
**5•6**$_{O,ac}$···Na^+	O···Na^+	1.271	0.025
	O···Na^+	1.383	0.014
	O···Na^+	1.353	0.017
	O···Na^+	1.345	0.017
	O···Na^+	1.323	0.019
**5•6**$_{O,c}$···Na^+	O···Na^+	1.365	0.016
	O···Na^+	1.352	0.016
	O···Na^+	1.312	0.020
	O···Na^+	1.370	0.015
	O···Na^+	1.343	0.017
**5•6**$_{O}$···Na^+	O···Na^+	1.323	0.019
	O···Na^+	1.341	0.017
	O···Na^+	1.348	0.017
	O···Na^+	1.250	0.027

Table S7. Ratio between the kinetic energy density, G_b, and potential energy density, V_b, $–G_b/V_b$, and electron density, ρ_b, at BCPs related to interactions between the receptors (**5•6**$_N$ or **5•6**$_O$) and cations (Li^+ or K^+). The values of all the parameters are in a.u.

Complex	BCP	$–G_b/V_b$	ρ_b
**5•6**$_N$···Li^+	O···Li^+	1.166	0.045
	N···Li^+	1.104	0.029
**5•6**$_N$···K^+	O···K^+	1.174	0.026
	N···K^+	1.225	0.015
	N···K^+	1.313	0.009
	C···K^+	1.299	0.010
	H···K^+	1.408	0.007
	H···K^+	1.555	0.003
**5•6**$_O$···Li^+	O···Li^+	1.254	0.022
	O···Li^+	1.215	0.024
	O···Li^+	1.295	0.016
	O···Li^+	1.187	0.036
**5•6**$_O$···K^+	O···K^+	1.272	0.016
	O···K^+	1.380	0.011
	O···K^+	1.211	0.020
	C···K^+	1.339	0.008

Table S8. Ratio between the kinetic energy density, G_b, and potential energy density, V_b, $–G_b/V_b$, and electron density, ρ_b, at BCPs related to interactions between the receptors (**5•6**$_N$(a–f)) and cation (Na^+). The values of all the parameters are in a.u.

Complex	BCP	$–G_b/V_b$	ρ_b
**5•6**$_N$(a)···Na^+	O···Na^+	1.220	0.031
	C···Na^+	1.314	0.008
	C···Na^+	1.356	0.006
	C···Na^+	1.290	0.010
	H···Na^+	1.439	0.002
**5•6**$_N$(b)···Na^+	O···Na^+	1.251	0.027
	N···Na^+	1.238	0.021
	C···Na^+	1.341	0.007
	C···Na^+	1.362	0.005
	H···Na^+	1.354	0.007
**5•6**$_N$(c)···Na^+	O···Na^+	1.258	0.025
	O···Na^+	1.383	0.014
	N···Na^+	1.194	0.024
	H···Na^+	1.451	0.002
**5•6**$_N$(d)···Na^+	O···Na^+	1.247	0.027
	O···Na^+	1.428	0.013
	C···Na^+	1.337	0.006
	H···Na^+	1.397	0.005
**5•6**$_N$(e)···Na^+	O···Na^+	1.206	0.035
	C···Na^+	1.310	0.008
	C···Na^+	1.347	0.006
	H···Na^+	1.348	0.007
**5•6**$_N$(f)···Na^+	O···Na^+	1.246	0.027
	O···Na^+	1.330	0.021
	O···Na^+	1.303	0.021

Table S9. Ratio between the kinetic energy density, G_b, and potential energy density, V_b, $-G_b/V_b$, and electron density, ρ_b, at BCPs related to interactions between the receptors (**5•6**$_O$(a–f)) and cation (Na^+). The values of all the parameters are in a.u.

Complex	BCP	$-G_b/V_b$	ρ_b
**5•6**$_O$(a)···Na^+	O···Na^+	1.343	0.018
	O···Na^+	1.322	0.019
	O···Na^+	1.362	0.016
	O···Na^+	1.262	0.025
**5•6**$_O$(b)···Na^+	O···Na^+	1.314	0.020
	O···Na^+	1.337	0.018
	O···Na^+	1.252	0.026
	C···Na^+	1.246	0.009
**5•6**$_O$(c)···Na^+	O···Na^+	1.258	0.025
	O···Na^+	1.383	0.014
	N···Na^+	1.194	0.024
	H···Na^+	1.451	0.002
**5•6**$_O$(d)···Na^+	O···Na^+	1.292	0.022
	O···Na^+	1.304	0.022
	O···Na^+	1.272	0.024
	O···Na^+	1.388	0.015
**5•6**$_O$(e)···Na^+	O···Na^+	1.320	0.019
	O···Na^+	1.397	0.007
	O···Na^+	1.322	0.020
	O···Na^+	1.308	0.024
	N···Na^+	1.305	0.015
	N···Na^+	1.313	0.014
**5•6**$_O$(f)···Na^+	O···Na^+	1.443	0.010
	O···Na^+	1.441	0.010
	O···Na^+	1.296	0.023
	O···Na^+	1.391	0.016
	O···Na^+	1.338	0.020
	O···Na^+	1.399	0.014

Table S10. Ratio between the kinetic energy density, G_b, and potential energy density, V_b, $-G_b/V_b$, and electron density, ρ_b, at BCPs related to interactions between the receptors (**5•6**$_N^-$ or **5•6**$_O^-$) and cation (Na^+). The values of all the parameters are in a.u.

Complex	BCP	$-G_b/V_b$	ρ_b
**5•6**$_N^-$···Na^+	O···Na^+	1.193	0.032
	O···Na^+	1.356	0.017
	C···Na^+	1.346	0.006
**5•6**$_O^-$···Na^+	O···Na^+	1.329	0.018
	O···Na^+	1.356	0.015
	O···Na^+	1.374	0.015
	O···Na^+	1.217	0.031

Table S11. VDD atomic charges organized in a crescent order for all complexes analyzed in this study.

1•4ac···Zn^{II}

Number	Atom	VDD
1	Zn	–0.643
4	N	–0.186
2	N	–0.157
3	N	–0.155
20	C	–0.079
56	H	–0.051
59	H	–0.051
19	C	–0.021
33	C	–0.020
64	H	–0.020
80	H	–0.007
76	H	–0.006
25	C	–0.004
82	H	–0.004
29	C	–0.003
77	H	–0.003
30	C	–0.002
95	H	–0.002
97	H	–0.002
12	C	–0.001
26	C	–0.001
39	C	–0.001
40	C	–0.001
43	C	–0.001
46	C	–0.001
11	C	0.000
27	C	0.000
31	C	0.000
41	C	0.000
42	C	0.000
44	C	0.000
45	C	0.000
28	C	0.001
32	C	0.001
68	H	0.001
74	H	0.001
85	H	0.001
86	H	0.002
89	H	0.002
94	H	0.003
34	C	0.004
79	H	0.004
73	H	0.005
91	H	0.007
92	H	0.007
38	C	0.008

83	H	0.009
88	H	0.009
54	H	0.010
18	C	0.011
70	H	0.012
61	H	0.013
24	C	0.014
71	H	0.014
21	C	0.015
22	C	0.015
35	C	0.015
62	H	0.015
81	H	0.016
37	C	0.017
65	H	0.017
84	H	0.017
87	H	0.017
96	H	0.017
67	H	0.018
90	H	0.018
93	H	0.018
16	C	0.019
5	N	0.020
7	C	0.020
47	H	0.020
78	H	0.024
57	H	0.026
58	H	0.026
63	H	0.026
66	H	0.026
69	H	0.026
36	C	0.028
72	H	0.028
60	H	0.029
13	C	0.030
75	H	0.030
10	C	0.031
17	C	0.031
15	C	0.032
23	C	0.035
8	C	0.036
14	C	0.043
9	C	0.046
51	H	0.048
50	H	0.049
6	N	0.050
55	H	0.053
53	H	0.055
52	H	0.059
48	H	0.061
49	H	0.061

1•4c····Zn^{II}

Number	Atom	VDD
43	Zn	–0.623
8	N	–0.165
5	N	–0.162
20	N	–0.117
37	H	–0.037
30	H	–0.027
21	N	–0.026
32	H	–0.017
14	C	–0.011
16	C	–0.010
6	C	–0.008
23	C	–0.007
13	C	–0.006
7	C	–0.002
15	C	–0.002
22	C	0.001
35	H	0.002
9	C	0.017
4	C	0.018
19	C	0.021
1	C	0.034
3	C	0.035
10	C	0.036
12	C	0.036
39	H	0.037
17	N	0.042
18	C	0.044
41	H	0.045
42	H	0.045
31	H	0.048
34	H	0.048
40	H	0.049
2	C	0.050
11	C	0.051
36	H	0.053
33	H	0.054
24	H	0.055
29	H	0.057
26	H	0.063
27	H	0.065
25	H	0.067
28	H	0.068
38	H	0.077

1•4····Zn^{II}

Number	Atom	VDD
1	Zn	–0.590
6	N	–0.166
5	N	–0.148

4	N	–0.147
30	H	–0.100
80	H	–0.083
89	H	–0.077
79	C	–0.071
74	H	–0.043
113	C	–0.016
34	C	–0.015
49	H	–0.011
55	H	–0.011
61	C	–0.010
62	C	–0.009
124	H	–0.009
68	H	–0.008
95	H	–0.008
16	C	–0.007
25	C	–0.007
63	H	–0.007
32	H	–0.006
51	H	–0.006
126	H	–0.006
143	H	–0.006
17	C	–0.005
115	H	–0.005
145	H	–0.005
42	C	–0.004
87	C	–0.004
28	C	–0.002
66	C	–0.002
100	C	–0.002
31	C	–0.001
35	C	–0.001
43	H	–0.001
64	C	–0.001
105	C	–0.001
122	C	–0.001
135	C	–0.001
136	C	–0.001
44	C	0.000
47	C	0.000
50	C	0.000
53	C	0.000
72	C	0.000
88	C	0.000
92	C	0.000
101	C	0.000
108	H	0.000
110	H	0.000
114	C	0.000
123	C	0.000
127	C	0.000
130	H	0.000

131	C	0.000
140	C	0.000
144	C	0.000
69	C	0.001
78	C	0.001
91	H	0.001
96	C	0.001
109	C	0.001
132	H	0.002
7	N	0.003
59	C	0.003
121	H	0.003
26	H	0.004
36	H	0.004
40	C	0.004
112	H	0.004
147	H	0.005
102	H	0.006
141	H	0.006
37	C	0.007
56	C	0.007
65	H	0.007
106	H	0.007
137	H	0.007
139	H	0.007
57	C	0.008
104	H	0.009
120	C	0.009
128	H	0.009
134	H	0.009
39	C	0.011
45	H	0.011
97	H	0.011
2	O	0.012
41	H	0.012
60	H	0.012
116	C	0.013
119	C	0.013
3	O	0.014
38	H	0.014
77	C	0.014
125	H	0.014
48	H	0.015
54	H	0.016
58	H	0.016
82	C	0.016
129	H	0.016
133	H	0.016
138	H	0.016
142	H	0.016
146	H	0.016
46	H	0.017

52	H	0.017
85	C	0.017
9	C	0.018
70	H	0.018
81	C	0.018
24	C	0.019
93	H	0.019
73	H	0.020
86	H	0.020
99	H	0.020
118	H	0.021
107	H	0.022
71	H	0.023
94	H	0.023
117	C	0.023
33	H	0.024
67	H	0.024
98	H	0.024
103	H	0.025
29	H	0.026
75	C	0.026
83	H	0.026
111	H	0.026
14	C	0.027
18	C	0.027
10	C	0.029
22	C	0.029
76	H	0.032
90	H	0.033
27	H	0.037
84	C	0.038
12	C	0.039
8	N	0.040
20	C	0.041
15	H	0.043
19	H	0.044
11	H	0.049
23	H	0.052
13	H	0.054
21	H	0.055

2•4ac···Zn^{II}

Number	Atom	VDD
1	Zn	–0.626
15	N	–0.182
14	N	–0.160
2	N	–0.158
3	N	–0.156
65	H	–0.039
26	C	–0.035
25	C	–0.034

71	H	–0.022
39	C	–0.020
76	H	–0.015
83	H	–0.008
89	H	–0.008
82	H	–0.006
91	H	–0.004
31	C	–0.003
35	C	–0.003
86	H	–0.003
101	H	–0.002
103	H	–0.002
19	C	–0.001
20	C	–0.001
33	C	–0.001
37	C	–0.001
45	C	–0.001
46	C	–0.001
49	C	–0.001
51	C	–0.001
8	C	0.000
9	C	0.000
32	C	0.000
34	C	0.000
36	C	0.000
38	C	0.000
47	C	0.000
48	C	0.000
50	C	0.000
52	C	0.000
97	H	0.000
104	H	0.002
40	C	0.003
88	H	0.003
92	H	0.003
100	H	0.003
30	C	0.005
85	H	0.005
106	H	0.006
27	C	0.007
44	C	0.007
98	H	0.007
77	H	0.008
80	H	0.008
95	H	0.008
73	H	0.009
94	H	0.009
79	H	0.010
74	H	0.012
24	C	0.013
67	H	0.013
68	H	0.013

41	C	0.014
53	H	0.014
70	H	0.014
28	C	0.015
90	H	0.015
13	C	0.016
43	C	0.016
60	H	0.016
93	H	0.016
96	H	0.016
4	C	0.017
99	H	0.017
102	H	0.017
105	H	0.017
29	C	0.018
72	H	0.021
66	H	0.022
69	H	0.022
75	H	0.022
81	H	0.023
87	H	0.023
23	C	0.025
78	H	0.025
16	N	0.026
21	C	0.026
42	C	0.026
84	H	0.026
7	C	0.027
10	C	0.027
5	C	0.030
12	C	0.032
18	C	0.032
6	C	0.039
22	C	0.039
11	C	0.041
56	H	0.043
57	H	0.044
17	N	0.045
62	H	0.047
61	H	0.048
64	H	0.049
54	H	0.051
63	H	0.053
55	H	0.054
58	H	0.056
59	H	0.056

2•4c····Zn^{II}

Number	Atom	VDD
52	Zn	–0.733
8	N	–0.149

5	N	–0.143
25	C	–0.119
37	H	–0.057
38	H	–0.043
21	C	–0.019
6	C	–0.008
49	H	–0.006
7	C	–0.005
14	C	–0.002
18	C	–0.001
13	C	0.000
16	C	0.001
15	C	0.002
17	C	0.002
26	C	0.003
43	H	0.004
40	H	0.009
24	C	0.015
27	C	0.016
20	C	0.017
9	C	0.020
4	C	0.023
22	N	0.023
48	H	0.023
19	N	0.026
45	H	0.028
50	H	0.028
41	H	0.031
46	H	0.031
1	C	0.032
10	C	0.033
12	C	0.033
3	C	0.034
36	H	0.036
44	H	0.036
42	H	0.041
47	H	0.042
39	H	0.045
2	C	0.047
11	C	0.048
28	C	0.049
30	H	0.050
23	N	0.051
35	H	0.051
29	N	0.052
32	H	0.058
33	H	0.059
31	H	0.063
34	H	0.063
51	H	0.063

2•4····Zn^{II}

Number	Atom	VDD
156	Zn	–0.610
72	N	–0.173
3	N	–0.156
4	N	–0.155
71	N	–0.148
69	H	–0.055
22	H	–0.035
86	C	–0.033
87	C	–0.029
88	H	–0.029
26	H	–0.027
101	H	–0.019
121	C	–0.017
107	H	–0.013
30	C	–0.009
50	H	–0.009
57	C	–0.009
67	H	–0.007
116	H	–0.007
118	H	–0.007
21	C	–0.006
42	H	–0.006
132	H	–0.006
134	H	–0.005
149	H	–0.005
48	H	–0.004
12	C	–0.003
13	C	–0.003
38	C	–0.003
55	C	–0.003
58	C	–0.003
68	C	–0.003
108	C	–0.003
31	C	–0.002
95	C	–0.002
123	H	–0.002
147	H	–0.002
43	C	–0.001
46	C	–0.001
78	C	–0.001
113	C	–0.001
130	C	–0.001
131	C	–0.001
143	C	–0.001
148	C	–0.001
151	H	–0.001
24	C	0.000
27	C	0.000
36	C	0.000
40	C	0.000

49	C	0.000
53	C	0.000
62	C	0.000
96	C	0.000
100	C	0.000
104	C	0.000
109	C	0.000
117	C	0.000
135	C	0.000
139	C	0.000
144	C	0.000
152	C	0.000
60	C	0.001
65	C	0.001
77	C	0.001
129	H	0.001
136	H	0.001
142	H	0.001
33	C	0.002
122	C	0.002
93	C	0.003
39	H	0.004
52	C	0.004
56	H	0.004
120	H	0.004
153	H	0.004
35	C	0.005
1	O	0.006
32	H	0.006
59	H	0.006
89	C	0.006
128	C	0.006
103	H	0.007
114	H	0.007
155	H	0.007
2	O	0.008
110	H	0.008
138	H	0.008
140	H	0.008
145	H	0.008
112	H	0.009
37	H	0.010
54	H	0.010
61	H	0.010
94	H	0.010
34	H	0.011
97	H	0.011
85	C	0.012
105	H	0.012
124	C	0.012
64	H	0.013
90	C	0.013

99	H	0.013
127	C	0.013
44	H	0.014
45	H	0.014
5	C	0.015
20	C	0.015
92	C	0.015
133	H	0.015
137	H	0.015
141	H	0.015
150	H	0.015
41	H	0.016
47	H	0.016
51	H	0.016
146	H	0.016
154	H	0.016
25	H	0.017
29	H	0.018
91	H	0.019
102	H	0.019
28	H	0.020
66	H	0.020
98	H	0.020
106	H	0.020
115	H	0.021
126	H	0.021
79	C	0.022
83	C	0.022
111	H	0.022
125	C	0.022
119	H	0.023
14	C	0.024
6	C	0.025
10	C	0.025
63	H	0.025
73	N	0.025
18	C	0.026
70	H	0.028
75	C	0.028
76	H	0.031
80	H	0.031
81	C	0.032
23	H	0.033
8	C	0.035
16	C	0.036
11	H	0.040
15	H	0.040
74	N	0.040
7	H	0.044
84	H	0.044
82	H	0.046
9	H	0.048

19	H	0.049
17	H	0.051

3•4ac····Zn^{II}

Number	Atom	VDD
1	Zn	–0.612
62	N	–0.165
60	N	–0.164
12	N	–0.155
13	N	–0.155
61	N	–0.154
32	C	–0.019
43	C	–0.019
77	H	–0.010
90	H	–0.010
85	H	–0.006
88	H	–0.006
91	H	–0.005
76	H	–0.004
70	H	–0.003
96	H	–0.003
11	C	–0.002
40	C	–0.002
71	H	–0.002
72	H	–0.002
2	C	–0.001
23	C	–0.001
24	C	–0.001
26	C	–0.001
27	C	–0.001
34	C	–0.001
49	C	–0.001
52	C	–0.001
53	C	–0.001
95	H	–0.001
112	H	–0.001
22	C	0.000
25	C	0.000
50	C	0.000
51	C	0.000
54	C	0.000
55	C	0.000
56	C	0.000
57	C	0.000
65	C	0.000
42	C	0.001
48	C	0.001
102	H	0.001
108	H	0.001
66	H	0.003
79	H	0.003

86	H	0.003
92	H	0.003
98	H	0.003
106	H	0.003
31	C	0.006
35	C	0.006
39	C	0.006
44	C	0.006
67	H	0.006
100	H	0.006
103	H	0.006
107	H	0.006
73	H	0.009
93	H	0.009
105	H	0.009
111	H	0.009
28	C	0.012
47	C	0.012
75	H	0.014
89	H	0.014
30	C	0.015
74	H	0.015
94	H	0.015
104	H	0.015
110	H	0.015
6	C	0.016
7	C	0.016
17	H	0.016
18	H	0.016
45	C	0.016
69	H	0.016
97	H	0.016
109	H	0.016
68	H	0.017
99	H	0.017
101	H	0.017
63	N	0.019
59	N	0.020
78	H	0.021
87	H	0.021
29	C	0.023
46	C	0.023
38	C	0.025
36	C	0.026
3	C	0.027
10	C	0.027
33	C	0.030
41	C	0.030
5	C	0.032
8	C	0.032
37	C	0.036
58	N	0.040

64	N	0.040
4	C	0.041
9	C	0.041
80	H	0.044
84	H	0.044
14	H	0.045
21	H	0.045
81	H	0.046
83	H	0.046
82	H	0.051
15	H	0.056
16	H	0.056
19	H	0.056
20	H	0.056

3•4c····Zn^{II}

Number	Atom	VDD
1	Zn	–0.652
23	N	–0.157
30	N	–0.151
9	N	–0.144
44	H	–0.064
2	C	–0.051
7	C	–0.044
32	C	–0.037
58	H	–0.028
33	C	–0.015
36	H	–0.011
14	C	–0.004
8	C	–0.001
15	C	–0.001
19	C	–0.001
16	C	0.000
25	C	0.000
17	C	0.001
18	C	0.002
20	C	0.005
53	H	0.010
46	H	0.012
3	C	0.013
10	C	0.014
6	N	0.017
31	N	0.017
43	H	0.019
4	C	0.020
29	C	0.020
5	C	0.023
51	H	0.027
11	C	0.029
13	C	0.029
26	C	0.030

48	H	0.030
49	H	0.031
28	C	0.034
37	H	0.034
45	H	0.035
50	H	0.036
21	C	0.037
38	H	0.038
42	H	0.038
52	H	0.038
24	N	0.040
34	N	0.041
47	H	0.041
22	N	0.043
12	C	0.046
27	C	0.046
55	H	0.053
39	H	0.055
41	H	0.055
54	H	0.057
57	H	0.059
40	H	0.061
56	H	0.061
35	N	0.065

3•4···Zn^{II}

Number	Atom	VDD
162	Zn	–0.594
153	N	–0.158
65	N	–0.151
64	N	–0.150
155	N	–0.150
154	N	–0.143
10	H	–0.060
54	H	–0.042
112	C	–0.018
95	C	–0.016
32	H	–0.011
77	H	–0.011
124	H	–0.011
149	H	–0.011
19	C	–0.009
42	C	–0.009
18	C	–0.008
29	H	–0.008
120	H	–0.008
13	H	–0.007
146	H	–0.006
1	C	–0.005
20	H	–0.005
87	H	–0.005

94	H	–0.005
132	H	–0.005
136	H	–0.005
21	C	–0.004
43	C	–0.004
125	H	–0.004
9	C	–0.003
63	C	–0.003
161	H	–0.003
53	C	–0.002
107	C	–0.002
28	C	–0.001
40	C	–0.001
47	C	–0.001
55	H	–0.001
71	C	–0.001
79	C	–0.001
80	C	–0.001
84	C	–0.001
119	C	–0.001
121	C	–0.001
130	C	–0.001
139	C	–0.001
15	C	0.000
31	C	0.000
34	C	0.000
44	H	0.000
45	C	0.000
67	C	0.000
75	C	0.000
85	C	0.000
122	C	0.000
126	C	0.000
131	C	0.000
135	C	0.000
143	C	0.000
147	C	0.000
158	C	0.000
12	C	0.001
38	C	0.001
93	C	0.001
50	C	0.002
70	O	0.002
98	C	0.002
111	H	0.002
114	H	0.003
127	H	0.003
133	H	0.003
22	H	0.004
26	C	0.004
66	O	0.004
145	H	0.004

23	C	0.005
24	C	0.005
37	C	0.005
78	H	0.005
88	H	0.005
138	H	0.005
141	H	0.005
48	H	0.006
82	H	0.006
106	C	0.006
110	C	0.006
142	H	0.006
41	H	0.007
72	H	0.007
81	H	0.007
99	C	0.007
113	C	0.007
148	H	0.007
46	H	0.008
73	H	0.008
128	H	0.009
160	H	0.009
39	H	0.010
27	H	0.011
92	C	0.011
118	C	0.011
36	H	0.012
69	H	0.012
52	H	0.013
76	H	0.013
123	H	0.013
150	H	0.013
8	C	0.014
25	H	0.014
35	H	0.014
74	H	0.014
89	C	0.014
115	C	0.014
129	H	0.014
159	H	0.014
56	C	0.015
68	H	0.015
83	H	0.015
86	H	0.015
134	H	0.015
137	H	0.015
140	H	0.015
144	H	0.015
16	H	0.016
30	H	0.016
33	H	0.016
156	N	0.017

51	H	0.018
90	C	0.019
116	C	0.019
117	H	0.019
14	H	0.020
17	H	0.020
91	H	0.020
152	N	0.020
100	C	0.022
104	C	0.023
61	C	0.024
2	C	0.025
49	H	0.025
6	C	0.026
57	C	0.027
96	C	0.027
108	C	0.027
97	H	0.029
102	C	0.030
11	H	0.032
101	H	0.032
4	C	0.036
151	N	0.036
157	N	0.036
59	C	0.037
3	H	0.041
62	H	0.041
109	H	0.041
105	H	0.043
103	H	0.045
58	H	0.047
7	H	0.048
5	H	0.051
60	H	0.051

1•4····Cd^{II}

Number	Atom	VDD
1	Cd	–0.500
6	N	–0.150
5	N	–0.142
4	N	–0.139
79	C	–0.102
91	H	–0.100
30	H	–0.081
74	H	–0.067
80	H	–0.057
26	H	–0.025
113	C	–0.016
49	H	–0.012
34	C	–0.011
55	H	–0.011

61	C	–0.010
89	H	–0.008
124	H	–0.008
25	C	–0.007
62	C	–0.007
97	H	–0.007
115	H	–0.007
126	H	–0.007
143	H	–0.006
145	H	–0.006
16	C	–0.005
17	C	–0.005
51	H	–0.003
87	C	–0.003
100	C	–0.003
35	C	–0.002
63	H	–0.002
64	C	–0.002
66	C	–0.002
68	H	–0.002
78	C	–0.002
101	C	–0.002
28	C	–0.001
42	C	–0.001
50	C	–0.001
92	C	–0.001
96	C	–0.001
114	C	–0.001
122	C	–0.001
135	C	–0.001
136	C	–0.001
44	C	0.000
47	C	0.000
53	C	0.000
72	C	0.000
88	C	0.000
123	C	0.000
127	C	0.000
130	H	0.000
131	C	0.000
132	H	0.000
140	C	0.000
144	C	0.000
31	C	0.001
59	C	0.001
69	C	0.001
105	C	0.001
109	C	0.001
110	H	0.001
121	H	0.001
36	H	0.002
108	H	0.002

32	H	0.003
43	H	0.003
77	C	0.003
112	H	0.003
56	C	0.004
147	H	0.004
37	C	0.005
40	C	0.005
141	H	0.005
57	C	0.006
65	H	0.006
102	H	0.006
120	C	0.006
137	H	0.006
139	H	0.007
39	C	0.009
104	H	0.009
128	H	0.009
134	H	0.009
106	H	0.010
2	O	0.011
3	O	0.011
38	H	0.011
45	H	0.011
60	H	0.011
95	H	0.011
7	N	0.012
41	H	0.012
116	C	0.012
119	C	0.012
9	C	0.013
24	C	0.013
48	H	0.013
125	H	0.013
29	H	0.014
58	H	0.014
82	C	0.014
85	C	0.015
129	H	0.015
133	H	0.015
138	H	0.015
54	H	0.016
142	H	0.016
146	H	0.016
46	H	0.017
52	H	0.018
73	H	0.018
81	C	0.019
117	C	0.019
71	H	0.020
86	H	0.020
118	H	0.020

70	H	0.021
93	H	0.021
33	H	0.022
94	H	0.023
98	H	0.023
99	H	0.023
103	H	0.024
14	C	0.025
18	C	0.025
22	C	0.026
107	H	0.026
10	C	0.027
67	H	0.027
75	C	0.027
83	H	0.027
111	H	0.027
76	H	0.029
8	N	0.036
12	C	0.036
20	C	0.037
90	H	0.037
27	H	0.038
15	H	0.041
19	H	0.041
11	H	0.046
84	C	0.046
23	H	0.047
13	H	0.051
21	H	0.051

2•4····Cd^{II}

Number	Atom	VDD
156	Cd	–0.480
72	N	–0.170
3	N	–0.151
4	N	–0.149
71	N	–0.142
69	H	–0.076
26	H	–0.052
87	C	–0.044
22	H	–0.040
88	H	–0.038
86	C	–0.032
101	H	–0.024
121	C	–0.017
107	H	–0.014
67	H	–0.013
30	C	–0.012
57	C	–0.012
21	C	–0.008
50	H	–0.008

116	H	–0.008
42	H	–0.007
132	H	–0.007
134	H	–0.005
149	H	–0.005
48	H	–0.004
58	C	–0.004
68	C	–0.004
123	H	–0.004
12	C	–0.003
13	C	–0.003
38	C	–0.003
55	C	–0.003
95	C	–0.003
118	H	–0.003
31	C	–0.002
108	C	–0.002
147	H	–0.002
24	C	–0.001
43	C	–0.001
46	C	–0.001
62	C	–0.001
77	C	–0.001
78	C	–0.001
100	C	–0.001
117	C	–0.001
130	C	–0.001
131	C	–0.001
143	C	–0.001
148	C	–0.001
151	H	–0.001
27	C	0.000
40	C	0.000
49	C	0.000
65	C	0.000
96	C	0.000
104	C	0.000
109	C	0.000
113	C	0.000
114	H	0.000
129	H	0.000
135	C	0.000
139	C	0.000
142	H	0.000
144	C	0.000
152	C	0.000
36	C	0.001
53	C	0.001
136	H	0.001
60	C	0.002
122	C	0.002
39	H	0.003

112	H	0.003
33	C	0.004
153	H	0.004
52	C	0.005
56	H	0.005
59	H	0.005
128	C	0.005
32	H	0.006
35	C	0.006
89	C	0.006
93	C	0.006
85	C	0.007
145	H	0.007
155	H	0.007
1	O	0.008
103	H	0.008
138	H	0.008
140	H	0.008
2	O	0.009
5	C	0.010
20	C	0.010
37	H	0.010
90	C	0.010
54	H	0.011
61	H	0.011
120	H	0.011
64	H	0.012
97	H	0.012
105	H	0.012
110	H	0.012
124	C	0.012
34	H	0.013
45	H	0.013
94	H	0.013
127	C	0.013
44	H	0.014
99	H	0.014
133	H	0.014
137	H	0.015
141	H	0.015
150	H	0.015
154	H	0.015
41	H	0.016
47	H	0.016
146	H	0.016
51	H	0.017
92	C	0.017
25	H	0.018
73	N	0.019
91	H	0.019
102	H	0.019
106	H	0.019

28	H	0.020
29	H	0.020
79	C	0.020
126	H	0.020
83	C	0.021
98	H	0.021
119	H	0.021
125	C	0.021
66	H	0.022
111	H	0.022
14	C	0.023
115	H	0.023
6	C	0.024
10	C	0.024
18	C	0.025
63	H	0.025
75	C	0.026
70	H	0.028
76	H	0.028
80	H	0.028
81	C	0.030
8	C	0.033
16	C	0.035
23	H	0.035
74	N	0.036
11	H	0.038
15	H	0.039
7	H	0.042
84	H	0.042
82	H	0.044
19	H	0.046
9	H	0.048
17	H	0.049

3•4····Cd^{II}

Number	Atom	VDD
162	Cd	–0.455
153	N	–0.164
155	N	–0.159
64	N	–0.150
65	N	–0.150
154	N	–0.141
54	H	–0.082
10	H	–0.081
112	C	–0.017
95	C	–0.016
32	H	–0.011
42	C	–0.011
19	C	–0.010
13	H	–0.009
20	H	–0.009

124	H	–0.009
18	C	–0.008
120	H	–0.008
29	H	–0.007
77	H	–0.007
78	H	–0.007
94	H	–0.007
125	H	–0.007
63	C	–0.006
146	H	–0.006
87	H	–0.005
1	C	–0.004
21	C	–0.004
43	C	–0.004
9	C	–0.003
107	C	–0.003
40	C	–0.002
53	C	–0.002
136	H	–0.002
138	H	–0.002
28	C	–0.001
47	C	–0.001
79	C	–0.001
80	C	–0.001
84	C	–0.001
93	C	–0.001
121	C	–0.001
130	C	–0.001
135	C	–0.001
12	C	0.000
15	C	0.000
31	C	0.000
34	C	0.000
67	C	0.000
71	C	0.000
72	H	0.000
75	C	0.000
85	C	0.000
98	C	0.000
119	C	0.000
122	C	0.000
126	C	0.000
131	C	0.000
139	C	0.000
143	C	0.000
147	C	0.000
158	C	0.000
161	H	0.000
38	C	0.001
50	C	0.001
127	H	0.001
132	H	0.001

149	H	0.001
45	C	0.002
70	O	0.002
111	H	0.002
142	H	0.002
22	H	0.003
44	H	0.003
52	H	0.003
106	C	0.003
114	H	0.003
145	H	0.003
41	H	0.004
99	C	0.004
23	C	0.005
37	C	0.005
66	O	0.005
88	H	0.005
133	H	0.005
26	C	0.006
81	H	0.006
82	H	0.006
113	C	0.006
141	H	0.006
24	C	0.007
110	C	0.007
48	H	0.008
56	C	0.008
39	H	0.009
73	H	0.009
128	H	0.009
148	H	0.009
160	H	0.009
8	C	0.010
46	H	0.010
92	C	0.010
118	C	0.010
36	H	0.011
123	H	0.012
27	H	0.013
69	H	0.013
76	H	0.013
89	C	0.013
129	H	0.013
159	H	0.013
16	H	0.014
25	H	0.014
74	H	0.014
115	C	0.014
150	H	0.014
156	N	0.014
35	H	0.015
68	H	0.015

83	H	0.015
86	H	0.015
134	H	0.015
137	H	0.015
140	H	0.015
144	H	0.015
152	N	0.015
33	H	0.016
30	H	0.017
90	C	0.019
91	H	0.019
117	H	0.019
14	H	0.020
116	C	0.020
17	H	0.021
51	H	0.021
100	C	0.021
104	C	0.022
2	C	0.024
61	C	0.024
6	C	0.025
49	H	0.025
55	H	0.025
57	C	0.025
96	C	0.025
108	C	0.025
97	H	0.027
101	H	0.028
102	C	0.028
11	H	0.033
59	C	0.034
151	N	0.034
4	C	0.035
157	N	0.035
109	H	0.038
62	H	0.039
3	H	0.040
105	H	0.040
103	H	0.042
58	H	0.044
7	H	0.045
60	H	0.049
5	H	0.050

1•4^{-}····Zn^{II}

Number	Atom	VDD
1	Zn	–0.957
5	N	–0.131
6	N	–0.129
4	N	–0.121
26	H	–0.051

30	H	–0.041
74	H	–0.037
80	H	–0.030
89	H	–0.022
78	C	–0.016
7	N	–0.014
79	C	–0.012
73	H	–0.010
115	H	–0.009
51	H	–0.008
124	H	–0.008
49	H	–0.007
110	H	–0.007
25	C	–0.006
34	C	–0.006
55	H	–0.006
61	C	–0.005
108	H	–0.005
126	H	–0.005
91	H	–0.004
59	C	–0.003
95	H	–0.003
143	H	–0.003
57	C	–0.002
66	C	–0.002
72	C	–0.002
87	C	–0.002
100	C	–0.002
114	C	–0.002
135	C	–0.002
145	H	–0.002
28	C	–0.001
50	C	–0.001
53	C	–0.001
88	C	–0.001
101	C	–0.001
122	C	–0.001
123	C	–0.001
136	C	–0.001
144	C	–0.001
31	C	0.000
44	C	0.000
47	C	0.000
56	C	0.000
69	C	0.000
92	C	0.000
96	C	0.000
105	C	0.000
109	C	0.000
127	C	0.000
131	C	0.000
140	C	0.000

32	H	0.001
60	H	0.002
62	C	0.002
64	C	0.002
130	H	0.002
68	H	0.003
113	C	0.003
132	H	0.003
3	O	0.004
58	H	0.004
97	H	0.004
112	H	0.004
147	H	0.005
141	H	0.006
42	C	0.007
102	H	0.008
106	H	0.008
137	H	0.008
139	H	0.008
35	C	0.009
43	H	0.009
86	H	0.009
104	H	0.009
16	C	0.010
39	C	0.010
40	C	0.010
63	H	0.010
17	C	0.011
65	H	0.011
93	H	0.011
99	H	0.012
2	O	0.013
45	H	0.013
134	H	0.013
36	H	0.014
85	C	0.014
128	H	0.014
37	C	0.015
121	H	0.015
24	C	0.016
29	H	0.016
84	C	0.016
119	C	0.016
9	C	0.017
48	H	0.017
52	H	0.017
125	H	0.017
41	H	0.018
90	H	0.018
46	H	0.019
54	H	0.019
94	H	0.019

103	H	0.019
107	H	0.019
129	H	0.019
70	H	0.020
98	H	0.020
111	H	0.020
120	C	0.020
133	H	0.020
71	H	0.021
138	H	0.021
142	H	0.021
146	H	0.021
38	H	0.022
81	C	0.022
116	C	0.022
33	H	0.023
18	C	0.024
14	C	0.026
82	C	0.026
67	H	0.027
83	H	0.027
77	C	0.028
8	N	0.030
117	C	0.030
118	H	0.030
20	C	0.033
75	C	0.033
27	H	0.034
76	H	0.034
12	C	0.035
22	C	0.040
10	C	0.041
19	H	0.041
15	H	0.044
21	H	0.052
13	H	0.055
11	H	0.056
23	H	0.058

2•4^{-}····Zn^{II}

Number	Atom	VDD
156	Zn	–0.617
4	N	–0.176
3	N	–0.175
72	N	–0.171
71	N	–0.163
69	H	–0.056
22	H	–0.035
26	H	–0.033
86	C	–0.032
88	H	–0.026

87	C	–0.024
101	H	–0.016
13	C	–0.012
107	H	–0.011
12	C	–0.010
50	H	–0.009
67	H	–0.009
30	C	–0.007
116	H	–0.007
21	C	–0.006
48	H	–0.006
42	H	–0.005
57	C	–0.005
78	C	–0.005
118	H	–0.005
68	C	–0.003
108	C	–0.003
132	H	–0.003
149	H	–0.003
95	C	–0.002
130	C	–0.002
134	H	–0.002
143	C	–0.002
24	C	–0.001
31	C	–0.001
38	C	–0.001
43	C	–0.001
58	C	–0.001
62	C	–0.001
100	C	–0.001
113	C	–0.001
131	C	–0.001
148	C	–0.001
152	C	–0.001
27	C	0.000
33	C	0.000
40	C	0.000
46	C	0.000
49	C	0.000
53	C	0.000
55	C	0.000
65	C	0.000
96	C	0.000
104	C	0.000
109	C	0.000
117	C	0.000
135	C	0.000
139	C	0.000
144	C	0.000
147	H	0.000
36	C	0.001
60	C	0.001

52	C	0.002
93	C	0.002
151	H	0.002
1	O	0.003
35	C	0.003
136	H	0.003
142	H	0.004
2	O	0.005
121	C	0.005
32	H	0.006
89	C	0.006
103	H	0.006
114	H	0.006
123	H	0.006
153	H	0.006
39	H	0.007
56	H	0.007
77	C	0.007
120	H	0.007
34	H	0.008
59	H	0.008
94	H	0.008
155	H	0.008
20	C	0.009
61	H	0.009
110	H	0.009
112	H	0.009
122	C	0.009
138	H	0.009
140	H	0.009
145	H	0.009
5	C	0.010
97	H	0.010
129	H	0.010
37	H	0.011
54	H	0.011
85	C	0.011
105	H	0.012
99	H	0.013
44	H	0.014
64	H	0.014
90	C	0.014
92	C	0.014
45	H	0.015
128	C	0.015
47	H	0.016
25	H	0.017
51	H	0.017
81	C	0.017
29	H	0.018
41	H	0.018
102	H	0.018

127	C	0.018
133	H	0.018
6	C	0.019
28	H	0.019
106	H	0.019
150	H	0.019
91	H	0.020
98	H	0.020
115	H	0.020
137	H	0.020
141	H	0.020
146	H	0.020
154	H	0.020
10	C	0.021
14	C	0.021
18	C	0.021
66	H	0.021
124	C	0.021
111	H	0.022
119	H	0.022
63	H	0.023
73	N	0.023
8	C	0.024
16	C	0.024
70	H	0.025
79	C	0.025
83	C	0.029
23	H	0.030
80	H	0.030
126	H	0.032
15	H	0.037
74	N	0.037
125	C	0.037
11	H	0.038
76	H	0.038
82	H	0.038
7	H	0.042
9	H	0.042
17	H	0.043
75	C	0.043
19	H	0.047
84	H	0.048

3•4^{-}····Zn^{II}

Number	Atom	VDD
162	Zn	–0.598
154	N	–0.181
65	N	–0.175
64	N	–0.172
153	N	–0.154
155	N	–0.136

10	H	–0.065
54	H	–0.044
63	C	–0.021
1	C	–0.018
32	H	–0.010
13	H	–0.009
29	H	–0.009
124	H	–0.008
161	H	–0.008
19	C	–0.007
55	H	–0.006
18	C	–0.005
42	C	–0.005
106	C	–0.005
20	H	–0.004
78	H	–0.004
87	H	–0.004
146	H	–0.004
77	H	–0.003
9	C	–0.002
21	C	–0.002
43	C	–0.002
79	C	–0.002
84	C	–0.002
121	C	–0.002
130	C	–0.002
15	C	–0.001
31	C	–0.001
45	C	–0.001
47	C	–0.001
53	C	–0.001
75	C	–0.001
80	C	–0.001
126	C	–0.001
135	C	–0.001
12	C	0.000
28	C	0.000
34	C	0.000
67	C	0.000
71	C	0.000
85	C	0.000
99	C	0.000
122	C	0.000
131	C	0.000
138	H	0.000
139	C	0.000
143	C	0.000
147	C	0.000
158	C	0.000
66	O	0.001
136	H	0.001
26	C	0.002

37	C	0.002
40	C	0.002
50	C	0.002
72	H	0.002
94	H	0.002
24	C	0.003
38	C	0.003
44	H	0.003
70	O	0.003
95	C	0.003
107	C	0.003
149	H	0.003
23	C	0.004
93	C	0.004
102	C	0.004
112	C	0.004
132	H	0.004
142	H	0.004
22	H	0.005
48	H	0.005
120	H	0.005
145	H	0.005
46	H	0.006
56	C	0.006
88	H	0.006
98	C	0.006
8	C	0.007
82	H	0.007
133	H	0.007
141	H	0.007
160	H	0.007
81	H	0.008
125	H	0.008
73	H	0.009
127	H	0.009
128	H	0.009
148	H	0.009
27	H	0.010
41	H	0.010
25	H	0.011
39	H	0.011
111	H	0.011
6	C	0.012
52	H	0.012
57	C	0.012
69	H	0.012
113	C	0.012
119	C	0.012
35	H	0.013
36	H	0.013
114	H	0.013
30	H	0.015

89	C	0.015
16	H	0.016
33	H	0.016
68	H	0.016
76	H	0.016
123	H	0.016
17	H	0.017
51	H	0.017
61	C	0.017
118	C	0.017
129	H	0.017
159	H	0.017
2	C	0.018
14	H	0.018
74	H	0.018
83	H	0.018
86	H	0.018
110	C	0.018
144	H	0.018
150	H	0.018
134	H	0.019
137	H	0.019
140	H	0.019
59	C	0.020
115	C	0.020
152	N	0.020
92	C	0.021
4	C	0.023
49	H	0.024
156	N	0.025
11	H	0.028
100	C	0.028
91	H	0.029
103	H	0.029
90	C	0.031
101	H	0.031
117	H	0.031
62	H	0.032
104	C	0.033
151	N	0.033
157	N	0.033
3	H	0.034
97	H	0.034
116	C	0.034
58	H	0.035
7	H	0.037
60	H	0.037
5	H	0.039
96	C	0.039
108	C	0.039
105	H	0.046
109	H	0.049

1•4a···Zn^{II}

Number	Atom	VDD
1	Zn	–0.606
6	N	–0.176
4	N	–0.159
5	N	–0.156
79	H	–0.074
78	C	–0.072
73	H	–0.044
88	H	–0.044
26	H	–0.036
29	H	–0.034
94	H	–0.022
112	C	–0.018
61	C	–0.013
77	C	–0.013
34	C	–0.012
49	H	–0.011
71	H	–0.011
55	H	–0.009
62	C	–0.008
25	C	–0.007
142	H	–0.007
144	H	–0.006
42	C	–0.005
72	C	–0.005
114	H	–0.005
123	H	–0.005
125	H	–0.005
16	C	–0.004
17	C	–0.004
63	H	–0.004
86	C	–0.004
109	H	–0.003
51	H	–0.002
99	C	–0.002
43	H	–0.001
50	C	–0.001
64	C	–0.001
66	C	–0.001
87	C	–0.001
100	C	–0.001
104	C	–0.001
107	H	–0.001
121	C	–0.001
122	C	–0.001
134	C	–0.001
135	C	–0.001
28	C	0.000
44	C	0.000
47	C	0.000
53	C	0.000

91	C	0.000
95	C	0.000
113	C	0.000
120	H	0.000
126	C	0.000
130	C	0.000
131	H	0.000
139	C	0.000
143	C	0.000
31	C	0.001
35	C	0.001
40	C	0.001
69	C	0.001
108	C	0.001
111	H	0.001
59	C	0.002
129	H	0.002
140	H	0.002
37	C	0.005
119	C	0.005
36	H	0.006
56	C	0.006
65	H	0.006
101	H	0.006
138	H	0.006
39	C	0.007
57	C	0.007
146	H	0.007
2	O	0.008
41	H	0.008
105	H	0.008
127	H	0.008
133	H	0.008
136	H	0.008
103	H	0.010
45	H	0.011
60	H	0.011
90	H	0.012
38	H	0.013
68	H	0.013
76	C	0.013
115	C	0.013
118	C	0.013
84	C	0.014
96	H	0.014
3	O	0.015
48	H	0.015
80	C	0.015
124	H	0.015
137	H	0.015
147	N	0.015
24	C	0.016

30	H	0.016
33	H	0.016
46	H	0.016
81	C	0.016
92	H	0.016
98	H	0.016
128	H	0.016
132	H	0.016
141	H	0.016
145	H	0.016
148	H	0.016
9	C	0.017
54	H	0.017
58	H	0.017
85	H	0.017
7	N	0.019
52	H	0.019
116	C	0.020
117	H	0.020
70	H	0.021
32	H	0.022
93	H	0.022
97	H	0.022
106	H	0.022
89	H	0.023
102	H	0.023
67	H	0.024
82	H	0.024
149	H	0.024
75	C	0.026
110	H	0.026
18	C	0.027
22	C	0.027
14	C	0.028
10	C	0.029
27	H	0.033
83	C	0.034
74	H	0.037
8	N	0.040
12	C	0.040
20	C	0.040
15	H	0.045
19	H	0.045
23	H	0.051
11	H	0.052
21	H	0.054
13	H	0.055

1•4b····Zn^{II}

Number	Atom	VDD
1	Zn	–0.623
6	N	–0.177

4	N	–0.161
5	N	–0.159
73	C	–0.070
68	H	–0.068
74	H	–0.059
85	H	–0.040
20	H	–0.034
91	H	–0.024
14	C	–0.020
107	C	–0.016
43	H	–0.015
72	C	–0.013
13	C	–0.012
23	H	–0.012
55	C	–0.011
62	H	–0.010
53	C	–0.009
28	C	–0.008
29	C	–0.008
128	H	–0.008
30	H	–0.006
54	H	–0.006
126	H	–0.006
19	C	–0.004
81	C	–0.004
102	H	–0.004
109	H	–0.004
139	H	–0.003
51	C	–0.002
94	C	–0.002
141	H	–0.002
31	C	–0.001
44	C	–0.001
60	C	–0.001
66	C	–0.001
82	C	–0.001
95	C	–0.001
103	C	–0.001
116	C	–0.001
122	H	–0.001
129	C	–0.001
138	C	–0.001
22	C	0.000
25	C	0.000
38	C	0.000
41	C	0.000
47	C	0.000
63	C	0.000
86	C	0.000
90	C	0.000
100	H	0.000
104	H	0.000

117	C	0.000
121	C	0.000
125	C	0.000
130	C	0.000
134	C	0.000
137	H	0.000
49	H	0.001
99	C	0.001
108	C	0.002
115	H	0.002
120	H	0.002
131	H	0.002
3	O	0.003
36	C	0.003
45	H	0.003
56	C	0.003
32	H	0.004
39	H	0.005
98	H	0.005
33	C	0.006
50	C	0.006
52	H	0.006
34	C	0.007
42	H	0.007
58	C	0.007
114	C	0.007
133	H	0.007
135	H	0.007
106	H	0.008
7	N	0.009
18	C	0.009
118	H	0.009
124	H	0.009
57	H	0.010
96	H	0.010
148	N	0.010
2	O	0.011
37	H	0.011
83	H	0.011
9	C	0.012
75	C	0.012
110	C	0.012
27	H	0.013
76	C	0.013
93	H	0.013
113	C	0.013
79	C	0.014
127	H	0.014
24	H	0.015
64	H	0.015
71	C	0.015
87	H	0.015

89	H	0.015
119	H	0.015
123	H	0.015
140	H	0.015
35	H	0.016
59	H	0.016
132	H	0.016
142	N	0.016
157	N	0.016
67	H	0.017
80	H	0.017
136	H	0.017
149	H	0.017
15	C	0.018
40	H	0.018
46	H	0.018
48	H	0.018
145	N	0.018
151	N	0.018
10	C	0.019
12	C	0.020
26	H	0.020
92	H	0.020
153	H	0.020
154	N	0.020
158	H	0.020
17	C	0.021
65	H	0.021
88	H	0.021
112	H	0.021
77	H	0.022
97	H	0.022
105	H	0.022
84	H	0.023
111	C	0.023
143	H	0.023
101	H	0.025
61	H	0.026
69	C	0.026
21	H	0.029
78	C	0.030
16	C	0.031
70	H	0.032
11	C	0.033
159	H	0.033
147	H	0.035
150	H	0.035
144	H	0.036
146	H	0.036
152	H	0.036
155	H	0.036
156	H	0.037

8	N	0.040

1•4c····Zn^{II}

Number	Atom	VDD
1	Zn	–0.623
6	N	–0.176
5	N	–0.158
4	N	–0.156
73	C	–0.070
74	H	–0.070
68	H	–0.066
85	H	–0.049
14	C	–0.023
20	H	–0.022
91	H	–0.021
23	H	–0.020
107	C	–0.016
43	H	–0.015
13	C	–0.013
55	C	–0.012
62	H	–0.012
53	C	–0.011
72	C	–0.010
28	C	–0.009
54	H	–0.008
128	H	–0.008
126	H	–0.007
109	H	–0.006
19	C	–0.005
29	C	–0.005
81	C	–0.004
30	H	–0.003
102	H	–0.003
139	H	–0.003
51	C	–0.002
60	C	–0.002
94	C	–0.002
141	H	–0.002
44	C	–0.001
66	C	–0.001
82	C	–0.001
95	C	–0.001
103	C	–0.001
116	C	–0.001
129	C	–0.001
138	C	–0.001
22	C	0.000
25	C	0.000
31	C	0.000
38	C	0.000
41	C	0.000
47	C	0.000

63	C	0.000
90	C	0.000
104	H	0.000
117	C	0.000
121	C	0.000
122	H	0.000
125	C	0.000
130	C	0.000
134	C	0.000
36	C	0.001
49	H	0.001
86	C	0.001
99	C	0.001
100	H	0.001
120	H	0.001
137	H	0.001
45	H	0.002
108	C	0.002
131	H	0.002
3	O	0.003
56	C	0.003
115	H	0.003
98	H	0.005
32	H	0.006
34	C	0.006
52	H	0.006
27	H	0.007
33	C	0.007
50	C	0.007
58	C	0.007
114	C	0.007
133	H	0.007
135	H	0.007
18	C	0.008
37	H	0.008
39	H	0.008
42	H	0.008
106	H	0.008
7	N	0.009
118	H	0.009
124	H	0.009
83	H	0.010
96	H	0.010
2	O	0.011
57	H	0.011
110	C	0.011
148	N	0.011
9	C	0.012
127	H	0.013
75	C	0.014
76	C	0.014
89	H	0.014

93	H	0.014
113	C	0.014
24	H	0.015
35	H	0.015
71	C	0.015
79	C	0.015
119	H	0.015
123	H	0.015
140	H	0.015
142	N	0.015
59	H	0.016
64	H	0.016
87	H	0.016
132	H	0.016
136	H	0.016
149	H	0.016
157	N	0.016
15	C	0.017
40	H	0.017
46	H	0.017
48	H	0.017
80	H	0.018
145	N	0.018
151	N	0.018
10	C	0.019
12	C	0.020
67	H	0.020
153	H	0.020
154	N	0.020
17	C	0.021
26	H	0.021
65	H	0.021
88	H	0.021
92	H	0.021
112	H	0.021
158	H	0.021
105	H	0.022
111	C	0.022
77	H	0.023
97	H	0.023
143	H	0.023
84	H	0.025
101	H	0.025
61	H	0.026
69	C	0.027
16	C	0.031
21	H	0.031
70	H	0.031
11	C	0.033
78	C	0.033
150	H	0.034
159	H	0.034

147	H	0.035
144	H	0.036
146	H	0.036
152	H	0.036
155	H	0.037
156	H	0.037
8	N	0.040

1•4d···Zn^{II}

Number	Atom	VDD
1	Zn	–0.636
5	N	–0.155
4	N	–0.151
6	N	–0.151
73	C	–0.122
24	H	–0.091
74	H	–0.056
83	H	–0.044
67	H	–0.039
85	H	–0.028
107	C	–0.016
14	C	–0.015
43	H	–0.015
13	C	–0.014
28	C	–0.010
65	H	–0.010
20	H	–0.007
19	C	–0.006
118	H	–0.006
120	H	–0.006
137	H	–0.006
139	H	–0.006
55	C	–0.005
81	C	–0.004
82	C	–0.004
36	C	–0.003
56	C	–0.003
66	C	–0.003
94	C	–0.003
103	C	–0.002
109	H	–0.002
29	C	–0.001
44	C	–0.001
58	C	–0.001
86	C	–0.001
116	C	–0.001
117	C	–0.001
129	C	–0.001
130	C	–0.001
145	N	–0.001
154	N	–0.001
3	O	0.000

22	C	0.000
25	C	0.000
26	H	0.000
38	C	0.000
47	C	0.000
53	C	0.000
60	C	0.000
99	C	0.000
121	C	0.000
125	C	0.000
134	C	0.000
138	C	0.000
142	N	0.000
41	C	0.001
51	C	0.001
63	C	0.001
90	C	0.001
106	H	0.001
124	H	0.001
126	H	0.001
148	N	0.001
157	N	0.001
9	C	0.002
18	C	0.002
37	H	0.002
49	H	0.002
95	C	0.002
96	H	0.002
108	C	0.002
115	H	0.002
151	N	0.002
45	H	0.003
91	H	0.003
30	H	0.004
50	C	0.004
57	H	0.004
89	H	0.004
141	H	0.004
34	C	0.005
31	C	0.006
135	H	0.006
59	H	0.007
71	C	0.007
98	H	0.007
100	H	0.007
104	H	0.007
131	H	0.007
133	H	0.007
54	H	0.008
114	C	0.008
122	H	0.008
128	H	0.008

42	H	0.009
33	C	0.010
52	H	0.010
102	H	0.010
39	H	0.011
72	C	0.011
32	H	0.012
35	H	0.012
2	O	0.013
110	C	0.013
113	C	0.013
15	C	0.014
119	H	0.014
12	C	0.015
17	C	0.015
64	H	0.015
76	C	0.015
123	H	0.015
127	H	0.015
132	H	0.015
136	H	0.015
140	H	0.015
10	C	0.016
23	H	0.016
62	H	0.016
40	H	0.017
46	H	0.017
48	H	0.017
79	C	0.017
150	O	0.017
87	H	0.018
152	O	0.018
61	H	0.019
11	C	0.020
93	H	0.020
16	C	0.021
112	H	0.021
7	N	0.022
27	H	0.022
80	H	0.023
88	H	0.023
111	C	0.023
68	H	0.024
105	H	0.024
92	H	0.025
69	C	0.027
101	H	0.027
77	H	0.028
21	H	0.029
70	H	0.029
75	C	0.029
97	H	0.029

84	H	0.033
147	O	0.036
158	O	0.036
144	O	0.037
155	O	0.037
143	O	0.039
8	N	0.040
159	O	0.040
156	O	0.043
146	O	0.045
149	O	0.045
153	O	0.045
78	C	0.051

2•4a···Zn^{II}

Number	Atom	VDD
164	Zn	–0.602
72	N	–0.175
71	N	–0.161
4	N	–0.160
3	N	–0.158
69	H	–0.048
22	H	–0.039
82	C	–0.039
89	C	–0.028
26	H	–0.025
90	H	–0.023
117	C	–0.018
112	H	–0.017
114	H	–0.014
30	C	–0.011
57	C	–0.011
67	H	–0.011
103	H	–0.011
77	C	–0.010
147	H	–0.008
38	C	–0.007
50	H	–0.007
149	H	–0.007
21	C	–0.006
42	H	–0.006
48	H	–0.005
68	C	–0.005
97	H	–0.005
128	H	–0.005
130	H	–0.005
12	C	–0.004
39	H	–0.004
13	C	–0.003
58	C	–0.003
119	H	–0.003
91	C	–0.002

104	C	–0.002
36	C	–0.001
43	C	–0.001
46	C	–0.001
96	C	–0.001
125	H	–0.001
126	C	–0.001
127	C	–0.001
139	C	–0.001
140	C	–0.001
24	C	0.000
27	C	0.000
31	C	0.000
40	C	0.000
49	C	0.000
62	C	0.000
65	C	0.000
83	C	0.000
92	C	0.000
105	C	0.000
109	C	0.000
113	C	0.000
118	C	0.000
131	C	0.000
132	H	0.000
135	C	0.000
144	C	0.000
148	C	0.000
55	C	0.001
76	C	0.001
100	C	0.001
138	H	0.001
59	H	0.002
101	H	0.002
60	C	0.003
145	H	0.003
37	H	0.004
53	C	0.004
124	C	0.004
88	C	0.005
160	H	0.005
33	C	0.006
35	C	0.006
52	C	0.006
56	H	0.006
143	H	0.006
151	H	0.006
158	N	0.006
81	C	0.007
95	H	0.007
141	H	0.007
32	H	0.008

84	H	0.008
99	H	0.008
134	H	0.008
136	H	0.008
61	H	0.009
110	H	0.009
1	O	0.010
2	O	0.010
64	H	0.010
93	H	0.011
108	H	0.011
116	H	0.011
86	C	0.012
106	H	0.012
120	C	0.012
123	C	0.012
5	C	0.013
45	H	0.013
54	H	0.013
25	H	0.014
41	H	0.014
85	C	0.014
129	H	0.014
142	H	0.014
146	H	0.014
150	H	0.014
163	H	0.014
20	C	0.015
34	H	0.015
44	H	0.015
133	H	0.015
137	H	0.015
152	N	0.015
161	N	0.015
29	H	0.016
47	H	0.016
51	H	0.016
87	H	0.018
111	H	0.018
121	C	0.018
28	H	0.019
66	H	0.019
115	H	0.019
78	C	0.020
80	C	0.020
94	H	0.020
98	H	0.020
107	H	0.020
122	H	0.020
153	H	0.021
155	N	0.021
63	H	0.022

102	H	0.022
10	C	0.024
162	H	0.024
6	C	0.025
14	C	0.025
18	C	0.025
75	C	0.025
73	N	0.026
159	H	0.027
79	C	0.028
156	H	0.029
154	H	0.031
157	H	0.031
70	H	0.033
8	C	0.034
16	C	0.035
23	H	0.035
74	N	0.036
11	H	0.040
15	H	0.040
7	H	0.047
19	H	0.047
17	H	0.049
9	H	0.050

2•4b····Zn^{II}

Number	Atom	VDD
168	Zn	–0.608
66	N	–0.166
3	N	–0.165
4	N	–0.164
65	N	–0.149
16	H	–0.058
63	H	–0.055
80	C	–0.036
81	C	–0.024
82	H	–0.024
115	C	–0.016
95	H	–0.015
9	C	–0.013
10	C	–0.011
61	H	–0.011
101	H	–0.011
20	H	–0.010
24	C	–0.009
44	H	–0.009
110	H	–0.009
51	C	–0.008
36	H	–0.007
126	H	–0.007
143	H	–0.006
112	H	–0.005

128	H	–0.005
141	H	–0.005
15	C	–0.004
32	C	–0.004
49	C	–0.003
62	C	–0.003
102	C	–0.003
117	H	–0.003
42	H	–0.002
52	C	–0.002
89	C	–0.002
21	C	–0.001
25	C	–0.001
30	C	–0.001
37	C	–0.001
40	C	–0.001
56	C	–0.001
72	C	–0.001
94	C	–0.001
107	C	–0.001
124	C	–0.001
125	C	–0.001
137	C	–0.001
146	C	–0.001
18	C	0.000
34	C	0.000
43	C	0.000
47	C	0.000
59	C	0.000
71	C	0.000
90	C	0.000
98	C	0.000
111	C	0.000
129	C	0.000
133	C	0.000
136	H	0.000
138	C	0.000
142	C	0.000
103	C	0.001
116	C	0.001
33	H	0.002
54	C	0.002
87	C	0.002
123	H	0.002
130	H	0.002
27	C	0.003
50	H	0.003
46	C	0.004
29	C	0.005
108	H	0.005
139	H	0.005
145	H	0.005

106	H	0.006
147	H	0.006
1	O	0.007
2	O	0.007
14	C	0.007
26	H	0.007
53	H	0.007
83	C	0.007
97	H	0.007
149	H	0.007
31	H	0.008
88	H	0.008
99	H	0.008
122	C	0.008
132	H	0.008
134	H	0.008
5	C	0.009
48	H	0.009
79	C	0.010
104	H	0.010
114	H	0.010
28	H	0.011
55	H	0.011
84	C	0.011
91	H	0.011
159	N	0.011
160	H	0.011
39	H	0.012
86	C	0.012
93	H	0.012
156	N	0.012
165	N	0.012
118	C	0.013
121	C	0.013
162	N	0.013
23	H	0.014
35	H	0.014
58	H	0.014
127	H	0.014
38	H	0.015
45	H	0.015
131	H	0.015
135	H	0.015
140	H	0.015
144	H	0.015
148	H	0.015
150	N	0.015
19	H	0.016
41	H	0.016
96	H	0.017
8	C	0.018
11	C	0.018

60	H	0.018
85	H	0.018
158	H	0.018
6	C	0.019
13	C	0.019
92	H	0.019
100	H	0.019
109	H	0.020
113	H	0.020
166	H	0.020
22	H	0.021
73	C	0.021
77	C	0.021
105	H	0.021
120	H	0.021
151	H	0.021
57	H	0.022
67	N	0.022
119	C	0.022
153	N	0.023
64	H	0.026
17	H	0.027
69	C	0.027
161	H	0.027
7	C	0.029
12	C	0.029
70	H	0.029
74	H	0.030
164	H	0.030
75	C	0.031
155	H	0.032
152	H	0.033
163	H	0.033
154	H	0.034
157	H	0.034
167	H	0.034
68	N	0.038
78	H	0.042
76	H	0.044

2•4c····Zn^{II}

Number	Atom	VDD
164	Zn	–0.597
71	N	–0.177
72	N	–0.177
3	N	–0.155
4	N	–0.154
69	H	–0.052
89	C	–0.039
82	C	–0.037
22	H	–0.035
90	H	–0.025

112	H	–0.019
77	C	–0.014
114	H	–0.014
117	C	–0.014
67	H	–0.009
97	H	–0.009
103	H	–0.009
161	N	–0.009
76	C	–0.008
128	H	–0.008
13	C	–0.007
147	H	–0.006
26	H	–0.005
73	N	–0.005
125	H	–0.005
12	C	–0.004
68	C	–0.004
119	H	–0.004
145	H	–0.004
21	C	–0.003
91	C	–0.003
104	C	–0.003
42	H	–0.002
48	H	–0.002
50	H	–0.002
81	C	–0.002
130	H	–0.002
138	H	–0.002
149	H	–0.002
158	N	–0.002
27	C	–0.001
43	C	–0.001
57	C	–0.001
58	C	–0.001
62	C	–0.001
83	C	–0.001
96	C	–0.001
109	C	–0.001
118	C	–0.001
124	C	–0.001
126	C	–0.001
139	C	–0.001
155	N	–0.001
38	C	0.000
40	C	0.000
46	C	0.000
49	C	0.000
65	C	0.000
100	C	0.000
105	C	0.000
113	C	0.000
127	C	0.000

131	C	0.000
135	C	0.000
140	C	0.000
144	C	0.000
148	C	0.000
24	C	0.001
92	C	0.001
152	N	0.001
30	C	0.002
132	H	0.002
143	H	0.002
162	O	0.002
31	C	0.003
39	H	0.003
55	C	0.004
59	H	0.005
88	C	0.005
84	H	0.007
99	H	0.007
134	H	0.007
136	H	0.007
151	H	0.007
60	C	0.008
101	H	0.008
141	H	0.008
93	H	0.009
32	H	0.010
80	C	0.010
95	H	0.010
110	H	0.010
36	C	0.011
53	C	0.011
64	H	0.011
86	C	0.011
108	H	0.011
116	H	0.011
120	C	0.011
123	C	0.011
159	O	0.011
56	H	0.012
106	H	0.012
25	H	0.013
37	H	0.013
52	C	0.013
85	C	0.013
29	H	0.014
33	C	0.014
45	H	0.014
61	H	0.014
129	H	0.014
146	H	0.014
150	H	0.014

5	C	0.015
35	C	0.015
78	C	0.015
133	H	0.015
137	H	0.015
142	H	0.015
20	C	0.016
44	H	0.016
47	H	0.016
75	C	0.016
41	H	0.017
54	H	0.017
79	C	0.017
121	C	0.017
122	H	0.017
34	H	0.018
51	H	0.018
87	H	0.018
111	H	0.019
98	H	0.020
107	H	0.020
115	H	0.020
28	H	0.021
66	H	0.021
102	H	0.021
163	O	0.021
94	H	0.022
2	O	0.023
10	C	0.023
1	O	0.024
14	C	0.024
154	O	0.024
6	C	0.025
18	C	0.025
63	H	0.025
74	N	0.025
160	O	0.025
23	H	0.030
157	O	0.031
16	C	0.033
8	C	0.035
70	H	0.035
156	O	0.036
153	O	0.039
15	H	0.040
11	H	0.042
19	H	0.045
17	H	0.046
7	H	0.048
9	H	0.050

2•4d···Zn^{II}

Number	Atom	VDD
168	Zn	–0.602
4	N	–0.173
3	N	–0.172
66	N	–0.159
65	N	–0.147
63	H	–0.070
16	H	–0.068
80	C	–0.045
9	C	–0.019
87	C	–0.019
115	C	–0.017
10	C	–0.016
101	H	–0.012
20	H	–0.011
88	H	–0.011
108	H	–0.011
61	H	–0.010
42	H	–0.008
95	H	–0.007
110	H	–0.007
128	H	–0.006
36	H	–0.005
126	H	–0.005
143	H	–0.005
49	C	–0.004
15	C	–0.003
24	C	–0.003
117	H	–0.003
5	C	–0.002
62	C	–0.002
102	C	–0.002
141	H	–0.002
162	N	–0.002
25	C	–0.001
37	C	–0.001
40	C	–0.001
56	C	–0.001
81	C	–0.001
89	C	–0.001
94	C	–0.001
111	C	–0.001
124	C	–0.001
125	C	–0.001
137	C	–0.001
142	C	–0.001
145	H	–0.001
153	N	–0.001
21	C	0.000
32	C	0.000
34	C	0.000

43	C	0.000
59	C	0.000
72	C	0.000
90	C	0.000
103	C	0.000
106	H	0.000
107	C	0.000
112	H	0.000
129	C	0.000
130	H	0.000
133	C	0.000
138	C	0.000
146	C	0.000
18	C	0.001
27	C	0.001
47	C	0.001
50	H	0.001
51	C	0.001
71	C	0.001
98	C	0.001
123	H	0.001
150	N	0.001
165	N	0.001
30	C	0.002
99	H	0.002
116	C	0.002
136	H	0.002
156	N	0.002
159	N	0.002
14	C	0.003
147	H	0.003
29	C	0.005
122	C	0.005
1	O	0.006
26	H	0.006
52	C	0.006
82	H	0.006
93	H	0.007
139	H	0.007
149	H	0.007
13	C	0.008
54	C	0.008
132	H	0.008
134	H	0.008
6	C	0.009
28	H	0.009
33	H	0.009
46	C	0.009
48	H	0.009
86	C	0.009
97	H	0.009
8	C	0.010

2	O	0.011
11	C	0.011
31	H	0.011
38	H	0.012
91	H	0.012
118	C	0.012
45	H	0.013
84	C	0.013
104	H	0.013
121	C	0.013
53	H	0.014
114	H	0.014
127	H	0.014
19	H	0.015
23	H	0.015
35	H	0.015
39	H	0.015
55	H	0.015
79	C	0.015
83	C	0.015
131	H	0.015
135	H	0.015
144	H	0.015
41	H	0.016
58	H	0.016
140	H	0.016
148	H	0.016
44	H	0.017
109	H	0.017
113	H	0.017
60	H	0.018
85	H	0.018
96	H	0.018
7	C	0.019
92	H	0.019
100	H	0.019
105	H	0.019
161	O	0.019
22	H	0.020
57	H	0.020
120	H	0.020
73	C	0.021
119	C	0.021
12	C	0.022
17	H	0.023
77	C	0.023
64	H	0.024
69	C	0.025
70	H	0.027
166	O	0.027
67	N	0.029
74	H	0.030

152	O	0.030
75	C	0.032
154	O	0.033
157	O	0.037
164	O	0.037
68	N	0.038
151	O	0.038
163	O	0.038
167	O	0.040
155	O	0.042
76	H	0.044
78	H	0.046
160	O	0.046
158	O	0.047

3•4a····Zn^{II}

Number	Atom	VDD
172	Zn	–0.584
148	N	–0.162
150	N	–0.159
64	N	–0.151
65	N	–0.150
149	N	–0.149
10	H	–0.055
54	H	–0.045
52	H	–0.024
107	C	–0.020
95	C	–0.016
32	H	–0.011
42	C	–0.011
119	H	–0.009
77	H	–0.008
94	H	–0.008
18	C	–0.007
19	C	–0.007
1	C	–0.006
20	H	–0.006
29	H	–0.006
78	H	–0.006
141	H	–0.006
13	H	–0.005
53	C	–0.005
87	H	–0.005
115	H	–0.005
48	H	–0.004
63	C	–0.004
131	H	–0.004
9	C	–0.003
21	C	–0.003
43	C	–0.003
93	C	–0.003
156	H	–0.003

120	H	–0.002
133	H	–0.002
28	C	–0.001
31	C	–0.001
79	C	–0.001
80	C	–0.001
84	C	–0.001
98	C	–0.001
109	H	–0.001
114	C	–0.001
116	C	–0.001
117	C	–0.001
125	C	–0.001
127	H	–0.001
130	C	–0.001
169	N	–0.001
15	C	0.000
34	C	0.000
40	C	0.000
50	C	0.000
67	C	0.000
71	C	0.000
72	H	0.000
75	C	0.000
85	C	0.000
102	C	0.000
108	C	0.000
121	C	0.000
126	C	0.000
134	C	0.000
138	C	0.000
142	C	0.000
144	H	0.000
153	C	0.000
12	C	0.001
44	H	0.001
47	C	0.001
70	O	0.001
106	H	0.001
22	H	0.002
103	C	0.002
137	H	0.002
140	H	0.002
41	H	0.003
122	H	0.003
26	C	0.004
97	C	0.004
23	C	0.005
24	C	0.005
38	C	0.005
45	C	0.005
105	C	0.005

81	H	0.006
82	H	0.006
88	H	0.006
128	H	0.006
136	H	0.006
155	H	0.007
164	H	0.007
37	C	0.008
66	O	0.008
123	H	0.008
163	N	0.008
73	H	0.009
143	H	0.009
27	H	0.010
69	H	0.010
92	C	0.010
36	H	0.011
113	C	0.011
8	C	0.012
16	H	0.012
39	H	0.012
46	H	0.012
89	C	0.012
110	C	0.012
157	N	0.012
159	H	0.012
25	H	0.013
56	C	0.013
76	H	0.013
118	H	0.013
33	H	0.014
68	H	0.014
74	H	0.014
83	H	0.014
86	H	0.014
124	H	0.014
132	H	0.014
145	H	0.014
154	H	0.014
35	H	0.015
129	H	0.015
135	H	0.015
139	H	0.015
30	H	0.016
90	C	0.016
14	H	0.017
111	C	0.017
147	N	0.017
160	N	0.017
91	H	0.018
112	H	0.018
49	H	0.019

17	H	0.020
51	H	0.020
162	H	0.020
165	H	0.020
101	C	0.021
151	N	0.022
158	H	0.022
166	N	0.022
2	C	0.023
61	C	0.023
99	C	0.023
100	C	0.023
6	C	0.024
170	H	0.024
104	C	0.025
57	C	0.026
96	C	0.026
171	H	0.026
11	H	0.029
146	N	0.031
167	H	0.031
152	N	0.033
168	H	0.033
4	C	0.034
161	H	0.034
55	H	0.035
59	C	0.035
3	H	0.040
62	H	0.040
7	H	0.046
58	H	0.047
5	H	0.049
60	H	0.050

3•4b····Zn^{II}

Number	Atom	VDD
174	Zn	–0.600
58	N	–0.158
147	N	–0.158
59	N	–0.151
148	N	–0.139
149	N	–0.137
7	H	–0.063
51	H	–0.054
57	C	–0.016
106	C	–0.016
89	C	–0.015
1	C	–0.012
29	H	–0.011
114	H	–0.011
16	C	–0.009
26	H	–0.008

39	C	–0.008
10	H	–0.007
15	C	–0.007
17	H	–0.007
71	H	–0.007
72	H	–0.007
118	H	–0.007
88	H	–0.006
119	H	–0.006
140	H	–0.006
81	H	–0.005
101	C	–0.005
18	C	–0.004
40	C	–0.003
6	C	–0.002
37	C	–0.002
50	C	–0.002
113	C	–0.002
132	H	–0.002
12	C	–0.001
25	C	–0.001
44	C	–0.001
73	C	–0.001
74	C	–0.001
78	C	–0.001
115	C	–0.001
124	C	–0.001
129	C	–0.001
130	H	–0.001
9	C	0.000
28	C	0.000
31	C	0.000
35	C	0.000
49	H	0.000
61	C	0.000
65	C	0.000
66	H	0.000
69	C	0.000
79	C	0.000
87	C	0.000
116	C	0.000
120	C	0.000
125	C	0.000
133	C	0.000
137	C	0.000
141	C	0.000
152	C	0.000
155	H	0.000
42	C	0.001
47	C	0.001
64	O	0.001
92	C	0.001

121	H	0.001
143	H	0.001
105	H	0.002
126	H	0.002
136	H	0.002
19	H	0.003
34	C	0.004
38	H	0.004
41	H	0.004
53	C	0.004
60	O	0.004
108	H	0.004
139	H	0.004
20	C	0.005
23	C	0.005
82	H	0.005
127	H	0.005
135	H	0.005
21	C	0.006
75	H	0.006
76	H	0.006
93	C	0.006
100	C	0.006
104	C	0.007
107	C	0.007
36	H	0.008
112	C	0.008
5	C	0.009
43	H	0.009
67	H	0.009
142	H	0.009
162	N	0.009
165	N	0.009
33	H	0.011
45	H	0.011
86	C	0.011
122	H	0.011
154	H	0.011
24	H	0.012
63	H	0.012
70	H	0.012
171	N	0.012
83	C	0.013
117	H	0.013
123	H	0.013
153	H	0.013
168	N	0.013
13	H	0.014
22	H	0.014
62	H	0.014
68	H	0.014
144	H	0.014

156	N	0.014
30	H	0.015
32	H	0.015
77	H	0.015
80	H	0.015
109	C	0.015
131	H	0.015
138	H	0.015
150	N	0.015
159	N	0.015
27	H	0.016
54	C	0.016
56	C	0.016
128	H	0.016
134	H	0.016
11	H	0.017
48	H	0.017
164	H	0.017
2	C	0.018
14	H	0.019
84	C	0.019
85	H	0.019
111	H	0.019
4	C	0.020
52	H	0.020
110	C	0.020
146	N	0.020
167	H	0.020
173	H	0.020
94	C	0.021
98	C	0.021
158	H	0.021
46	H	0.023
102	C	0.024
90	C	0.026
91	H	0.027
8	H	0.028
3	C	0.029
55	C	0.029
96	C	0.029
166	H	0.029
170	H	0.029
95	H	0.030
151	N	0.031
161	H	0.031
160	H	0.033
163	H	0.033
169	H	0.033
172	H	0.033
157	H	0.034
145	N	0.035
103	H	0.036

99	H	0.040
97	H	0.043

3•4c····Zn^{II}

Number	Atom	VDD
172	Zn	–0.588
148	N	–0.167
150	N	–0.163
149	N	–0.157
65	N	–0.149
64	N	–0.145
10	H	–0.046
52	H	–0.034
54	H	–0.029
95	C	–0.015
98	C	–0.015
107	C	–0.015
77	H	–0.010
13	H	–0.009
94	H	–0.009
131	H	–0.009
87	H	–0.007
109	H	–0.007
141	H	–0.007
42	C	–0.006
53	C	–0.006
120	H	–0.006
1	C	–0.005
29	H	–0.005
32	H	–0.005
48	H	–0.005
78	H	–0.005
122	H	–0.005
93	C	–0.004
102	C	–0.004
9	C	–0.003
63	C	–0.003
97	C	–0.002
108	C	–0.002
127	H	–0.002
133	H	–0.002
144	H	–0.002
166	N	–0.002
24	C	–0.001
28	C	–0.001
50	C	–0.001
79	C	–0.001
80	C	–0.001
84	C	–0.001
103	C	–0.001
106	H	–0.001
116	C	–0.001

125	C	–0.001
153	C	–0.001
31	C	0.000
34	C	0.000
43	C	0.000
67	C	0.000
71	C	0.000
75	C	0.000
85	C	0.000
117	C	0.000
121	C	0.000
126	C	0.000
130	C	0.000
134	C	0.000
138	C	0.000
142	C	0.000
163	N	0.000
12	C	0.001
15	C	0.001
16	H	0.001
40	C	0.001
47	C	0.001
72	H	0.001
105	C	0.001
115	H	0.001
147	N	0.001
18	C	0.002
27	H	0.002
119	H	0.002
140	H	0.002
169	N	0.002
26	C	0.003
44	H	0.003
137	H	0.003
157	N	0.003
41	H	0.004
114	C	0.004
160	N	0.004
88	H	0.005
123	H	0.005
156	H	0.005
170	O	0.005
81	H	0.006
82	H	0.006
151	N	0.006
155	H	0.006
69	H	0.008
73	H	0.008
101	C	0.008
128	H	0.008
136	H	0.008
143	H	0.008

36	H	0.009
45	C	0.009
92	C	0.009
99	C	0.009
38	C	0.010
89	C	0.010
96	C	0.010
110	C	0.011
165	O	0.011
21	C	0.012
37	C	0.012
100	C	0.012
113	C	0.012
132	H	0.012
39	H	0.013
76	H	0.013
129	H	0.013
135	H	0.013
139	H	0.013
145	H	0.013
154	H	0.013
74	H	0.014
83	H	0.014
86	H	0.014
90	C	0.014
118	H	0.014
124	H	0.014
8	C	0.015
46	H	0.015
56	C	0.015
66	O	0.015
19	C	0.016
30	H	0.016
33	H	0.016
91	H	0.016
35	H	0.017
111	C	0.017
112	H	0.017
14	H	0.018
23	C	0.018
17	H	0.019
49	H	0.019
162	O	0.019
20	H	0.020
68	H	0.020
51	H	0.021
70	O	0.022
104	C	0.022
22	H	0.023
2	C	0.024
61	C	0.024
146	N	0.024

158	O	0.024
6	C	0.026
25	H	0.026
57	C	0.026
161	O	0.027
168	O	0.029
152	N	0.030
167	O	0.031
171	O	0.031
11	H	0.033
4	C	0.035
55	H	0.035
59	C	0.036
164	O	0.037
3	H	0.040
62	H	0.040
159	O	0.044
7	H	0.046
58	H	0.048
5	H	0.049
60	H	0.050

3•4d····Zn^{II}

Number	Atom	VDD
174	Zn	–0.604
147	N	–0.155
59	N	–0.153
149	N	–0.146
58	N	–0.145
148	N	–0.138
7	H	–0.053
51	H	–0.049
49	H	–0.021
1	C	–0.016
106	C	–0.016
89	C	–0.015
10	H	–0.013
57	C	–0.011
29	H	–0.010
26	H	–0.009
39	C	–0.009
118	H	–0.009
5	C	–0.008
71	H	–0.007
72	H	–0.007
155	H	–0.007
15	C	–0.006
88	H	–0.006
130	H	–0.006
140	H	–0.006
45	H	–0.005
81	H	–0.005

126	H	–0.005
16	C	–0.004
37	C	–0.004
6	C	–0.003
18	C	–0.003
50	C	–0.003
114	H	–0.002
159	N	–0.002
25	C	–0.001
40	C	–0.001
47	C	–0.001
53	C	–0.001
73	C	–0.001
74	C	–0.001
78	C	–0.001
115	C	–0.001
116	C	–0.001
124	C	–0.001
129	C	–0.001
168	N	–0.001
9	C	0.000
12	C	0.000
28	C	0.000
31	C	0.000
38	H	0.000
44	C	0.000
61	C	0.000
65	C	0.000
69	C	0.000
79	C	0.000
87	C	0.000
108	H	0.000
120	C	0.000
125	C	0.000
133	C	0.000
137	C	0.000
141	C	0.000
143	H	0.000
152	C	0.000
171	N	0.000
23	C	0.001
66	H	0.001
113	C	0.001
132	H	0.001
156	N	0.001
17	H	0.002
21	C	0.002
64	O	0.002
162	N	0.002
165	N	0.002
35	C	0.003
41	H	0.003

101	C	0.003
105	H	0.003
107	C	0.003
119	H	0.003
92	C	0.004
139	H	0.004
20	C	0.005
82	H	0.005
136	H	0.005
42	C	0.006
75	H	0.006
76	H	0.006
127	H	0.006
100	C	0.007
104	C	0.007
121	H	0.007
135	H	0.007
154	H	0.007
19	H	0.008
93	C	0.008
122	H	0.008
34	C	0.009
67	H	0.009
22	H	0.010
24	H	0.010
36	H	0.010
63	H	0.010
112	C	0.010
142	H	0.010
2	C	0.011
33	H	0.011
60	O	0.011
86	C	0.011
70	H	0.012
109	C	0.012
4	C	0.013
43	H	0.013
83	C	0.013
56	C	0.014
68	H	0.014
144	H	0.014
27	H	0.015
30	H	0.015
32	H	0.015
54	C	0.015
77	H	0.015
80	H	0.015
117	H	0.015
123	H	0.015
131	H	0.015
138	H	0.015
13	H	0.016

62	H	0.016
128	H	0.016
134	H	0.016
153	H	0.016
163	O	0.016
48	H	0.017
172	O	0.017
3	C	0.018
11	H	0.019
55	C	0.019
110	C	0.019
111	H	0.019
14	H	0.020
46	H	0.020
84	C	0.020
85	H	0.020
150	N	0.020
167	O	0.022
8	H	0.023
146	N	0.023
94	C	0.024
98	C	0.024
169	O	0.024
90	C	0.027
102	C	0.027
52	H	0.029
91	H	0.029
96	C	0.031
161	O	0.032
95	H	0.034
158	O	0.035
151	N	0.036
157	O	0.036
166	O	0.036
145	N	0.037
103	H	0.041
170	O	0.041
160	O	0.042
99	H	0.043
97	H	0.045
173	O	0.045
164	O	0.046

5•6$_{N,ac}$···Na^+

Number	Atom	VDD
3	O	–0.113
44	Na	–0.100
11	N	–0.061
21	N	–0.060
15	C	–0.046
32	H	–0.038
39	H	–0.011

26	H	–0.010
10	C	–0.007
22	C	–0.007
24	H	–0.006
38	H	–0.006
14	C	–0.005
16	C	–0.004
12	C	–0.003
20	C	–0.003
13	C	–0.002
23	C	–0.002
1	C	–0.001
31	H	0.000
41	H	0.000
7	H	0.006
29	H	0.007
43	H	0.008
5	H	0.009
17	C	0.010
19	C	0.010
28	H	0.013
37	H	0.014
4	N	0.017
18	C	0.019
2	C	0.022
9	H	0.022
35	H	0.022
33	H	0.023
6	H	0.027
27	H	0.028
36	H	0.028
34	H	0.029
42	H	0.032
30	H	0.033
25	H	0.035
40	H	0.035
8	H	0.037

5•6$_{N,c}$···Na^+

Number	Atom	VDD
4	O	–0.117
1	Na	–0.088
23	H	–0.035
14	C	–0.032
13	C	–0.024
15	C	–0.021
40	H	–0.016
7	N	–0.004
12	C	–0.004
8	C	–0.002
16	C	–0.002
2	C	–0.001

19	C	–0.001
9	C	0.000
10	C	0.000
17	N	0.000
18	C	0.000
6	C	0.001
11	C	0.003
34	H	0.006
32	H	0.008
25	H	0.010
29	H	0.010
31	H	0.012
36	H	0.012
27	H	0.013
38	H	0.013
33	H	0.014
20	H	0.015
37	H	0.015
28	H	0.016
5	N	0.018
30	H	0.018
3	C	0.019
24	H	0.022
26	H	0.022
39	H	0.022
21	H	0.023
35	H	0.023
22	H	0.031

5•6$_N$···Na^+

Number	Atom	VDD
5	O	–0.113
155	Na	–0.109
137	H	–0.036
76	H	–0.030
135	H	–0.028
121	C	–0.027
19	C	–0.026
122	H	–0.025
10	C	–0.021
36	C	–0.019
17	C	–0.018
35	C	–0.016
38	C	–0.015
87	H	–0.015
33	C	–0.014
15	C	–0.010
30	C	–0.009
41	H	–0.009
69	C	–0.009
7	N	–0.008
31	C	–0.008

85	C	–0.008
21	H	–0.007
104	H	–0.007
120	C	–0.007
71	H	–0.006
106	H	–0.006
3	O	–0.005
12	C	–0.005
13	C	–0.005
59	H	–0.005
70	C	–0.005
2	O	–0.004
123	C	–0.004
6	N	–0.003
20	C	–0.003
86	C	–0.003
27	C	–0.002
44	H	–0.002
81	C	–0.002
134	C	–0.002
153	H	–0.002
40	C	–0.001
46	C	–0.001
50	H	–0.001
94	C	–0.001
98	H	–0.001
107	C	–0.001
117	H	–0.001
119	H	–0.001
129	C	–0.001
142	C	–0.001
143	C	–0.001
23	C	0.000
43	C	0.000
49	C	0.000
52	C	0.000
55	C	0.000
58	C	0.000
61	C	0.000
74	C	0.000
95	C	0.000
99	C	0.000
101	H	0.000
103	C	0.000
109	C	0.000
113	C	0.000
114	H	0.000
116	C	0.000
130	C	0.000
132	H	0.000
138	C	0.000
146	H	0.000

147	C	0.000
151	C	0.000
152	H	0.000
1	O	0.001
67	C	0.001
72	C	0.001
77	C	0.001
110	H	0.001
149	H	0.001
57	H	0.002
139	H	0.002
4	O	0.003
93	H	0.003
108	H	0.003
112	H	0.003
53	H	0.004
65	C	0.004
68	H	0.004
92	C	0.004
47	H	0.005
73	H	0.005
88	C	0.005
144	H	0.005
148	H	0.005
37	H	0.006
64	C	0.006
96	H	0.006
100	H	0.006
124	C	0.006
127	C	0.006
60	H	0.007
63	H	0.007
79	H	0.007
25	H	0.008
51	H	0.008
54	H	0.008
56	H	0.008
66	H	0.008
78	H	0.008
91	C	0.008
97	H	0.008
102	H	0.008
105	H	0.008
128	H	0.008
8	N	0.009
45	H	0.009
62	H	0.009
111	H	0.009
115	H	0.009
118	H	0.009
133	H	0.009
18	H	0.010

24	H	0.010
29	H	0.010
48	H	0.010
125	H	0.010
141	H	0.010
11	H	0.011
75	H	0.011
83	H	0.011
89	C	0.011
90	H	0.011
126	C	0.011
145	H	0.011
150	H	0.011
42	H	0.012
154	H	0.012
26	H	0.013
34	H	0.013
39	H	0.013
80	H	0.013
131	H	0.013
140	H	0.013
9	H	0.014
82	H	0.014
16	H	0.016
28	H	0.016
32	H	0.016
22	H	0.017
84	C	0.017
14	H	0.019
136	H	0.019

5•6$_{O,ac}$···Na^+

Number	Atom	VDD
3	O	–0.097
40	Na	–0.083
20	O	–0.066
11	O	–0.062
17	O	–0.062
14	O	–0.061
9	H	–0.008
13	C	–0.005
12	C	–0.004
15	C	–0.004
16	C	–0.004
18	C	–0.004
19	C	–0.004
21	C	–0.002
10	C	–0.001
37	H	–0.001
1	C	0.000
22	H	0.000
28	H	0.007

35	H	0.009
4	N	0.010
32	H	0.010
25	H	0.011
29	H	0.011
34	H	0.011
24	H	0.012
39	H	0.012
5	H	0.018
7	H	0.019
2	C	0.020
6	H	0.025
23	H	0.031
26	H	0.031
27	H	0.032
30	H	0.032
31	H	0.032
33	H	0.033
36	H	0.033
38	H	0.034
8	H	0.036

5•6$_{O,c}$···Na^+

Number	Atom	VDD
36	Na	–0.082
5	O	–0.068
11	O	–0.057
2	O	–0.056
8	O	–0.054
15	O	–0.046
14	C	–0.015
7	C	–0.009
3	C	–0.008
26	H	–0.008
9	C	–0.004
10	C	–0.004
12	C	–0.004
13	C	–0.004
4	C	–0.003
6	C	–0.003
1	C	–0.002
16	N	–0.002
20	H	–0.002
17	H	0.011
30	H	0.011
27	H	0.012
34	H	0.017
21	H	0.019
32	H	0.020
23	H	0.023
35	H	0.028
24	H	0.030

31	H	0.030
22	H	0.031
33	H	0.031
18	H	0.032
28	H	0.032
29	H	0.032
19	H	0.034
25	H	0.035

5•6$_{O}$···Na^{+}

Number	Atom	VDD
6	O	–0.104
1	Na	–0.090
2	O	–0.070
4	O	–0.059
3	O	–0.054
61	H	–0.027
122	C	–0.026
121	C	–0.024
141	H	–0.022
37	C	–0.019
39	H	–0.017
38	C	–0.013
147	H	–0.013
128	C	–0.012
86	C	–0.009
124	C	–0.009
114	H	–0.008
24	H	–0.007
48	C	–0.007
54	C	–0.006
118	H	–0.006
12	C	–0.005
13	H	–0.005
94	H	–0.005
45	C	–0.004
51	C	–0.004
125	C	–0.004
142	H	–0.004
154	H	–0.004
8	N	–0.003
21	C	–0.003
66	C	–0.003
81	H	–0.003
138	H	–0.003
7	N	–0.002
42	C	–0.002
55	H	–0.002
80	H	–0.002
103	H	–0.002
130	C	–0.002
143	C	–0.002

14	C	–0.001
57	C	–0.001
60	C	–0.001
74	C	–0.001
76	C	–0.001
95	C	–0.001
99	H	–0.001
108	C	–0.001
135	C	–0.001
139	C	–0.001
152	C	–0.001
22	C	0.000
25	C	0.000
29	C	0.000
63	C	0.000
67	C	0.000
72	C	0.000
79	C	0.000
83	C	0.000
93	C	0.000
96	C	0.000
100	C	0.000
101	H	0.000
104	C	0.000
109	C	0.000
113	C	0.000
117	C	0.000
123	H	0.000
127	C	0.000
131	C	0.000
144	C	0.000
145	H	0.000
148	C	0.000
75	H	0.001
19	C	0.002
50	H	0.002
69	C	0.002
71	C	0.002
88	H	0.002
105	H	0.002
133	H	0.002
15	C	0.003
43	H	0.003
73	H	0.003
85	H	0.003
87	C	0.003
115	H	0.003
17	C	0.004
27	H	0.004
35	C	0.004
40	C	0.004
68	H	0.004

98	H	0.004
106	H	0.004
110	H	0.004
120	H	0.004
149	H	0.004
5	O	0.005
20	H	0.005
26	H	0.005
77	H	0.005
111	H	0.005
153	H	0.005
23	H	0.006
58	H	0.006
70	H	0.006
92	C	0.006
129	H	0.006
9	N	0.007
16	H	0.007
31	H	0.007
36	H	0.007
41	H	0.007
64	H	0.007
89	C	0.007
28	H	0.008
30	H	0.008
33	C	0.008
84	H	0.008
102	H	0.008
112	H	0.008
116	H	0.008
119	H	0.008
150	H	0.008
18	H	0.009
62	H	0.009
78	H	0.009
97	H	0.009
107	H	0.009
137	H	0.009
82	H	0.010
90	C	0.010
126	H	0.010
134	H	0.010
47	H	0.011
91	H	0.011
155	H	0.011
32	C	0.012
65	H	0.012
132	H	0.012
136	H	0.012
151	H	0.012
10	H	0.014
34	H	0.014

11	C	0.015
146	H	0.015
52	H	0.016
140	H	0.016
44	H	0.021
59	H	0.024
53	H	0.025
46	H	0.027
49	H	0.027
56	H	0.030

5•6$_N$···K^+

Number	Atom	VDD
5	O	–0.093
155	K	–0.066
6	N	–0.052
7	N	–0.041
71	H	–0.041
10	C	–0.037
11	H	–0.032
39	H	–0.029
70	C	–0.025
93	H	–0.022
137	H	–0.019
38	C	–0.018
114	H	–0.018
92	C	–0.016
122	H	–0.015
25	H	–0.013
69	C	–0.012
24	H	–0.011
30	C	–0.010
76	H	–0.010
132	H	–0.010
85	C	–0.009
108	H	–0.009
19	C	–0.006
23	C	–0.006
59	H	–0.006
74	C	–0.006
120	C	–0.006
3	O	–0.005
12	C	–0.005
44	H	–0.005
121	C	–0.004
20	C	–0.003
27	C	–0.003
81	C	–0.003
104	H	–0.003
2	O	–0.002
50	H	–0.002
106	H	–0.002

117	H	–0.002
149	H	–0.002
60	H	–0.001
77	C	–0.001
94	C	–0.001
99	C	–0.001
142	C	–0.001
152	H	–0.001
40	C	0.000
43	C	0.000
46	C	0.000
49	C	0.000
52	C	0.000
55	C	0.000
58	C	0.000
61	C	0.000
95	C	0.000
98	H	0.000
103	C	0.000
107	C	0.000
109	C	0.000
113	C	0.000
116	C	0.000
129	C	0.000
130	C	0.000
138	C	0.000
143	C	0.000
147	C	0.000
151	C	0.000
91	C	0.001
101	H	0.001
112	H	0.001
134	C	0.001
148	H	0.001
41	H	0.002
47	H	0.002
72	C	0.002
123	C	0.002
144	H	0.002
153	H	0.002
36	C	0.003
79	H	0.003
146	H	0.003
29	H	0.004
31	C	0.004
86	C	0.004
87	H	0.004
128	H	0.004
135	H	0.004
45	H	0.005
54	H	0.005
56	H	0.005

63	H	0.005
67	C	0.005
73	H	0.005
78	H	0.005
89	C	0.005
96	H	0.005
100	H	0.005
127	C	0.005
133	H	0.005
136	H	0.005
139	H	0.005
37	H	0.006
53	H	0.006
1	O	0.007
17	C	0.007
57	H	0.007
83	H	0.007
84	C	0.007
131	H	0.007
140	H	0.007
141	H	0.007
4	O	0.008
13	C	0.008
21	H	0.008
35	C	0.008
51	H	0.008
88	C	0.008
111	H	0.008
118	H	0.008
124	C	0.008
126	C	0.008
145	H	0.008
32	H	0.009
33	C	0.009
48	H	0.009
90	H	0.009
110	H	0.009
115	H	0.009
150	H	0.009
154	H	0.009
8	N	0.010
9	H	0.010
64	C	0.010
97	H	0.010
102	H	0.010
119	H	0.010
125	H	0.010
42	H	0.011
65	C	0.011
68	H	0.011
105	H	0.011
62	H	0.012

15	C	0.013
34	H	0.013
18	H	0.016
66	H	0.016
14	H	0.017
28	H	0.019
75	H	0.019
82	H	0.019
16	H	0.021
22	H	0.021
80	H	0.021
26	H	0.023

5•6$_N$···Li^+

Number	Atom	VDD
155	Li	–0.174
5	O	–0.133
7	N	–0.077
10	C	–0.036
67	C	–0.034
12	C	–0.032
69	C	–0.027
87	H	–0.027
68	H	–0.020
122	H	–0.019
101	H	–0.017
59	H	–0.015
85	C	–0.012
86	C	–0.012
137	H	–0.011
11	H	–0.009
74	C	–0.008
120	C	–0.008
135	H	–0.008
44	H	–0.006
2	O	–0.005
3	O	–0.005
77	C	–0.005
81	C	–0.005
106	H	–0.005
30	C	–0.003
114	H	–0.003
21	H	–0.002
38	C	–0.002
45	H	–0.002
78	H	–0.002
121	C	–0.002
20	C	–0.001
39	H	–0.001
94	C	–0.001
107	C	–0.001
116	C	–0.001

117	H	–0.001
132	H	–0.001
142	C	–0.001
6	N	0.000
13	C	0.000
22	H	0.000
23	C	0.000
27	C	0.000
31	C	0.000
36	C	0.000
40	C	0.000
43	C	0.000
46	C	0.000
49	C	0.000
52	C	0.000
55	C	0.000
58	C	0.000
61	C	0.000
95	C	0.000
99	C	0.000
103	C	0.000
109	C	0.000
113	C	0.000
129	C	0.000
130	C	0.000
134	C	0.000
138	C	0.000
143	C	0.000
147	C	0.000
151	C	0.000
153	H	0.000
25	H	0.001
53	H	0.001
119	H	0.001
146	H	0.001
152	H	0.001
32	H	0.002
33	C	0.002
37	H	0.002
100	H	0.002
139	H	0.002
144	H	0.002
149	H	0.002
19	C	0.003
29	H	0.003
35	C	0.003
57	H	0.003
79	H	0.003
108	H	0.003
110	H	0.003
148	H	0.003
41	H	0.004

50	H	0.004
76	H	0.004
93	H	0.004
96	H	0.004
112	H	0.004
123	C	0.004
1	O	0.005
48	H	0.005
60	H	0.005
70	C	0.005
88	C	0.005
92	C	0.005
4	O	0.006
26	H	0.006
34	H	0.006
47	H	0.006
65	C	0.006
128	H	0.006
51	H	0.008
98	H	0.008
127	C	0.008
131	H	0.008
133	H	0.008
136	H	0.008
140	H	0.008
141	H	0.008
17	C	0.009
42	H	0.009
54	H	0.009
97	H	0.009
104	H	0.009
105	H	0.009
154	H	0.009
28	H	0.010
56	H	0.010
66	H	0.010
89	C	0.010
91	C	0.010
102	H	0.010
118	H	0.010
126	C	0.010
145	H	0.010
150	H	0.010
15	C	0.011
111	H	0.011
115	H	0.011
24	H	0.012
62	H	0.012
63	H	0.012
72	C	0.012
90	H	0.012
124	C	0.012

125	H	0.012
64	C	0.013
14	H	0.015
71	H	0.015
84	C	0.015
9	H	0.016
8	N	0.018
18	H	0.019
83	H	0.019
16	H	0.021
73	H	0.021
82	H	0.023
75	H	0.030
80	H	0.034

5•6$_{O}$···K^+

Number	Atom	VDD
1	K	–0.073
6	O	–0.071
2	O	–0.053
3	O	–0.049
86	C	–0.030
37	C	–0.026
24	H	–0.021
38	C	–0.021
87	C	–0.018
93	C	–0.018
114	H	–0.018
99	H	–0.015
13	H	–0.014
35	C	–0.014
52	H	–0.013
118	H	–0.012
7	N	–0.011
147	H	–0.011
11	C	–0.009
40	C	–0.008
8	N	–0.007
121	C	–0.007
4	O	–0.006
103	H	–0.006
12	C	–0.005
21	C	–0.005
129	H	–0.005
5	O	–0.004
45	C	–0.004
94	H	–0.004
154	H	–0.004
66	C	–0.003
72	C	–0.003
92	C	–0.003
29	C	–0.002

42	C	–0.002
48	C	–0.002
80	H	–0.002
95	C	–0.002
108	C	–0.002
128	C	–0.002
138	H	–0.002
33	C	–0.001
61	H	–0.001
71	C	–0.001
85	H	–0.001
100	C	–0.001
117	C	–0.001
130	C	–0.001
141	H	–0.001
145	H	–0.001
22	C	0.000
47	H	0.000
51	C	0.000
54	C	0.000
57	C	0.000
60	C	0.000
67	C	0.000
73	H	0.000
74	C	0.000
76	C	0.000
79	C	0.000
81	H	0.000
83	C	0.000
88	H	0.000
96	C	0.000
104	C	0.000
109	C	0.000
113	C	0.000
131	C	0.000
135	C	0.000
137	H	0.000
139	C	0.000
143	C	0.000
144	C	0.000
148	C	0.000
152	C	0.000
25	C	0.001
75	H	0.001
90	C	0.001
14	C	0.002
19	C	0.002
32	C	0.002
69	C	0.002
89	C	0.002
98	H	0.002
134	H	0.002

77	H	0.003
123	H	0.003
127	C	0.003
133	H	0.003
142	H	0.003
149	H	0.003
58	H	0.004
106	H	0.004
122	C	0.004
27	H	0.005
39	H	0.005
70	H	0.005
101	H	0.005
111	H	0.005
115	H	0.005
120	H	0.005
146	H	0.005
155	H	0.005
15	C	0.006
20	H	0.006
23	H	0.006
26	H	0.006
105	H	0.006
110	H	0.006
150	H	0.006
153	H	0.006
17	C	0.007
63	C	0.007
64	H	0.007
68	H	0.007
124	C	0.007
151	H	0.007
10	H	0.008
55	H	0.008
59	H	0.008
78	H	0.008
82	H	0.008
84	H	0.008
132	H	0.008
36	H	0.009
62	H	0.009
65	H	0.009
125	C	0.009
136	H	0.009
140	H	0.009
9	N	0.010
31	H	0.010
50	H	0.010
53	H	0.010
126	H	0.010
16	H	0.011
56	H	0.011

91	H	0.011
102	H	0.011
112	H	0.011
119	H	0.011
18	H	0.012
28	H	0.012
107	H	0.012
30	H	0.013
41	H	0.013
116	H	0.013
97	H	0.014
44	H	0.015
34	H	0.017
43	H	0.021
49	H	0.022
46	H	0.024

5•6$_{O}$···Li^{+}

Number	Atom	VDD
1	Li	–0.161
6	O	–0.120
2	O	–0.080
3	O	–0.063
4	O	–0.061
121	C	–0.034
37	C	–0.014
122	C	–0.013
128	C	–0.012
61	H	–0.010
86	C	–0.010
154	H	–0.009
141	H	–0.008
24	H	–0.007
13	H	–0.006
48	C	–0.006
12	C	–0.005
51	C	–0.005
54	C	–0.005
94	H	–0.005
118	H	–0.005
138	H	–0.005
39	H	–0.004
66	C	–0.004
69	C	–0.004
147	H	–0.004
8	N	–0.003
38	C	–0.003
45	C	–0.003
72	C	–0.003
81	H	–0.003
120	H	–0.003
5	O	–0.002

7	N	–0.002
14	C	–0.002
21	C	–0.002
59	H	–0.002
98	H	–0.002
130	C	–0.002
143	C	–0.002
43	H	–0.001
57	C	–0.001
60	C	–0.001
76	C	–0.001
80	H	–0.001
93	C	–0.001
95	C	–0.001
99	H	–0.001
108	C	–0.001
114	H	–0.001
22	C	0.000
25	C	0.000
29	C	0.000
67	C	0.000
79	C	0.000
83	C	0.000
96	C	0.000
100	C	0.000
103	H	0.000
104	C	0.000
109	C	0.000
113	C	0.000
117	C	0.000
123	H	0.000
131	C	0.000
135	C	0.000
139	C	0.000
144	C	0.000
148	C	0.000
152	C	0.000
50	H	0.001
71	C	0.001
85	H	0.001
106	H	0.001
111	H	0.001
129	H	0.001
153	H	0.001
19	C	0.002
42	C	0.002
70	H	0.002
73	H	0.002
127	C	0.002
15	C	0.003
35	C	0.003
52	H	0.003

74	C	0.003
77	H	0.003
88	H	0.003
124	C	0.003
150	H	0.003
26	H	0.004
36	H	0.004
101	H	0.004
105	H	0.004
125	C	0.004
142	H	0.004
17	C	0.005
23	H	0.005
27	H	0.005
40	C	0.005
75	H	0.005
87	C	0.005
110	H	0.005
115	H	0.005
133	H	0.005
137	H	0.005
20	H	0.006
134	H	0.006
16	H	0.007
30	H	0.007
33	C	0.007
92	C	0.007
145	H	0.007
149	H	0.007
28	H	0.008
41	H	0.008
68	H	0.008
84	H	0.008
89	C	0.008
97	H	0.008
18	H	0.009
31	H	0.009
63	C	0.009
65	H	0.009
78	H	0.009
102	H	0.009
107	H	0.009
112	H	0.009
116	H	0.009
119	H	0.009
9	N	0.010
62	H	0.010
82	H	0.010
64	H	0.011
90	C	0.011
126	H	0.011
32	C	0.012

91	H	0.012
132	H	0.012
140	H	0.012
146	H	0.012
151	H	0.012
155	H	0.012
34	H	0.013
55	H	0.013
136	H	0.013
10	H	0.015
11	C	0.018
47	H	0.019
44	H	0.021
58	H	0.023
46	H	0.028
56	H	0.029
53	H	0.032
49	H	0.033

5•6$_{N^-}$···Na$^+$

Number	Atom	VDD
155	Na	–0.110
5	O	–0.104
1	O	–0.071
21	H	–0.039
35	C	–0.036
17	C	–0.035
120	C	–0.029
36	C	–0.025
121	C	–0.024
19	C	–0.022
33	C	–0.018
18	H	–0.015
122	H	–0.012
44	H	–0.011
131	H	–0.010
30	C	–0.009
84	C	–0.009
87	H	–0.009
38	C	–0.008
31	C	–0.007
76	H	–0.007
2	O	–0.006
3	O	–0.005
15	C	–0.005
104	H	–0.005
140	H	–0.005
6	N	–0.004
8	N	–0.004
37	H	–0.004
59	H	–0.004
7	N	–0.003

153	H	–0.003
20	C	–0.002
40	C	–0.002
86	C	–0.002
106	H	–0.002
129	C	–0.002
142	C	–0.002
27	C	–0.001
81	C	–0.001
94	C	–0.001
107	C	–0.001
116	C	–0.001
146	H	–0.001
23	C	0.000
43	C	0.000
46	C	0.000
49	C	0.000
52	C	0.000
55	C	0.000
58	C	0.000
61	C	0.000
74	C	0.000
77	C	0.000
95	C	0.000
99	C	0.000
103	C	0.000
109	C	0.000
113	C	0.000
117	H	0.000
119	H	0.000
127	C	0.000
130	C	0.000
134	C	0.000
138	C	0.000
143	C	0.000
147	C	0.000
151	C	0.000
10	C	0.001
67	C	0.001
69	C	0.001
98	H	0.001
101	H	0.001
110	H	0.001
114	H	0.001
57	H	0.002
70	C	0.002
71	H	0.002
72	C	0.002
85	C	0.002
152	H	0.002
50	H	0.003
53	H	0.003

132	H	0.003
149	H	0.003
13	C	0.004
128	H	0.004
137	H	0.004
144	H	0.004
4	O	0.005
34	H	0.005
92	C	0.005
108	H	0.005
112	H	0.005
123	C	0.005
148	H	0.005
25	H	0.006
68	H	0.006
75	H	0.006
78	H	0.006
93	H	0.006
96	H	0.006
100	H	0.006
139	H	0.006
60	H	0.007
73	H	0.007
135	H	0.007
9	H	0.008
16	H	0.008
29	H	0.008
65	C	0.008
79	H	0.008
124	C	0.008
126	C	0.008
39	H	0.009
63	H	0.009
82	H	0.009
88	C	0.009
91	C	0.009
12	C	0.010
47	H	0.010
51	H	0.010
54	H	0.010
56	H	0.010
64	C	0.010
80	H	0.010
105	H	0.010
118	H	0.010
133	H	0.010
11	H	0.011
62	H	0.011
97	H	0.011
102	H	0.011
111	H	0.011
136	H	0.011

141	H	0.011
154	H	0.011
24	H	0.012
26	H	0.012
32	H	0.012
45	H	0.012
48	H	0.012
66	H	0.012
83	H	0.012
115	H	0.012
125	H	0.012
145	H	0.012
150	H	0.012
22	H	0.013
14	H	0.014
28	H	0.014
41	H	0.015
42	H	0.016
89	C	0.016
90	H	0.016

5•6$_O$$^{-}$····Na^{+}

Number	Atom	VDD
5	O	–0.121
155	Na	–0.095
1	O	–0.067
3	O	–0.061
2	O	–0.056
127	C	–0.029
60	H	–0.027
120	C	–0.024
36	C	–0.021
153	H	–0.020
121	C	–0.014
126	C	–0.014
140	H	–0.012
38	H	–0.010
146	H	–0.009
37	C	–0.008
124	C	–0.008
44	C	–0.007
10	C	–0.006
93	H	–0.006
23	H	–0.005
42	H	–0.005
47	C	–0.005
50	C	–0.005
117	H	–0.005
141	H	–0.005
41	C	–0.004
53	C	–0.004
85	C	–0.004

92	C	–0.004
98	H	–0.004
6	N	–0.003
7	N	–0.003
102	H	–0.003
119	H	–0.003
128	H	–0.003
142	C	–0.003
56	C	–0.002
59	C	–0.002
80	H	–0.002
86	C	–0.002
123	C	–0.002
20	C	–0.001
34	C	–0.001
78	C	–0.001
87	H	–0.001
94	C	–0.001
107	C	–0.001
129	C	–0.001
134	C	–0.001
137	H	–0.001
151	C	–0.001
8	N	0.000
21	C	0.000
24	C	0.000
28	C	0.000
79	H	0.000
95	C	0.000
99	C	0.000
103	C	0.000
108	C	0.000
110	H	0.000
112	C	0.000
113	H	0.000
116	C	0.000
130	C	0.000
132	H	0.000
138	C	0.000
143	C	0.000
147	C	0.000
62	C	0.001
73	C	0.001
75	C	0.001
82	C	0.001
39	C	0.002
46	H	0.002
122	H	0.002
25	H	0.003
35	H	0.003
97	H	0.003
100	H	0.003

149	H	0.003
152	H	0.003
12	H	0.004
14	C	0.004
40	H	0.004
68	C	0.004
74	H	0.004
104	H	0.004
105	H	0.004
11	C	0.005
22	H	0.005
31	C	0.005
32	C	0.005
61	H	0.005
66	C	0.005
71	C	0.005
109	H	0.005
114	H	0.005
9	H	0.006
13	C	0.006
26	H	0.006
51	H	0.006
65	C	0.006
84	H	0.006
88	C	0.006
91	C	0.006
18	C	0.007
29	H	0.007
30	H	0.007
96	H	0.007
125	H	0.007
133	H	0.007
148	H	0.007
27	H	0.008
54	H	0.008
57	H	0.008
72	H	0.008
76	H	0.008
89	C	0.008
106	H	0.008
111	H	0.008
115	H	0.008
118	H	0.008
136	H	0.008
144	H	0.008
4	O	0.009
63	H	0.009
101	H	0.009
15	H	0.010
67	H	0.010
81	H	0.010
16	C	0.011

19	H	0.011
33	H	0.011
49	H	0.011
69	H	0.011
70	C	0.011
83	H	0.011
90	H	0.011
131	H	0.011
135	H	0.011
77	H	0.012
145	H	0.012
150	H	0.012
139	H	0.013
154	H	0.013
64	H	0.015
17	H	0.016
43	H	0.018
58	H	0.022
48	H	0.023
52	H	0.026
55	H	0.026
45	H	0.027

5•6$_N$(a)···Na^+

Number	Atom	VDD
161	Na	–0.118
81	O	–0.103
99	C	–0.049
12	C	–0.036
138	H	–0.035
134	H	–0.033
142	H	–0.030
7	C	–0.023
156	H	–0.022
43	H	–0.019
20	C	–0.017
21	C	–0.017
11	C	–0.015
75	H	–0.015
16	C	–0.013
18	C	–0.012
19	C	–0.012
17	C	–0.011
98	C	–0.010
6	N	–0.007
66	H	–0.007
118	H	–0.007
33	C	–0.006
54	H	–0.006
72	H	–0.006
100	C	–0.006
3	O	–0.005

13	C	–0.005
34	C	–0.005
104	C	–0.005
5	N	–0.004
2	O	–0.003
60	H	–0.003
122	H	–0.003
15	C	–0.002
38	C	–0.002
56	H	–0.002
108	C	–0.002
148	H	–0.002
22	C	–0.001
24	C	–0.001
90	C	–0.001
94	C	–0.001
106	C	–0.001
111	C	–0.001
153	H	–0.001
8	C	0.000
14	C	0.000
23	C	0.000
25	C	0.000
26	C	0.000
27	C	0.000
28	C	0.000
29	C	0.000
36	C	0.000
37	C	0.000
84	C	0.000
85	C	0.000
91	C	0.000
92	C	0.000
93	C	0.000
95	C	0.000
96	C	0.000
97	C	0.000
105	C	0.000
107	C	0.000
109	C	0.000
110	C	0.000
114	N	0.000
125	H	0.000
131	H	0.000
35	C	0.001
64	H	0.001
112	N	0.001
146	H	0.001
160	H	0.001
1	O	0.002
10	C	0.002
32	C	0.002

82	N	0.002
116	H	0.002
133	H	0.002
155	H	0.002
62	H	0.003
73	H	0.003
89	C	0.003
127	H	0.003
129	H	0.003
149	H	0.003
152	H	0.003
4	O	0.004
9	C	0.004
31	C	0.004
67	H	0.004
119	H	0.004
128	H	0.004
58	H	0.005
71	H	0.005
77	H	0.005
83	C	0.005
86	C	0.005
101	C	0.005
103	C	0.005
120	H	0.005
30	C	0.006
57	H	0.006
69	H	0.006
113	N	0.006
150	H	0.006
39	H	0.007
61	H	0.007
76	H	0.007
123	H	0.007
124	H	0.007
159	H	0.007
42	H	0.008
46	H	0.008
63	H	0.008
87	C	0.008
121	H	0.008
144	H	0.008
52	H	0.009
59	H	0.009
65	H	0.009
68	H	0.009
70	H	0.009
74	H	0.009
80	H	0.009
117	H	0.009
130	H	0.009
132	H	0.009

137	H	0.009
157	H	0.009
158	H	0.009
45	H	0.010
49	H	0.010
88	C	0.010
126	H	0.010
136	H	0.010
143	H	0.010
135	H	0.011
140	H	0.011
154	H	0.011
78	H	0.012
115	H	0.012
145	H	0.012
47	H	0.013
51	H	0.013
55	H	0.013
79	H	0.013
141	H	0.013
147	H	0.013
151	H	0.013
53	H	0.014
102	C	0.014
50	H	0.015
139	H	0.016
41	H	0.017
48	H	0.017
40	H	0.018
44	H	0.018

5•6$_N$(b)···Na^+

Number	Atom	VDD
161	Na	–0.108
81	O	–0.105
112	N	–0.066
142	H	–0.055
12	C	–0.037
43	H	–0.032
99	C	–0.027
11	C	–0.022
17	C	–0.017
16	C	–0.014
57	H	–0.013
18	C	–0.012
19	C	–0.012
21	C	–0.012
5	N	–0.011
84	C	–0.011
7	C	–0.010
140	H	–0.010
20	C	–0.009

127	H	–0.008
60	H	–0.007
116	H	–0.007
13	C	–0.006
98	C	–0.006
155	H	–0.006
3	O	–0.005
6	N	–0.005
123	H	–0.005
4	O	–0.004
34	C	–0.003
42	H	–0.003
54	H	–0.003
77	H	–0.003
85	C	–0.003
131	H	–0.003
138	H	–0.003
15	C	–0.002
33	C	–0.002
100	C	–0.002
130	H	–0.002
22	C	–0.001
35	C	–0.001
38	C	–0.001
90	C	–0.001
94	C	–0.001
104	C	–0.001
106	C	–0.001
108	C	–0.001
14	C	0.000
23	C	0.000
24	C	0.000
25	C	0.000
26	C	0.000
27	C	0.000
28	C	0.000
29	C	0.000
36	C	0.000
37	C	0.000
72	H	0.000
91	C	0.000
92	C	0.000
93	C	0.000
95	C	0.000
96	C	0.000
97	C	0.000
105	C	0.000
107	C	0.000
109	C	0.000
110	C	0.000
111	C	0.000
125	H	0.000

132	H	0.000
32	C	0.001
64	H	0.001
118	H	0.001
150	H	0.001
152	H	0.001
153	H	0.001
10	C	0.002
30	C	0.002
73	H	0.002
89	C	0.002
134	H	0.002
143	H	0.002
157	H	0.002
31	C	0.003
56	H	0.003
63	H	0.003
69	H	0.003
121	H	0.003
148	H	0.003
2	O	0.004
66	H	0.004
76	H	0.004
114	N	0.004
128	H	0.004
136	H	0.004
146	H	0.004
149	H	0.004
59	H	0.005
86	C	0.005
103	C	0.005
113	N	0.005
119	H	0.005
122	H	0.005
8	C	0.006
9	C	0.006
61	H	0.006
71	H	0.006
74	H	0.006
79	H	0.006
88	C	0.006
159	H	0.006
46	H	0.007
70	H	0.007
82	N	0.007
83	C	0.007
101	C	0.007
156	H	0.007
39	H	0.008
1	O	0.009
65	H	0.009
67	H	0.009

87	C	0.009
120	H	0.009
124	H	0.009
126	H	0.009
133	H	0.009
135	H	0.009
139	H	0.009
158	H	0.009
58	H	0.010
62	H	0.010
68	H	0.010
129	H	0.010
145	H	0.010
45	H	0.011
75	H	0.011
117	H	0.011
151	H	0.011
160	H	0.011
41	H	0.012
50	H	0.012
115	H	0.012
137	H	0.012
144	H	0.012
147	H	0.012
154	H	0.012
78	H	0.013
102	C	0.013
49	H	0.014
80	H	0.014
40	H	0.015
51	H	0.015
52	H	0.015
53	H	0.015
47	H	0.016
48	H	0.016
55	H	0.017
141	H	0.019
44	H	0.021

5•6$_N$(c)···Na^+

Number	Atom	VDD
161	Na	–0.101
81	O	–0.098
114	O	–0.070
5	N	–0.069
140	H	–0.042
12	C	–0.035
148	H	–0.033
21	C	–0.030
99	C	–0.022
53	H	–0.021
7	C	–0.020

16	C	–0.019
75	H	–0.013
11	C	–0.010
46	H	–0.008
13	C	–0.007
98	C	–0.007
72	H	–0.006
84	C	–0.006
85	C	–0.006
3	O	–0.005
6	N	–0.005
14	C	–0.005
15	C	–0.005
33	C	–0.005
34	C	–0.005
56	H	–0.005
112	N	–0.005
45	H	–0.004
60	H	–0.004
128	H	–0.004
57	H	–0.003
64	H	–0.003
66	H	–0.003
130	H	–0.003
146	H	–0.003
137	H	–0.002
144	H	–0.002
154	H	–0.002
27	C	–0.001
35	C	–0.001
38	C	–0.001
90	C	–0.001
93	C	–0.001
94	C	–0.001
104	C	–0.001
108	C	–0.001
111	C	–0.001
115	N	–0.001
159	H	–0.001
22	C	0.000
23	C	0.000
24	C	0.000
25	C	0.000
26	C	0.000
28	C	0.000
29	C	0.000
36	C	0.000
37	C	0.000
92	C	0.000
96	C	0.000
97	C	0.000
105	C	0.000

106	C	0.000
107	C	0.000
109	C	0.000
110	C	0.000
118	N	0.000
119	O	0.000
124	H	0.000
2	O	0.001
73	H	0.001
91	C	0.001
95	C	0.001
126	H	0.001
135	H	0.001
20	C	0.002
32	C	0.002
39	H	0.002
100	C	0.002
113	O	0.002
131	H	0.002
139	H	0.002
152	H	0.002
158	H	0.002
69	H	0.003
155	H	0.003
156	H	0.003
10	C	0.004
17	C	0.004
133	H	0.004
149	H	0.004
1	O	0.005
8	C	0.005
30	C	0.005
31	C	0.005
63	H	0.005
67	H	0.005
71	H	0.005
77	H	0.005
83	C	0.005
89	C	0.005
134	H	0.005
49	H	0.006
65	H	0.006
76	H	0.006
86	C	0.006
103	C	0.006
122	H	0.006
4	O	0.007
9	C	0.007
52	H	0.007
58	H	0.007
59	H	0.007
62	H	0.007

74	H	0.007
127	H	0.007
142	H	0.007
54	H	0.008
61	H	0.008
80	H	0.008
82	N	0.008
87	C	0.008
125	H	0.008
42	H	0.009
43	H	0.009
68	H	0.009
70	H	0.009
101	C	0.009
123	H	0.009
136	H	0.009
138	H	0.009
143	H	0.009
145	H	0.009
150	H	0.009
18	C	0.010
19	C	0.010
117	O	0.010
129	H	0.010
132	H	0.010
151	H	0.010
160	H	0.010
78	H	0.011
79	H	0.011
88	C	0.011
102	C	0.011
141	H	0.011
153	H	0.011
157	H	0.011
55	H	0.012
121	H	0.012
50	H	0.013
116	O	0.013
147	H	0.014
120	O	0.015
41	H	0.017
40	H	0.018
51	H	0.018
44	H	0.025
48	H	0.025
47	H	0.029

5•6$_N$(d)···Na^+

Number	Atom	VDD
161	Na	–0.099
81	O	–0.096
117	O	–0.085

43	H	–0.046
12	C	–0.037
122	H	–0.036
17	C	–0.033
158	H	–0.029
56	H	–0.027
131	H	–0.025
85	C	–0.024
18	C	–0.019
7	C	–0.016
11	C	–0.014
77	H	–0.013
50	H	–0.011
84	C	–0.011
103	C	–0.010
127	H	–0.009
6	N	–0.008
98	C	–0.007
4	O	–0.006
16	C	–0.006
156	H	–0.006
5	N	–0.005
2	O	–0.004
3	O	–0.004
13	C	–0.003
57	H	–0.003
15	C	–0.002
32	C	–0.002
33	C	–0.002
62	H	–0.002
71	H	–0.002
134	H	–0.002
142	H	–0.002
22	C	–0.001
31	C	–0.001
38	C	–0.001
90	C	–0.001
94	C	–0.001
104	C	–0.001
110	C	–0.001
112	N	–0.001
118	N	–0.001
14	C	0.000
23	C	0.000
24	C	0.000
25	C	0.000
26	C	0.000
27	C	0.000
28	C	0.000
29	C	0.000
36	C	0.000
37	C	0.000

51	H	0.000
76	H	0.000
91	C	0.000
92	C	0.000
93	C	0.000
95	C	0.000
96	C	0.000
97	C	0.000
105	C	0.000
106	C	0.000
107	C	0.000
108	C	0.000
109	C	0.000
146	H	0.000
70	H	0.001
102	C	0.001
111	C	0.001
149	H	0.001
151	H	0.001
34	C	0.002
64	H	0.002
133	H	0.002
140	H	0.002
143	H	0.002
148	H	0.002
30	C	0.003
35	C	0.003
39	H	0.003
66	H	0.003
136	H	0.003
137	H	0.003
138	H	0.003
19	C	0.004
42	H	0.004
144	H	0.004
159	H	0.004
8	C	0.005
58	H	0.005
68	H	0.005
75	H	0.005
83	C	0.005
86	C	0.005
99	C	0.005
129	H	0.005
153	H	0.005
154	H	0.005
160	H	0.005
10	C	0.006
21	C	0.006
61	H	0.006
72	H	0.006
113	O	0.006

124	H	0.006
1	O	0.007
46	H	0.007
69	H	0.007
73	H	0.007
89	C	0.007
101	C	0.007
114	O	0.007
115	N	0.007
157	H	0.007
9	C	0.008
60	H	0.008
65	H	0.008
67	H	0.008
125	H	0.008
128	H	0.008
20	C	0.009
49	H	0.009
54	H	0.009
82	N	0.009
145	H	0.009
150	H	0.009
152	H	0.009
155	H	0.009
63	H	0.010
79	H	0.010
87	C	0.010
100	C	0.010
126	H	0.010
130	H	0.010
141	H	0.010
147	H	0.010
59	H	0.011
74	H	0.011
78	H	0.011
80	H	0.011
88	C	0.011
119	O	0.011
120	O	0.011
121	H	0.011
123	H	0.011
132	H	0.011
135	H	0.011
139	H	0.011
45	H	0.012
47	H	0.013
48	H	0.016
53	H	0.016
55	H	0.016
40	H	0.017
41	H	0.017
44	H	0.018

52	H	0.018
116	O	0.034

5•6$_N$(e)···Na$^+$

Number	Atom	VDD
89	O	–0.117
163	Na	–0.109
47	H	–0.053
12	C	–0.038
107	C	–0.034
11	C	–0.031
150	H	–0.029
58	H	–0.027
142	H	–0.025
17	C	–0.019
18	C	–0.017
106	C	–0.014
146	H	–0.014
19	C	–0.013
20	C	–0.012
76	H	–0.012
16	C	–0.011
21	C	–0.011
121	H	–0.010
46	H	–0.008
74	H	–0.008
92	C	–0.008
7	C	–0.007
2	O	–0.006
3	O	–0.006
34	C	–0.006
130	H	–0.006
4	O	–0.005
5	N	–0.005
6	N	–0.004
132	H	–0.004
13	C	–0.003
33	C	–0.003
108	C	–0.003
112	C	–0.003
161	H	–0.003
22	C	–0.002
35	C	–0.002
93	C	–0.002
15	C	–0.001
38	C	–0.001
98	C	–0.001
102	C	–0.001
116	C	–0.001
117	C	–0.001
139	H	–0.001
141	H	–0.001

160	H	–0.001
10	C	0.000
23	C	0.000
24	C	0.000
25	C	0.000
26	C	0.000
27	C	0.000
28	C	0.000
29	C	0.000
36	C	0.000
37	C	0.000
39	N	0.000
40	N	0.000
41	N	0.000
56	H	0.000
64	H	0.000
99	C	0.000
100	C	0.000
101	C	0.000
103	C	0.000
104	C	0.000
105	C	0.000
113	C	0.000
114	C	0.000
115	C	0.000
118	C	0.000
119	C	0.000
126	H	0.000
128	H	0.000
156	H	0.000
14	C	0.001
68	H	0.001
75	H	0.001
133	H	0.001
137	H	0.001
158	H	0.001
32	C	0.002
30	C	0.003
62	H	0.003
66	H	0.003
82	H	0.003
123	H	0.003
135	H	0.003
136	H	0.003
31	C	0.004
61	H	0.004
71	H	0.004
59	H	0.005
73	H	0.005
78	H	0.005
94	C	0.005
97	C	0.005

109	C	0.005
154	H	0.005
157	H	0.005
77	H	0.006
80	H	0.006
111	C	0.006
124	H	0.006
127	H	0.006
84	H	0.007
151	H	0.007
9	C	0.008
42	N	0.008
49	H	0.008
63	H	0.008
67	H	0.008
69	H	0.008
72	H	0.008
88	H	0.008
90	N	0.008
96	C	0.008
125	H	0.008
129	H	0.008
131	H	0.008
144	H	0.008
145	H	0.008
148	H	0.008
152	H	0.008
1	O	0.009
43	H	0.009
51	H	0.009
65	H	0.009
70	H	0.009
79	H	0.009
81	H	0.009
134	H	0.009
138	H	0.009
140	H	0.009
8	C	0.010
143	H	0.010
52	H	0.011
54	H	0.011
60	H	0.011
85	H	0.011
95	C	0.011
110	C	0.011
122	H	0.011
147	H	0.011
153	H	0.011
155	H	0.011
48	H	0.012
149	H	0.012
159	H	0.012

162	H	0.012
53	H	0.013
83	H	0.013
45	H	0.014
50	H	0.014
87	H	0.014
55	H	0.015
44	H	0.016
86	H	0.016
91	C	0.016
120	H	0.016
57	H	0.017

5•6$_N$(f)···Na^+

Number	Atom	VDD
89	O	–0.105
47	O	–0.103
163	Na	–0.091
1	O	–0.075
107	C	–0.038
19	C	–0.029
146	H	–0.029
55	H	–0.023
69	H	–0.022
106	C	–0.019
142	H	–0.019
150	H	–0.017
18	C	–0.016
12	C	–0.011
20	C	–0.010
92	C	–0.009
51	H	–0.008
3	O	–0.007
67	H	–0.006
108	C	–0.006
130	H	–0.006
7	C	–0.005
34	C	–0.005
82	H	–0.005
84	H	–0.005
156	H	–0.005
4	O	–0.004
35	C	–0.004
121	H	–0.004
112	C	–0.003
6	N	–0.002
15	C	–0.002
23	C	–0.002
38	C	–0.002
116	C	–0.002
126	H	–0.002
141	H	–0.002

161	H	–0.002
22	C	–0.001
36	C	–0.001
39	N	–0.001
70	H	–0.001
76	H	–0.001
83	H	–0.001
93	C	–0.001
98	C	–0.001
102	C	–0.001
114	C	–0.001
119	C	–0.001
132	H	–0.001
139	H	–0.001
14	C	0.000
24	C	0.000
25	C	0.000
26	C	0.000
27	C	0.000
28	C	0.000
29	C	0.000
33	C	0.000
37	C	0.000
42	N	0.000
48	N	0.000
88	H	0.000
99	C	0.000
100	C	0.000
101	C	0.000
103	C	0.000
104	C	0.000
105	C	0.000
113	C	0.000
115	C	0.000
117	C	0.000
118	C	0.000
133	H	0.000
11	C	0.001
30	C	0.001
86	H	0.001
123	H	0.001
128	H	0.001
137	H	0.001
31	C	0.002
32	C	0.002
54	H	0.002
79	H	0.002
154	H	0.002
17	C	0.003
21	C	0.003
40	O	0.003
61	H	0.003

85	H	0.003
135	H	0.003
136	H	0.003
5	N	0.004
13	C	0.004
97	C	0.004
109	C	0.004
111	C	0.004
157	H	0.004
160	H	0.004
2	O	0.005
8	C	0.005
81	H	0.005
90	N	0.005
124	H	0.005
127	H	0.005
10	C	0.006
57	H	0.006
62	H	0.006
72	H	0.006
74	H	0.006
75	H	0.006
80	H	0.006
94	C	0.006
131	H	0.006
158	H	0.006
9	C	0.007
87	H	0.007
96	C	0.007
144	H	0.007
145	H	0.007
152	H	0.007
16	C	0.008
44	O	0.008
58	H	0.008
125	H	0.008
129	H	0.008
140	H	0.008
59	H	0.009
73	H	0.009
77	H	0.009
78	H	0.009
134	H	0.009
138	H	0.009
148	H	0.009
151	H	0.009
43	O	0.010
45	N	0.010
50	O	0.010
52	H	0.010
71	H	0.010
122	H	0.010

41	O	0.011
53	H	0.011
68	H	0.011
95	C	0.011
110	C	0.011
143	H	0.011
149	H	0.011
153	H	0.011
162	H	0.011
56	H	0.012
159	H	0.012
60	H	0.013
91	C	0.013
155	H	0.013
63	H	0.014
147	H	0.014
120	H	0.015
65	H	0.018
64	H	0.020
49	O	0.021
66	H	0.023
46	O	0.052

5•6$_O$(a)···Na^+

Number	Atom	VDD
6	O	–0.105
1	Na	–0.094
2	O	–0.065
4	O	–0.061
3	O	–0.060
58	C	–0.023
57	C	–0.022
23	C	–0.021
102	H	–0.021
145	H	–0.020
149	H	–0.018
62	C	–0.017
156	H	–0.013
59	C	–0.012
60	C	–0.010
80	H	–0.007
143	H	–0.007
27	C	–0.006
147	H	–0.006
8	N	–0.005
11	C	–0.005
28	C	–0.005
29	C	–0.005
30	C	–0.005
61	C	–0.005
75	H	–0.005
132	H	–0.005

16	C	–0.004
43	C	–0.004
113	H	–0.004
24	C	–0.003
34	C	–0.003
39	C	–0.003
63	C	–0.003
129	H	–0.003
154	H	–0.003
161	H	–0.003
7	N	–0.002
22	C	–0.002
26	C	–0.002
32	C	–0.002
67	C	–0.002
112	H	–0.002
122	H	–0.002
5	O	–0.001
19	C	–0.001
31	C	–0.001
38	C	–0.001
49	C	–0.001
53	C	–0.001
65	C	–0.001
66	C	–0.001
68	C	–0.001
70	C	–0.001
71	N	–0.001
109	H	–0.001
119	H	–0.001
12	C	0.000
17	C	0.000
18	C	0.000
33	C	0.000
40	C	0.000
41	C	0.000
42	C	0.000
48	C	0.000
50	C	0.000
51	C	0.000
52	C	0.000
54	C	0.000
55	C	0.000
56	C	0.000
64	C	0.000
69	C	0.000
88	H	0.000
15	C	0.001
35	C	0.001
73	N	0.001
108	H	0.001
120	H	0.001

146	H	0.001
9	N	0.002
36	C	0.002
44	C	0.002
87	H	0.002
123	H	0.002
13	C	0.003
37	C	0.003
82	H	0.003
90	H	0.003
116	H	0.003
118	H	0.003
127	H	0.003
134	H	0.003
135	H	0.003
139	H	0.003
14	C	0.004
25	C	0.004
81	H	0.004
93	H	0.004
101	H	0.004
150	H	0.004
21	C	0.005
78	H	0.005
106	H	0.005
110	H	0.005
124	H	0.005
130	H	0.005
137	H	0.005
47	C	0.006
79	H	0.006
126	H	0.006
157	H	0.006
72	N	0.007
76	H	0.007
85	H	0.007
107	H	0.007
133	H	0.007
153	H	0.007
160	H	0.007
10	C	0.008
45	C	0.008
46	C	0.008
84	H	0.008
89	H	0.008
96	H	0.008
104	H	0.008
111	H	0.008
115	H	0.008
121	H	0.008
125	H	0.008
128	H	0.008

140	H	0.008
77	H	0.009
83	H	0.009
98	H	0.009
114	H	0.009
117	H	0.009
131	H	0.009
142	H	0.009
151	H	0.009
159	H	0.009
20	C	0.010
105	H	0.010
136	H	0.010
158	H	0.010
86	H	0.011
95	H	0.012
103	H	0.012
141	H	0.012
155	H	0.012
74	H	0.013
138	H	0.013
152	H	0.013
144	H	0.015
148	H	0.017
91	H	0.021
100	H	0.022
94	H	0.025
97	H	0.026
99	H	0.027
92	H	0.028

5•6$_O$(b)···Na^+

Number	Atom	VDD
6	O	–0.100
1	Na	–0.099
2	O	–0.072
3	O	–0.062
57	C	–0.040
23	C	–0.027
98	H	–0.027
62	C	–0.024
22	C	–0.022
58	C	–0.019
60	C	–0.017
43	C	–0.009
4	O	–0.008
59	C	–0.008
61	C	–0.008
72	N	–0.008
73	N	–0.008
95	H	–0.008
7	N	–0.007

28	C	–0.007
82	H	–0.007
132	H	–0.007
135	H	–0.007
24	C	–0.006
145	H	–0.006
119	H	–0.005
146	H	–0.005
147	H	–0.005
5	O	–0.004
8	N	–0.004
149	H	–0.004
30	C	–0.003
112	H	–0.003
113	H	–0.003
11	C	–0.002
16	C	–0.002
21	C	–0.002
27	C	–0.002
63	C	–0.002
67	C	–0.002
87	H	–0.002
19	C	–0.001
26	C	–0.001
29	C	–0.001
38	C	–0.001
39	C	–0.001
49	C	–0.001
53	C	–0.001
65	C	–0.001
66	C	–0.001
69	C	–0.001
75	H	–0.001
123	H	–0.001
125	H	–0.001
12	C	0.000
15	C	0.000
17	C	0.000
18	C	0.000
32	C	0.000
33	C	0.000
34	C	0.000
37	C	0.000
40	C	0.000
41	C	0.000
42	C	0.000
48	C	0.000
50	C	0.000
51	C	0.000
52	C	0.000
54	C	0.000
55	C	0.000

56	C	0.000
64	C	0.000
68	C	0.000
70	C	0.000
102	H	0.000
108	H	0.000
31	C	0.001
35	C	0.001
109	H	0.001
122	H	0.001
126	H	0.001
143	H	0.001
154	H	0.001
13	C	0.002
14	C	0.002
36	C	0.002
117	H	0.002
139	H	0.002
9	N	0.003
44	C	0.003
127	H	0.003
137	H	0.003
159	H	0.003
78	H	0.004
81	H	0.004
121	H	0.004
130	H	0.004
133	H	0.004
150	H	0.004
157	H	0.004
47	C	0.005
71	N	0.005
100	H	0.005
106	H	0.005
107	H	0.005
110	H	0.005
129	H	0.005
160	H	0.005
25	C	0.006
76	H	0.006
79	H	0.006
84	H	0.006
105	H	0.006
116	H	0.006
151	H	0.006
156	H	0.006
77	H	0.007
142	H	0.007
153	H	0.007
20	C	0.008
45	C	0.008
104	H	0.008

111	H	0.008
115	H	0.008
124	H	0.008
131	H	0.008
134	H	0.008
136	H	0.008
88	H	0.009
101	H	0.009
114	H	0.009
120	H	0.009
128	H	0.009
140	H	0.009
46	C	0.010
80	H	0.010
83	H	0.010
86	H	0.011
97	H	0.011
103	H	0.011
118	H	0.011
158	H	0.011
161	H	0.012
85	H	0.013
138	H	0.013
141	H	0.013
152	H	0.013
155	H	0.013
10	C	0.014
144	H	0.015
148	H	0.015
74	H	0.016
89	H	0.017
90	H	0.017
93	H	0.017
99	H	0.018
91	H	0.022
96	H	0.022
92	H	0.026
94	H	0.028

5•6$_{O}$(c)···Na^+

Number	Atom	VDD
6	O	–0.105
1	Na	–0.088
3	O	–0.068
2	O	–0.065
4	O	–0.046
57	C	–0.031
62	C	–0.027
94	H	–0.025
73	O	–0.024
154	H	–0.013
23	C	–0.012

159	H	–0.012
24	C	–0.011
78	O	–0.010
108	H	–0.010
43	C	–0.009
58	C	–0.007
27	C	–0.006
28	C	–0.006
150	H	–0.006
29	C	–0.005
30	C	–0.005
61	C	–0.005
81	H	–0.004
86	H	–0.004
119	H	–0.004
142	H	–0.004
148	H	–0.004
8	N	–0.003
11	C	–0.003
77	N	–0.003
137	H	–0.003
7	N	–0.002
16	C	–0.002
48	C	–0.002
63	C	–0.002
67	C	–0.002
118	H	–0.002
128	H	–0.002
139	H	–0.002
12	C	–0.001
32	C	–0.001
34	C	–0.001
40	C	–0.001
53	C	–0.001
70	C	–0.001
71	N	–0.001
74	N	–0.001
102	H	–0.001
126	H	–0.001
134	H	–0.001
5	O	0.000
17	C	0.000
18	C	0.000
19	C	0.000
26	C	0.000
31	C	0.000
33	C	0.000
38	C	0.000
39	C	0.000
41	C	0.000
42	C	0.000
49	C	0.000

50	C	0.000
51	C	0.000
52	C	0.000
54	C	0.000
55	C	0.000
56	C	0.000
60	C	0.000
64	C	0.000
65	C	0.000
66	C	0.000
68	C	0.000
69	C	0.000
129	H	0.000
35	C	0.001
125	H	0.001
151	H	0.001
13	C	0.002
15	C	0.002
36	C	0.002
37	C	0.002
107	H	0.002
114	H	0.002
115	H	0.002
132	H	0.002
14	C	0.003
59	C	0.003
88	H	0.003
122	H	0.003
144	H	0.003
152	H	0.003
9	N	0.004
25	C	0.004
44	C	0.004
87	H	0.004
96	H	0.004
101	H	0.004
135	H	0.004
158	H	0.004
161	H	0.004
22	C	0.005
84	H	0.005
112	H	0.005
116	H	0.005
124	H	0.005
131	H	0.005
140	H	0.005
47	C	0.006
72	O	0.006
82	H	0.006
95	H	0.006
113	H	0.006
145	H	0.006

147	H	0.006
155	H	0.006
156	H	0.006
45	C	0.007
85	H	0.007
91	H	0.007
110	H	0.007
123	H	0.007
127	H	0.007
133	H	0.007
21	C	0.008
79	O	0.008
83	H	0.008
104	H	0.008
121	H	0.008
130	H	0.008
136	H	0.008
138	H	0.008
46	C	0.009
89	H	0.009
90	H	0.009
93	H	0.009
99	H	0.009
111	H	0.009
117	H	0.009
120	H	0.009
141	H	0.010
160	H	0.010
20	C	0.011
75	O	0.011
76	O	0.011
109	H	0.011
157	H	0.011
143	H	0.012
146	H	0.012
10	C	0.013
149	H	0.013
80	H	0.014
153	H	0.014
92	H	0.015
106	H	0.017
97	H	0.020
105	H	0.023
98	H	0.027
103	H	0.028
100	H	0.029

5•6$_O$(d)···Na^+

Number	Atom	VDD
79	O	–0.099
6	O	–0.098
1	Na	–0.085

2	O	–0.075
3	O	–0.072
23	C	–0.028
24	C	–0.027
94	H	–0.021
125	H	–0.021
139	H	–0.016
43	C	–0.014
48	C	–0.013
86	H	–0.009
62	C	–0.008
57	C	–0.006
81	H	–0.006
108	H	–0.006
152	H	–0.006
160	H	–0.006
11	C	–0.005
28	C	–0.005
102	H	–0.005
155	H	–0.005
16	C	–0.004
27	C	–0.004
7	N	–0.003
8	N	–0.003
118	H	–0.003
128	H	–0.003
135	H	–0.003
4	O	–0.002
26	C	–0.002
38	C	–0.002
73	O	–0.002
106	H	–0.002
131	H	–0.002
151	H	–0.002
19	C	–0.001
31	C	–0.001
39	C	–0.001
49	C	–0.001
51	C	–0.001
53	C	–0.001
63	C	–0.001
67	C	–0.001
70	C	–0.001
71	N	–0.001
74	N	–0.001
114	H	–0.001
119	H	–0.001
138	H	–0.001
159	H	–0.001
12	C	0.000
17	C	0.000
18	C	0.000

30	C	0.000
32	C	0.000
33	C	0.000
37	C	0.000
40	C	0.000
41	C	0.000
42	C	0.000
50	C	0.000
52	C	0.000
54	C	0.000
55	C	0.000
56	C	0.000
64	C	0.000
65	C	0.000
66	C	0.000
68	C	0.000
115	H	0.000
145	H	0.000
29	C	0.001
34	C	0.001
61	C	0.001
69	C	0.001
77	N	0.001
129	H	0.001
15	C	0.002
22	C	0.002
36	C	0.002
58	C	0.002
122	H	0.002
156	H	0.002
25	C	0.003
35	C	0.003
109	H	0.003
116	H	0.003
127	H	0.003
141	H	0.003
149	H	0.003
5	O	0.004
13	C	0.004
14	C	0.004
44	C	0.004
47	C	0.004
88	H	0.004
123	H	0.004
132	H	0.004
133	H	0.004
146	H	0.004
60	C	0.005
76	O	0.005
84	H	0.005
110	H	0.005
148	H	0.005

10	C	0.006
87	H	0.006
112	H	0.006
113	H	0.006
158	H	0.006
85	H	0.007
95	H	0.007
101	H	0.007
153	H	0.007
45	C	0.008
82	H	0.008
104	H	0.008
117	H	0.008
121	H	0.008
136	H	0.008
143	H	0.008
154	H	0.008
83	H	0.009
89	H	0.009
90	H	0.009
111	H	0.009
120	H	0.009
130	H	0.009
137	H	0.009
144	H	0.009
147	H	0.009
157	H	0.009
21	C	0.010
46	C	0.010
59	C	0.010
91	H	0.010
126	H	0.010
134	H	0.010
140	H	0.010
142	H	0.010
150	H	0.010
161	H	0.010
80	H	0.011
93	H	0.011
107	H	0.011
124	H	0.011
9	N	0.012
20	C	0.012
99	H	0.013
72	O	0.014
75	O	0.014
96	H	0.014
103	H	0.017
105	H	0.018
92	H	0.019
78	O	0.021
97	H	0.022

98	H	0.027
100	H	0.027

5•6$_O$(e)···Na$^+$

Number	Atom	VDD
6	O	–0.111
1	Na	–0.091
4	O	–0.076
2	O	–0.060
77	N	–0.056
71	N	–0.053
3	O	–0.034
116	H	–0.022
98	H	–0.017
23	C	–0.016
129	H	–0.015
159	H	–0.014
57	C	–0.011
72	N	–0.010
101	H	–0.010
43	C	–0.007
132	H	–0.007
26	C	–0.006
29	C	–0.006
32	C	–0.006
34	C	–0.006
80	H	–0.006
85	H	–0.006
145	H	–0.006
27	C	–0.005
48	C	–0.005
58	C	–0.005
143	H	–0.005
147	H	–0.005
11	C	–0.004
78	N	–0.004
106	H	–0.004
149	H	–0.004
8	N	–0.003
30	C	–0.003
31	C	–0.003
39	C	–0.003
110	H	–0.003
135	H	–0.003
7	N	–0.002
16	C	–0.002
22	C	–0.002
28	C	–0.002
62	C	–0.002
122	H	–0.002
12	C	–0.001
24	C	–0.001

40	C	–0.001
49	C	–0.001
51	C	–0.001
53	C	–0.001
63	C	–0.001
67	C	–0.001
119	H	–0.001
150	H	–0.001
17	C	0.000
18	C	0.000
19	C	0.000
33	C	0.000
38	C	0.000
41	C	0.000
42	C	0.000
50	C	0.000
52	C	0.000
54	C	0.000
55	C	0.000
56	C	0.000
64	C	0.000
65	C	0.000
66	C	0.000
68	C	0.000
69	C	0.000
70	C	0.000
92	H	0.000
109	H	0.000
137	H	0.000
154	H	0.000
35	C	0.001
113	H	0.001
120	H	0.001
15	C	0.002
59	C	0.002
60	C	0.002
61	C	0.002
123	H	0.002
130	H	0.002
156	H	0.002
167	H	0.002
13	C	0.003
47	C	0.003
93	H	0.003
105	H	0.003
118	H	0.003
124	H	0.003
146	H	0.003
14	C	0.004
36	C	0.004
44	C	0.004
74	N	0.004

76	N	0.004
84	H	0.004
86	H	0.004
87	H	0.004
107	H	0.004
126	H	0.004
139	H	0.004
142	H	0.004
164	H	0.004
37	C	0.005
83	H	0.005
103	H	0.005
114	H	0.005
140	H	0.005
25	C	0.006
134	H	0.006
136	H	0.006
151	H	0.006
153	H	0.006
21	C	0.007
81	H	0.007
89	H	0.007
90	H	0.007
97	H	0.007
112	H	0.007
127	H	0.007
170	H	0.007
45	C	0.008
73	N	0.008
88	H	0.008
104	H	0.008
108	H	0.008
111	H	0.008
121	H	0.008
128	H	0.008
133	H	0.008
152	H	0.008
155	H	0.008
5	O	0.009
46	C	0.009
75	N	0.009
82	H	0.009
94	H	0.009
117	H	0.009
125	H	0.009
131	H	0.009
138	H	0.009
144	H	0.009
148	H	0.009
115	H	0.010
141	H	0.010
91	H	0.011

161	H	0.011
162	H	0.011
20	C	0.012
99	H	0.012
9	N	0.013
79	H	0.013
102	H	0.013
168	H	0.013
169	H	0.013
171	H	0.013
100	H	0.015
163	H	0.015
165	H	0.015
160	H	0.016
10	C	0.017
95	H	0.017
166	H	0.020
96	H	0.022
157	H	0.022
158	H	0.027

5•6$_O$(f)···Na$^+$

Number	Atom	VDD
6	O	–0.095
72	O	–0.087
91	O	–0.085
1	Na	–0.077
84	O	–0.073
2	O	–0.060
4	O	–0.051
132	H	–0.036
145	H	–0.020
109	H	–0.019
113	H	–0.018
3	O	–0.016
48	C	–0.011
23	C	–0.010
57	C	–0.009
161	H	–0.008
28	C	–0.007
24	C	–0.006
29	C	–0.006
43	C	–0.006
96	H	–0.006
101	H	–0.006
117	H	–0.006
151	H	–0.006
39	C	–0.005
58	C	–0.005
122	H	–0.005
148	H	–0.005
31	C	–0.004

11	C	–0.003
27	C	–0.003
30	C	–0.003
34	C	–0.003
86	N	–0.003
7	N	–0.002
8	N	–0.002
16	C	–0.002
33	C	–0.002
126	H	–0.002
135	H	–0.002
146	H	–0.002
159	H	–0.002
162	H	–0.002
165	H	–0.002
170	H	–0.002
12	C	–0.001
19	C	–0.001
38	C	–0.001
40	C	–0.001
49	C	–0.001
51	C	–0.001
53	C	–0.001
63	C	–0.001
67	C	–0.001
74	N	–0.001
80	N	–0.001
92	N	–0.001
138	H	–0.001
17	C	0.000
18	C	0.000
32	C	0.000
41	C	0.000
42	C	0.000
50	C	0.000
52	C	0.000
54	C	0.000
55	C	0.000
56	C	0.000
64	C	0.000
65	C	0.000
66	C	0.000
68	C	0.000
69	C	0.000
70	C	0.000
155	H	0.000
169	H	0.000
26	C	0.001
59	C	0.001
62	C	0.001
77	N	0.001
121	H	0.001

125	H	0.001
129	H	0.001
136	H	0.001
167	H	0.001
15	C	0.002
47	C	0.002
114	H	0.002
123	H	0.002
134	H	0.002
153	H	0.002
13	C	0.003
35	C	0.003
37	C	0.003
103	H	0.003
139	H	0.003
140	H	0.003
163	H	0.003
166	H	0.003
14	C	0.004
25	C	0.004
36	C	0.004
44	C	0.004
100	H	0.004
102	H	0.004
142	H	0.004
22	C	0.005
60	C	0.005
61	C	0.005
110	H	0.005
5	O	0.006
83	N	0.006
97	H	0.006
99	H	0.006
119	H	0.006
130	H	0.006
156	H	0.006
158	H	0.006
71	N	0.007
75	O	0.007
88	O	0.007
89	N	0.007
94	O	0.007
104	H	0.007
105	H	0.007
106	H	0.007
108	H	0.007
127	H	0.007
128	H	0.007
152	H	0.007
154	H	0.007
157	H	0.007
160	H	0.007

21	C	0.008
46	C	0.008
98	H	0.008
118	H	0.008
120	H	0.008
124	H	0.008
137	H	0.008
143	H	0.008
144	H	0.008
149	H	0.008
164	H	0.008
168	H	0.008
171	H	0.008
45	C	0.009
112	H	0.009
133	H	0.009
141	H	0.009
150	H	0.009
10	C	0.010
131	H	0.010
147	H	0.010
20	C	0.011
95	H	0.011
9	N	0.013
79	O	0.013
111	H	0.013
78	O	0.014
93	O	0.014
107	H	0.014
82	O	0.015
116	H	0.015
115	H	0.016
81	O	0.018
76	O	0.022
87	O	0.022
90	O	0.045
85	O	0.054
73	O	0.057

Table S12. Optimized Cartesian coordinates for the compounds investigated in this study using the BLYP–D3(BJ)/Def2–TZVP computational model.

1•4$_{ac}$···Zn^{2+}

Zn	-0.213988000	0.491807000	-2.975729000
N	1.076967000	-0.299515000	-4.314689000
N	-1.248523000	1.017780000	-4.628327000
N	-0.648015000	0.135649000	-1.105695000
N	-1.742436000	-0.348245000	-0.517153000
N	-1.423692000	-0.445034000	0.786132000
C	-2.440836000	1.650173000	-4.687076000
C	-3.067950000	1.925902000	-5.899707000
C	-2.438317000	1.528325000	-7.082499000
C	-1.206392000	0.870964000	-7.019356000
C	-0.618445000	0.621722000	-5.774970000
C	0.691608000	-0.064967000	-5.601973000
C	1.522094000	-0.442290000	-6.662349000
C	2.747210000	-1.058398000	-6.390215000
C	3.127280000	-1.281310000	-5.063969000
C	2.262863000	-0.883445000	-4.046918000
C	-0.136821000	-0.022475000	1.029337000
C	0.364932000	0.366508000	-0.197407000
C	1.626966000	0.896287000	-0.733019000
C	1.535088000	1.934704000	-1.704467000
C	2.670751000	2.352890000	-2.417727000
C	3.894285000	1.724835000	-2.114418000
C	4.026488000	0.730020000	-1.132801000
C	2.862046000	0.313348000	-0.451213000
C	2.620093000	3.471193000	-3.472181000
C	1.188508000	4.000273000	-3.704841000
C	3.163054000	2.924763000	-4.820063000
C	3.509491000	4.652028000	-2.994180000
C	5.372682000	0.056601000	-0.817281000
C	6.550239000	0.715521000	-1.566955000
C	5.646487000	0.146717000	0.708434000
C	5.289831000	-1.435624000	-1.243056000
C	-2.374033000	-0.950466000	1.745189000
C	-3.273149000	-1.941284000	1.342829000
C	-4.188614000	-2.447726000	2.275302000
C	-4.148025000	-1.927350000	3.581156000

C	-3.252058000	-0.925747000	3.994426000
C	-2.348752000	-0.431376000	3.043446000
C	-5.199999000	-3.552251000	1.919865000
C	-5.124828000	-3.954400000	0.431421000
C	-6.636547000	-3.045044000	2.216674000
C	-4.898545000	-4.805261000	2.785788000
C	-3.300537000	-0.398145000	5.439411000
C	-4.703033000	0.213917000	5.702764000
C	-3.051372000	-1.576646000	6.418393000
C	-2.235942000	0.688462000	5.701584000
H	-2.887720000	1.934601000	-3.737402000
H	-4.026704000	2.437035000	-5.910143000
H	-2.900632000	1.724186000	-8.047289000
H	-0.717952000	0.552360000	-7.934631000
H	1.229551000	-0.252972000	-7.690074000
H	3.398398000	-1.355089000	-7.209228000
H	4.074021000	-1.753118000	-4.816240000
H	2.510506000	-1.017625000	-2.997671000
H	0.299775000	-0.038704000	2.016476000
H	0.614445000	2.517829000	-1.763484000
H	4.781500000	2.042727000	-2.650400000
H	2.911534000	-0.491354000	0.277692000
H	0.509813000	3.211463000	-4.061403000
H	1.206031000	4.780403000	-4.473589000
H	0.758918000	4.446346000	-2.799085000
H	4.192907000	2.562975000	-4.731909000
H	3.153459000	3.719100000	-5.575595000
H	2.542107000	2.097638000	-5.188409000
H	3.151219000	5.049010000	-2.036813000
H	3.485905000	5.462754000	-3.732064000
H	4.552758000	4.344138000	-2.865497000
H	6.446380000	0.630485000	-2.656623000
H	7.483446000	0.213234000	-1.289902000
H	6.657235000	1.776054000	-1.307513000
H	5.679053000	1.191761000	1.039273000
H	6.614846000	-0.312822000	0.937958000
H	4.885094000	-0.377163000	1.298102000
H	4.487694000	-1.969855000	-0.719850000
H	6.233708000	-1.943942000	-1.013688000
H	5.118841000	-1.523661000	-2.324919000

H	-3.237889000	-2.313370000	0.325635000
H	-4.848299000	-2.320939000	4.311622000
H	-1.661089000	0.368630000	3.297576000
H	-5.340321000	-3.105184000	-0.230694000
H	-5.870338000	-4.730880000	0.226024000
H	-4.141698000	-4.366201000	0.167864000
H	-6.774023000	-2.793852000	3.274337000
H	-7.365560000	-3.824415000	1.963458000
H	-6.869490000	-2.152408000	1.622405000
H	-3.883876000	-5.177521000	2.595966000
H	-5.608246000	-5.605878000	2.544478000
H	-4.986998000	-4.591108000	3.856959000
H	-4.905371000	1.039698000	5.009072000
H	-4.753241000	0.605600000	6.725795000
H	-5.500824000	-0.529001000	5.589189000
H	-3.802638000	-2.366782000	6.308418000
H	-3.095946000	-1.216815000	7.453408000
H	-2.062425000	-2.022808000	6.253167000
H	-1.217157000	0.307348000	5.549510000
H	-2.306646000	1.025455000	6.741871000
H	-2.383753000	1.568918000	5.062086000

2•4$_{ac}$···Zn^{2+}

N	-2.087826000	-0.767221000	-1.961102000
N	-1.865246000	1.915067000	-1.990976000
C	-2.075224000	-2.113978000	-1.980319000
C	-3.101709000	-2.853635000	-2.562975000
C	-4.163206000	-2.168461000	-3.157872000
C	-4.169409000	-0.772098000	-3.147823000
C	-3.117278000	-0.082493000	-2.535418000
C	-3.028302000	1.401062000	-2.480691000
C	-4.057803000	2.255328000	-2.893225000
C	-3.882517000	3.638029000	-2.805672000
C	-2.683597000	4.147641000	-2.299743000
C	-1.698321000	3.249303000	-1.896176000
N	1.335067000	-0.212180000	-1.556980000
N	0.136137000	0.963709000	0.523692000
N	-0.320140000	1.577957000	1.603894000
N	0.729678000	1.646993000	2.458671000
C	1.852909000	1.082751000	1.913650000

C	1.466700000	0.647670000	0.656119000
C	2.124529000	-0.048438000	-0.450564000
C	3.418734000	-0.564872000	-0.404942000
C	3.894536000	-1.281210000	-1.509318000
C	3.073403000	-1.465518000	-2.620582000
C	1.781696000	-0.911145000	-2.632225000
C	0.859873000	-1.067483000	-3.778003000
C	0.244927000	0.058506000	-4.357141000
C	-0.614679000	-0.088741000	-5.457213000
C	-0.873100000	-1.393797000	-5.910269000
C	-0.285461000	-2.540194000	-5.348962000
C	0.603796000	-2.353107000	-4.280390000
C	-1.197145000	1.109832000	-6.231842000
C	-0.586069000	1.109700000	-7.660705000
C	-0.864101000	2.460768000	-5.564935000
C	-2.738052000	0.977105000	-6.337061000
C	-0.575530000	-3.926922000	-5.952629000
C	0.034956000	-3.967895000	-7.380982000
C	0.040141000	-5.069303000	-5.116836000
C	-2.105876000	-4.159678000	-6.041128000
C	0.589180000	2.238875000	3.764804000
C	-0.633903000	2.118368000	4.429024000
C	-0.769688000	2.670628000	5.710113000
C	0.348633000	3.316060000	6.266952000
C	1.582508000	3.446341000	5.605900000
C	1.691345000	2.895114000	4.321686000
C	-2.079055000	2.573835000	6.513864000
C	-3.191656000	1.844884000	5.731028000
C	-1.807956000	1.792061000	7.827052000
C	-2.573727000	4.006443000	6.850184000
C	2.751084000	4.175816000	6.293343000
C	3.111976000	3.418082000	7.599786000
C	4.007816000	4.239886000	5.399335000
C	2.317190000	5.626406000	6.634729000
Zn	-0.588372000	0.464121000	-1.327223000
H	-1.218991000	-2.597682000	-1.517515000
H	-3.062259000	-3.938905000	-2.550837000
H	-4.976001000	-2.713035000	-3.632478000
H	-4.981237000	-0.231594000	-3.623058000
H	-4.992061000	1.851739000	-3.269819000

H	-4.677112000	4.308609000	-3.124534000
H	-2.512293000	5.217094000	-2.214196000
H	-0.749883000	3.591257000	-1.488676000
H	2.782975000	1.016159000	2.457667000
H	4.034531000	-0.426449000	0.479519000
H	4.901687000	-1.692000000	-1.498583000
H	3.424727000	-2.008355000	-3.493110000
H	0.512715000	1.049996000	-4.000587000
H	-1.530770000	-1.520877000	-6.765385000
H	1.090510000	-3.204919000	-3.815302000
H	0.506752000	1.190893000	-7.616702000
H	-0.971117000	1.961213000	-8.235018000
H	-0.835637000	0.193866000	-8.208397000
H	-1.259605000	2.519275000	-4.544914000
H	-1.311771000	3.276829000	-6.143298000
H	0.216965000	2.642910000	-5.528321000
H	-3.036900000	0.047613000	-6.833670000
H	-3.148303000	1.811105000	-6.919136000
H	-3.203938000	0.994822000	-5.344160000
H	-0.411321000	-3.206526000	-8.031540000
H	-0.141175000	-4.948847000	-7.838859000
H	1.117573000	-3.794778000	-7.347846000
H	1.135011000	-5.010739000	-5.081702000
H	-0.218310000	-6.034125000	-5.567145000
H	-0.342205000	-5.072032000	-4.086778000
H	-2.565404000	-4.129897000	-5.045107000
H	-2.308940000	-5.145217000	-6.476461000
H	-2.604594000	-3.412485000	-6.668916000
H	-1.449878000	1.587749000	3.952599000
H	0.254424000	3.737211000	7.263351000
H	2.608169000	3.001918000	3.751034000
H	-3.436008000	2.361039000	4.792985000
H	-4.103360000	1.813967000	6.338284000
H	-2.916161000	0.807399000	5.500165000
H	-1.450567000	0.777153000	7.611706000
H	-2.731354000	1.712213000	8.413466000
H	-1.058440000	2.291273000	8.451186000
H	-1.850556000	4.556362000	7.463087000
H	-3.514228000	3.954605000	7.412324000
H	-2.753021000	4.584618000	5.934753000

H	2.265874000	3.380482000	8.294981000
H	3.940808000	3.923870000	8.109639000
H	3.420379000	2.387187000	7.384480000
H	4.382238000	3.238673000	5.147088000
H	4.809933000	4.762096000	5.932763000
H	3.819814000	4.791736000	4.468628000
H	2.047891000	6.179654000	5.726170000
H	3.141664000	6.156364000	7.126745000
H	1.456686000	5.647033000	7.313096000

3•4$_{ac}$···Zn^{2+}

C	-0.047063000	2.977210000	-0.277839000
C	-0.387288000	4.325823000	-0.449173000
C	-1.730341000	4.706241000	-0.434508000
C	-2.715922000	3.733703000	-0.248455000
C	-2.315291000	2.408875000	-0.084001000
C	2.774622000	0.620348000	-0.100684000
C	3.915795000	1.404975000	-0.257384000
C	3.754504000	2.782125000	-0.429722000
C	2.468499000	3.325036000	-0.440621000
C	1.359179000	2.483695000	-0.279454000
N	-1.020187000	2.041485000	-0.098477000
N	1.534054000	1.143656000	-0.111504000
Zn	-0.257596000	0.126062000	0.116636000
H	0.381693000	5.077262000	-0.594740000
H	-2.001717000	5.751020000	-0.567539000
H	-3.771568000	3.990485000	-0.231004000
H	-3.042584000	1.613290000	0.063503000
H	2.844688000	-0.456585000	0.037638000
H	4.900179000	0.945150000	-0.243351000
H	4.619644000	3.429434000	-0.554100000
H	2.338832000	4.394312000	-0.571581000
C	-1.983354000	-3.017203000	-9.518951000
C	-3.749360000	-2.922384000	-7.736175000
C	-2.704483000	-2.087942000	-8.506757000
C	0.754201000	2.010349000	-8.728828000
C	1.440913000	0.825859000	-7.996274000
C	2.447506000	1.399919000	-6.975988000
C	0.355662000	-0.033600000	-7.322459000
C	-0.622324000	-0.669885000	-8.106613000

C	-1.658410000	-1.454704000	-7.571565000
C	-1.703019000	-1.609770000	-6.178864000
C	-0.726807000	-0.993022000	-5.391086000
C	-1.162715000	-2.290852000	-3.271890000
C	-1.023729000	-1.951244000	-1.934521000
C	-1.248551000	-2.635465000	-0.660843000
C	-1.710733000	-3.947880000	-0.498493000
C	-1.868842000	-4.443042000	0.800062000
C	-1.570254000	-3.637793000	1.904116000
C	-1.111341000	-2.334216000	1.675247000
C	-0.753094000	-1.353153000	2.699942000
C	-0.727445000	-1.344527000	4.086317000
C	0.294691000	-0.209877000	-5.933226000
C	-0.080753000	0.441968000	5.746174000
C	-0.919986000	0.016316000	6.779860000
C	-0.720298000	0.513520000	8.075483000
C	0.320350000	1.439195000	8.263710000
C	1.164921000	1.880238000	7.230010000
C	0.953192000	1.359812000	5.945970000
C	2.289329000	2.886259000	7.536154000
C	3.094211000	3.265396000	6.274453000
C	3.263214000	2.257030000	8.568756000
C	-1.606090000	0.097074000	9.263639000
C	-2.642393000	-0.978259000	8.872270000
C	-0.709351000	-0.480190000	10.391160000
C	-2.364721000	1.346145000	9.786994000
C	2.221997000	-0.036054000	-9.022582000
C	-3.444750000	-0.957907000	-9.272382000
N	-0.774625000	-1.176695000	-3.963287000
N	-0.406421000	-0.178641000	-3.112893000
N	-0.556049000	-0.660187000	-1.895338000
N	-0.961559000	-1.866044000	0.414599000
N	-0.322319000	-0.117158000	2.283090000
N	-0.042324000	0.653825000	3.314733000
N	-0.286878000	-0.095071000	4.425835000
C	1.668644000	4.179751000	8.127999000
H	-1.445012000	-3.821227000	-9.001162000
H	-1.261974000	-2.467969000	-10.134665000
H	-2.716452000	-3.475384000	-10.193889000
H	-4.460453000	-3.362972000	-8.444071000

H	-4.326245000	-2.307966000	-7.032053000
H	-3.283811000	-3.749542000	-7.183414000
H	0.196076000	2.637242000	-8.021784000
H	0.054618000	1.663099000	-9.497808000
H	1.509329000	2.635354000	-9.221376000
H	3.198009000	2.000294000	-7.502668000
H	2.979345000	0.604714000	-6.437206000
H	1.956090000	2.052910000	-6.242703000
H	-0.578212000	-0.546019000	-9.184564000
H	-2.502270000	-2.174375000	-5.709624000
H	-1.461866000	-3.200447000	-3.771063000
H	-1.940059000	-4.565190000	-1.362752000
H	-2.226230000	-5.458976000	0.952100000
H	-1.691486000	-4.012795000	2.916714000
H	-0.945851000	-2.098603000	4.827673000
H	1.026540000	0.238786000	-5.272012000
H	-1.736166000	-0.667356000	6.570423000
H	0.480852000	1.835766000	9.261687000
H	1.579518000	1.644960000	5.108994000
H	3.873314000	3.987738000	6.543818000
H	2.458571000	3.732770000	5.510685000
H	3.592229000	2.393088000	5.831216000
H	3.707745000	1.333690000	8.176321000
H	2.758927000	2.016455000	9.511584000
H	4.074516000	2.960173000	8.792835000
H	-2.159025000	-1.894413000	8.506841000
H	-3.341161000	-0.618248000	8.105330000
H	-3.237068000	-1.249439000	9.751910000
H	-0.145964000	-1.352406000	10.036500000
H	-1.331152000	-0.795762000	11.237599000
H	0.007674000	0.259070000	10.764465000
H	-3.008107000	1.067772000	10.630786000
H	-2.997536000	1.778331000	9.001444000
H	-1.675232000	2.124446000	10.133630000
H	2.995088000	0.572522000	-9.507534000
H	1.567991000	-0.430145000	-9.808426000
H	2.712388000	-0.886057000	-8.531559000
H	-2.756373000	-0.361863000	-9.882215000
H	-3.954652000	-0.281662000	-8.574560000
H	-4.197521000	-1.389446000	-9.943165000

H	2.461061000	4.902305000	8.359610000
H	1.117343000	3.982339000	9.054368000
H	0.976743000	4.645370000	7.414599000

1•4$_c$···Zn^{2+}

C	-1.234230000	2.148973000	3.318876000
C	-2.619708000	1.945882000	3.257846000
C	-3.167998000	1.123415000	2.268851000
C	-2.320752000	0.443708000	1.384376000
N	-0.976265000	0.567378000	1.542729000
C	-0.415583000	1.452018000	2.431325000
C	1.061383000	1.671889000	2.266651000
N	1.676903000	0.818132000	1.401551000
C	2.936948000	0.981354000	0.936793000
C	3.698206000	2.040567000	1.444471000
C	3.129243000	2.891389000	2.400485000
C	1.796061000	2.727284000	2.808053000
C	-2.818903000	-0.285895000	0.143449000
C	3.366334000	0.004184000	-0.144003000
C	-3.145170000	-1.814134000	0.229197000
C	2.536823000	0.155569000	-1.477592000
N	-1.193520000	-2.482032000	-1.159407000
C	-0.416987000	-2.025916000	-2.194776000
C	0.847212000	-1.787483000	-1.655372000
N	0.767872000	-2.155856000	-0.326345000
N	-0.490470000	-2.579999000	-0.034690000
C	-2.678419000	-2.600204000	-1.021812000
C	2.101161000	-1.161160000	-2.208049000
H	-0.812026000	2.842682000	4.039777000
H	-3.272745000	2.468789000	3.953275000
H	-4.246170000	1.032777000	2.161851000
H	4.714863000	2.199665000	1.094581000
H	3.716087000	3.710462000	2.810827000
H	1.346247000	3.428651000	3.504198000
H	-2.061475000	-0.111783000	-0.634151000
H	-3.713850000	0.245445000	-0.201853000
H	3.263626000	-1.013955000	0.258102000
H	4.431106000	0.137283000	-0.357763000
H	-4.227273000	-1.962097000	0.316231000
H	-2.694669000	-2.266971000	1.117107000

H	3.137414000	0.738294000	-2.183794000
H	1.638099000	0.764080000	-1.295007000
H	-0.804490000	-1.908950000	-3.198757000
H	-3.110963000	-2.211422000	-1.948898000
H	-2.930330000	-3.662050000	-0.933094000
H	2.915876000	-1.897249000	-2.174587000
H	1.931791000	-0.930904000	-3.265591000
Zn	0.475162000	-0.615500000	0.853076000

2•4$_c$···Zn^{2+}

C	-1.150541000	3.499510000	1.818751000
C	-2.546298000	3.451491000	1.857307000
C	-3.194202000	2.249610000	1.588927000
C	-2.452323000	1.118222000	1.221128000
N	-1.094685000	1.203649000	1.111397000
C	-0.433366000	2.354445000	1.467221000
C	1.055146000	2.294641000	1.531734000
N	1.657192000	1.136862000	1.108904000
C	2.999705000	0.953701000	1.248906000
C	3.790024000	1.971371000	1.801670000
C	3.202646000	3.168589000	2.199853000
C	1.820656000	3.330971000	2.069421000
C	-3.110095000	-0.223009000	1.037220000
C	3.622394000	-0.328483000	0.770895000
C	-4.309490000	-0.265034000	0.053352000
C	4.274187000	-0.180382000	-0.644854000
C	-4.773291000	-1.715614000	-0.252806000
C	4.098927000	-1.446485000	-1.526704000
N	-3.583869000	-2.546574000	-0.513630000
C	-2.582732000	-2.225123000	-1.368233000
C	-1.443115000	-2.813362000	-0.831797000
N	-1.811315000	-3.501989000	0.314851000
N	-3.080695000	-3.321340000	0.528214000
C	-0.054457000	-2.509183000	-1.126943000
C	0.287029000	-1.200891000	-1.653145000
C	1.656069000	-0.925985000	-1.945051000
C	2.639459000	-1.832407000	-1.554997000
C	2.184875000	-3.067099000	-1.027359000
N	0.904645000	-3.401816000	-0.820966000
H	-0.636985000	4.421294000	2.068243000

H	-3.115507000	4.338400000	2.126800000
H	-4.274266000	2.171580000	1.677495000
H	4.858272000	1.807939000	1.918566000
H	3.806836000	3.965610000	2.627591000
H	1.353623000	4.247485000	2.412020000
H	-3.437979000	-0.573212000	2.028311000
H	-2.333296000	-0.926322000	0.721378000
H	2.853517000	-1.112108000	0.766072000
H	4.377954000	-0.653665000	1.495845000
H	-4.027401000	0.227964000	-0.886846000
H	-5.165615000	0.288908000	0.456623000
H	5.339423000	0.053241000	-0.541606000
H	3.824494000	0.675752000	-1.164193000
H	-5.443886000	-1.737516000	-1.117837000
H	-5.281372000	-2.176842000	0.599036000
H	4.694262000	-2.278895000	-1.132710000
H	4.476049000	-1.232210000	-2.535497000
H	-2.746165000	-1.620505000	-2.250697000
H	-0.494373000	-0.601215000	-2.129446000
H	1.919832000	0.015143000	-2.426316000
H	2.916586000	-3.832547000	-0.759077000
Zn	0.264594000	-0.051857000	0.224448000

3•4$_{c}$···Zn^{2+}

Zn	0.255125000	-0.482749000	0.780539000
C	-0.897578000	1.805173000	1.853183000
C	-2.119474000	2.208063000	1.293356000
C	-3.295872000	1.641401000	1.772434000
C	-3.243305000	0.713218000	2.836169000
N	-2.073031000	0.371115000	3.413668000
C	-0.931981000	0.877184000	2.913822000
C	0.341964000	0.324853000	3.463382000
N	1.170967000	-0.268112000	2.541843000
C	2.386040000	-0.767140000	2.911787000
C	2.779989000	-0.681302000	4.252635000
C	1.929726000	-0.120378000	5.205725000
C	0.689422000	0.388606000	4.807169000
C	-4.494593000	0.044199000	3.371892000
C	3.262989000	-1.437154000	1.879542000
C	-5.421006000	-0.609681000	2.312671000

C	4.673743000	-0.814053000	1.699115000
C	-4.903276000	-1.906138000	1.656596000
C	4.698058000	0.615819000	1.117800000
C	-2.202624000	-0.922994000	-0.571205000
C	-3.560450000	-1.149342000	-0.404800000
N	-3.678431000	-1.678928000	0.850601000
N	-1.596310000	-1.339273000	0.594325000
N	-2.483428000	-1.797557000	1.454880000
C	-1.345125000	-0.342203000	-1.609046000
C	-1.708326000	-0.138111000	-2.939729000
C	-0.743484000	0.335927000	-3.838601000
C	0.555844000	0.592950000	-3.398579000
C	0.869918000	0.394103000	-2.047159000
N	-0.076531000	-0.059874000	-1.179714000
N	3.901574000	0.675166000	-0.118768000
C	2.594826000	1.032754000	-0.239418000
C	2.212007000	0.548869000	-1.487369000
N	3.305383000	-0.054990000	-2.069395000
N	4.318247000	0.004571000	-1.239348000
H	0.035082000	2.276563000	1.548009000
H	-2.144814000	2.961248000	0.508635000
H	-4.253984000	1.932857000	1.348025000
H	3.745003000	-1.085846000	4.546754000
H	2.230254000	-0.077628000	6.250395000
H	0.000673000	0.840096000	5.515472000
H	-5.090271000	0.803902000	3.899896000
H	-4.187065000	-0.694298000	4.120545000
H	2.743453000	-1.464971000	0.909669000
H	3.400420000	-2.486420000	2.175857000
H	-5.691651000	0.112541000	1.530892000
H	-6.361891000	-0.881560000	2.808158000
H	5.242615000	-1.470921000	1.031466000
H	5.207078000	-0.798306000	2.658106000
H	-5.658510000	-2.332827000	0.988784000
H	-4.630842000	-2.656291000	2.405033000
H	5.724460000	0.910688000	0.879847000
H	4.276199000	1.353758000	1.808938000
H	-4.415489000	-0.968058000	-1.041291000
H	-2.712663000	-0.380104000	-3.277133000
H	-1.004768000	0.489113000	-4.883331000

H	1.328847000	0.942096000	-4.077132000
H	2.100901000	1.648686000	0.498327000

1•4····Zn^{2+}

Zn	6.904939000	11.895618000	18.751338000
O	8.188900000	9.386404000	25.846924000
O	6.363781000	14.027412000	25.637602000
N	6.980171000	10.427039000	17.352814000
N	5.321088000	12.572028000	17.657708000
N	6.423284000	11.742637000	20.658695000
N	5.314979000	11.197629000	21.139967000
N	5.484246000	11.176889000	22.479249000
C	4.590488000	13.695497000	17.872739000
C	3.375310000	13.867105000	17.195709000
H	2.785154000	14.762063000	17.364975000
C	2.938220000	12.891220000	16.301851000
H	1.997364000	13.016393000	15.770553000
C	3.716585000	11.752344000	16.081131000
H	3.381272000	10.995626000	15.380392000
C	4.919512000	11.610705000	16.778114000
C	5.827712000	10.437781000	16.611886000
C	5.544869000	9.389360000	15.732729000
H	4.634268000	9.391188000	15.144031000
C	6.448398000	8.331874000	15.616621000
H	6.236738000	7.504572000	14.942921000
C	7.627806000	8.355429000	16.357657000
H	8.357711000	7.555747000	16.266879000
C	7.891362000	9.426667000	17.221681000
C	9.206550000	9.526656000	17.958166000
H	9.733157000	10.413258000	17.572590000
H	9.817814000	8.659817000	17.681267000
C	9.133183000	9.633411000	19.493870000
H	10.146060000	9.844629000	19.856056000
H	8.550709000	10.515139000	19.785457000
C	8.558404000	8.400747000	20.218953000
H	7.547239000	8.185770000	19.844945000
H	9.174366000	7.522512000	19.969332000
C	8.499784000	8.599382000	21.725587000
C	9.635106000	8.995913000	22.447910000
H	10.589400000	9.115983000	21.937066000

C	9.584386000	9.250945000	23.821402000
H	10.490377000	9.539787000	24.342853000
C	8.371007000	9.110265000	24.514145000
C	7.240616000	8.644452000	23.819930000
H	6.314326000	8.497124000	24.367886000
C	7.309604000	8.404603000	22.447549000
H	6.413224000	8.067242000	21.927756000
C	9.204293000	10.125260000	26.580766000
H	10.186642000	9.652130000	26.453220000
H	8.904870000	10.001824000	27.626753000
C	9.244232000	11.612747000	26.195792000
H	10.026283000	12.097480000	26.797130000
H	9.562147000	11.704728000	25.149471000
C	7.885817000	12.308432000	26.386129000
H	7.105361000	11.651697000	25.983666000
H	7.656623000	12.426504000	27.452977000
C	7.770147000	13.664378000	25.677406000
H	8.319035000	14.454880000	26.205613000
H	8.167210000	13.605628000	24.654192000
C	5.766750000	14.325501000	24.431744000
C	4.406452000	13.986299000	24.320037000
H	3.901462000	13.549100000	25.175380000
C	3.731982000	14.188871000	23.122936000
H	2.683260000	13.900524000	23.055427000
C	4.365603000	14.760226000	22.001862000
C	5.706300000	15.142564000	22.147625000
H	6.230532000	15.640353000	21.334246000
C	6.409464000	14.934301000	23.345868000
H	7.444537000	15.257324000	23.422275000
C	3.530327000	14.974293000	20.742737000
H	2.652284000	15.574499000	21.019654000
H	3.126032000	14.000519000	20.418963000
C	4.222927000	15.660532000	19.553751000
H	3.464917000	16.066238000	18.872698000
H	4.786311000	16.533276000	19.906681000
C	5.191771000	14.747544000	18.778652000
H	5.843418000	15.367833000	18.143513000
H	5.848697000	14.244294000	19.497189000
C	6.699389000	11.701504000	22.835476000
H	7.034231000	11.735507000	23.854917000

C	7.306242000	12.089599000	21.652128000
C	8.595383000	12.658583000	21.251327000
C	8.679331000	13.303706000	19.984019000
H	7.778830000	13.761207000	19.565531000
C	9.921697000	13.617724000	19.403368000
C	11.069530000	13.350903000	20.164925000
H	12.038534000	13.594989000	19.742390000
C	11.020915000	12.824723000	21.466465000
C	9.765396000	12.449393000	21.985070000
H	9.698466000	11.941954000	22.940190000
C	10.058970000	14.278989000	18.021306000
C	8.713867000	14.381596000	17.276076000
H	8.264078000	13.389960000	17.100057000
H	8.868423000	14.834439000	16.290497000
H	7.991780000	15.006866000	17.812639000
C	11.032642000	13.447278000	17.145313000
H	12.031728000	13.380725000	17.587554000
H	11.138040000	13.912456000	16.158011000
H	10.657439000	12.424959000	17.001105000
C	10.622188000	15.715543000	18.208984000
H	9.950562000	16.321228000	18.830135000
H	10.730340000	16.209283000	17.235873000
H	11.605175000	15.697748000	18.691904000
C	12.332121000	12.611005000	22.242074000
C	13.212234000	13.886916000	22.164274000
H	13.522071000	14.120873000	21.139913000
H	14.123594000	13.738812000	22.755154000
H	12.680734000	14.757235000	22.568697000
C	12.080400000	12.292342000	23.729804000
H	11.517011000	13.093729000	24.223835000
H	13.038465000	12.186793000	24.250767000
H	11.533214000	11.352554000	23.853726000
C	13.087722000	11.418254000	21.593718000
H	12.489552000	10.499770000	21.648922000
H	14.032810000	11.240524000	22.120899000
H	13.319906000	11.613588000	20.540046000
C	4.481455000	10.579275000	23.320902000
C	3.462480000	9.830289000	22.717034000
H	3.448277000	9.728783000	21.639926000
C	2.491132000	9.223890000	23.516722000

C	2.587400000	9.385073000	24.914866000
H	1.837368000	8.911191000	25.537457000
C	3.605895000	10.121652000	25.531961000
C	4.562855000	10.734223000	24.702314000
H	5.351307000	11.332734000	25.141197000
C	1.334354000	8.400336000	22.922771000
C	1.434788000	8.274722000	21.387379000
H	1.386828000	9.254652000	20.893927000
H	0.596083000	7.676196000	21.013750000
H	2.362669000	7.773348000	21.081035000
C	-0.006303000	9.101567000	23.272500000
H	-0.151862000	9.180827000	24.355725000
H	-0.848337000	8.531231000	22.861361000
H	-0.038344000	10.114514000	22.851327000
C	1.349054000	6.972801000	23.531216000
H	2.294609000	6.460937000	23.310952000
H	0.530381000	6.377334000	23.108955000
H	1.218809000	6.990782000	24.618737000
C	3.728015000	10.248083000	27.062764000
C	2.496398000	9.673161000	27.795312000
H	1.570032000	10.175548000	27.489292000
H	2.613991000	9.820726000	28.874953000
H	2.381307000	8.595661000	27.625287000
C	4.984899000	9.457350000	27.517177000
H	4.885777000	8.393174000	27.268351000
H	5.106731000	9.540753000	28.604550000
H	5.899278000	9.826316000	27.040145000
C	3.876016000	11.738592000	27.461900000
H	4.766589000	12.205250000	27.027604000
H	3.957824000	11.824997000	28.552099000
H	2.997381000	12.316728000	27.146833000

2•4····Zn^{2+}

O	10.006895000	14.692799000	11.105126000
O	8.304012000	11.390529000	12.940447000
N	3.008025000	11.378308000	7.000067000
N	3.332261000	14.053453000	6.700581000
C	2.997288000	10.031200000	7.119357000
C	2.008244000	9.280100000	6.466231000
H	2.013543000	8.197756000	6.561473000

C	1.037083000	9.925265000	5.708280000
H	0.272240000	9.352974000	5.188651000
C	1.054697000	11.319133000	5.611371000
H	0.309945000	11.831437000	5.012560000
C	2.058661000	12.032106000	6.268193000
C	2.182440000	13.512129000	6.199421000
C	1.188446000	14.331497000	5.659791000
H	0.262998000	13.906846000	5.286256000
C	1.388913000	15.713070000	5.625944000
H	0.623011000	16.365510000	5.212896000
C	2.572864000	16.244548000	6.129840000
H	2.754797000	17.315840000	6.112486000
C	3.544358000	15.392273000	6.676410000
C	4.851894000	15.933210000	7.190475000
H	5.156034000	15.394625000	8.095332000
H	4.700419000	16.980756000	7.479074000
C	5.972384000	15.863501000	6.120908000
H	5.583630000	16.260991000	5.173961000
H	6.237485000	14.812552000	5.938307000
C	7.240581000	16.661841000	6.503233000
H	7.908649000	16.665401000	5.632099000
H	6.954850000	17.708665000	6.678454000
C	7.992618000	16.132668000	7.706801000
C	7.694204000	16.573537000	9.010121000
H	6.918057000	17.322164000	9.163400000
C	8.397388000	16.101377000	10.114319000
H	8.172962000	16.465756000	11.112854000
C	9.430090000	15.161503000	9.945574000
C	9.769191000	14.735983000	8.652842000
H	10.598037000	14.055779000	8.485175000
C	9.043195000	15.217703000	7.555644000
H	9.326575000	14.887898000	6.556322000
C	11.149160000	13.802185000	11.016202000
H	11.997291000	14.342654000	10.572947000
H	10.902376000	12.960560000	10.350967000
C	11.489480000	13.311703000	12.424117000
H	11.871647000	14.155353000	13.012588000
H	12.320542000	12.597372000	12.329980000
C	10.305849000	12.682225000	13.187875000
H	10.628737000	12.416404000	14.201398000

H	9.516229000	13.432931000	13.301333000
C	9.682678000	11.460205000	12.507175000
H	9.701992000	11.560656000	11.411031000
H	10.204796000	10.526736000	12.762878000
C	7.426085000	10.590920000	12.247037000
C	7.780807000	9.709692000	11.216423000
H	8.819744000	9.558644000	10.939609000
C	6.779927000	8.969157000	10.567194000
H	7.073217000	8.258744000	9.794459000
C	5.431295000	9.080104000	10.924234000
C	5.099637000	9.979253000	11.959293000
H	4.061547000	10.074882000	12.277216000
C	6.074081000	10.719916000	12.616794000
H	5.816830000	11.398239000	13.426780000
C	4.357603000	8.213750000	10.295714000
H	3.768808000	7.745384000	11.096527000
H	4.823579000	7.390846000	9.735953000
C	3.373132000	8.963249000	9.369412000
H	2.503455000	8.320951000	9.175471000
H	2.993641000	9.862592000	9.874914000
C	4.007061000	9.368907000	8.015226000
H	4.862912000	10.031518000	8.188382000
H	4.393490000	8.470821000	7.514825000
N	6.434812000	11.980983000	7.280637000
N	5.236177000	13.091044000	9.410722000
N	4.783496000	13.651730000	10.517674000
N	5.864226000	13.765914000	11.333522000
C	6.995534000	13.289064000	10.735295000
H	7.946888000	13.282055000	11.239719000
C	6.587542000	12.850769000	9.488264000
C	7.235106000	12.172901000	8.372620000
C	8.532835000	11.664585000	8.414300000
H	9.142197000	11.823933000	9.295610000
C	9.004715000	10.934332000	7.323163000
H	10.014614000	10.529874000	7.330284000
C	8.169859000	10.715150000	6.226912000
H	8.511391000	10.148809000	5.365575000
C	6.871296000	11.249543000	6.222651000
C	5.929485000	11.002023000	5.106309000
C	5.308421000	12.065813000	4.429472000

H	5.556151000	13.084963000	4.708369000
C	4.450495000	11.816845000	3.346565000
C	4.193776000	10.476236000	3.013624000
H	3.532148000	10.270869000	2.176791000
C	4.785350000	9.388348000	3.677337000
C	5.676149000	9.675566000	4.721036000
H	6.167868000	8.872569000	5.261099000
C	3.871395000	12.940422000	2.464360000
C	4.514177000	12.828398000	1.054175000
H	5.605363000	12.921533000	1.115886000
H	4.137439000	13.626154000	0.401906000
H	4.283777000	11.867375000	0.580781000
C	4.178986000	14.343460000	3.025933000
H	3.767153000	14.477950000	4.031557000
H	3.732157000	15.105781000	2.377525000
H	5.257461000	14.538317000	3.064643000
C	2.334402000	12.787531000	2.337556000
H	2.052686000	11.825336000	1.896409000
H	1.929350000	13.578405000	1.694772000
H	1.850962000	12.865236000	3.318121000
C	4.506917000	7.950500000	3.200277000
C	5.136289000	7.779780000	1.789581000
H	4.690856000	8.470850000	1.064418000
H	4.975117000	6.757483000	1.426671000
H	6.216721000	7.967305000	1.818628000
C	5.117502000	6.891167000	4.143002000
H	6.211421000	6.959291000	4.186443000
H	4.869873000	5.888438000	3.776673000
H	4.722741000	6.977677000	5.164898000
C	2.980166000	7.697197000	3.111803000
H	2.509063000	7.782532000	4.098747000
H	2.790959000	6.685307000	2.734162000
H	2.482012000	8.400559000	2.435182000
C	5.731017000	14.262859000	12.675466000
C	4.520263000	14.067944000	13.345773000
H	3.705762000	13.569236000	12.834724000
C	4.397484000	14.502205000	14.671402000
C	5.510319000	15.126279000	15.264580000
H	5.424047000	15.459059000	16.294031000
C	6.726883000	15.337926000	14.596103000

C	6.829587000	14.882933000	13.273443000
H	7.748097000	15.012159000	12.714438000
C	3.104738000	14.306840000	15.483680000
C	2.008498000	13.583454000	14.672798000
H	1.720218000	14.151082000	13.778320000
H	1.112442000	13.469357000	15.293558000
H	2.325675000	12.578379000	14.363930000
C	3.419205000	13.460671000	16.746223000
H	3.812670000	12.474007000	16.469871000
H	2.505713000	13.311828000	17.334865000
H	4.155200000	13.951589000	17.392358000
C	2.561984000	15.697213000	15.911328000
H	3.280248000	16.239803000	16.536569000
H	1.638411000	15.576039000	16.490595000
H	2.338163000	16.317175000	15.033913000
C	7.911853000	16.004596000	15.319480000
C	8.531014000	14.958277000	16.286238000
H	7.798948000	14.627283000	17.032219000
H	9.386508000	15.392960000	16.818168000
H	8.876860000	14.073412000	15.737912000
C	8.998985000	16.484110000	14.330997000
H	9.456065000	15.660178000	13.771793000
H	9.801008000	16.983101000	14.886907000
H	8.595218000	17.206557000	13.609093000
C	7.431387000	17.234945000	16.131656000
H	6.943439000	17.971604000	15.481094000
H	8.290411000	17.719449000	16.610874000
H	6.727636000	16.960872000	16.925259000
Zn	4.503974000	12.629485000	7.573461000

3•4····Zn^{2+}

C	10.184795000	10.897276000	9.245082000
C	9.343829000	11.981692000	9.508197000
H	9.751570000	12.959318000	9.742148000
C	7.959598000	11.791816000	9.476256000
H	7.290460000	12.622333000	9.689762000
C	7.447876000	10.535589000	9.163431000
H	6.375205000	10.365269000	9.116017000
C	8.329621000	9.474014000	8.905319000
C	7.823724000	8.122271000	8.477738000

H	8.591716000	7.359325000	8.660309000
H	6.946566000	7.852408000	9.081763000
C	7.427403000	8.128413000	6.979283000
H	8.320692000	8.331197000	6.373052000
H	6.732139000	8.962660000	6.810644000
C	6.744349000	6.823803000	6.504633000
H	5.923088000	6.584831000	7.196750000
H	6.276861000	7.028480000	5.533256000
C	7.654616000	5.621137000	6.354133000
C	8.087199000	4.863925000	7.460483000
H	7.757734000	5.133095000	8.463114000
C	8.882220000	3.730869000	7.300256000
H	9.181546000	3.130130000	8.155549000
C	9.270921000	3.313726000	6.015439000
C	8.843086000	4.048781000	4.898295000
H	9.112992000	3.753265000	3.889583000
C	8.048372000	5.186679000	5.081031000
H	7.720568000	5.738654000	4.201939000
C	11.430372000	2.069281000	3.889756000
H	11.273509000	3.137207000	3.692334000
H	11.430655000	1.577166000	2.906052000
C	12.790167000	1.850278000	4.574494000
H	12.740196000	2.191192000	5.616832000
H	13.011787000	0.773087000	4.612711000
C	13.953404000	2.541644000	3.858321000
H	13.945866000	2.300545000	2.789147000
H	14.922774000	2.235574000	4.270100000
C	14.445714000	4.678341000	4.940149000
C	14.856821000	4.096235000	6.148101000
H	14.754673000	3.030019000	6.322540000
C	15.433556000	4.897933000	7.140930000
H	15.762544000	4.426608000	8.066808000
C	15.624342000	6.274757000	6.967260000
C	15.189827000	6.840889000	5.753603000
H	15.331978000	7.903547000	5.564515000
C	14.607785000	6.062552000	4.755159000
H	14.301819000	6.508674000	3.811995000
C	16.363686000	7.083260000	8.021630000
H	16.376836000	6.512046000	8.960889000
H	17.417103000	7.182146000	7.718851000

C	15.816722000	8.498401000	8.295447000
H	16.463928000	8.973856000	9.044073000
H	15.888945000	9.112595000	7.387168000
C	14.360976000	8.482005000	8.783920000
H	13.750286000	7.918303000	8.066149000
H	14.298235000	7.927497000	9.735325000
C	13.712759000	9.832749000	8.984556000
C	14.432113000	11.027546000	9.119447000
H	15.515729000	11.019212000	9.065962000
C	13.743262000	12.224524000	9.318358000
H	14.288853000	13.159826000	9.422328000
C	12.349495000	12.218943000	9.375494000
H	11.806512000	13.147721000	9.513790000
C	11.670338000	11.003911000	9.232532000
N	9.663663000	9.668298000	8.969682000
N	12.360699000	9.847591000	9.042708000
O	13.853006000	3.995806000	3.898075000
C	10.251671000	1.514668000	4.692935000
H	10.429745000	0.468502000	4.968349000
H	9.316472000	1.562458000	4.121076000
O	10.074373000	2.196270000	5.967136000
C	7.805390000	8.731581000	-0.057845000
H	7.510957000	7.718460000	-0.359202000
H	8.647699000	9.049916000	-0.684107000
H	6.964473000	9.400312000	-0.275115000
C	6.882983000	8.401062000	2.252801000
H	6.041356000	9.051879000	1.985533000
H	7.043705000	8.492527000	3.334265000
H	6.593444000	7.364902000	2.035412000
C	8.145859000	8.806320000	1.445821000
C	11.313974000	5.630978000	-1.237325000
H	11.387732000	6.676940000	-1.559373000
H	10.267482000	5.312910000	-1.326194000
H	11.903404000	5.028696000	-1.938321000
C	11.857998000	5.431736000	0.193187000
C	13.345457000	5.875850000	0.220965000
H	13.917738000	5.316920000	-0.529833000
H	13.805814000	5.690250000	1.198649000
H	13.438518000	6.945051000	-0.006400000
C	11.073846000	6.248314000	1.238380000

C	10.015394000	7.104893000	0.888453000
H	9.727242000	7.171619000	-0.152559000
C	9.313473000	7.878090000	1.830377000
C	9.699017000	7.784098000	3.173753000
H	9.188043000	8.346336000	3.947332000
C	10.742372000	6.930045000	3.531655000
C	11.477643000	5.689248000	5.589800000
H	11.662481000	4.755862000	5.087475000
C	11.529360000	6.062617000	6.920326000
C	11.767621000	5.359151000	8.172790000
C	12.098940000	4.004685000	8.302356000
H	12.220871000	3.385306000	7.420955000
C	12.236528000	3.474902000	9.586140000
H	12.489469000	2.424921000	9.714734000
C	12.030166000	4.283396000	10.710474000
H	12.116218000	3.875809000	11.714143000
C	11.692509000	5.627588000	10.513204000
C	11.399242000	6.605605000	11.561608000
C	11.366881000	6.580446000	12.948434000
H	11.592376000	5.814646000	13.675907000
C	11.431852000	6.159910000	2.593843000
H	12.245227000	5.513406000	2.909636000
C	10.801039000	8.360185000	14.646866000
C	10.198661000	7.546477000	15.602885000
H	9.853838000	6.552368000	15.330086000
C	10.003745000	8.038768000	16.906868000
C	10.426745000	9.346618000	17.176842000
H	10.277602000	9.743279000	18.174602000
C	11.035843000	10.179269000	16.214202000
C	11.222423000	9.665179000	14.927789000
H	11.692832000	10.247282000	14.144108000
C	11.466627000	11.605272000	16.602051000
C	12.179220000	12.334881000	15.442925000
H	12.471967000	13.339362000	15.769473000
H	11.523741000	12.449425000	14.569226000
H	13.090606000	11.808019000	15.130844000
C	12.438753000	11.539477000	17.809645000
H	13.335210000	10.957439000	17.560884000
H	11.970209000	11.080956000	18.687844000
H	12.754906000	12.551234000	18.091741000

C	9.327787000	7.143954000	17.962990000
C	7.900779000	6.774788000	17.476589000
H	7.921207000	6.225718000	16.526996000
H	7.291132000	7.675940000	17.335414000
H	7.404053000	6.138145000	18.219065000
C	10.165264000	5.850274000	18.145527000
H	10.239672000	5.276214000	17.213476000
H	9.696396000	5.203269000	18.896804000
H	11.182114000	6.086225000	18.483292000
C	9.210046000	7.843286000	19.334121000
H	8.729863000	7.165076000	20.048793000
H	8.597129000	8.751656000	19.279327000
H	10.193208000	8.110109000	19.741420000
C	11.769491000	3.924810000	0.548920000
H	12.303148000	3.329102000	-0.202085000
H	10.725693000	3.586437000	0.575200000
H	12.222722000	3.719275000	1.523733000
C	8.532844000	10.268763000	1.793276000
H	9.422783000	10.580282000	1.232572000
H	8.747634000	10.388095000	2.862621000
H	7.710422000	10.947594000	1.535148000
N	11.074181000	6.808496000	4.923580000
N	10.895362000	7.855177000	5.779715000
N	11.170655000	7.388640000	6.979058000
N	11.584022000	6.135539000	9.264031000
N	11.053112000	7.877282000	11.174872000
N	10.806431000	8.633879000	12.222322000
N	10.997726000	7.843605000	13.317223000
C	10.203059000	12.418518000	16.993072000
H	10.484660000	13.441740000	17.271312000
H	9.681191000	11.970850000	17.846217000
H	9.498115000	12.471452000	16.153580000
Zn	11.118436000	8.193516000	8.957651000

1•4····Cd^{2+}

Cd	7.156901000	12.254130000	18.673580000
O	8.109861000	9.360023000	25.910334000
O	6.301430000	13.723373000	24.956105000
N	6.623035000	10.438279000	17.404441000
N	5.342326000	12.917460000	17.482673000

N	6.420632000	11.867454000	20.756195000
N	5.316968000	11.314307000	21.218403000
N	5.571973000	11.041055000	22.519506000
C	4.813188000	14.163655000	17.535352000
C	3.617511000	14.440437000	16.860696000
H	3.190204000	15.437583000	16.902768000
C	2.995426000	13.429624000	16.129758000
H	2.073075000	13.631160000	15.589592000
C	3.562172000	12.154381000	16.087787000
H	3.081027000	11.372815000	15.510799000
C	4.750122000	11.910546000	16.788879000
C	5.399902000	10.560621000	16.816280000
C	4.776853000	9.434882000	16.261263000
H	3.800017000	9.513112000	15.797738000
C	5.417552000	8.196921000	16.315899000
H	4.939224000	7.316982000	15.891583000
C	6.674932000	8.101041000	16.909393000
H	7.203110000	7.152242000	16.951624000
C	7.271287000	9.247047000	17.450962000
C	8.641801000	9.206601000	18.082754000
H	9.212834000	10.066929000	17.701521000
H	9.165769000	8.307083000	17.738163000
C	8.653545000	9.253308000	19.631921000
H	9.669142000	9.521270000	19.945534000
H	8.009760000	10.068352000	19.983090000
C	8.226257000	7.962567000	20.368464000
H	7.217592000	7.660239000	20.055553000
H	8.906299000	7.146000000	20.081609000
C	8.243491000	8.177166000	21.875000000
C	9.421961000	8.572159000	22.523726000
H	10.353913000	8.609867000	21.963428000
C	9.437309000	8.948195000	23.870988000
H	10.372154000	9.259172000	24.328312000
C	8.246112000	8.914809000	24.616012000
C	7.081743000	8.406992000	24.012904000
H	6.172690000	8.337096000	24.603723000
C	7.084134000	8.063030000	22.660223000
H	6.155301000	7.720869000	22.206181000
C	9.158902000	10.163555000	26.518508000
H	10.129471000	9.659713000	26.420348000

H	8.891470000	10.173396000	27.580191000
C	9.216007000	11.595330000	25.955330000
H	10.047746000	12.119247000	26.446802000
H	9.474359000	11.547083000	24.888724000
C	7.890889000	12.363528000	26.133445000
H	7.068067000	11.674117000	25.915124000
H	7.759786000	12.664421000	27.180221000
C	7.725544000	13.579145000	25.203175000
H	8.117264000	14.505222000	25.646180000
H	8.245165000	13.415750000	24.248268000
C	5.844094000	14.301677000	23.792572000
C	4.491988000	14.050209000	23.493621000
H	3.899505000	13.464989000	24.191034000
C	3.936498000	14.527341000	22.313894000
H	2.890894000	14.308522000	22.099567000
C	4.682856000	15.296481000	21.398848000
C	6.018945000	15.563321000	21.727620000
H	6.626664000	16.195518000	21.082493000
C	6.608762000	15.071114000	22.906828000
H	7.645812000	15.309276000	23.127074000
C	3.960673000	15.863963000	20.181154000
H	3.353723000	16.720986000	20.510258000
H	3.234746000	15.113945000	19.831398000
C	4.819432000	16.332507000	18.994534000
H	4.177158000	16.853251000	18.272031000
H	5.544838000	17.086718000	19.326020000
C	5.604446000	15.213699000	18.284085000
H	6.303494000	15.671022000	17.567076000
H	6.219793000	14.694376000	19.029330000
C	6.844163000	11.406465000	22.864928000
H	7.237450000	11.227897000	23.847959000
C	7.396027000	11.960482000	21.723697000
C	8.702614000	12.502427000	21.331187000
C	8.733780000	13.469328000	20.275351000
H	7.848295000	14.096255000	20.151569000
C	9.966099000	13.910233000	19.727263000
C	11.140979000	13.398775000	20.288708000
H	12.091687000	13.729949000	19.894193000
C	11.144137000	12.516576000	21.385725000
C	9.904815000	12.045795000	21.870311000

H	9.872960000	11.293643000	22.651820000
C	9.996261000	14.963823000	18.602822000
C	9.303809000	14.390737000	17.335112000
H	9.783256000	13.457937000	17.011816000
H	9.359127000	15.104284000	16.504156000
H	8.232290000	14.205059000	17.510401000
C	11.435917000	15.359179000	18.203662000
H	11.984371000	15.795992000	19.046278000
H	11.398634000	16.113058000	17.409566000
H	12.007213000	14.504585000	17.821141000
C	9.253179000	16.242133000	19.074916000
H	8.207125000	16.042635000	19.328061000
H	9.268241000	16.999889000	18.282625000
H	9.738317000	16.668365000	19.960873000
C	12.457454000	12.074266000	22.051812000
C	13.699107000	12.735964000	21.415258000
H	13.822160000	12.452743000	20.362242000
H	14.596795000	12.405291000	21.949103000
H	13.661404000	13.830344000	21.482577000
C	12.406479000	12.471560000	23.552785000
H	12.291564000	13.556290000	23.668732000
H	13.339497000	12.172798000	24.044883000
H	11.579110000	11.980470000	24.075717000
C	12.607975000	10.534631000	21.933806000
H	11.811623000	10.011438000	22.470019000
H	13.564524000	10.222815000	22.369485000
H	12.592403000	10.214605000	20.884196000
C	4.579960000	10.418039000	23.349278000
C	3.728692000	9.462101000	22.783343000
H	3.823931000	9.229292000	21.729598000
C	2.791901000	8.819195000	23.597389000
C	2.749816000	9.170161000	24.964191000
H	2.027385000	8.669392000	25.599168000
C	3.591722000	10.132702000	25.536110000
C	4.523423000	10.765880000	24.694298000
H	5.177296000	11.543466000	25.070912000
C	1.831977000	7.743412000	23.058747000
C	2.022974000	7.493736000	21.547414000
H	1.835727000	8.401253000	20.957974000
H	1.314690000	6.727320000	21.211992000

H	3.034247000	7.130365000	21.319930000
C	0.368747000	8.202300000	23.298066000
H	0.158142000	8.351769000	24.362774000
H	-0.329434000	7.444081000	22.922559000
H	0.163737000	9.145958000	22.776520000
C	2.092191000	6.412022000	23.813108000
H	3.124935000	6.072708000	23.661560000
H	1.416540000	5.630615000	23.444275000
H	1.925604000	6.516841000	24.891064000
C	3.551661000	10.495887000	27.031632000
C	2.325970000	9.889579000	27.748347000
H	1.384075000	10.231281000	27.300793000
H	2.328332000	10.201223000	28.799189000
H	2.341854000	8.792919000	27.734167000
C	4.839667000	9.943755000	27.701123000
H	4.883612000	8.850387000	27.622882000
H	4.852922000	10.210797000	28.765435000
H	5.744590000	10.347459000	27.233285000
C	3.502102000	12.037195000	27.199347000
H	4.390446000	12.530923000	26.789252000
H	3.446869000	12.292348000	28.264568000
H	2.616622000	12.457267000	26.705084000

2•4····Cd^{2+}

O	9.971005000	14.798316000	11.041701000
O	8.325582000	11.260221000	12.797488000
N	2.694547000	11.433972000	6.835178000
N	2.993652000	14.174566000	6.644234000
C	2.671059000	10.086083000	6.924698000
C	1.622933000	9.368209000	6.328416000
H	1.601660000	8.284792000	6.406607000
C	0.623116000	10.055725000	5.645715000
H	-0.190683000	9.513879000	5.169090000
C	0.668677000	11.450518000	5.572695000
H	-0.103821000	11.987277000	5.033496000
C	1.725214000	12.132245000	6.185395000
C	1.851968000	13.622484000	6.150307000
C	0.844823000	14.446014000	5.634918000
H	-0.078498000	14.024028000	5.253685000
C	1.030586000	15.829855000	5.628755000

H	0.255111000	16.480594000	5.230933000
C	2.207274000	16.369255000	6.144928000
H	2.370437000	17.443702000	6.156292000
C	3.190041000	15.514168000	6.664166000
C	4.494082000	16.047351000	7.209196000
H	4.749275000	15.533852000	8.145651000
H	4.353726000	17.106094000	7.459057000
C	5.657425000	15.924012000	6.191236000
H	5.307913000	16.294182000	5.218437000
H	5.914432000	14.864082000	6.045419000
C	6.929988000	16.714697000	6.581182000
H	7.580138000	16.743902000	5.696886000
H	6.641998000	17.756257000	6.782895000
C	7.729990000	16.177005000	7.753033000
C	7.486029000	16.603757000	9.072610000
H	6.693565000	17.324613000	9.269994000
C	8.266768000	16.159169000	10.136477000
H	8.077830000	16.513794000	11.145885000
C	9.321675000	15.254684000	9.913337000
C	9.605554000	14.844478000	8.602566000
H	10.445653000	14.191823000	8.388747000
C	8.805434000	15.301184000	7.546481000
H	9.053420000	14.987771000	6.532486000
C	11.074710000	13.866268000	10.898873000
H	11.932658000	14.386448000	10.450104000
H	10.779817000	13.056244000	10.215870000
C	11.436896000	13.315224000	12.279671000
H	11.818612000	14.137840000	12.897807000
H	12.276864000	12.618400000	12.141760000
C	10.278035000	12.634208000	13.039736000
H	10.609279000	12.391989000	14.056513000
H	9.457206000	13.350544000	13.150808000
C	9.706721000	11.374212000	12.374093000
H	9.739189000	11.451331000	11.277685000
H	10.259909000	10.466992000	12.655839000
C	7.440906000	10.509536000	12.056921000
C	7.786732000	9.650547000	11.005450000
H	8.826028000	9.471126000	10.746283000
C	6.774807000	8.970557000	10.306370000
H	7.062866000	8.275820000	9.517581000

C	5.421092000	9.119050000	10.632003000
C	5.099471000	9.992789000	11.692628000
H	4.059034000	10.116365000	11.992391000
C	6.084415000	10.672515000	12.398760000
H	5.831081000	11.332195000	13.225372000
C	4.336087000	8.279825000	9.979614000
H	3.808090000	7.731084000	10.772174000
H	4.796363000	7.514923000	9.338964000
C	3.271363000	9.052849000	9.161303000
H	2.361944000	8.441417000	9.088487000
H	2.987700000	9.975716000	9.686785000
C	3.744189000	9.396210000	7.726402000
H	4.646448000	10.020580000	7.772169000
H	4.030414000	8.470023000	7.213717000
N	6.592030000	11.964018000	7.302066000
N	5.357574000	13.161758000	9.442634000
N	4.924283000	13.722411000	10.553705000
N	6.009786000	13.796194000	11.371652000
C	7.120427000	13.294272000	10.762534000
H	8.068312000	13.255343000	11.267692000
C	6.699053000	12.878133000	9.510201000
C	7.357050000	12.184889000	8.404626000
C	8.663769000	11.694292000	8.464695000
H	9.259785000	11.870119000	9.350184000
C	9.166392000	10.962577000	7.390167000
H	10.183385000	10.577293000	7.419520000
C	8.355351000	10.715679000	6.280388000
H	8.721214000	10.147480000	5.430193000
C	7.050868000	11.229900000	6.257484000
C	6.106925000	10.965990000	5.144278000
C	5.452162000	12.026530000	4.481541000
H	5.728059000	13.048599000	4.726670000
C	4.554034000	11.766707000	3.437020000
C	4.289136000	10.418753000	3.121150000
H	3.590470000	10.207904000	2.319144000
C	4.924165000	9.342974000	3.755613000
C	5.854742000	9.642750000	4.766795000
H	6.375436000	8.841968000	5.285553000
C	3.949022000	12.878510000	2.557845000
C	4.591488000	12.772118000	1.146291000

H	5.681020000	12.884312000	1.205050000
H	4.199231000	13.560595000	0.491909000
H	4.375799000	11.804525000	0.679276000
C	4.231946000	14.288187000	3.115381000
H	3.825866000	14.413940000	4.124608000
H	3.763465000	15.039729000	2.469629000
H	5.305962000	14.508399000	3.144566000
C	2.414738000	12.701376000	2.431152000
H	2.146048000	11.734549000	1.992371000
H	2.000880000	13.484467000	1.784500000
H	1.928145000	12.775014000	3.409984000
C	4.684433000	7.878075000	3.343342000
C	5.998251000	7.323272000	2.727288000
H	6.290176000	7.903058000	1.843120000
H	5.860554000	6.278658000	2.422130000
H	6.826564000	7.357680000	3.445553000
C	4.305484000	7.030177000	4.585115000
H	5.094000000	7.037071000	5.346448000
H	4.143133000	5.986627000	4.290662000
H	3.377920000	7.400347000	5.038911000
C	3.552364000	7.740280000	2.302941000
H	2.601927000	8.139353000	2.681499000
H	3.397697000	6.681426000	2.066936000
H	3.794380000	8.252002000	1.363725000
C	5.888330000	14.273502000	12.721852000
C	4.678855000	14.073887000	13.393052000
H	3.860108000	13.589982000	12.874575000
C	4.561447000	14.484829000	14.726838000
C	5.680209000	15.088160000	15.329066000
H	5.599621000	15.400997000	16.365415000
C	6.896776000	15.302449000	14.661374000
C	6.992012000	14.876858000	13.328066000
H	7.908826000	15.015839000	12.768453000
C	3.267877000	14.286319000	15.537197000
C	2.169982000	13.571485000	14.720998000
H	1.880902000	14.147295000	13.832008000
H	1.274532000	13.452765000	15.341853000
H	2.486128000	12.569038000	14.402659000
C	3.578170000	13.430829000	16.794343000
H	3.971343000	12.445808000	16.511938000

H	2.663086000	13.278976000	17.379779000
H	4.312871000	13.916597000	17.445697000
C	2.729294000	15.675474000	15.973947000
H	3.450598000	16.212103000	16.600923000
H	1.806242000	15.553652000	16.554033000
H	2.506059000	16.301358000	15.100544000
C	8.088563000	15.940162000	15.399305000
C	8.671747000	14.878780000	16.372246000
H	7.924288000	14.563409000	17.109461000
H	9.530848000	15.293266000	16.914358000
H	9.004046000	13.987300000	15.825663000
C	9.200970000	16.391528000	14.426972000
H	9.642889000	15.552679000	13.877951000
H	10.007409000	16.869867000	14.994479000
H	8.829077000	17.122221000	13.696603000
C	7.625113000	17.180365000	16.206574000
H	7.162248000	17.928416000	15.550741000
H	8.487565000	17.645045000	16.699162000
H	6.903850000	16.919570000	16.988861000
Cd	4.456711000	12.669474000	7.443881000

3•4····Cd^{2+}

C	9.989359000	11.346937000	8.926237000
C	9.191953000	12.490965000	8.811942000
H	9.610457000	13.478854000	8.971013000
C	7.843570000	12.352852000	8.473004000
H	7.210489000	13.233437000	8.389689000
C	7.321746000	11.084296000	8.236231000
H	6.276671000	10.953279000	7.967206000
C	8.159298000	9.962494000	8.343589000
C	7.647145000	8.577450000	8.028987000
H	8.345786000	7.823013000	8.417953000
H	6.692949000	8.422492000	8.552361000
C	7.425920000	8.365566000	6.509086000
H	8.389195000	8.438544000	5.988534000
H	6.802785000	9.190024000	6.136856000
C	6.722732000	7.029397000	6.153264000
H	5.878440000	6.881177000	6.842924000
H	6.282755000	7.144501000	5.154896000
C	7.599862000	5.791131000	6.142430000

C	7.988681000	5.131849000	7.324925000
H	7.648590000	5.506765000	8.289813000
C	8.759899000	3.971126000	7.291490000
H	9.033102000	3.454016000	8.207770000
C	9.179448000	3.432840000	6.062245000
C	8.782706000	4.059157000	4.871063000
H	9.073047000	3.665770000	3.901979000
C	8.004564000	5.221916000	4.926451000
H	7.706386000	5.690979000	3.990589000
C	11.475594000	2.044089000	4.150756000
H	11.300058000	3.075423000	3.819074000
H	11.541284000	1.444305000	3.230890000
C	12.803191000	1.938714000	4.923394000
H	12.697055000	2.388769000	5.917791000
H	13.039476000	0.877592000	5.094885000
C	14.002522000	2.570368000	4.208553000
H	14.076881000	2.200170000	3.179453000
H	14.943010000	2.336179000	4.722362000
C	14.378764000	4.853177000	5.035839000
C	14.769293000	4.459922000	6.323311000
H	14.722866000	3.420253000	6.632469000
C	15.254725000	5.420909000	7.223636000
H	15.585079000	5.092169000	8.208702000
C	15.364771000	6.774138000	6.878510000
C	14.940702000	7.149000000	5.588134000
H	15.030231000	8.185703000	5.266843000
C	14.460104000	6.212091000	4.679176000
H	14.169830000	6.512733000	3.675975000
C	16.070776000	7.769244000	7.786597000
H	16.324682000	7.279827000	8.737612000
H	17.032325000	8.024013000	7.316728000
C	15.328068000	9.094968000	8.071859000
H	16.061299000	9.853068000	8.375522000
H	14.855341000	9.471020000	7.154737000
C	14.254335000	8.969434000	9.178890000
H	13.550900000	8.170543000	8.908133000
H	14.739009000	8.661585000	10.117165000
C	13.500247000	10.259624000	9.406508000
C	14.149804000	11.422635000	9.849827000
H	15.214808000	11.399995000	10.065817000

C	13.415106000	12.594683000	10.013117000
H	13.898929000	13.501976000	10.368044000
C	12.049136000	12.601747000	9.719125000
H	11.472820000	13.511175000	9.851055000
C	11.441767000	11.420895000	9.276636000
N	9.454074000	10.116454000	8.695412000
N	12.176133000	10.284462000	9.144919000
O	13.878169000	4.013652000	4.060653000
C	10.262650000	1.542910000	4.943346000
H	10.443235000	0.529839000	5.321592000
H	9.361551000	1.516706000	4.317885000
O	10.001353000	2.328731000	6.141372000
C	7.216952000	7.671059000	0.427965000
H	6.783885000	6.945264000	1.128682000
H	7.641041000	7.115300000	-0.415468000
H	6.404904000	8.297747000	0.039154000
C	7.593993000	9.347355000	2.259664000
H	6.794119000	9.965970000	1.836962000
H	8.296264000	10.017259000	2.772993000
H	7.137692000	8.682047000	3.005291000
C	8.283874000	8.552608000	1.129371000
C	11.441322000	5.201984000	-1.241543000
H	11.532032000	6.225624000	-1.625170000
H	10.393330000	4.888572000	-1.329573000
H	12.037115000	4.552965000	-1.893816000
C	11.956040000	5.084260000	0.208567000
C	13.444450000	5.524427000	0.241889000
H	14.031189000	4.917806000	-0.459149000
H	13.882245000	5.399508000	1.239487000
H	13.546903000	6.577028000	-0.049864000
C	11.155677000	5.963197000	1.188149000
C	10.128695000	6.820096000	0.773096000
H	9.865756000	6.847420000	-0.278397000
C	9.413724000	7.652087000	1.660875000
C	9.757538000	7.614225000	3.013369000
H	9.239504000	8.219055000	3.746279000
C	10.777542000	6.755031000	3.439830000
C	11.431485000	5.609713000	5.582506000
H	11.607761000	4.645462000	5.140990000
C	11.457459000	6.051424000	6.894455000

C	11.659352000	5.372172000	8.171615000
C	11.976645000	4.013822000	8.305833000
H	12.086459000	3.391307000	7.426794000
C	12.098079000	3.476631000	9.586859000
H	12.336677000	2.422978000	9.712962000
C	11.893281000	4.284643000	10.709934000
H	11.969072000	3.874385000	11.713329000
C	11.564546000	5.632151000	10.511706000
C	11.284111000	6.586555000	11.588809000
C	11.312774000	6.492883000	12.973309000
H	11.579071000	5.693474000	13.648518000
C	11.474549000	5.932465000	2.559837000
H	12.269431000	5.291739000	2.927795000
C	10.824207000	8.174600000	14.787546000
C	10.364701000	7.269784000	15.748473000
H	10.062997000	6.270997000	15.450358000
C	10.259734000	7.680938000	17.084837000
C	10.620874000	9.004587000	17.389577000
H	10.544750000	9.331471000	18.422368000
C	11.069358000	9.927305000	16.428866000
C	11.172678000	9.490044000	15.101034000
H	11.526197000	10.145015000	14.313500000
C	11.448659000	11.357623000	16.854996000
C	11.848240000	12.236856000	15.650083000
H	12.099562000	13.244621000	16.000546000
H	11.027252000	12.330422000	14.926959000
H	12.730246000	11.838744000	15.131516000
C	12.651325000	11.290054000	17.833882000
H	13.521875000	10.825419000	17.353287000
H	12.412403000	10.710639000	18.732898000
H	12.934279000	12.301015000	18.152684000
C	9.755936000	6.744973000	18.199168000
C	8.489054000	7.363995000	18.848053000
H	7.689259000	7.489589000	18.107123000
H	8.698090000	8.344291000	19.291289000
H	8.117673000	6.709337000	19.646131000
C	9.395410000	5.341902000	17.666542000
H	8.594937000	5.383381000	16.916286000
H	9.038165000	4.717350000	18.493086000
H	10.264877000	4.836082000	17.225697000

C	10.867296000	6.593004000	19.272418000
H	10.529753000	5.915924000	20.066537000
H	11.119141000	7.553369000	19.736524000
H	11.783071000	6.177195000	18.833494000
C	11.852424000	3.600924000	0.648043000
H	12.404944000	2.961847000	-0.051737000
H	10.806889000	3.267158000	0.664093000
H	12.277065000	3.450231000	1.645739000
C	8.877966000	9.562264000	0.110797000
H	9.333358000	9.053999000	-0.746817000
H	9.646898000	10.186020000	0.583603000
H	8.088105000	10.220842000	-0.271076000
N	11.068431000	6.695473000	4.844243000
N	10.890589000	7.790067000	5.639105000
N	11.124632000	7.383037000	6.867356000
N	11.464879000	6.141744000	9.263817000
N	10.909268000	7.872833000	11.284713000
N	10.702633000	8.572701000	12.381253000
N	10.949964000	7.730411000	13.424013000
C	10.237894000	12.021102000	17.563298000
H	10.496373000	13.043434000	17.865663000
H	9.941104000	11.473582000	18.464793000
H	9.369520000	12.069767000	16.893989000
Cd	10.916048000	8.391629000	8.937617000

1•4a···Zn^{2+}

Zn	6.338962000	11.848761000	18.822235000
O	8.037290000	9.464708000	25.830472000
O	7.099139000	14.265609000	25.577675000
N	7.233614000	10.886591000	17.329428000
N	5.341583000	12.816973000	17.389134000
N	5.946358000	11.584988000	20.679192000
N	4.828855000	11.121590000	21.212531000
N	5.078450000	11.103746000	22.551510000
C	4.385799000	13.762422000	17.565774000
C	3.788471000	14.346697000	16.437416000
H	3.017097000	15.100622000	16.571745000
C	4.184703000	13.951008000	15.161803000
H	3.723935000	14.395772000	14.282558000
C	5.175702000	12.973118000	15.008750000

H	5.479822000	12.659560000	14.015637000
C	5.748156000	12.409192000	16.148224000
C	6.810135000	11.358886000	16.116698000
C	7.377458000	10.865764000	14.941478000
H	7.060486000	11.234223000	13.971590000
C	8.374634000	9.888315000	15.028600000
H	8.830425000	9.497852000	14.121416000
C	8.782444000	9.420515000	16.276555000
H	9.554392000	8.660061000	16.359242000
C	8.195115000	9.935926000	17.441946000
C	8.566489000	9.428816000	18.814598000
H	8.639248000	10.264361000	19.522330000
H	9.566358000	8.982963000	18.753093000
C	7.564683000	8.352105000	19.330454000
H	6.635094000	8.837383000	19.660911000
H	7.295852000	7.703161000	18.485556000
C	8.118068000	7.442848000	20.462967000
H	7.496849000	6.536254000	20.469205000
H	9.128671000	7.112897000	20.185307000
C	8.140053000	8.007462000	21.872813000
C	9.333282000	8.359874000	22.513798000
H	10.277564000	8.249621000	21.983542000
C	9.360606000	8.829469000	23.837338000
H	10.314126000	9.077519000	24.291666000
C	8.165053000	8.944898000	24.559286000
C	6.962086000	8.534143000	23.950911000
H	6.038794000	8.578943000	24.524042000
C	6.954688000	8.091228000	22.632568000
H	6.009391000	7.776471000	22.191936000
C	9.198301000	10.006921000	26.528871000
H	10.060568000	9.345010000	26.382842000
H	8.913134000	9.961780000	27.585162000
C	9.530981000	11.450315000	26.111672000
H	10.515145000	11.700028000	26.532379000
H	9.676826000	11.473362000	25.021245000
C	8.479114000	12.505361000	26.538860000
H	7.476705000	12.065931000	26.508751000
H	8.646475000	12.795902000	27.582837000
C	8.458014000	13.758623000	25.633218000
H	9.115415000	14.552858000	26.011051000

H	8.794445000	13.504802000	24.618739000
C	6.552565000	14.650312000	24.376194000
C	5.147937000	14.645159000	24.314226000
H	4.589565000	14.369427000	25.205399000
C	4.487767000	15.006814000	23.141739000
H	3.398291000	15.002447000	23.127799000
C	5.193151000	15.417533000	21.993833000
C	6.594327000	15.433393000	22.078401000
H	7.182412000	15.795951000	21.236112000
C	7.277924000	15.047508000	23.240675000
H	8.362769000	15.104727000	23.262754000
C	4.448611000	16.005421000	20.801452000
H	4.637722000	17.089154000	20.794153000
H	3.367786000	15.896689000	20.965009000
C	4.805388000	15.478355000	19.388702000
H	4.583526000	16.262362000	18.652020000
H	5.883502000	15.296853000	19.317630000
C	4.036471000	14.194150000	18.969397000
H	4.233584000	13.382564000	19.680547000
H	2.959111000	14.399553000	19.014411000
C	6.354755000	11.541819000	22.838472000
C	6.920157000	11.877450000	21.602198000
C	8.219959000	12.367964000	21.132232000
C	8.241594000	13.230163000	19.998385000
H	7.330240000	13.772079000	19.739080000
C	9.450272000	13.564592000	19.361365000
C	10.633533000	13.060984000	19.921718000
H	11.573602000	13.312803000	19.445187000
C	10.663854000	12.282553000	21.087936000
C	9.433505000	11.916293000	21.670756000
H	9.414801000	11.215823000	22.499396000
C	9.545102000	14.451402000	18.105356000
C	8.198187000	15.093525000	17.725495000
H	7.425703000	14.346825000	17.513377000
H	8.321641000	15.698954000	16.820464000
H	7.832761000	15.758183000	18.516254000
C	10.025567000	13.563730000	16.924104000
H	11.002274000	13.112547000	17.132201000
H	10.118812000	14.166732000	16.012499000
H	9.312936000	12.752462000	16.729028000

C	10.564401000	15.598809000	18.339252000
H	10.266456000	16.218242000	19.194322000
H	10.611048000	16.241565000	17.451957000
H	11.575658000	15.224387000	18.527596000
C	12.014684000	11.785449000	21.637563000
C	13.149136000	12.806696000	21.368883000
H	13.376995000	12.913061000	20.302540000
H	14.068457000	12.464101000	21.857316000
H	12.900266000	13.796422000	21.771420000
C	11.948762000	11.547183000	23.161770000
H	11.677602000	12.467494000	23.694166000
H	12.928743000	11.222147000	23.529564000
H	11.227461000	10.768719000	23.422144000
C	12.358146000	10.452507000	20.916986000
H	11.588940000	9.694026000	21.102054000
H	13.318121000	10.062639000	21.277228000
H	12.437158000	10.603461000	19.833034000
C	4.139541000	10.536220000	23.480776000
C	3.541120000	9.308908000	23.172506000
H	3.747171000	8.850095000	22.212963000
C	2.710470000	8.698381000	24.116637000
C	2.521286000	9.351636000	25.354655000
H	1.896152000	8.868769000	26.097677000
C	3.102805000	10.586300000	25.665825000
C	3.919217000	11.187787000	24.690029000
H	4.363735000	12.161568000	24.861431000
C	2.040839000	7.335844000	23.863620000
C	2.354866000	6.784174000	22.456629000
H	2.007243000	7.462428000	21.666414000
H	1.845738000	5.824021000	22.316375000
H	3.430227000	6.606663000	22.319331000
C	0.502198000	7.489718000	23.999287000
H	0.214742000	7.853961000	24.991895000
H	0.012575000	6.520222000	23.846048000
H	0.111451000	8.193077000	23.253132000
C	2.559844000	6.319920000	24.916585000
H	3.647765000	6.195659000	24.837320000
H	2.091355000	5.341095000	24.757084000
H	2.328693000	6.639817000	25.939032000
C	2.886569000	11.308429000	27.007668000

C	2.102434000	10.443140000	28.016897000
H	1.093118000	10.208971000	27.656915000
H	1.994446000	10.990293000	28.960425000
H	2.621600000	9.501199000	28.235460000
C	4.266545000	11.658482000	27.628669000
H	4.867475000	10.752050000	27.778697000
H	4.128303000	12.140362000	28.603950000
H	4.838753000	12.351715000	27.000077000
C	2.087003000	12.613556000	26.747841000
H	2.616260000	13.284224000	26.059889000
H	1.928501000	13.154013000	27.689064000
H	1.105581000	12.389271000	26.312267000
N	6.851525000	11.631468000	24.086692000
H	7.858437000	11.652224000	24.162438000
H	6.394402000	11.102635000	24.823086000

1•4b····Zn^{2+}

Zn	5.901215000	11.825502000	19.227614000
O	9.083908000	9.519710000	25.329528000
O	6.673017000	13.197871000	25.580406000
N	5.485977000	10.544437000	17.732662000
N	5.470832000	13.267081000	17.940594000
N	5.848647000	11.330612000	21.104599000
N	4.890991000	10.678846000	21.752955000
N	5.370820000	10.521729000	23.004196000
C	5.905691000	14.541132000	18.062785000
C	6.029060000	15.351636000	16.915038000
C	5.674922000	14.817572000	15.657580000
C	5.201812000	13.474316000	15.553610000
C	5.093599000	12.708376000	16.736382000
C	4.782073000	11.253793000	16.788552000
C	3.837290000	10.574077000	16.000062000
C	3.834658000	9.155171000	16.005104000
C	4.707503000	8.455426000	16.876051000
C	5.473478000	9.193029000	17.801865000
C	6.377315000	8.514596000	18.807259000
H	7.057875000	9.261073000	19.228653000
H	7.026070000	7.808630000	18.261515000
C	5.678430000	7.749667000	19.957999000
H	5.126866000	8.458353000	20.586953000

H	4.927304000	7.067118000	19.539423000
C	6.672419000	6.922081000	20.823383000
H	6.119130000	6.077711000	21.252259000
H	7.431627000	6.479497000	20.160699000
C	7.354558000	7.654859000	21.967230000
C	8.470730000	8.488119000	21.795014000
H	8.891882000	8.631357000	20.800218000
C	9.093952000	9.131552000	22.876441000
H	9.944747000	9.778806000	22.686108000
C	8.604759000	8.934400000	24.178733000
C	7.506690000	8.077165000	24.371167000
H	7.144039000	7.911457000	25.381891000
C	6.897070000	7.460042000	23.284693000
H	6.046288000	6.804872000	23.464058000
C	10.136328000	10.517916000	25.288322000
H	10.820671000	10.324889000	24.454004000
H	10.692955000	10.366636000	26.220731000
C	9.563473000	11.939582000	25.224709000
H	10.404269000	12.636438000	25.091661000
H	8.949498000	12.037125000	24.321164000
C	8.755663000	12.344206000	26.476457000
H	8.107572000	11.520333000	26.797786000
H	9.448900000	12.534840000	27.308017000
C	7.890459000	13.592004000	26.280458000
H	7.591466000	14.028783000	27.241789000
H	8.436194000	14.351916000	25.710043000
C	6.104257000	13.988967000	24.607425000
C	4.938344000	13.468127000	24.017683000
H	4.473310000	12.591911000	24.458180000
C	4.410430000	14.048762000	22.867944000
H	3.533402000	13.598146000	22.404015000
C	4.982462000	15.199055000	22.296746000
C	6.052567000	15.787965000	22.984705000
H	6.471051000	16.729243000	22.633886000
C	6.616355000	15.203578000	24.126775000
H	7.438529000	15.706708000	24.624736000
C	4.417044000	15.794211000	21.017015000
H	3.801537000	16.674606000	21.256291000
H	3.733732000	15.065973000	20.556089000
C	5.480951000	16.228300000	19.985334000

H	4.973733000	16.754905000	19.167588000
H	6.152684000	16.960302000	20.450832000
C	6.336304000	15.051365000	19.416812000
H	7.388999000	15.361407000	19.343112000
H	6.333798000	14.228413000	20.142186000
C	6.605503000	11.092674000	23.148711000
H	7.134088000	11.073446000	24.082812000
C	6.930397000	11.604792000	21.906557000
C	8.074198000	12.284403000	21.279289000
C	8.444088000	11.858712000	19.975928000
H	8.104657000	10.884599000	19.628446000
C	9.378584000	12.576960000	19.213378000
C	9.963288000	13.705888000	19.810812000
H	10.690848000	14.271811000	19.239006000
C	9.675722000	14.115378000	21.122309000
C	8.698094000	13.400457000	21.841727000
H	8.375699000	13.741766000	22.817469000
C	9.784015000	12.167414000	17.785550000
C	9.158881000	10.823069000	17.354817000
H	9.457731000	10.004406000	18.022050000
H	9.507102000	10.566449000	16.347694000
H	8.064010000	10.866629000	17.318912000
C	11.327755000	12.034292000	17.698560000
H	11.836694000	12.981040000	17.909135000
H	11.618712000	11.718582000	16.689424000
H	11.696337000	11.285193000	18.410054000
C	9.300578000	13.270327000	16.804252000
H	8.208335000	13.368605000	16.830419000
H	9.597783000	13.018625000	15.778785000
H	9.737061000	14.245040000	17.051990000
C	10.353990000	15.365049000	21.714248000
C	11.797265000	15.535920000	21.178751000
H	12.400576000	14.638920000	21.365790000
H	12.278097000	16.379458000	21.687341000
H	11.825690000	15.753576000	20.105133000
C	9.509545000	16.602792000	21.305812000
H	9.477723000	16.709733000	20.213558000
H	9.948141000	17.517856000	21.722991000
H	8.481835000	16.513563000	21.674824000
C	10.430205000	15.285869000	23.256282000

H	9.439375000	15.270088000	23.720468000
H	10.960405000	16.162807000	23.644744000
H	10.974994000	14.389984000	23.577608000
C	4.620597000	9.794248000	23.989159000
C	3.685844000	8.851908000	23.556049000
H	3.525001000	8.715423000	22.494514000
C	2.999959000	8.082198000	24.502351000
C	3.290942000	8.298991000	25.861141000
H	2.766200000	7.701145000	26.600953000
C	4.230408000	9.243369000	26.307329000
C	4.898200000	10.008427000	25.340420000
H	5.621154000	10.760193000	25.634270000
C	1.978526000	7.004556000	24.096715000
C	0.601655000	7.347426000	24.724838000
H	0.653048000	7.380391000	25.818977000
H	-0.139028000	6.587320000	24.447375000
H	0.241653000	8.322411000	24.372838000
C	2.462324000	5.626750000	24.623470000
H	3.442926000	5.369510000	24.202130000
H	1.750152000	4.843083000	24.337027000
H	2.549696000	5.616306000	25.715677000
C	1.809665000	6.906936000	22.565141000
H	1.451761000	7.849855000	22.131639000
H	1.071207000	6.132091000	22.328609000
H	2.750088000	6.629625000	22.069882000
C	4.504500000	9.408553000	27.812817000
C	3.205008000	9.881105000	28.516086000
H	2.868774000	10.845071000	28.113544000
H	3.382010000	10.001601000	29.591976000
H	2.390894000	9.158087000	28.389423000
C	4.949134000	8.043119000	28.401469000
H	4.176442000	7.275072000	28.285881000
H	5.156779000	8.147682000	29.473407000
H	5.862694000	7.683524000	27.910485000
C	5.621415000	10.437387000	28.088111000
H	6.570485000	10.125436000	27.632929000
H	5.784606000	10.520924000	29.168773000
H	5.364723000	11.435328000	27.711409000
N	4.715811000	7.058028000	16.834358000
H	5.401206000	6.599036000	17.424353000

H	4.706784000	6.651801000	15.900441000
N	2.939037000	8.472231000	15.206145000
H	2.719826000	8.812729000	14.277163000
H	2.819703000	7.476136000	15.343437000
N	2.903945000	11.245058000	15.187654000
H	2.664502000	12.176135000	15.525265000
H	2.053368000	10.705511000	15.029032000
N	4.986040000	12.981806000	14.271303000
H	4.670522000	13.684435000	13.604890000
H	4.423705000	12.135866000	14.190232000
N	5.790911000	15.576188000	14.510491000
H	6.092423000	15.143690000	13.644951000
H	5.959096000	16.571047000	14.583444000
N	6.509113000	16.662690000	16.983963000
H	6.862720000	16.961216000	17.885932000
H	7.137826000	16.937429000	16.232118000

1•4c····Zn^{2+}

Zn	6.639246000	11.931186000	18.876302000
O	8.727221000	9.170491000	25.677287000
O	7.761556000	13.820784000	25.916677000
N	6.937760000	10.829200000	17.256565000
N	5.191423000	12.828302000	17.789343000
N	6.164310000	11.315930000	20.687277000
N	5.040398000	10.823239000	21.197304000
N	5.212289000	10.829089000	22.528932000
C	4.333590000	13.795323000	18.197058000
C	3.471628000	14.385044000	17.258806000
H	2.777832000	15.158119000	17.579719000
C	3.505865000	13.968325000	15.929550000
H	2.842746000	14.421536000	15.196073000
C	4.385016000	12.951333000	15.539475000
H	4.400924000	12.612533000	14.508766000
C	5.225621000	12.382899000	16.497082000
C	6.173179000	11.261775000	16.208095000
C	6.287555000	10.634431000	14.966873000
H	5.695229000	10.961672000	14.118642000
C	7.176871000	9.561601000	14.828748000
H	7.280407000	9.066786000	13.865596000
C	7.916569000	9.121387000	15.925507000

H	8.594682000	8.276589000	15.835835000
C	7.781784000	9.774394000	17.160471000
C	8.482998000	9.292703000	18.409390000
H	8.706277000	10.136968000	19.077123000
H	9.446327000	8.852028000	18.123386000
C	7.638676000	8.213216000	19.153030000
H	6.748347000	8.682289000	19.594238000
H	7.272052000	7.503487000	18.399043000
C	8.415674000	7.404250000	20.230888000
H	7.918720000	6.426452000	20.303643000
H	9.426087000	7.194826000	19.853465000
C	8.516901000	7.975483000	21.637317000
C	9.748509000	8.342981000	22.196014000
H	10.645375000	8.290539000	21.581105000
C	9.875763000	8.747944000	23.533622000
H	10.853945000	9.015708000	23.921339000
C	8.747393000	8.779517000	24.368534000
C	7.501358000	8.387444000	23.828315000
H	6.633558000	8.372272000	24.483690000
C	7.396529000	8.003036000	22.493102000
H	6.428163000	7.672607000	22.117349000
C	9.941494000	9.605989000	26.366239000
H	10.779840000	8.962893000	26.068418000
H	9.718804000	9.404264000	27.419551000
C	10.286593000	11.090923000	26.156221000
H	11.283120000	11.249791000	26.593930000
H	10.394156000	11.271814000	25.081464000
C	9.261224000	12.085743000	26.764882000
H	8.264155000	11.639765000	26.725548000
H	9.492230000	12.249242000	27.824963000
C	9.174798000	13.447320000	26.028474000
H	9.678821000	14.253091000	26.576191000
H	9.614446000	13.377824000	25.028746000
C	7.216147000	14.311288000	24.765477000
C	5.806727000	14.284828000	24.709160000
H	5.267375000	13.895413000	25.569437000
C	5.125758000	14.768640000	23.593115000
H	4.035730000	14.768104000	23.601170000
C	5.806713000	15.308398000	22.484455000
C	7.211899000	15.318012000	22.549958000

H	7.786005000	15.768450000	21.741349000
C	7.917099000	14.824907000	23.657290000
H	9.001744000	14.881998000	23.666946000
C	5.039402000	16.034879000	21.384598000
H	5.381914000	17.079753000	21.368518000
H	3.977695000	16.075515000	21.663889000
C	5.142268000	15.515628000	19.927534000
H	4.777597000	16.302927000	19.253610000
H	6.196561000	15.366796000	19.664216000
C	4.333831000	14.219612000	19.650448000
H	4.705231000	13.402439000	20.277918000
H	3.292346000	14.392664000	19.953104000
C	6.459171000	11.321065000	22.842544000
C	7.081766000	11.659977000	21.642406000
C	8.332602000	12.274309000	21.143814000
C	8.188788000	13.180701000	20.034582000
H	7.263813000	13.762702000	19.980173000
C	9.327495000	13.692289000	19.363023000
C	10.583190000	13.289936000	19.836820000
H	11.464708000	13.682680000	19.349677000
C	10.756015000	12.464842000	20.956014000
C	9.607648000	11.918682000	21.575635000
H	9.706652000	11.218137000	22.393819000
C	9.239077000	14.762926000	18.256975000
C	8.016763000	14.545402000	17.338281000
H	8.063435000	13.562501000	16.850383000
H	8.006561000	15.305369000	16.548825000
H	7.068974000	14.634099000	17.874650000
C	10.500561000	14.763038000	17.357202000
H	11.396604000	15.099226000	17.889185000
H	10.346450000	15.457449000	16.523694000
H	10.699193000	13.768785000	16.937191000
C	9.121650000	16.152591000	18.945719000
H	8.200280000	16.236948000	19.533092000
H	9.112650000	16.945415000	18.187878000
H	9.970412000	16.330882000	19.615844000
C	12.155553000	12.200847000	21.534403000
C	13.276591000	12.878094000	20.716735000
H	13.315796000	12.503999000	19.685542000
H	14.243599000	12.657144000	21.181527000

H	13.169187000	13.969670000	20.691301000
C	12.180034000	12.777373000	22.977387000
H	11.989162000	13.857861000	22.970464000
H	13.164290000	12.607822000	23.430101000
H	11.428250000	12.300513000	23.614470000
C	12.435787000	10.678941000	21.579927000
H	11.728031000	10.160663000	22.229269000
H	13.445184000	10.501682000	21.969330000
H	12.376166000	10.235339000	20.577851000
C	4.152839000	10.322634000	23.378246000
C	3.769724000	8.987958000	23.220437000
H	4.275888000	8.369350000	22.489013000
C	2.744895000	8.481649000	24.026443000
C	2.136387000	9.362034000	24.945211000
H	1.339446000	8.975026000	25.569884000
C	2.509120000	10.706322000	25.094356000
C	3.550373000	11.186199000	24.283021000
H	3.900182000	12.209142000	24.356879000
C	2.284034000	7.015475000	23.944409000
C	3.078893000	6.214663000	22.890371000
H	2.950732000	6.629386000	21.881535000
H	2.717698000	5.180200000	22.869282000
H	4.151248000	6.184106000	23.125661000
C	0.780550000	6.974381000	23.561994000
H	0.157206000	7.487622000	24.302720000
H	0.438857000	5.933919000	23.501116000
H	0.610238000	7.448199000	22.586738000
C	2.489858000	6.343347000	25.328660000
H	3.547681000	6.361234000	25.620115000
H	2.163304000	5.297115000	25.288825000
H	1.912862000	6.843588000	26.114556000
C	1.863409000	11.644798000	26.130034000
C	0.605407000	11.025810000	26.776396000
H	-0.158175000	10.782840000	26.026480000
H	0.165838000	11.742068000	27.480116000
H	0.838742000	10.116360000	27.343520000
C	2.910392000	11.927642000	27.242106000
H	3.209712000	10.998809000	27.742878000
H	2.487379000	12.603867000	27.995415000
H	3.814669000	12.394880000	26.833391000

C	1.456077000	12.979956000	25.454219000
H	2.322208000	13.517080000	25.049105000
H	0.978709000	13.638626000	26.189327000
H	0.745270000	12.807913000	24.636205000
N	7.032073000	11.355482000	24.160138000
O	6.339239000	11.116735000	25.148350000
O	8.241605000	11.623272000	24.151122000

1•4d···Zn^{2+}

Zn	6.462166000	11.816278000	18.955335000
O	7.977456000	9.287840000	25.908719000
O	6.832254000	13.339302000	25.352227000
N	6.442041000	10.388574000	17.431609000
N	4.789638000	12.545696000	17.917106000
N	5.959017000	11.457473000	20.850116000
N	4.919769000	10.895961000	21.448025000
N	5.223347000	10.914403000	22.766830000
C	4.173253000	13.734667000	18.134271000
C	3.445800000	14.302451000	17.070717000
C	3.423317000	13.696359000	15.818930000
C	4.028164000	12.448679000	15.656230000
C	4.653095000	11.855519000	16.756924000
C	5.248800000	10.480718000	16.785420000
C	4.641222000	9.329611000	16.269336000
C	5.329579000	8.118405000	16.326302000
C	6.618572000	8.096074000	16.849963000
C	7.179882000	9.245503000	17.427605000
C	8.553246000	9.290013000	18.040172000
H	9.037114000	10.201072000	17.662008000
H	9.146339000	8.445494000	17.680066000
C	8.573270000	9.331002000	19.587361000
H	9.591552000	9.607719000	19.880406000
H	7.938487000	10.150120000	19.941665000
C	8.151466000	8.044659000	20.326217000
H	7.150411000	7.724390000	20.005593000
H	8.841378000	7.230363000	20.057882000
C	8.145736000	8.278606000	21.828710000
C	9.301054000	8.726475000	22.486051000
H	10.235089000	8.828944000	21.934629000
C	9.295524000	9.057802000	23.842917000

H	10.213791000	9.399894000	24.307337000
C	8.111858000	8.930092000	24.591626000
C	6.966186000	8.405076000	23.966392000
H	6.064984000	8.264797000	24.556683000
C	6.986600000	8.101748000	22.604933000
H	6.078315000	7.722496000	22.137934000
C	9.095395000	9.947173000	26.568362000
H	10.021134000	9.394929000	26.366088000
H	8.873803000	9.841819000	27.635356000
C	9.206549000	11.429423000	26.167853000
H	10.252169000	11.749368000	26.280199000
H	8.979190000	11.515231000	25.095998000
C	8.291711000	12.370245000	26.983322000
H	7.353987000	11.862494000	27.243812000
H	8.780409000	12.623457000	27.933505000
C	7.932882000	13.664715000	26.256187000
H	7.580363000	14.440961000	26.947821000
H	8.790553000	14.058051000	25.694093000
C	6.467905000	14.188535000	24.325151000
C	5.102251000	14.198219000	23.999916000
H	4.405117000	13.656287000	24.633490000
C	4.653418000	14.903938000	22.884735000
H	3.586257000	14.921886000	22.666582000
C	5.543008000	15.627485000	22.070396000
C	6.902665000	15.623359000	22.424320000
H	7.613770000	16.198338000	21.831248000
C	7.372487000	14.915864000	23.537020000
H	8.432157000	14.935411000	23.775726000
C	5.040340000	16.418184000	20.878072000
H	5.611608000	17.351122000	20.787459000
H	3.993824000	16.707640000	21.043720000
C	5.133062000	15.683331000	19.517611000
H	4.807851000	16.371098000	18.727692000
H	6.182392000	15.443920000	19.302058000
C	4.287304000	14.381481000	19.488385000
H	4.691070000	13.650486000	20.195231000
H	3.270475000	14.616504000	19.828244000
C	6.453176000	11.470787000	22.994824000
H	6.866733000	11.572272000	23.983933000
C	6.922030000	11.851775000	21.751554000

C	8.142031000	12.435859000	21.199225000
C	8.046498000	13.117284000	19.935265000
H	7.165682000	13.752866000	19.785172000
C	9.226021000	13.458202000	19.212571000
C	10.452274000	13.122263000	19.792658000
H	11.362403000	13.359155000	19.255014000
C	10.569436000	12.555105000	21.078364000
C	9.391523000	12.188300000	21.757050000
H	9.443924000	11.650212000	22.696460000
C	9.173713000	14.283017000	17.914009000
C	7.898020000	13.989915000	17.088972000
H	7.831765000	12.924997000	16.821373000
H	7.922116000	14.555293000	16.150597000
H	6.983965000	14.292338000	17.610080000
C	10.394766000	13.995365000	17.006337000
H	11.335613000	14.329049000	17.455347000
H	10.282639000	14.538238000	16.061130000
H	10.481661000	12.926151000	16.773753000
C	9.180902000	15.786706000	18.316669000
H	8.336856000	16.030553000	18.973514000
H	9.109126000	16.415688000	17.421397000
H	10.106128000	16.044979000	18.843830000
C	11.961952000	12.302064000	21.669049000
C	12.622655000	11.144816000	20.866852000
H	12.023519000	10.227714000	20.932868000
H	13.616312000	10.930405000	21.277679000
H	12.742879000	11.403467000	19.808392000
C	12.831026000	13.583317000	21.541327000
H	13.004635000	13.871188000	20.498833000
H	13.811020000	13.404152000	21.998215000
H	12.363814000	14.429829000	22.059670000
C	11.900926000	11.909014000	23.160769000
H	11.413832000	12.685506000	23.764246000
H	12.918826000	11.778927000	23.543539000
H	11.369295000	10.964569000	23.315713000
C	4.325015000	10.350989000	23.736249000
C	3.374300000	9.415809000	23.308402000
H	3.323008000	9.151057000	22.259928000
C	2.522808000	8.830786000	24.249122000
C	2.660178000	9.221350000	25.598470000

H	2.001008000	8.769143000	26.330955000
C	3.606944000	10.157360000	26.035421000
C	4.458875000	10.725981000	25.071826000
H	5.209087000	11.452047000	25.366108000
C	1.468459000	7.779593000	23.861190000
C	1.479079000	7.471568000	22.348578000
H	1.259456000	8.363762000	21.747554000
H	0.709559000	6.724506000	22.123525000
H	2.443354000	7.057486000	22.024248000
C	0.059735000	8.305576000	24.246234000
H	-0.026748000	8.493421000	25.322389000
H	-0.701754000	7.564044000	23.975432000
H	-0.169171000	9.240001000	23.718250000
C	1.762176000	6.463641000	24.630972000
H	2.758972000	6.078539000	24.380775000
H	1.023064000	5.699617000	24.361454000
H	1.714765000	6.606329000	25.716462000
C	3.772542000	10.537804000	27.518567000
C	2.568784000	10.084543000	28.373772000
H	1.628030000	10.515558000	28.008511000
H	2.709462000	10.416696000	29.408655000
H	2.465792000	8.993052000	28.395824000
C	5.054416000	9.839688000	28.049388000
H	4.953593000	8.748437000	27.998550000
H	5.229234000	10.119087000	29.096047000
H	5.936375000	10.116571000	27.460432000
C	3.919202000	12.075190000	27.663983000
H	4.807151000	12.461058000	27.150097000
H	4.011226000	12.340603000	28.724013000
H	3.038269000	12.592260000	27.262311000
N	7.384902000	6.809051000	16.862210000
O	8.392628000	6.770278000	16.160305000
O	6.931735000	5.929451000	17.595953000
N	4.686392000	6.832891000	15.878039000
O	5.224427000	6.269473000	14.933271000
O	3.711068000	6.487835000	16.539420000
N	3.238497000	9.365322000	15.735714000
O	3.017757000	8.722744000	14.714823000
O	2.438696000	10.050629000	16.386341000
N	4.061352000	11.793296000	14.306890000

O	5.129404000	11.237029000	14.015066000
O	3.042839000	11.859141000	13.628039000
N	2.784404000	14.407453000	14.654304000
O	3.565641000	14.760589000	13.776248000
O	1.573459000	14.570488000	14.731928000
N	2.744222000	15.611963000	17.266430000
O	1.863745000	15.631875000	18.124840000
O	3.134818000	16.541494000	16.559355000

2•4a····Zn^{2+}

O	10.370465000	14.930118000	10.874501000
O	9.100557000	11.432084000	13.578355000
N	2.928558000	10.613406000	7.470708000
N	3.155177000	13.182986000	6.676578000
C	2.871685000	9.401201000	8.066406000
C	1.688563000	8.648288000	7.989542000
H	1.643388000	7.671206000	8.463586000
C	0.590460000	9.157632000	7.300703000
H	-0.329933000	8.582162000	7.232554000
C	0.673865000	10.415142000	6.693790000
H	-0.183408000	10.825570000	6.170400000
C	1.864946000	11.135799000	6.794567000
C	2.046312000	12.512267000	6.247629000
C	1.138705000	13.133395000	5.388748000
H	0.265515000	12.603301000	5.023674000
C	1.371640000	14.457843000	5.003787000
H	0.679303000	14.957068000	4.329743000
C	2.476668000	15.140794000	5.507407000
H	2.649669000	16.183057000	5.252174000
C	3.371872000	14.481008000	6.364194000
C	4.569245000	15.183485000	6.961905000
H	4.822786000	14.724730000	7.926516000
H	4.279650000	16.221951000	7.171856000
C	5.802900000	15.189294000	6.026075000
H	5.470523000	15.474837000	5.018819000
H	6.200496000	14.172429000	5.946466000
C	6.926419000	16.162708000	6.459064000
H	7.585013000	16.298252000	5.589432000
H	6.482754000	17.148267000	6.657940000
C	7.786356000	15.753256000	7.643677000

C	7.681733000	16.399872000	8.890535000
H	6.920987000	17.166374000	9.035695000
C	8.559313000	16.118087000	9.936311000
H	8.484419000	16.644489000	10.884915000
C	9.577439000	15.163215000	9.772183000
C	9.695226000	14.501403000	8.539738000
H	10.486856000	13.778839000	8.370531000
C	8.803791000	14.798898000	7.499619000
H	8.934859000	14.294859000	6.542015000
C	11.375030000	13.887485000	10.814124000
H	12.198904000	14.214650000	10.162268000
H	10.933653000	12.981491000	10.370518000
C	11.896216000	13.616293000	12.229282000
H	12.309545000	14.550677000	12.629776000
H	12.743613000	12.919934000	12.138934000
C	10.854253000	13.068653000	13.232332000
H	11.266140000	13.129486000	14.246772000
H	9.977335000	13.726666000	13.225644000
C	10.404065000	11.625035000	12.957187000
H	10.314645000	11.436013000	11.879248000
H	11.114090000	10.893754000	13.367449000
C	8.199994000	10.563101000	13.005392000
C	8.523716000	9.547180000	12.092489000
H	9.561974000	9.296910000	11.888147000
C	7.494928000	8.812522000	11.471594000
H	7.766697000	8.021118000	10.773177000
C	6.143189000	9.047579000	11.754434000
C	5.850824000	10.023024000	12.729221000
H	4.815281000	10.204916000	13.012860000
C	6.851129000	10.765107000	13.348467000
H	6.605836000	11.530033000	14.080919000
C	5.034147000	8.205334000	11.144480000
H	4.547772000	7.654797000	11.961971000
H	5.478340000	7.437948000	10.496394000
C	3.917265000	8.963614000	10.359650000
H	2.945087000	8.522922000	10.615036000
H	3.872071000	10.014792000	10.669517000
C	4.082455000	8.890364000	8.815285000
H	4.978815000	9.439034000	8.498810000
H	4.236903000	7.839839000	8.532052000

N	6.407155000	11.701988000	7.183448000
N	5.109326000	12.269870000	9.462587000
N	4.592050000	12.847977000	10.520885000
N	5.660593000	13.175777000	11.308335000
C	6.854973000	12.798271000	10.722770000
C	6.483292000	12.185106000	9.512350000
C	7.176784000	11.701682000	8.318064000
C	8.517341000	11.290996000	8.248471000
C	9.116292000	11.145107000	6.976516000
C	8.300121000	11.162747000	5.817302000
C	6.925238000	11.408166000	5.965803000
C	5.996082000	11.427837000	4.801706000
C	6.118683000	12.444216000	3.841040000
H	6.901172000	13.185834000	3.963201000
C	5.233136000	12.522775000	2.756760000
C	4.252721000	11.523455000	2.647728000
H	3.574175000	11.556646000	1.799834000
C	4.131424000	10.461172000	3.558697000
C	5.005336000	10.441481000	4.658400000
H	4.960859000	9.634101000	5.383577000
C	5.293791000	13.662078000	1.721719000
C	5.334052000	13.072227000	0.287657000
H	6.217499000	12.436267000	0.150829000
H	5.379798000	13.882680000	-0.449534000
H	4.445659000	12.471522000	0.063587000
C	6.537503000	14.558549000	1.907939000
H	6.539530000	15.066694000	2.880435000
H	6.550988000	15.333649000	1.133443000
H	7.467820000	13.983554000	1.817429000
C	4.020895000	14.535903000	1.886486000
H	3.108957000	13.950529000	1.718352000
H	4.030020000	15.362988000	1.165843000
H	3.968944000	14.960377000	2.897728000
C	3.119837000	9.333510000	3.274160000
C	3.536948000	8.622504000	1.957298000
H	3.526403000	9.312395000	1.106021000
H	2.843342000	7.801906000	1.735917000
H	4.547740000	8.204721000	2.042695000
C	3.083551000	8.280999000	4.401558000
H	4.048490000	7.771001000	4.513440000

H	2.334754000	7.515390000	4.167949000
H	2.814592000	8.726190000	5.364582000
C	1.697695000	9.925952000	3.100530000
H	1.375511000	10.443542000	4.011190000
H	0.978051000	9.125389000	2.889364000
H	1.651375000	10.639605000	2.270751000
C	5.471510000	13.788897000	12.593981000
C	4.492103000	13.270528000	13.448767000
H	3.878899000	12.447481000	13.103066000
C	4.340420000	13.811582000	14.728272000
C	5.197295000	14.867966000	15.106007000
H	5.096535000	15.278157000	16.104598000
C	6.165241000	15.409424000	14.252037000
C	6.288126000	14.854295000	12.964304000
H	6.998731000	15.260626000	12.251922000
C	3.311743000	13.263139000	15.733996000
C	2.419758000	12.165259000	15.114916000
H	1.860267000	12.538546000	14.247498000
H	1.692100000	11.821645000	15.858639000
H	3.005354000	11.290199000	14.802861000
C	4.074052000	12.654839000	16.941999000
H	4.735470000	11.840676000	16.617930000
H	3.362569000	12.247178000	17.670669000
H	4.687728000	13.404669000	17.454543000
C	2.398021000	14.418571000	16.221536000
H	2.971438000	15.212320000	16.712762000
H	1.666541000	14.040358000	16.946331000
H	1.849764000	14.864882000	15.382342000
C	7.078064000	16.582926000	14.652855000
C	6.898720000	16.986733000	16.131778000
H	5.877293000	17.326558000	16.342073000
H	7.575933000	17.815569000	16.368453000
H	7.137248000	16.158498000	16.811055000
C	8.560133000	16.177464000	14.434704000
H	8.816433000	15.299265000	15.040981000
H	9.223111000	17.000293000	14.728781000
H	8.773440000	15.942872000	13.385140000
C	6.737353000	17.808158000	13.762054000
H	6.876012000	17.588019000	12.696141000
H	7.386970000	18.654029000	14.018970000

H	5.695589000	18.118833000	13.909245000
Zn	4.451559000	11.944204000	7.592586000
N	8.901741000	10.968654000	4.566036000
H	8.243789000	10.949107000	3.790134000
H	9.544265000	10.178921000	4.514283000
N	10.475285000	10.895430000	6.854047000
H	11.071644000	11.226400000	7.606624000
H	10.870982000	11.110639000	5.941581000
N	9.277600000	11.089658000	9.417734000
H	10.079236000	10.479590000	9.262342000
H	8.721392000	10.665147000	10.167589000
N	8.057609000	13.082894000	11.251706000
H	8.130149000	13.256453000	12.248042000
H	8.866537000	12.682939000	10.789067000

2•4b····Zn^{2+}

O	9.907640000	14.585090000	10.983879000
O	8.827305000	11.300497000	12.846848000
N	3.023096000	10.764865000	7.242949000
N	2.991431000	13.453297000	6.877608000
C	3.239646000	9.447840000	7.425539000
C	2.490659000	8.500262000	6.699439000
C	1.659364000	8.962036000	5.658411000
C	1.545775000	10.356060000	5.410278000
C	2.132630000	11.259750000	6.321184000
C	1.942313000	12.734631000	6.348624000
C	0.761258000	13.420698000	5.984890000
C	0.781049000	14.845664000	5.987660000
C	1.868949000	15.538227000	6.557521000
C	2.966370000	14.796254000	7.030434000
C	4.152942000	15.480638000	7.668051000
H	4.666861000	14.786189000	8.339283000
H	3.791999000	16.293504000	8.317213000
C	5.167893000	16.056420000	6.651507000
H	4.636084000	16.632403000	5.882618000
H	5.656691000	15.226324000	6.124516000
C	6.233330000	16.968069000	7.299740000
H	6.817197000	17.430069000	6.491708000
H	5.724839000	17.791958000	7.820722000
C	7.184987000	16.283384000	8.263025000

C	7.102541000	16.493194000	9.651712000
H	6.314177000	17.127623000	10.054770000
C	8.030787000	15.935545000	10.529114000
H	7.965983000	16.112271000	11.600271000
C	9.075090000	15.136522000	10.033423000
C	9.176305000	14.909150000	8.652248000
H	9.988990000	14.321152000	8.238112000
C	8.231986000	15.478897000	7.789119000
H	8.343038000	15.318118000	6.716678000
C	11.078172000	13.848660000	10.549878000
H	11.742328000	14.516606000	9.983219000
H	10.765704000	13.038490000	9.873323000
C	11.791065000	13.298009000	11.787608000
H	12.277879000	14.126696000	12.317111000
H	12.597118000	12.637853000	11.434770000
C	10.869808000	12.565550000	12.785206000
H	11.453295000	12.260522000	13.661571000
H	10.119032000	13.275857000	13.143046000
C	10.125211000	11.358496000	12.204494000
H	9.973555000	11.469550000	11.120578000
H	10.663803000	10.414668000	12.369828000
C	7.859893000	10.471667000	12.322141000
C	8.060009000	9.575789000	11.262889000
H	9.044549000	9.429709000	10.829808000
C	6.977866000	8.823234000	10.782396000
H	7.155112000	8.109123000	9.978657000
C	5.696254000	8.935142000	11.335573000
C	5.522533000	9.836951000	12.404026000
H	4.543911000	9.931744000	12.873340000
C	6.582297000	10.588884000	12.899334000
H	6.442777000	11.269355000	13.735425000
C	4.540335000	8.076277000	10.862395000
H	3.948801000	7.765468000	11.733922000
H	4.933808000	7.153418000	10.411541000
C	3.584890000	8.756110000	9.854454000
H	2.699921000	8.117406000	9.723417000
H	3.218911000	9.708453000	10.262385000
C	4.236723000	9.020179000	8.474925000
H	5.014743000	9.784368000	8.580567000
H	4.763342000	8.115628000	8.135870000

N	6.265916000	11.802540000	7.353871000
N	5.091907000	12.673923000	9.603189000
N	4.671946000	13.259232000	10.707580000
N	5.789339000	13.488232000	11.447362000
C	6.909374000	13.032064000	10.810804000
H	7.895899000	13.133206000	11.229076000
C	6.457506000	12.514394000	9.610582000
C	7.092839000	11.933863000	8.433560000
C	8.416220000	11.496626000	8.385410000
H	9.049585000	11.604732000	9.257655000
C	8.882330000	10.895351000	7.215058000
H	9.909387000	10.541663000	7.153435000
C	8.025441000	10.751913000	6.122875000
H	8.368728000	10.304577000	5.194695000
C	6.706116000	11.226854000	6.207876000
C	5.771442000	11.133069000	5.059011000
C	5.183799000	12.295863000	4.526647000
H	5.409673000	13.253893000	4.982983000
C	4.390013000	12.227106000	3.374059000
C	4.161711000	10.957268000	2.811959000
H	3.563224000	10.896654000	1.908226000
C	4.717774000	9.775762000	3.325561000
C	5.540289000	9.888475000	4.458300000
H	6.013068000	9.007212000	4.882083000
C	3.866244000	13.483398000	2.645628000
C	4.538173000	13.549511000	1.246786000
H	5.630021000	13.589999000	1.343001000
H	4.206676000	14.446808000	0.709486000
H	4.285306000	12.676464000	0.634046000
C	4.193858000	14.780359000	3.410702000
H	3.751847000	14.775539000	4.411888000
H	3.787914000	15.642685000	2.868878000
H	5.275355000	14.934244000	3.508067000
C	2.328396000	13.414119000	2.466338000
H	2.014878000	12.500167000	1.948155000
H	1.979213000	14.264370000	1.867814000
H	1.825100000	13.470530000	3.440622000
C	4.553486000	8.428186000	2.595651000
C	5.708919000	8.317373000	1.561605000
H	5.665574000	9.136318000	0.833208000

H	5.639855000	7.367992000	1.015796000
H	6.685861000	8.356075000	2.059479000
C	4.641107000	7.235421000	3.578027000
H	5.636282000	7.142211000	4.028254000
H	4.447209000	6.300022000	3.039994000
H	3.907901000	7.323024000	4.387717000
C	3.202873000	8.335324000	1.846482000
H	2.359510000	8.441215000	2.539560000
H	3.118280000	7.353474000	1.365748000
H	3.107245000	9.085831000	1.053531000
C	5.710920000	14.187649000	12.700211000
C	4.555959000	14.927161000	12.988303000
H	3.744653000	14.939001000	12.271723000
C	4.478943000	15.633577000	14.191243000
C	5.583977000	15.576109000	15.066877000
H	5.529290000	16.125926000	16.000089000
C	6.743100000	14.842886000	14.786741000
C	6.794692000	14.133104000	13.573158000
H	7.665375000	13.533489000	13.330992000
C	3.246107000	16.470771000	14.576018000
C	2.130840000	16.394789000	13.511455000
H	2.460591000	16.799601000	12.545217000
H	1.270503000	16.988463000	13.841035000
H	1.783100000	15.365169000	13.357865000
C	2.676259000	15.945491000	15.920846000
H	2.372240000	14.894653000	15.833494000
H	1.797680000	16.534026000	16.212973000
H	3.411302000	16.019406000	16.730183000
C	3.673201000	17.954659000	14.736626000
H	4.425214000	18.079586000	15.524058000
H	2.804947000	18.570085000	15.002706000
H	4.095367000	18.343262000	13.800819000
C	7.956280000	14.801645000	15.733970000
C	8.327153000	13.325886000	16.037938000
H	7.495770000	12.813045000	16.538158000
H	9.197008000	13.289982000	16.705508000
H	8.577796000	12.762956000	15.132378000
C	9.147497000	15.508831000	15.031582000
H	9.386360000	15.042526000	14.068474000
H	10.042572000	15.460471000	15.664336000

H	8.917895000	16.565652000	14.845465000
C	7.680219000	15.519459000	17.071899000
H	7.465579000	16.585842000	16.929424000
H	8.565095000	15.446996000	17.714734000
H	6.840054000	15.063931000	17.610979000
Zn	4.290067000	12.233195000	7.788493000
N	1.829822000	16.940556000	6.570503000
H	2.612619000	17.395335000	7.028330000
H	0.948123000	17.343045000	6.885352000
N	-0.309650000	15.544884000	5.504080000
H	-0.122171000	16.501312000	5.212707000
H	-0.895121000	15.037499000	4.844066000
N	-0.439453000	12.836726000	5.576051000
H	-1.274615000	13.245149000	5.988775000
H	-0.484048000	11.826693000	5.500610000
N	0.838690000	10.740478000	4.264663000
H	1.065913000	11.667480000	3.915029000
H	0.909290000	10.055315000	3.513637000
N	1.020591000	8.078637000	4.803450000
H	0.089995000	8.305043000	4.468750000
H	1.213299000	7.088942000	4.889688000
N	2.624402000	7.119749000	6.903338000
H	3.165219000	6.848669000	7.716999000
H	1.754835000	6.591335000	6.863461000

2•4c····Zn^{2+}

O	11.402232000	14.393711000	11.239937000
O	9.151097000	10.888429000	13.347680000
N	2.916516000	10.787341000	7.518650000
N	3.680751000	13.326645000	6.989914000
C	2.618082000	9.583580000	8.060407000
C	1.289916000	9.130851000	8.019431000
H	1.039806000	8.161497000	8.442723000
C	0.307011000	9.930300000	7.438309000
H	-0.724184000	9.586577000	7.399255000
C	0.641439000	11.185895000	6.915067000
H	-0.127381000	11.825218000	6.492856000
C	1.970388000	11.606055000	6.971203000
C	2.437939000	12.963207000	6.549333000
C	1.665492000	13.862672000	5.815596000

H	0.687955000	13.580139000	5.437775000
C	2.175695000	15.143282000	5.571106000
H	1.590497000	15.858774000	4.998105000
C	3.416579000	15.509420000	6.088814000
H	3.798682000	16.517486000	5.948899000
C	4.170617000	14.576708000	6.819164000
C	5.474185000	14.967449000	7.494925000
H	5.739199000	14.213089000	8.243409000
H	5.269781000	15.891527000	8.055650000
C	6.676410000	15.234181000	6.549617000
H	6.308634000	15.737040000	5.645622000
H	7.104627000	14.282635000	6.216152000
C	7.790291000	16.135912000	7.154572000
H	8.425742000	16.445799000	6.312929000
H	7.317796000	17.054565000	7.529560000
C	8.700217000	15.576142000	8.240005000
C	8.634106000	16.055382000	9.566007000
H	7.867611000	16.782263000	9.833275000
C	9.562570000	15.675232000	10.532294000
H	9.517102000	16.069883000	11.543110000
C	10.590524000	14.755030000	10.217337000
C	10.690863000	14.289655000	8.882980000
H	11.497327000	13.624551000	8.593492000
C	9.758227000	14.698188000	7.927341000
H	9.893740000	14.362201000	6.899455000
C	12.233500000	13.191246000	11.131630000
H	13.194737000	13.480552000	10.685141000
H	11.738509000	12.478738000	10.469579000
C	12.457414000	12.600795000	12.528639000
H	12.987414000	13.345997000	13.136166000
H	13.155348000	11.758622000	12.404941000
C	11.195645000	12.160694000	13.311969000
H	11.461716000	12.067894000	14.372328000
H	10.446249000	12.954302000	13.246134000
C	10.538361000	10.846256000	12.858827000
H	10.523965000	10.742642000	11.768846000
H	11.035039000	9.966648000	13.290630000
C	8.174179000	10.125669000	12.782855000
C	8.381930000	9.028903000	11.928103000
H	9.385477000	8.671740000	11.728619000

C	7.285444000	8.392531000	11.328911000
H	7.477024000	7.556700000	10.657137000
C	5.962424000	8.784482000	11.582869000
C	5.772295000	9.828370000	12.513269000
H	4.759533000	10.111912000	12.794568000
C	6.844936000	10.494470000	13.098065000
H	6.685355000	11.306535000	13.803217000
C	4.777059000	8.026329000	10.996830000
H	4.295580000	7.477038000	11.818708000
H	5.151450000	7.254799000	10.310823000
C	3.664286000	8.863982000	10.285199000
H	2.680771000	8.488585000	10.595175000
H	3.707152000	9.911581000	10.605115000
C	3.724176000	8.796111000	8.729817000
H	4.700564000	9.158069000	8.377493000
H	3.648282000	7.745134000	8.420359000
N	6.664725000	11.620369000	7.162527000
N	5.445760000	11.771492000	9.458873000
N	4.863095000	12.388334000	10.463878000
N	5.846446000	12.915480000	11.221819000
C	7.072466000	12.577481000	10.682621000
C	6.817102000	11.856375000	9.502163000
C	7.516498000	11.541671000	8.236586000
C	8.875187000	11.278231000	7.945492000
C	9.319519000	11.409552000	6.624732000
C	8.426079000	11.680657000	5.595939000
C	7.051460000	11.665342000	5.871716000
C	5.974101000	11.615455000	4.855810000
C	5.735300000	12.677352000	3.972828000
H	6.367807000	13.554201000	4.017535000
C	4.684285000	12.601172000	3.046570000
C	3.915514000	11.424800000	3.025144000
H	3.127532000	11.339541000	2.282684000
C	4.138970000	10.335633000	3.884554000
C	5.169850000	10.457124000	4.827621000
H	5.414275000	9.627623000	5.485625000
C	4.419929000	13.720548000	2.024224000
C	4.930660000	13.226366000	0.641602000
H	6.006053000	13.013428000	0.674570000
H	4.755293000	13.995154000	-0.120772000

H	4.411059000	12.312601000	0.329297000
C	5.155075000	15.028248000	2.392197000
H	4.859589000	15.387831000	3.387277000
H	4.900268000	15.808538000	1.666383000
H	6.245159000	14.912237000	2.372982000
C	2.903028000	14.025082000	1.932235000
H	2.324711000	13.160230000	1.589446000
H	2.730717000	14.836561000	1.215672000
H	2.504816000	14.338352000	2.905445000
C	3.323706000	9.041118000	3.709903000
C	3.653917000	8.442624000	2.314978000
H	3.389855000	9.134566000	1.507214000
H	3.091224000	7.513970000	2.161023000
H	4.723196000	8.213788000	2.230597000
C	3.660185000	7.987025000	4.785780000
H	4.707109000	7.663693000	4.731145000
H	3.036712000	7.098507000	4.635029000
H	3.462314000	8.364212000	5.796648000
C	1.808706000	9.359044000	3.790507000
H	1.551935000	9.797546000	4.761461000
H	1.225404000	8.438602000	3.666096000
H	1.492675000	10.057622000	3.007992000
C	5.463592000	13.707475000	12.371783000
C	4.567578000	13.132458000	13.274653000
H	4.228815000	12.118678000	13.109891000
C	4.136626000	13.876467000	14.379742000
C	4.620123000	15.189283000	14.504642000
H	4.296214000	15.776952000	15.358797000
C	5.497617000	15.788690000	13.581819000
C	5.923417000	15.020629000	12.490524000
H	6.602189000	15.420789000	11.747050000
C	3.182775000	13.296682000	15.439308000
C	2.754636000	11.850827000	15.108474000
H	2.227520000	11.792712000	14.146571000
H	2.069178000	11.486623000	15.881892000
H	3.612063000	11.165387000	15.083537000
C	3.902149000	13.295072000	16.814723000
H	4.810656000	12.680397000	16.782435000
H	3.237628000	12.885248000	17.585098000
H	4.188955000	14.306935000	17.123307000

C	1.911050000	14.182483000	15.517186000
H	2.152050000	15.215984000	15.790138000
H	1.224801000	13.789076000	16.277115000
H	1.385023000	14.198824000	14.554057000
C	5.953809000	17.242331000	13.801374000
C	6.694264000	17.337952000	15.162551000
H	6.050036000	17.044213000	15.998820000
H	7.019348000	18.370585000	15.339294000
H	7.580566000	16.691278000	15.173398000
C	6.912082000	17.726982000	12.694458000
H	7.827241000	17.123830000	12.663856000
H	7.206993000	18.763808000	12.891014000
H	6.437605000	17.701773000	11.704178000
C	4.708046000	18.167853000	13.818754000
H	4.166413000	18.113024000	12.865605000
H	5.015148000	19.208231000	13.980593000
H	4.011312000	17.896240000	14.619889000
Zn	4.684450000	11.685174000	7.599248000
N	8.909026000	11.883335000	4.201553000
O	9.377773000	10.902927000	3.626291000
O	8.780349000	13.029020000	3.759109000
N	10.763863000	11.183019000	6.260944000
O	11.142944000	10.018771000	6.263819000
O	11.388243000	12.198276000	5.956509000
N	9.804871000	10.627282000	8.894163000
O	9.326520000	9.648837000	9.480397000
O	10.969254000	11.030970000	8.952795000
N	8.323869000	12.910769000	11.259784000
O	9.306684000	12.469620000	10.622978000
O	8.379836000	13.614335000	12.274591000

2•4d····Zn^{2+}

O	9.579468000	14.829011000	11.031857000
O	9.748552000	11.083446000	11.593629000
N	2.786898000	11.288343000	7.324607000
N	3.338738000	13.706393000	6.332656000
C	2.601562000	10.141738000	8.010892000
C	1.332161000	9.517641000	7.959387000
C	0.321144000	10.046111000	7.180541000
C	0.587778000	11.167364000	6.363076000

C	1.840023000	11.834540000	6.505511000
C	2.147764000	13.151626000	5.930447000
C	1.257629000	13.938979000	5.150308000
C	1.624559000	15.262932000	4.828900000
C	2.816728000	15.776869000	5.296767000
C	3.687300000	14.979065000	6.079137000
C	4.957230000	15.524655000	6.665359000
H	5.297174000	14.861456000	7.466934000
H	4.712856000	16.482812000	7.138278000
C	6.104609000	15.746293000	5.643728000
H	5.709535000	16.162912000	4.708595000
H	6.547003000	14.774368000	5.393033000
C	7.189536000	16.708884000	6.189568000
H	7.941484000	16.846379000	5.402249000
H	6.725504000	17.690497000	6.357581000
C	7.863695000	16.235257000	7.460491000
C	7.328500000	16.535679000	8.729337000
H	6.441878000	17.163143000	8.807322000
C	7.929714000	16.077621000	9.897685000
H	7.517049000	16.331193000	10.870915000
C	9.102099000	15.302135000	9.831277000
C	9.680418000	15.035271000	8.580814000
H	10.613453000	14.486421000	8.502360000
C	9.052590000	15.494663000	7.416622000
H	9.515134000	15.280855000	6.453590000
C	10.863396000	14.139991000	11.055459000
H	11.622797000	14.788654000	10.598251000
H	10.788709000	13.211932000	10.470776000
C	11.215289000	13.856274000	12.511580000
H	11.247478000	14.815046000	13.045589000
H	12.240246000	13.457551000	12.530671000
C	10.279457000	12.889903000	13.266040000
H	10.535985000	12.942766000	14.334100000
H	9.237479000	13.226579000	13.193621000
C	10.386263000	11.412486000	12.872823000
H	11.434723000	11.124808000	12.731120000
H	9.958161000	10.766569000	13.648272000
C	8.473656000	10.573963000	11.605081000
C	8.102594000	9.805803000	10.485677000
H	8.854510000	9.581379000	9.733456000

C	6.806042000	9.310179000	10.374472000
H	6.556771000	8.678804000	9.522410000
C	5.838187000	9.557129000	11.368920000
C	6.234690000	10.302850000	12.490988000
H	5.518395000	10.494311000	13.287827000
C	7.531139000	10.806706000	12.622887000
H	7.788168000	11.387547000	13.503228000
C	4.433895000	9.002050000	11.275921000
H	4.000836000	8.970070000	12.283413000
H	4.461645000	7.963422000	10.916722000
C	3.451762000	9.802711000	10.379913000
H	2.435839000	9.460516000	10.611647000
H	3.487637000	10.871535000	10.626686000
C	3.724551000	9.615955000	8.859821000
H	4.655908000	10.120338000	8.576507000
H	3.854843000	8.548504000	8.648934000
N	6.461135000	12.074020000	7.091711000
N	5.175957000	12.864823000	9.297055000
N	4.672812000	13.281864000	10.446181000
N	5.741902000	13.442642000	11.269195000
C	6.913673000	13.139042000	10.637452000
H	7.872323000	13.229800000	11.120265000
C	6.545357000	12.751719000	9.362300000
C	7.260361000	12.289591000	8.180854000
C	8.636013000	12.076205000	8.110345000
H	9.249229000	12.205437000	8.994359000
C	9.193146000	11.696908000	6.887015000
H	10.265252000	11.531389000	6.805551000
C	8.371762000	11.526581000	5.770076000
H	8.780041000	11.229097000	4.808508000
C	6.986797000	11.697885000	5.900447000
C	6.033195000	11.467913000	4.786816000
C	5.972818000	12.378333000	3.718597000
H	6.630743000	13.240820000	3.726567000
C	5.081195000	12.173240000	2.659295000
C	4.300962000	10.996848000	2.679053000
H	3.635443000	10.809651000	1.844280000
C	4.376701000	10.042462000	3.703721000
C	5.228387000	10.321514000	4.790357000
H	5.337940000	9.602272000	5.599727000

C	4.938137000	13.182638000	1.505120000
C	5.046126000	12.451131000	0.141196000
H	6.010132000	11.936283000	0.047159000
H	4.965205000	13.176944000	-0.676559000
H	4.249225000	11.712245000	0.004119000
C	6.022802000	14.281331000	1.550864000
H	5.952258000	14.891885000	2.460669000
H	5.893676000	14.956537000	0.697871000
H	7.034266000	13.859775000	1.490820000
C	3.548150000	13.861464000	1.620159000
H	2.733252000	13.133483000	1.553914000
H	3.413368000	14.594564000	0.816068000
H	3.453581000	14.394858000	2.575550000
C	3.657341000	8.679821000	3.633571000
C	4.740511000	7.566628000	3.574687000
H	5.390550000	7.701870000	2.701980000
H	4.262790000	6.582543000	3.495862000
H	5.371465000	7.564347000	4.471890000
C	2.783721000	8.469692000	4.895181000
H	3.374126000	8.542753000	5.816488000
H	2.322472000	7.475532000	4.879552000
H	1.978711000	9.210870000	4.929137000
C	2.756767000	8.546223000	2.386898000
H	1.969386000	9.308117000	2.366186000
H	2.269859000	7.564348000	2.392918000
H	3.336465000	8.617906000	1.458962000
C	5.572459000	13.805069000	12.648864000
C	4.459712000	13.314460000	13.334910000
H	3.737607000	12.705616000	12.804695000
C	4.316964000	13.600940000	14.698627000
C	5.310650000	14.387305000	15.309136000
H	5.208854000	14.612662000	16.366498000
C	6.424153000	14.902054000	14.623171000
C	6.553055000	14.588551000	13.261741000
H	7.389611000	14.961018000	12.681459000
C	3.133401000	13.073904000	15.529632000
C	2.154302000	12.233973000	14.681443000
H	1.718976000	12.819769000	13.861413000
H	1.328849000	11.885658000	15.312245000
H	2.640791000	11.344579000	14.257821000

C	3.678728000	12.183380000	16.677932000
H	4.233429000	11.323885000	16.279332000
H	2.848071000	11.801975000	17.283939000
H	4.348370000	12.740424000	17.342821000
C	2.358254000	14.278684000	16.127116000
H	2.992625000	14.885707000	16.782799000
H	1.509486000	13.920455000	16.722202000
H	1.970489000	14.928529000	15.332676000
C	7.460046000	15.764013000	15.368019000
C	8.204718000	14.861929000	16.388772000
H	7.513325000	14.420552000	17.115970000
H	8.946659000	15.450080000	16.942753000
H	8.727791000	14.042602000	15.878757000
C	8.494391000	16.390048000	14.407676000
H	9.078201000	15.632713000	13.872454000
H	9.198541000	17.004803000	14.979704000
H	8.014950000	17.041781000	13.665679000
C	6.741524000	16.915201000	16.120012000
H	6.182627000	17.550863000	15.422016000
H	7.480252000	17.540487000	16.635464000
H	6.041601000	16.541818000	16.875362000
Zn	4.489000000	12.411315000	7.430748000
N	3.197693000	17.182632000	4.949936000
O	3.300329000	17.432128000	3.747893000
O	3.382772000	17.951712000	5.894714000
N	0.749832000	16.106973000	3.943127000
O	0.693524000	15.757623000	2.769119000
O	0.213264000	17.066171000	4.491429000
N	0.067121000	13.384357000	4.630293000
O	0.861910000	12.355954000	3.087125000
O	-0.956891000	13.987148000	4.421831000
N	-0.220690000	11.520569000	5.293954000
O	-1.364580000	11.189717000	5.086101000
O	1.337447000	11.391141000	3.733454000
N	-1.030072000	9.397919000	7.146471000
O	-1.093232000	8.331393000	6.538249000
O	-1.916196000	10.003017000	7.739013000
N	1.076521000	8.259190000	8.728033000
O	1.854226000	7.324694000	8.519747000
O	0.113522000	8.273972000	9.495682000

3•4a····Zn^{2+}

C	10.312870000	10.789246000	9.173802000
C	9.398898000	11.829739000	9.363701000
H	9.735681000	12.853611000	9.486925000
C	8.034397000	11.532297000	9.412432000
H	7.309577000	12.326569000	9.576505000
C	7.612242000	10.216384000	9.241142000
H	6.554741000	9.964200000	9.253885000
C	8.565775000	9.203385000	9.051206000
C	8.143186000	7.780637000	8.765551000
H	8.980298000	7.091678000	8.936227000
H	7.338856000	7.498525000	9.459138000
C	7.623292000	7.648450000	7.308792000
H	8.456526000	7.832591000	6.619062000
H	6.890522000	8.447694000	7.134549000
C	6.940557000	6.293256000	6.979395000
H	6.316407000	5.992332000	7.833311000
H	6.249852000	6.467685000	6.145027000
C	7.868735000	5.156897000	6.595591000
C	8.489839000	4.341856000	7.556953000
H	8.298475000	4.511719000	8.615717000
C	9.326953000	3.287963000	7.184418000
H	9.785912000	2.652099000	7.937972000
C	9.573482000	3.031413000	5.828277000
C	8.949694000	3.816558000	4.850638000
H	9.130833000	3.644265000	3.793041000
C	8.107677000	4.861751000	5.242855000
H	7.634083000	5.462628000	4.468987000
C	11.736842000	1.775857000	3.376100000
H	11.648023000	2.831542000	3.090851000
H	11.637322000	1.207907000	2.438788000
C	13.115506000	1.484830000	4.002866000
H	13.170128000	1.906354000	5.011580000
H	13.223936000	0.396238000	4.126759000
C	14.328623000	1.959137000	3.184833000
H	14.254373000	1.609929000	2.148951000
H	15.258274000	1.556754000	3.609592000
C	14.903382000	4.152263000	4.109771000
C	15.263365000	3.651122000	5.368739000
H	15.292438000	2.584127000	5.565423000

C	15.547559000	4.544251000	6.412098000
H	15.784865000	4.135218000	7.393949000
C	15.499566000	5.933491000	6.241125000
C	15.243023000	6.412011000	4.941973000
H	15.247934000	7.482413000	4.743106000
C	14.964495000	5.540209000	3.891576000
H	14.773856000	5.918376000	2.890506000
C	15.723850000	6.851722000	7.431940000
H	15.502633000	6.275786000	8.342548000
H	16.787638000	7.126999000	7.505182000
C	14.872104000	8.142037000	7.439945000
H	15.330637000	8.911150000	6.804592000
H	13.881385000	7.941365000	7.019448000
C	14.705664000	8.699997000	8.877495000
H	14.205619000	7.942233000	9.497965000
H	15.695834000	8.873640000	9.318862000
C	13.911098000	9.980913000	8.929062000
C	14.528716000	11.236301000	8.833553000
H	15.610200000	11.297981000	8.741316000
C	13.746668000	12.389464000	8.862841000
H	14.209709000	13.371109000	8.790862000
C	12.360329000	12.277171000	8.985460000
H	11.742323000	13.168833000	8.998303000
C	11.785298000	11.004878000	9.074768000
N	9.880432000	9.504246000	9.050672000
N	12.567022000	9.890211000	9.044203000
O	14.439226000	3.401520000	3.055282000
C	10.558850000	1.362960000	4.269239000
H	10.637885000	0.302424000	4.538657000
H	9.599275000	1.516399000	3.764574000
O	10.547641000	2.071946000	5.556658000
C	6.800843000	7.304654000	1.322417000
H	6.692862000	6.391130000	1.921791000
H	7.249647000	7.028538000	0.361687000
H	5.798042000	7.700929000	1.121084000
C	6.917775000	8.738374000	3.379827000
H	5.924535000	9.136078000	3.142059000
H	7.457860000	9.507872000	3.946155000
H	6.776519000	7.865033000	4.029526000
C	7.651688000	8.363281000	2.073833000

C	11.281930000	6.496564000	-1.110616000
H	11.017082000	7.538518000	-1.328742000
H	10.367729000	5.890403000	-1.145824000
H	11.940352000	6.149492000	-1.915233000
C	12.009501000	6.360290000	0.243415000
C	13.304818000	7.213937000	0.192525000
H	13.949614000	6.875418000	-0.627939000
H	13.878427000	7.138017000	1.123919000
H	13.067013000	8.272125000	0.027924000
C	11.141679000	6.833135000	1.423688000
C	9.853326000	7.354464000	1.261134000
H	9.445766000	7.431575000	0.259598000
C	9.055544000	7.790394000	2.341003000
C	9.576580000	7.667468000	3.630746000
H	9.006931000	7.960493000	4.503126000
C	10.862126000	7.141706000	3.810444000
C	11.736262000	5.765751000	5.734473000
C	11.804946000	6.041133000	7.113574000
C	12.053336000	5.306810000	8.365882000
C	12.447087000	3.969915000	8.509521000
C	12.554420000	3.442693000	9.832877000
C	12.257048000	4.250779000	10.959537000
C	11.876315000	5.583407000	10.733576000
C	11.524351000	6.586633000	11.745622000
C	11.204930000	6.599429000	13.120092000
C	11.651826000	6.744755000	2.734754000
H	12.663696000	6.396633000	2.913855000
C	10.649071000	8.498055000	14.689054000
C	9.534923000	8.046469000	15.402943000
H	8.886633000	7.297643000	14.959510000
C	9.263076000	8.588457000	16.669042000
C	10.132649000	9.580077000	17.153498000
H	9.930144000	10.004100000	18.132928000
C	11.247810000	10.052365000	16.440253000
C	11.504193000	9.485008000	15.183482000
H	12.352214000	9.793240000	14.581651000
C	12.141101000	11.149051000	17.049690000
C	13.325119000	11.517016000	16.129965000
H	13.929265000	12.296627000	16.607891000
H	12.983638000	11.908407000	15.162704000

H	13.981865000	10.656329000	15.947452000
C	12.711449000	10.649824000	18.403772000
H	13.318993000	9.746992000	18.262470000
H	11.916607000	10.414260000	19.120748000
H	13.346836000	11.424154000	18.851356000
C	8.062844000	8.137810000	17.522367000
C	7.164387000	9.364684000	17.832139000
H	6.793096000	9.821910000	16.906271000
H	7.703927000	10.132816000	18.397372000
H	6.300432000	9.055068000	18.432761000
C	7.204258000	7.072695000	16.808143000
H	6.772881000	7.456447000	15.874260000
H	6.372505000	6.775399000	17.457109000
H	7.780992000	6.166850000	16.579139000
C	8.593134000	7.535352000	18.851056000
H	7.754555000	7.222188000	19.485456000
H	9.188983000	8.262380000	19.414962000
H	9.223622000	6.657473000	18.658826000
C	12.379363000	4.866735000	0.456267000
H	12.952812000	4.495233000	-0.402118000
H	11.473157000	4.252966000	0.549238000
H	12.994255000	4.713164000	1.350611000
C	7.784469000	9.638534000	1.198996000
H	8.261202000	9.421861000	0.236480000
H	8.383896000	10.402469000	1.710072000
H	6.792435000	10.059636000	0.993583000
N	11.332155000	6.953047000	5.155314000
N	11.164162000	7.932408000	6.100800000
N	11.446510000	7.365056000	7.241960000
N	11.817409000	6.073714000	9.468276000
N	11.396245000	7.890451000	11.315265000
N	11.055564000	8.704903000	12.276229000
N	10.937906000	7.921378000	13.402343000
C	11.286749000	12.423196000	17.284491000
H	11.903624000	13.213395000	17.730238000
H	10.448222000	12.230669000	17.963300000
H	10.876458000	12.798762000	16.338364000
Zn	11.445772000	8.143351000	9.191812000
N	12.006907000	4.666761000	4.985175000
H	11.795163000	4.756498000	3.995204000

H	11.731954000	3.771206000	5.372750000
N	11.083828000	5.609109000	14.036020000
H	11.039387000	5.864488000	15.017587000
H	11.513172000	4.722340000	13.754995000
N	12.835279000	3.100642000	7.462311000
H	13.290138000	3.559604000	6.676697000
H	12.118162000	2.442339000	7.151710000
N	12.948645000	2.139315000	9.970163000
H	13.367408000	1.731398000	9.135900000
H	13.318404000	1.826294000	10.859778000
N	12.403925000	3.730042000	12.296474000
H	13.375377000	3.746786000	12.618383000
H	12.058393000	2.771622000	12.374604000

3•4b····Zn^{2+}

C	10.226892000	10.944845000	9.161505000
C	9.552474000	12.153418000	8.905214000
C	8.189295000	12.115923000	8.528726000
C	7.564699000	10.867215000	8.312357000
C	8.351305000	9.696723000	8.387978000
C	7.787824000	8.346301000	8.017603000
H	8.483134000	7.568411000	8.357322000
H	6.851625000	8.171972000	8.574592000
C	7.516320000	8.181822000	6.501870000
H	8.467151000	8.266750000	5.964335000
H	6.892396000	9.014390000	6.152562000
C	6.811170000	6.853037000	6.132531000
H	5.964193000	6.697464000	6.818444000
H	6.377037000	6.966861000	5.131466000
C	7.698417000	5.623279000	6.129151000
C	8.112437000	4.988872000	7.316816000
H	7.774585000	5.369512000	8.279627000
C	8.912200000	3.847814000	7.292115000
H	9.207816000	3.351143000	8.212961000
C	9.325610000	3.297383000	6.066565000
C	8.902396000	3.898161000	4.870619000
H	9.181356000	3.493204000	3.903459000
C	8.105097000	5.048064000	4.917490000
H	7.790991000	5.501181000	3.978592000
C	11.550988000	1.928602000	4.107408000

H	11.348747000	2.965437000	3.811897000
H	11.571145000	1.346915000	3.173590000
C	12.926343000	1.841772000	4.789567000
H	12.874404000	2.259049000	5.803592000
H	13.215946000	0.786468000	4.905611000
C	14.030145000	2.547618000	3.996735000
H	14.044344000	2.191462000	2.959788000
H	15.020958000	2.369854000	4.432033000
C	14.368630000	4.835891000	4.806587000
C	14.864605000	4.449585000	6.059082000
H	14.850185000	3.410398000	6.372963000
C	15.417140000	5.416353000	6.912079000
H	15.829738000	5.095343000	7.868088000
C	15.485898000	6.767680000	6.552384000
C	14.950820000	7.137237000	5.302999000
H	14.999630000	8.177110000	4.981484000
C	14.406007000	6.193367000	4.438433000
H	14.031282000	6.485075000	3.460745000
C	16.194399000	7.791702000	7.418874000
H	16.717357000	7.276436000	8.237419000
H	16.975186000	8.277722000	6.816342000
C	15.290600000	8.896121000	8.007896000
H	15.926237000	9.697811000	8.409879000
H	14.685716000	9.353506000	7.213305000
C	14.339273000	8.385891000	9.117114000
H	13.642176000	7.662919000	8.681634000
H	14.915332000	7.830803000	9.873497000
C	13.566582000	9.513498000	9.760230000
C	14.144587000	10.334365000	10.751725000
C	13.370997000	11.369600000	11.313333000
C	12.061195000	11.634399000	10.814848000
C	11.588414000	10.839607000	9.738850000
N	9.625860000	9.764325000	8.818668000
N	12.329972000	9.775513000	9.297874000
O	13.802646000	3.981772000	3.880570000
C	10.393159000	1.401779000	4.959974000
H	10.621692000	0.401632000	5.346741000
H	9.466246000	1.336624000	4.375693000
O	10.157497000	2.201500000	6.152251000
C	7.160549000	8.186212000	0.524315000

H	6.663619000	7.517387000	1.239050000
H	7.503934000	7.580887000	-0.321825000
H	6.415832000	8.896926000	0.145304000
C	7.751666000	9.812166000	2.342815000
H	7.014133000	10.514071000	1.936523000
H	8.529589000	10.402308000	2.844337000
H	7.242061000	9.196776000	3.096174000
C	8.330233000	8.949438000	1.200760000
C	11.154802000	5.379156000	-1.262411000
H	11.437279000	6.389690000	-1.583175000
H	10.073815000	5.258353000	-1.406258000
H	11.655684000	4.667701000	-1.929084000
C	11.575170000	5.102844000	0.196480000
C	13.119355000	5.217248000	0.299409000
H	13.594563000	4.540898000	-0.421907000
H	13.482646000	4.947749000	1.298024000
H	13.451195000	6.239085000	0.076303000
C	10.923030000	6.083485000	1.189660000
C	9.972820000	7.035863000	0.802283000
H	9.681874000	7.087788000	-0.240913000
C	9.372315000	7.935608000	1.708293000
C	9.750815000	7.865607000	3.050767000
H	9.319997000	8.522057000	3.795440000
C	10.698430000	6.914216000	3.449158000
C	11.440418000	5.718039000	5.529753000
H	11.616011000	4.771280000	5.051848000
C	11.501587000	6.119920000	6.850835000
C	11.754816000	5.437177000	8.111275000
C	12.150401000	4.101299000	8.248008000
H	12.290126000	3.481582000	7.370074000
C	12.322238000	3.586448000	9.533464000
H	12.622398000	2.549576000	9.666867000
C	12.088746000	4.393888000	10.651860000
H	12.197847000	3.998823000	11.658303000
C	11.683011000	5.719235000	10.449112000
C	11.356281000	6.679384000	11.501387000
C	11.455972000	6.682904000	12.885114000
H	11.836231000	5.963424000	13.594640000
C	11.283643000	6.027385000	2.549317000
H	12.027932000	5.312947000	2.886880000

C	10.843203000	8.431429000	14.600684000
C	10.589891000	7.543603000	15.650048000
H	10.467755000	6.485532000	15.444347000
C	10.454469000	8.036861000	16.955126000
C	10.566172000	9.424771000	17.140312000
H	10.453893000	9.819961000	18.145531000
C	10.809140000	10.328628000	16.091901000
C	10.956574000	9.810481000	14.797576000
H	11.153917000	10.448588000	13.944452000
C	10.906149000	11.834978000	16.397378000
C	11.203027000	12.668491000	15.132133000
H	11.260105000	13.729817000	15.402589000
H	10.410001000	12.554198000	14.381647000
H	12.160834000	12.380557000	14.679565000
C	12.047810000	12.077235000	17.419886000
H	13.010741000	11.738676000	17.016100000
H	11.870334000	11.544554000	18.360587000
H	12.127894000	13.146810000	17.651298000
C	10.173323000	7.119990000	18.159956000
C	8.813314000	7.517860000	18.793377000
H	7.998073000	7.417201000	18.065781000
H	8.819604000	8.552853000	19.154174000
H	8.592678000	6.866930000	19.648310000
C	10.110023000	5.630347000	17.759372000
H	9.301182000	5.431489000	17.043990000
H	9.917766000	5.019891000	18.648945000
H	11.057856000	5.286464000	17.324072000
C	11.307006000	7.295629000	19.205508000
H	11.121910000	6.648565000	20.071765000
H	11.371707000	8.327662000	19.568305000
H	12.280060000	7.024130000	18.776647000
C	11.142658000	3.659774000	0.570417000
H	11.591238000	2.937678000	-0.123334000
H	10.051729000	3.551873000	0.517702000
H	11.465277000	3.399783000	1.584527000
C	9.007897000	9.885944000	0.164841000
H	9.400134000	9.324039000	-0.690536000
H	9.841763000	10.434245000	0.620964000
H	8.283177000	10.615851000	-0.216722000
N	11.032731000	6.822042000	4.841516000

N	10.863981000	7.887895000	5.677679000
N	11.143805000	7.446459000	6.884990000
N	11.536152000	6.213022000	9.197303000
N	10.834684000	7.893288000	11.128576000
N	10.604160000	8.642575000	12.180611000
N	10.982959000	7.906195000	13.269608000
C	9.557113000	12.318153000	16.993562000
H	9.605349000	13.391970000	17.214306000
H	9.313189000	11.796509000	17.925883000
H	8.735170000	12.148317000	16.286349000
Zn	11.000158000	8.251475000	8.857223000
N	6.210930000	10.842616000	7.947791000
H	5.619462000	11.488801000	8.467632000
H	5.785054000	9.922526000	7.909658000
N	7.518399000	13.311365000	8.293677000
H	7.626088000	14.070569000	8.958461000
H	6.605243000	13.265633000	7.857882000
N	10.177233000	13.412442000	9.015894000
H	9.749457000	14.111199000	8.407705000
H	11.183266000	13.378113000	8.855835000
N	11.279683000	12.560797000	11.475420000
H	11.627657000	12.958550000	12.336596000
H	10.580437000	13.099786000	10.971176000
N	13.840663000	12.097822000	12.402969000
H	13.702479000	13.104030000	12.405234000
H	14.715059000	11.804251000	12.821215000
N	15.436333000	10.100127000	11.253346000
H	15.988044000	10.940124000	11.420969000
H	15.982471000	9.422747000	10.732308000

3•4c····Zn^{2+}

C	9.265420000	10.394786000	8.707153000
C	8.176541000	11.216137000	8.398473000
H	8.314203000	12.275979000	8.213860000
C	6.900793000	10.655177000	8.312480000
H	6.043530000	11.283705000	8.081986000
C	6.739986000	9.285122000	8.505729000
H	5.759377000	8.823072000	8.422758000
C	7.855603000	8.492188000	8.811234000
C	7.717514000	6.989802000	8.949932000

H	8.587928000	6.587425000	9.486739000
H	6.835042000	6.767476000	9.565249000
C	7.586633000	6.269736000	7.575777000
H	8.490164000	6.481422000	6.999677000
H	6.753050000	6.707389000	7.010903000
C	7.393977000	4.743957000	7.718791000
H	7.959423000	4.395043000	8.597472000
H	6.340549000	4.516824000	7.941301000
C	7.874523000	3.889750000	6.550098000
C	7.983723000	2.462814000	6.764263000
H	7.537616000	2.042284000	7.665592000
C	8.693487000	1.622311000	5.938186000
H	8.847725000	0.578562000	6.199832000
C	9.360177000	2.135497000	4.801119000
C	9.051643000	3.546147000	4.328930000
H	8.361258000	3.425389000	3.459367000
C	8.317298000	4.373056000	5.362902000
H	8.224676000	5.419067000	5.085811000
C	12.265010000	2.026296000	2.857470000
H	12.260664000	3.100130000	3.040619000
H	12.603449000	1.890662000	1.817010000
C	13.250386000	1.336592000	3.819143000
H	12.889537000	1.426317000	4.847532000
H	13.324754000	0.261473000	3.596142000
C	14.679476000	1.909915000	3.703418000
H	15.135277000	1.597567000	2.757000000
H	15.313645000	1.547341000	4.520798000
C	14.834051000	4.169827000	4.722623000
C	15.032843000	3.755659000	6.051785000
H	15.115973000	2.704134000	6.308173000
C	15.086271000	4.712407000	7.072178000
H	15.260206000	4.364390000	8.089128000
C	14.934435000	6.087771000	6.825265000
C	14.769597000	6.484095000	5.484893000
H	14.681686000	7.538302000	5.228691000
C	14.732635000	5.549004000	4.453641000
H	14.638566000	5.865040000	3.418291000
C	15.000949000	7.093451000	7.969398000
H	14.671823000	6.594071000	8.892825000
H	16.047519000	7.383395000	8.153424000

C	14.156843000	8.368062000	7.755583000
H	14.616242000	9.007732000	6.991020000
H	13.171825000	8.091202000	7.366217000
C	13.974482000	9.199955000	9.053064000
H	13.646058000	8.539044000	9.867396000
H	14.937052000	9.622260000	9.365022000
C	12.964665000	10.312750000	8.895694000
C	13.337395000	11.651222000	8.713212000
H	14.390586000	11.919611000	8.701048000
C	12.349008000	12.623533000	8.560684000
H	12.622101000	13.668648000	8.433009000
C	11.002560000	12.251547000	8.573644000
H	10.232697000	13.007057000	8.459396000
C	10.671553000	10.902148000	8.739146000
N	9.078349000	9.061120000	8.938046000
N	11.653358000	9.974546000	8.906079000
O	14.722499000	3.363634000	3.624465000
C	10.824434000	1.520358000	2.853121000
H	10.752980000	0.500322000	2.462897000
H	10.179051000	2.191853000	2.280983000
O	10.208169000	1.335205000	4.214138000
C	6.993131000	7.610127000	0.776942000
H	6.764146000	6.638542000	1.233762000
H	7.616565000	7.430955000	-0.105996000
H	6.051919000	8.058961000	0.436222000
C	6.724705000	8.804271000	2.969134000
H	5.805394000	9.266430000	2.591399000
H	7.157593000	9.486751000	3.712683000
H	6.440997000	7.870604000	3.473628000
C	7.694459000	8.548334000	1.795052000
C	11.866874000	6.970922000	-0.827632000
H	11.628489000	8.031637000	-0.974141000
H	10.988045000	6.375107000	-1.105287000
H	12.673135000	6.717171000	-1.525426000
C	12.325895000	6.676485000	0.616516000
C	13.569213000	7.553839000	0.923764000
H	14.374658000	7.330796000	0.213256000
H	13.952530000	7.374735000	1.935141000
H	13.323639000	8.619912000	0.840741000
C	11.232452000	6.990683000	1.654244000

C	10.008583000	7.581886000	1.313142000
H	9.816141000	7.812874000	0.271372000
C	9.010179000	7.897548000	2.258603000
C	9.263371000	7.592637000	3.598791000
H	8.541652000	7.811195000	4.378216000
C	10.477373000	6.989833000	3.942551000
C	11.175947000	5.541745000	5.941026000
C	11.339759000	5.881804000	7.308199000
C	11.812112000	5.283829000	8.538821000
C	12.325648000	3.974829000	8.792700000
C	13.066220000	3.757675000	9.954901000
C	13.147281000	4.748209000	10.944687000
C	12.319385000	5.886401000	10.790676000
C	11.933835000	6.870257000	11.809471000
C	11.954639000	6.957408000	13.200055000
C	11.461227000	6.694152000	3.009744000
H	12.385529000	6.231388000	3.339492000
C	11.105203000	8.764593000	14.806815000
C	9.770643000	9.044919000	15.125765000
H	8.988347000	8.778570000	14.423442000
C	9.483527000	9.650572000	16.352097000
C	10.566772000	9.954239000	17.205351000
H	10.347211000	10.417753000	18.160604000
C	11.904604000	9.686473000	16.886310000
C	12.169218000	9.077299000	15.647142000
H	13.184114000	8.847211000	15.342572000
C	13.076323000	10.019058000	17.827554000
C	14.054725000	10.980851000	17.101832000
H	14.894259000	11.227534000	17.763016000
H	13.551851000	11.915808000	16.823818000
H	14.470757000	10.531361000	16.191766000
C	13.814553000	8.702578000	18.191625000
H	14.217304000	8.199448000	17.304688000
H	13.139850000	8.002831000	18.699982000
H	14.653255000	8.919423000	18.864295000
C	8.047248000	9.978621000	16.796831000
C	7.932987000	11.504919000	17.055989000
H	8.156032000	12.075216000	16.145163000
H	8.620512000	11.837466000	17.841677000
H	6.914043000	11.754029000	17.376919000

C	7.001938000	9.584696000	15.731455000
H	7.162702000	10.120599000	14.786282000
H	5.999335000	9.843029000	16.090941000
H	7.011187000	8.505307000	15.530325000
C	7.732990000	9.203912000	18.104838000
H	6.711164000	9.426863000	18.435582000
H	8.414897000	9.482565000	18.915996000
H	7.815256000	8.120944000	17.948848000
C	12.710111000	5.176278000	0.710622000
H	13.477688000	4.939629000	-0.036448000
H	11.836185000	4.541407000	0.516859000
H	13.113163000	4.908905000	1.693100000
C	8.011081000	9.907390000	1.115869000
H	8.660194000	9.782214000	0.242124000
H	8.513297000	10.586993000	1.816172000
H	7.083199000	10.385227000	0.777771000
N	10.706025000	6.687405000	5.332871000
N	10.550894000	7.683986000	6.252969000
N	10.920487000	7.192489000	7.400906000
N	11.751027000	6.133081000	9.601354000
N	11.315675000	8.013860000	11.377102000
N	10.977254000	8.794454000	12.382998000
N	11.375625000	8.160101000	13.520636000
C	12.604133000	10.694973000	19.132364000
H	13.473135000	10.908934000	19.764882000
H	11.930854000	10.047258000	19.707646000
H	12.093671000	11.647232000	18.939674000
Zn	10.887867000	8.110567000	9.280616000
N	12.260796000	5.918054000	14.184728000
O	11.925265000	4.772675000	13.835374000
O	12.781064000	6.246931000	15.247936000
N	11.180699000	4.249041000	5.415469000
O	10.171119000	4.232720000	3.840903000
O	12.220096000	3.594579000	5.333445000
N	14.100772000	4.650911000	12.061952000
O	14.445740000	3.534796000	12.448804000
O	14.508039000	5.741876000	12.513078000
N	13.762033000	2.432902000	10.150692000
O	13.072165000	1.530362000	10.606214000
O	14.942405000	2.409673000	9.815831000

N	11.911214000	2.840248000	7.970349000
O	10.714695000	2.896007000	7.603224000
O	12.696917000	1.914193000	7.749769000

3•4d····Zn^{2+}

C	9.832450000	10.459488000	9.868694000
C	9.356343000	11.099566000	11.022878000
C	8.420519000	10.430399000	11.819311000
C	8.016719000	9.148219000	11.465459000
C	8.570073000	8.510882000	10.344568000
C	8.174930000	7.155386000	9.842433000
H	9.079921000	6.561118000	9.649919000
H	7.591827000	6.614707000	10.591962000
C	7.360611000	7.297169000	8.526527000
H	7.985396000	7.786785000	7.767900000
H	6.500989000	7.955461000	8.713094000
C	6.843253000	5.938932000	7.998076000
H	6.266118000	5.445634000	8.792912000
H	6.134023000	6.152939000	7.188399000
C	7.913913000	4.999301000	7.484926000
C	8.589534000	4.113482000	8.345194000
H	8.344522000	4.100209000	9.406902000
C	9.514111000	3.190200000	7.858130000
H	9.995333000	2.475846000	8.521367000
C	9.787438000	3.123636000	6.482206000
C	9.135169000	4.010172000	5.608826000
H	9.318262000	3.971680000	4.539002000
C	8.215920000	4.934337000	6.114831000
H	7.699754000	5.595905000	5.419957000
C	11.680818000	2.013543000	3.812547000
H	11.624085000	3.100333000	3.664411000
H	11.478684000	1.559383000	2.830999000
C	13.086605000	1.623329000	4.295422000
H	13.245447000	2.026116000	5.302677000
H	13.155217000	0.528821000	4.385985000
C	14.213224000	2.067819000	3.354888000
H	14.128245000	1.561536000	2.387507000
H	15.200537000	1.832989000	3.776086000
C	14.500059000	4.426209000	3.924129000
C	14.943507000	4.166724000	5.232558000

H	15.082175000	3.150479000	5.586082000
C	15.230257000	5.235620000	6.090677000
H	15.568325000	5.016874000	7.103407000
C	15.101556000	6.568684000	5.681603000
C	14.688335000	6.808805000	4.356591000
H	14.588125000	7.828728000	3.989641000
C	14.386250000	5.762076000	3.491044000
H	14.061607000	5.955534000	2.472237000
C	15.377557000	7.700003000	6.652547000
H	15.430950000	7.278893000	7.666375000
H	16.362231000	8.151284000	6.460124000
C	14.295914000	8.805564000	6.611014000
H	14.516238000	9.519112000	5.808982000
H	13.329120000	8.360268000	6.367767000
C	14.171160000	9.547779000	7.966327000
H	14.034419000	8.817112000	8.774518000
H	15.096351000	10.095797000	8.182467000
C	13.007284000	10.497689000	8.008901000
C	12.941859000	11.686669000	7.262537000
C	11.785162000	12.461977000	7.257271000
C	10.735029000	12.115638000	8.110494000
C	10.865908000	10.974935000	8.905368000
N	9.491824000	9.177912000	9.611981000
N	11.937323000	10.155505000	8.767998000
O	14.152926000	3.476117000	3.002849000
C	10.580412000	1.565670000	4.771397000
H	10.649195000	0.487283000	4.963068000
H	9.582755000	1.779109000	4.371699000
O	10.720539000	2.193213000	6.087895000
C	10.239771000	10.598818000	0.531378000
H	9.348802000	10.603760000	1.171874000
H	10.051762000	9.921489000	-0.308969000
H	10.373794000	11.607943000	0.123371000
C	11.732334000	11.222259000	2.454406000
H	11.873930000	12.213408000	2.009390000
H	12.631350000	10.998274000	3.042716000
H	10.874342000	11.288335000	3.136099000
C	11.500043000	10.182937000	1.336900000
C	10.241440000	5.694954000	-0.941674000
H	11.056698000	6.256229000	-1.413278000

H	9.345371000	6.328122000	-0.924078000
H	10.029688000	4.832633000	-1.584357000
C	10.621351000	5.201133000	0.471712000
C	11.926615000	4.365558000	0.376426000
H	11.776986000	3.512346000	-0.297399000
H	12.237488000	3.981790000	1.354109000
H	12.749989000	4.971773000	-0.021969000
C	10.866862000	6.367149000	1.447940000
C	11.046430000	7.688916000	1.017160000
H	10.991225000	7.900922000	-0.044494000
C	11.296340000	8.764132000	1.896511000
C	11.364920000	8.487300000	3.264690000
H	11.560557000	9.268663000	3.988658000
C	11.189275000	7.170631000	3.705211000
C	11.799143000	5.789380000	5.724920000
H	12.203760000	4.953803000	5.176468000
C	11.745496000	6.067436000	7.078639000
C	12.136240000	5.369933000	8.301221000
C	12.603387000	4.054566000	8.411585000
H	12.721256000	3.434918000	7.528112000
C	12.873737000	3.558232000	9.690997000
H	13.236236000	2.539334000	9.806967000
C	12.663919000	4.350784000	10.828859000
H	12.865048000	3.963711000	11.824156000
C	12.198574000	5.657926000	10.648517000
C	11.883995000	6.652239000	11.682939000
C	11.650102000	6.638724000	13.051696000
H	11.723747000	5.866958000	13.803873000
C	10.948912000	6.116655000	2.828963000
H	10.814158000	5.112131000	3.212918000
C	10.797669000	8.440388000	14.619261000
C	9.898016000	7.672841000	15.362492000
H	9.576127000	6.706414000	14.989523000
C	9.374275000	8.200830000	16.550492000
C	9.794078000	9.486640000	16.931889000
H	9.386295000	9.907584000	17.846151000
C	10.698840000	10.265586000	16.189520000
C	11.206878000	9.718158000	15.003273000
H	11.909883000	10.262692000	14.383416000
C	11.080321000	11.674969000	16.674479000

C	12.160515000	12.326827000	15.784365000
H	12.408295000	13.318293000	16.179886000
H	11.812521000	12.464822000	14.752969000
H	13.086512000	11.736865000	15.769449000
C	11.623612000	11.595336000	18.125647000
H	12.507608000	10.947719000	18.180935000
H	10.873286000	11.208335000	18.824052000
H	11.910027000	12.596434000	18.469847000
C	8.349706000	7.438639000	17.408743000
C	7.050213000	8.282268000	17.509716000
H	6.625730000	8.468509000	16.515369000
H	7.229997000	9.251219000	17.988746000
H	6.303099000	7.747243000	18.108460000
C	7.993392000	6.062047000	16.807637000
H	7.531817000	6.155320000	15.815658000
H	7.273235000	5.554475000	17.459162000
H	8.875053000	5.412330000	16.726911000
C	8.942379000	7.220579000	18.826614000
H	8.222366000	6.680927000	19.453717000
H	9.171245000	8.170469000	19.322801000
H	9.866955000	6.631074000	18.780352000
C	9.470863000	4.300230000	0.991850000
H	9.288438000	3.486764000	0.279954000
H	8.540997000	4.871629000	1.104053000
H	9.709206000	3.838303000	1.957881000
C	12.739163000	10.180345000	0.401066000
H	12.606275000	9.501706000	-0.449238000
H	13.641385000	9.871762000	0.944735000
H	12.908880000	11.187463000	0.001419000
N	11.295003000	6.901810000	5.112043000
N	10.927948000	7.844333000	6.022652000
N	11.210295000	7.329435000	7.203972000
N	11.968809000	6.131023000	9.403152000
N	11.641479000	7.935482000	11.255512000
N	11.261682000	8.702976000	12.257754000
N	11.263441000	7.914809000	13.361505000
C	9.809453000	12.567623000	16.644159000
H	10.054270000	13.576457000	16.998279000
H	9.016740000	12.167754000	17.286864000
H	9.410895000	12.648289000	15.625733000

Zn	11.327006000	8.131585000	9.132771000
N	14.101858000	12.079348000	6.401516000
O	15.174930000	12.247299000	6.978805000
O	13.873025000	12.174266000	5.193528000
N	11.674573000	13.618042000	6.300216000
O	12.405864000	14.573502000	6.528537000
O	10.885434000	13.446682000	5.375204000
N	9.462523000	12.903834000	8.126612000
O	8.435235000	12.252727000	8.365943000
O	9.542577000	14.104602000	7.898901000
N	9.982213000	12.358480000	11.523321000
O	10.077078000	12.464375000	12.746863000
O	10.404084000	13.162053000	10.684851000
N	7.831839000	11.064388000	13.049998000
O	8.040498000	10.469060000	14.105220000
O	7.176417000	12.082757000	12.858635000
N	7.060149000	8.408177000	12.343273000
O	7.516333000	7.397730000	12.890617000
O	5.921782000	8.862032000	12.433540000

1•4^{-}····Zn^{2+}

Zn	6.718376000	11.797349000	18.309203000
O	8.568890000	9.657944000	26.236490000
O	6.263655000	13.682627000	24.585503000
N	5.792644000	9.911474000	18.065022000
N	4.776088000	12.422072000	17.927644000
N	6.634637000	12.032719000	20.392984000
N	5.536998000	11.433388000	20.845124000
N	5.814418000	11.059872000	22.111039000
C	4.371144000	13.718092000	17.896810000
C	3.067870000	14.035332000	17.513134000
H	2.744309000	15.071003000	17.487653000
C	2.183280000	13.005650000	17.164523000
H	1.166005000	13.238597000	16.857909000
C	2.609590000	11.681914000	17.218384000
H	1.926352000	10.881070000	16.955648000
C	3.926217000	11.401897000	17.617971000
C	4.474356000	10.030622000	17.713909000
C	3.706815000	8.887401000	17.443591000
H	2.662918000	8.977730000	17.162041000

C	4.289684000	7.628422000	17.549847000
H	3.699810000	6.734267000	17.362737000
C	5.644451000	7.528342000	17.886611000
H	6.131013000	6.559273000	17.960550000
C	6.385231000	8.688149000	18.125435000
C	7.858274000	8.640865000	18.448367000
H	8.352129000	9.419300000	17.845386000
H	8.267601000	7.673334000	18.130723000
C	8.201852000	8.894280000	19.935457000
H	9.262207000	9.169885000	19.997039000
H	7.644064000	9.766410000	20.282581000
C	7.923744000	7.714473000	20.892579000
H	6.883774000	7.377996000	20.778346000
H	8.562442000	6.862934000	20.610384000
C	8.160494000	8.110922000	22.340579000
C	9.393529000	8.637582000	22.756725000
H	10.228614000	8.667375000	22.058771000
C	9.582136000	9.149178000	24.044772000
H	10.551642000	9.550516000	24.323907000
C	8.518256000	9.135094000	24.962433000
C	7.305000000	8.535868000	24.589042000
H	6.496151000	8.497436000	25.311800000
C	7.135925000	8.036682000	23.297461000
H	6.173427000	7.609479000	23.024429000
C	9.564746000	10.665278000	26.553376000
H	10.576971000	10.266343000	26.395713000
H	9.429489000	10.824706000	27.628551000
C	9.369021000	11.977766000	25.778232000
H	10.092742000	12.706493000	26.171722000
H	9.634691000	11.827355000	24.723861000
C	7.933282000	12.522750000	25.858153000
H	7.241313000	11.714345000	25.597379000
H	7.682196000	12.818934000	26.885338000
C	7.681284000	13.680028000	24.885973000
H	7.974590000	14.650921000	25.312086000
H	8.245914000	13.533379000	23.953816000
C	5.821379000	14.345679000	23.458696000
C	4.514941000	14.041872000	23.044966000
H	3.930218000	13.328487000	23.619277000
C	3.989242000	14.640573000	21.902475000

H	2.979435000	14.380348000	21.587229000
C	4.725950000	15.572832000	21.152555000
C	6.025551000	15.870950000	21.588571000
H	6.626709000	16.598655000	21.044944000
C	6.582500000	15.265580000	22.722616000
H	7.594552000	15.520348000	23.022442000
C	4.107003000	16.236380000	19.933882000
H	3.918946000	17.299620000	20.146083000
H	3.122039000	15.783680000	19.761577000
C	4.956268000	16.152426000	18.642708000
H	4.399438000	16.603388000	17.809597000
H	5.857253000	16.767374000	18.762209000
C	5.422863000	14.733641000	18.282576000
H	6.152836000	14.784177000	17.458317000
H	5.964309000	14.320887000	19.143111000
C	7.083155000	11.419409000	22.470165000
H	7.496542000	11.165894000	23.431798000
C	7.615455000	12.061818000	21.362497000
C	8.919279000	12.685567000	21.123136000
C	8.985523000	13.822887000	20.292589000
H	8.062117000	14.226003000	19.894920000
C	10.206014000	14.447918000	20.031272000
C	11.365368000	13.898293000	20.614348000
H	12.316828000	14.378290000	20.414637000
C	11.339233000	12.762519000	21.432842000
C	10.090773000	12.164516000	21.687415000
H	10.024264000	11.267232000	22.296768000
C	10.319354000	15.711402000	19.158870000
C	8.963424000	16.117161000	18.543624000
H	8.561641000	15.325531000	17.899145000
H	9.091919000	17.016606000	17.929819000
H	8.219390000	16.346097000	19.316416000
C	11.317811000	15.457398000	17.999019000
H	12.321823000	15.218787000	18.367529000
H	11.398661000	16.352157000	17.368559000
H	10.980401000	14.623314000	17.370256000
C	10.829715000	16.883670000	20.038668000
H	10.134816000	17.078038000	20.865743000
H	10.917000000	17.799230000	19.439543000
H	11.814428000	16.665472000	20.468182000

C	12.622634000	12.139487000	22.018054000
C	13.890131000	12.942730000	21.655715000
H	14.053601000	12.980772000	20.571608000
H	14.768240000	12.464084000	22.105588000
H	13.842053000	13.970995000	22.035397000
C	12.512559000	12.079117000	23.562665000
H	12.407702000	13.085157000	23.988232000
H	13.411261000	11.616698000	23.990488000
H	11.646797000	11.488884000	23.879001000
C	12.779086000	10.701299000	21.456659000
H	11.921955000	10.071728000	21.721093000
H	13.683345000	10.229619000	21.861935000
H	12.860669000	10.718798000	20.362641000
C	4.823936000	10.363424000	22.877077000
C	4.068642000	9.376634000	22.235457000
H	4.255370000	9.180081000	21.186593000
C	3.118819000	8.660846000	22.967139000
C	2.955302000	8.980213000	24.332646000
H	2.215719000	8.428344000	24.902957000
C	3.696750000	9.978997000	24.977298000
C	4.654920000	10.676337000	24.219108000
H	5.241896000	11.477753000	24.652898000
C	2.272373000	7.541170000	22.334594000
C	2.599147000	7.341426000	20.838951000
H	2.408899000	8.250399000	20.254602000
H	1.969692000	6.541960000	20.429607000
H	3.646374000	7.051056000	20.686182000
C	0.769445000	7.903458000	22.464159000
H	0.467248000	8.009796000	23.512199000
H	0.149346000	7.117810000	22.013734000
H	0.551734000	8.849801000	21.952642000
C	2.554215000	6.209581000	23.079866000
H	3.614273000	5.935458000	23.002169000
H	1.957618000	5.396902000	22.646119000
H	2.302063000	6.280430000	24.143890000
C	3.511806000	10.334393000	26.465001000
C	2.288713000	9.627722000	27.087333000
H	1.360935000	9.885498000	26.560797000
H	2.178242000	9.941476000	28.132292000
H	2.398764000	8.536037000	27.083288000

C	4.782140000	9.901667000	27.244056000
H	4.928851000	8.815858000	27.181266000
H	4.684144000	10.168540000	28.304171000
H	5.683894000	10.387209000	26.854667000
C	3.318697000	11.867372000	26.612379000
H	4.194975000	12.429568000	26.271126000
H	3.152033000	12.121891000	27.666569000
H	2.447671000	12.208909000	26.038360000

2•4$^{-}\cdots$Zn^{2+}

O	-4.075406000	-3.253484000	-1.515220000
O	-4.166895000	0.797442000	-2.862553000
N	2.908780000	1.673448000	0.866931000
N	2.248680000	-0.302958000	2.596184000
C	3.196233000	2.602566000	-0.078692000
C	4.267123000	3.477684000	0.081713000
H	4.486478000	4.206376000	-0.693692000
C	5.049861000	3.405692000	1.252106000
H	5.892540000	4.078959000	1.391113000
C	4.738921000	2.472067000	2.225853000
H	5.336049000	2.414367000	3.129906000
C	3.658169000	1.585540000	2.032720000
C	3.276250000	0.552384000	2.978573000
C	3.873932000	0.391317000	4.248017000
H	4.667168000	1.061724000	4.562441000
C	3.437474000	-0.608378000	5.098603000
H	3.889694000	-0.727879000	6.080364000
C	2.402834000	-1.473324000	4.680060000
H	2.046214000	-2.271091000	5.325622000
C	1.832927000	-1.300685000	3.422679000
C	0.783524000	-2.258026000	2.910253000
H	0.008467000	-1.715577000	2.356389000
H	0.291032000	-2.724081000	3.773508000
C	1.398265000	-3.371103000	2.025620000
H	2.294380000	-3.760177000	2.527107000
H	1.731011000	-2.942037000	1.070377000
C	0.440505000	-4.555066000	1.753946000
H	1.020958000	-5.359290000	1.282163000
H	0.089915000	-4.949170000	2.718892000
C	-0.750262000	-4.230118000	0.876157000

C	-1.962266000	-3.761319000	1.417182000
H	-2.059138000	-3.637561000	2.494818000
C	-3.056123000	-3.471290000	0.605728000
H	-3.990211000	-3.123603000	1.038161000
C	-2.966495000	-3.640225000	-0.786652000
C	-1.780941000	-4.137701000	-1.343996000
H	-1.692709000	-4.320896000	-2.409724000
C	-0.690251000	-4.415688000	-0.510606000
H	0.224765000	-4.799990000	-0.960422000
C	-4.070552000	-3.457282000	-2.947648000
H	-4.105746000	-4.535156000	-3.164073000
H	-3.135620000	-3.061585000	-3.371069000
C	-5.283113000	-2.740336000	-3.543196000
H	-6.198209000	-3.236187000	-3.194203000
H	-5.246170000	-2.883870000	-4.633400000
C	-5.373341000	-1.241392000	-3.190896000
H	-6.297443000	-0.823039000	-3.608722000
H	-5.446207000	-1.133818000	-2.103448000
C	-4.177857000	-0.407575000	-3.656368000
H	-3.231390000	-0.945704000	-3.497078000
H	-4.238415000	-0.157121000	-4.726778000
C	-3.012465000	1.550568000	-2.817045000
C	-1.889565000	1.347604000	-3.629104000
H	-1.882812000	0.580190000	-4.396986000
C	-0.763995000	2.170591000	-3.469145000
H	0.093558000	2.016261000	-4.123168000
C	-0.730981000	3.198841000	-2.520898000
C	-1.870021000	3.374516000	-1.708704000
H	-1.880352000	4.172075000	-0.965981000
C	-2.996293000	2.572156000	-1.851141000
H	-3.875263000	2.716132000	-1.227789000
C	0.445758000	4.146739000	-2.397542000
H	0.060665000	5.175697000	-2.364372000
H	1.068131000	4.079872000	-3.301333000
C	1.338914000	3.931898000	-1.153786000
H	1.951072000	4.830142000	-0.996950000
H	0.712008000	3.819862000	-0.257560000
C	2.281517000	2.712089000	-1.274722000
H	1.694919000	1.794009000	-1.392587000
H	2.890127000	2.820207000	-2.183308000

N	1.429132000	-0.796819000	-1.035827000
N	-0.479146000	0.181079000	0.563237000
N	-1.519193000	0.625538000	1.251056000
N	-2.613521000	0.213638000	0.553868000
C	-2.264687000	-0.492853000	-0.558822000
H	-3.005029000	-0.877544000	-1.237306000
C	-0.879856000	-0.512322000	-0.560382000
C	0.138294000	-1.011517000	-1.464994000
C	-0.121956000	-1.578198000	-2.713359000
H	-1.148228000	-1.733841000	-3.025869000
C	0.944240000	-1.908315000	-3.547739000
H	0.763123000	-2.349631000	-4.525046000
C	2.256631000	-1.655474000	-3.115758000
H	3.110640000	-1.895191000	-3.742488000
C	2.477162000	-1.103081000	-1.850682000
C	3.845301000	-0.797744000	-1.363246000
C	4.333940000	-1.375226000	-0.179792000
H	3.697966000	-2.061232000	0.369833000
C	5.640930000	-1.114182000	0.253837000
C	6.415430000	-0.219786000	-0.504884000
H	7.428443000	-0.007213000	-0.176431000
C	5.950568000	0.392845000	-1.678133000
C	4.655313000	0.072035000	-2.108274000
H	4.247169000	0.512561000	-3.012283000
C	6.273015000	-1.825450000	1.465850000
C	7.379877000	-2.782400000	0.945421000
H	6.957782000	-3.529400000	0.261218000
H	7.850240000	-3.311140000	1.784734000
H	8.162605000	-2.235364000	0.406810000
C	5.242197000	-2.657152000	2.257088000
H	4.420457000	-2.038012000	2.628323000
H	5.729479000	-3.119313000	3.123969000
H	4.820528000	-3.465789000	1.647205000
C	6.906356000	-0.786452000	2.425973000
H	7.701972000	-0.211234000	1.939797000
H	7.345854000	-1.295264000	3.293222000
H	6.150410000	-0.082635000	2.787454000
C	6.873171000	1.332773000	-2.478371000
C	7.960701000	0.470935000	-3.174589000
H	8.554881000	-0.085585000	-2.439833000

H	8.642216000	1.109386000	-3.751899000
H	7.504846000	-0.254632000	-3.860183000
C	6.100591000	2.123843000	-3.557341000
H	5.670736000	1.465932000	-4.322601000
H	6.782710000	2.814826000	-4.066763000
H	5.289401000	2.716531000	-3.114552000
C	7.557874000	2.353892000	-1.534301000
H	6.811300000	2.948130000	-0.996113000
H	8.189960000	3.035257000	-2.117476000
H	8.198544000	1.864847000	-0.792344000
C	-3.933601000	0.604415000	0.957295000
C	-4.108405000	1.861507000	1.539988000
H	-3.240040000	2.489504000	1.696898000
C	-5.398737000	2.286119000	1.879714000
C	-6.468460000	1.410434000	1.623778000
H	-7.472637000	1.736350000	1.878143000
C	-6.302165000	0.137199000	1.055444000
C	-5.001670000	-0.259661000	0.712143000
H	-4.814507000	-1.228345000	0.265633000
C	-5.671176000	3.669773000	2.498459000
C	-4.376594000	4.488402000	2.691354000
H	-3.671032000	3.983751000	3.363484000
H	-4.621093000	5.460567000	3.135439000
H	-3.870154000	4.679096000	1.736156000
C	-6.618625000	4.466775000	1.562702000
H	-6.164013000	4.605954000	0.573430000
H	-6.823500000	5.457240000	1.988500000
H	-7.578436000	3.955866000	1.424633000
C	-6.345047000	3.482964000	3.883804000
H	-7.297639000	2.947030000	3.801884000
H	-6.547056000	4.460424000	4.339987000
H	-5.693980000	2.915593000	4.560768000
C	-7.526572000	-0.761905000	0.796004000
C	-8.338803000	-0.151897000	-0.378257000
H	-8.683516000	0.861107000	-0.138711000
H	-9.220441000	-0.769895000	-0.592522000
H	-7.728008000	-0.092877000	-1.287549000
C	-7.122387000	-2.206490000	0.427583000
H	-6.539446000	-2.258718000	-0.498198000
H	-8.024731000	-2.812177000	0.281583000

H	-6.534190000	-2.674755000	1.227475000
C	-8.419245000	-0.826319000	2.062487000
H	-7.856786000	-1.220169000	2.918409000
H	-9.275570000	-1.487851000	1.881486000
H	-8.815907000	0.156428000	2.340034000
Zn	1.563446000	0.186927000	0.769594000

3•4^{-}····Zn^{2+}

C	-1.965932000	3.213253000	0.856885000
C	-2.405798000	4.543544000	0.704761000
H	-2.913280000	5.048732000	1.520409000
C	-2.179018000	5.210994000	-0.490696000
H	-2.523556000	6.235046000	-0.617671000
C	-1.493174000	4.559451000	-1.530368000
H	-1.297371000	5.062267000	-2.473796000
C	-1.058900000	3.244843000	-1.340170000
C	-0.256657000	2.527605000	-2.399832000
H	-0.274601000	1.447849000	-2.206717000
H	-0.726944000	2.688110000	-3.380711000
C	1.204022000	3.036232000	-2.451852000
H	1.698056000	2.811279000	-1.497470000
H	1.182245000	4.130831000	-2.546351000
C	2.032970000	2.465796000	-3.628618000
H	1.450381000	2.578599000	-4.555829000
H	2.924764000	3.095074000	-3.748800000
C	2.484360000	1.025832000	-3.481617000
C	1.611183000	-0.060242000	-3.687164000
H	0.575503000	0.123931000	-3.967698000
C	2.049481000	-1.378170000	-3.577214000
H	1.371514000	-2.210529000	-3.748555000
C	3.389453000	-1.652755000	-3.258527000
C	4.278760000	-0.587553000	-3.053761000
H	5.319571000	-0.759981000	-2.798726000
C	3.816609000	0.729639000	-3.165726000
H	4.521236000	1.543247000	-3.001514000
C	5.675824000	-3.387897000	-1.674533000
H	5.574130000	-2.393429000	-1.222696000
H	6.756790000	-3.583793000	-1.738782000
C	5.012973000	-4.449207000	-0.780623000
H	3.921598000	-4.348228000	-0.830745000

H	5.248738000	-5.451985000	-1.169420000
C	5.470494000	-4.394792000	0.680718000
H	6.564991000	-4.356556000	0.739294000
H	5.132157000	-5.276136000	1.240392000
C	3.803093000	-3.202129000	2.007274000
C	2.815054000	-4.178744000	1.824450000
H	2.966879000	-5.016398000	1.151497000
C	1.604639000	-4.075207000	2.520414000
H	0.846902000	-4.842593000	2.365655000
C	1.343857000	-3.023942000	3.407501000
C	2.343295000	-2.045185000	3.562178000
H	2.186672000	-1.209096000	4.241467000
C	3.553684000	-2.126667000	2.876025000
H	4.322096000	-1.370103000	3.015454000
C	0.057134000	-3.010653000	4.217496000
H	-0.662493000	-3.690818000	3.739055000
H	0.261400000	-3.439620000	5.210989000
C	-0.611415000	-1.636034000	4.419842000
H	-1.499895000	-1.786328000	5.047224000
H	0.053299000	-0.967537000	4.984521000
C	-1.001009000	-0.966936000	3.094606000
H	-0.103809000	-0.855171000	2.473888000
H	-1.671681000	-1.637240000	2.531039000
C	-1.668147000	0.388782000	3.203430000
C	-2.359894000	0.818710000	4.333817000
H	-2.414574000	0.189988000	5.216135000
C	-2.988138000	2.081873000	4.317420000
H	-3.545355000	2.425455000	5.186243000
C	-2.893259000	2.881979000	3.190243000
H	-3.381099000	3.851491000	3.168460000
C	-2.161122000	2.428126000	2.073452000
N	-1.304695000	2.592573000	-0.183527000
N	-1.572976000	1.180834000	2.106062000
O	5.030734000	-3.192171000	1.367375000
C	5.117553000	-3.364809000	-3.100794000
H	5.146021000	-4.370696000	-3.538062000
H	5.701763000	-2.696295000	-3.746825000
O	3.717703000	-2.989926000	-3.149673000
C	7.347451000	4.306074000	-1.709322000
H	6.917050000	3.755355000	-2.555748000

H	8.312970000	3.848841000	-1.464413000
H	7.536186000	5.337889000	-2.032220000
C	5.078987000	5.017604000	-0.905258000
H	5.310109000	6.048736000	-1.198041000
H	4.357823000	5.059468000	-0.079275000
H	4.594995000	4.530693000	-1.762063000
C	6.380489000	4.294464000	-0.495409000
C	9.568407000	0.579894000	1.190754000
H	9.533220000	1.434692000	1.877661000
H	9.842880000	0.947164000	0.193749000
H	10.370694000	-0.086028000	1.530314000
C	8.232634000	-0.192787000	1.171211000
C	7.947516000	-0.720022000	2.603318000
H	8.791546000	-1.327064000	2.955468000
H	7.049847000	-1.348113000	2.631178000
H	7.808166000	0.112041000	3.304824000
C	7.052894000	0.685104000	0.711046000
C	7.207814000	2.023961000	0.332106000
H	8.200391000	2.460247000	0.345900000
C	6.126683000	2.837335000	-0.067728000
C	4.849501000	2.274307000	-0.075358000
H	3.977400000	2.841285000	-0.374613000
C	4.684007000	0.934558000	0.295973000
C	3.003925000	-0.885754000	-0.125819000
H	3.727377000	-1.643042000	-0.368218000
C	1.619881000	-0.879144000	-0.131855000
C	0.595737000	-1.841796000	-0.491821000
C	0.825834000	-3.157793000	-0.885920000
H	1.841634000	-3.533726000	-0.943118000
C	-0.265710000	-3.970314000	-1.224947000
H	-0.107743000	-4.998090000	-1.540771000
C	-1.565391000	-3.438128000	-1.167161000
H	-2.426549000	-4.043629000	-1.439701000
C	-1.741296000	-2.110994000	-0.777826000
C	-3.018637000	-1.417727000	-0.686769000
C	-4.349265000	-1.802548000	-0.778916000
H	-4.823400000	-2.758898000	-0.939186000
C	5.756757000	0.134924000	0.679740000
H	5.587520000	-0.897304000	0.970492000
C	-6.491964000	-0.493574000	-0.513466000

C	-7.302110000	-1.316735000	-1.306906000
H	-6.842025000	-2.043941000	-1.967234000
C	-8.691495000	-1.165730000	-1.261867000
C	-9.221360000	-0.166165000	-0.420234000
H	-10.298116000	-0.042561000	-0.387610000
C	-8.420534000	0.671999000	0.365502000
C	-7.027897000	0.488629000	0.314678000
H	-6.354945000	1.097870000	0.908490000
C	-9.000180000	1.773073000	1.273653000
C	-8.562285000	1.512211000	2.739372000
H	-8.971772000	2.288742000	3.398207000
H	-7.471048000	1.523049000	2.845335000
H	-8.928072000	0.538341000	3.088684000
C	-10.542271000	1.821633000	1.227870000
H	-10.989905000	0.880503000	1.571526000
H	-10.913102000	2.034229000	0.217358000
H	-10.902491000	2.620396000	1.887219000
C	-9.644816000	-2.034844000	-2.103676000
C	-10.465276000	-1.120440000	-3.051925000
H	-9.802677000	-0.566351000	-3.728786000
H	-11.065436000	-0.392275000	-2.494533000
H	-11.149988000	-1.725246000	-3.660099000
C	-8.890623000	-3.072697000	-2.962332000
H	-8.213415000	-2.593262000	-3.681068000
H	-9.611815000	-3.667312000	-3.535452000
H	-8.308395000	-3.767562000	-2.342920000
C	-10.608425000	-2.795176000	-1.153618000
H	-11.297157000	-3.420398000	-1.736015000
H	-11.209377000	-2.106034000	-0.549520000
H	-10.048640000	-3.445695000	-0.469562000
C	8.374264000	-1.398661000	0.206298000
H	9.218259000	-2.030649000	0.511582000
H	8.552628000	-1.059921000	-0.822439000
H	7.470486000	-2.015350000	0.212297000
C	7.021400000	5.069921000	0.685872000
H	7.976875000	4.627238000	0.989788000
H	6.355329000	5.071480000	1.557878000
H	7.210769000	6.111303000	0.395664000
N	3.365943000	0.375781000	0.242073000
N	2.271206000	1.157618000	0.479712000

N	1.225772000	0.386083000	0.249709000
N	-0.671956000	-1.323279000	-0.443120000
N	-3.007773000	-0.067450000	-0.409620000
N	-4.235858000	0.402153000	-0.329302000
N	-5.067265000	-0.661434000	-0.552807000
C	-8.451679000	3.148520000	0.807869000
H	-8.848454000	3.948533000	1.446207000
H	-8.749457000	3.356019000	-0.227647000
H	-7.357008000	3.186105000	0.858402000
Zn	-0.917551000	0.625304000	0.246917000

5•6$_{N,ac}$···Na^+

C	1.302005000	-3.561398000	1.957204000
C	0.912537000	-3.737746000	0.502856000
O	0.624360000	-2.760133000	-0.216048000
N	0.895323000	-4.997707000	0.013998000
H	2.215141000	-2.955366000	2.006286000
H	1.474565000	-4.506478000	2.481389000
H	0.509586000	-3.004829000	2.471023000
H	1.131903000	-5.800213000	0.584546000
H	0.649169000	-5.151395000	-0.959048000
Na	0.173030000	-0.739318000	-0.912617000
C	-2.120922000	-0.741659000	1.192227000
N	-2.226718000	-0.182990000	-0.175460000
C	-2.444589000	1.301310000	-0.118725000
C	-3.330913000	-0.843777000	-0.906557000
C	-1.147404000	1.993892000	0.232814000
C	-0.102978000	1.929843000	-0.697022000
C	1.208226000	2.298403000	-0.373970000
C	1.448470000	2.869263000	0.883685000
C	0.396761000	3.030233000	1.795383000
C	-0.888460000	2.565790000	1.486500000
C	2.303936000	1.910411000	-1.341026000
N	2.432216000	0.415442000	-1.384440000
C	3.108105000	-0.079401000	-0.163008000
C	3.186716000	-0.007626000	-2.585267000
H	-1.907911000	-1.814897000	1.129033000
H	-3.052826000	-0.602697000	1.769869000
H	-1.306129000	-0.250002000	1.736641000
H	-3.243246000	1.551816000	0.600522000

H	-2.783374000	1.616611000	-1.113414000
H	-3.376654000	-0.459047000	-1.931684000
H	-4.311700000	-0.677859000	-0.425862000
H	-3.146625000	-1.923892000	-0.943736000
H	-0.310844000	1.545043000	-1.698907000
H	2.457379000	3.176194000	1.155222000
H	0.587880000	3.484389000	2.765443000
H	-1.686241000	2.636912000	2.224167000
H	3.266363000	2.377579000	-1.069613000
H	2.047962000	2.242823000	-2.354657000
H	3.141942000	-1.174678000	-0.185755000
H	2.553587000	0.240519000	0.726899000
H	4.141789000	0.302270000	-0.077739000
H	3.250685000	-1.101893000	-2.606762000
H	4.214235000	0.398452000	-2.599036000
H	2.666240000	0.333089000	-3.487458000

5•6$_{N,c}$····Na^+

Na	-0.456344000	0.878381000	2.954466000
C	-1.789826000	-2.744411000	1.571174000
C	-0.419247000	-2.135788000	1.821890000
O	-0.283551000	-1.216620000	2.667018000
N	0.619646000	-2.603940000	1.103269000
C	-2.769672000	-1.697901000	0.986318000
N	-2.501341000	-1.413959000	-0.433850000
C	-2.556433000	0.017926000	-0.769293000
C	-3.309162000	-2.250120000	-1.333091000
C	-1.324271000	0.754863000	-0.263490000
C	-0.067396000	0.151743000	-0.419471000
C	1.105005000	0.743319000	0.062733000
C	1.020602000	2.010853000	0.673523000
C	-0.225106000	2.652732000	0.797261000
C	-1.400010000	2.019381000	0.345605000
C	2.448288000	0.047625000	-0.109222000
N	2.362303000	-1.420490000	-0.139219000
C	3.521043000	-2.049452000	-0.796899000
C	1.990419000	-2.040878000	1.128002000
H	-2.169432000	-3.091203000	2.541995000
H	-1.728819000	-3.612873000	0.904862000
H	0.422150000	-3.294588000	0.385308000

H	-2.673932000	-0.771337000	1.566360000
H	-3.804048000	-2.052924000	1.134656000
H	-3.474084000	0.512890000	-0.395005000
H	-2.586186000	0.097847000	-1.864741000
H	-2.979179000	-2.105498000	-2.367940000
H	-4.392204000	-2.027439000	-1.273324000
H	-3.167256000	-3.307321000	-1.079248000
H	-0.010766000	-0.822425000	-0.893496000
H	1.924271000	2.506280000	1.029241000
H	-0.278735000	3.652596000	1.227326000
H	-2.363720000	2.517109000	0.451639000
H	3.147844000	0.405664000	0.672897000
H	2.878737000	0.366202000	-1.069108000
H	3.360705000	-3.130802000	-0.868730000
H	4.474334000	-1.872019000	-0.264503000
H	3.616523000	-1.657080000	-1.815195000
H	2.679761000	-2.860595000	1.362923000
H	2.008893000	-1.331720000	1.962891000

5•6$_{N}$···Na^{+}

O	9.691159000	9.131006000	8.415612000
O	9.633237000	11.983581000	10.044194000
O	9.144363000	12.297159000	13.015544000
O	8.024497000	11.648916000	15.650064000
O	8.941379000	7.277190000	12.192628000
N	12.729941000	2.742277000	9.605208000
N	8.528231000	4.277919000	15.078864000
N	9.430724000	9.431654000	12.799134000
H	9.127894000	10.409672000	12.746827000
C	10.350086000	2.887041000	12.597025000
H	11.042250000	2.705470000	13.420317000
C	8.989917000	3.096345000	12.888040000
C	8.101440000	3.325089000	11.820892000
H	7.043554000	3.477182000	12.026248000
C	8.564037000	3.323963000	10.500289000
H	7.866664000	3.492053000	9.681537000
C	9.917537000	3.082554000	10.224487000
H	10.285559000	3.046090000	9.204380000
C	10.830067000	2.872486000	11.271597000
C	12.310997000	2.597810000	11.007480000

H	12.912601000	3.251578000	11.663750000
H	12.525542000	1.573370000	11.346036000
C	13.340814000	1.520749000	9.060060000
H	13.521593000	1.651186000	7.986938000
H	12.650174000	0.679502000	9.187203000
H	14.302338000	1.258939000	9.544927000
C	13.550704000	3.938520000	9.357267000
H	13.969295000	3.837663000	8.346688000
H	14.413937000	3.997006000	10.051779000
C	12.771534000	5.241754000	9.422547000
C	11.660015000	5.453407000	8.587765000
H	11.341374000	4.655625000	7.920425000
C	10.966443000	6.668942000	8.593209000
H	10.112747000	6.821076000	7.938482000
C	11.379851000	7.722680000	9.428531000
C	12.498311000	7.522885000	10.258313000
H	12.835744000	8.322236000	10.912857000
C	13.176415000	6.295179000	10.260547000
H	14.045089000	6.165106000	10.905561000
C	10.643397000	9.043863000	9.478620000
H	10.129440000	9.144874000	10.443170000
H	11.370475000	9.869552000	9.425963000
C	8.708538000	10.166319000	8.594452000
H	8.086899000	9.952322000	9.480414000
H	8.073911000	10.095704000	7.702586000
C	9.293431000	11.583177000	8.696322000
H	10.219103000	11.638165000	8.108454000
H	8.580708000	12.305913000	8.268605000
C	8.584778000	12.725267000	10.690076000
H	7.704250000	12.088947000	10.875772000
H	8.269801000	13.560707000	10.040660000
C	9.087919000	13.310437000	11.995448000
H	10.086043000	13.754564000	11.843979000
H	8.399974000	14.109287000	12.319454000
C	9.610711000	12.810317000	14.281451000
H	9.043806000	13.719686000	14.537143000
H	10.679408000	13.069812000	14.203737000
C	9.422618000	11.773095000	15.371649000
H	9.861961000	10.809372000	15.072592000
H	9.960030000	12.118786000	16.273904000

C	7.716708000	10.692741000	16.697135000
H	6.726681000	10.989871000	17.061631000
H	8.437773000	10.805318000	17.522890000
C	7.689586000	9.268616000	16.191847000
C	6.544738000	8.776268000	15.545052000
H	5.672260000	9.418164000	15.435716000
C	6.509976000	7.477426000	15.041140000
H	5.611452000	7.117974000	14.540867000
C	7.621897000	6.629127000	15.160440000
C	8.762739000	7.115933000	15.811098000
H	9.624684000	6.463611000	15.924475000
C	8.795606000	8.416809000	16.322695000
H	9.689759000	8.769285000	16.836249000
C	7.564056000	5.249093000	14.535492000
H	6.525009000	4.862696000	14.616417000
H	7.766780000	5.365208000	13.462718000
C	8.249020000	3.981728000	16.498159000
H	8.277513000	4.900893000	17.088749000
H	9.011752000	3.295447000	16.883324000
H	7.253642000	3.514755000	16.637259000
C	8.489551000	3.007574000	14.316434000
H	7.460399000	2.591310000	14.308754000
H	9.115602000	2.291804000	14.862579000
C	8.576799000	8.465931000	12.355079000
C	7.197551000	8.891509000	11.988584000
C	6.499672000	8.050346000	11.108746000
H	7.000358000	7.151406000	10.770359000
C	5.211451000	8.372876000	10.673510000
C	4.639358000	9.557699000	11.167180000
H	3.635176000	9.821208000	10.845017000
C	5.303024000	10.417303000	12.061168000
C	6.600050000	10.070286000	12.465334000
H	7.124671000	10.694167000	13.180828000
C	4.436237000	7.493760000	9.674074000
C	4.175923000	8.312010000	8.381596000
H	3.584430000	9.211881000	8.586723000
H	3.624836000	7.705919000	7.651077000
H	5.122228000	8.625753000	7.922142000
C	3.081905000	7.064936000	10.296969000
H	2.451978000	7.928842000	10.537904000

H	3.241201000	6.493511000	11.220331000
H	2.525879000	6.431851000	9.593528000
C	5.221050000	6.218351000	9.294750000
H	6.180981000	6.457812000	8.819637000
H	4.635523000	5.624506000	8.582755000
H	5.417484000	5.589344000	10.173010000
C	4.583673000	11.681504000	12.571576000
H	2.633484000	10.668962000	12.779169000
C	4.141395000	12.547348000	11.362455000
H	5.008611000	12.843290000	10.757909000
H	3.642811000	13.459184000	11.714550000
H	3.440311000	12.016433000	10.708639000
C	3.333744000	11.254819000	13.385988000
H	3.622129000	10.644133000	14.251145000
H	2.801329000	12.140587000	13.755522000
C	5.484067000	12.546468000	13.478515000
H	5.830778000	12.007700000	14.365578000
H	4.922636000	13.425803000	13.816906000
H	6.372348000	12.901599000	12.944262000
C	10.821411000	9.256951000	13.042577000
C	11.316766000	8.120671000	13.693476000
H	10.609867000	7.397766000	14.077896000
C	12.695425000	7.954399000	13.882696000
C	13.558662000	8.970834000	13.436080000
H	14.625660000	8.855390000	13.577913000
C	13.079715000	10.134826000	12.809000000
C	11.696950000	10.261344000	12.612371000
H	11.284000000	11.121594000	12.095103000
C	13.204471000	6.695147000	14.612885000
C	14.729297000	6.504630000	14.464702000
H	15.289917000	7.323481000	14.929728000
H	15.028348000	6.440782000	13.410446000
H	15.036156000	5.575695000	14.960509000
C	12.506952000	5.429912000	14.046206000
H	11.422500000	5.439573000	14.214600000
H	12.892779000	4.529416000	14.540111000
H	12.716114000	5.328098000	12.971279000
C	12.860252000	6.827572000	16.120754000
H	11.780101000	6.934820000	16.270194000
H	13.350670000	7.708615000	16.552238000

H	13.197093000	5.940541000	16.673035000
C	14.011536000	11.256585000	12.308900000
C	15.502134000	10.933225000	12.543966000
H	15.731296000	10.811586000	13.610094000
H	16.120748000	11.754961000	12.164045000
H	15.805926000	10.019159000	12.018136000
C	13.666416000	12.567536000	13.063619000
H	13.788690000	12.438076000	14.146535000
H	12.633022000	12.879118000	12.871574000
H	14.327514000	13.380719000	12.738230000
C	13.791452000	11.468481000	10.786766000
H	12.753642000	11.738756000	10.558982000
H	14.038017000	10.557352000	10.226190000
H	14.438058000	12.276209000	10.420854000
Na	10.389489000	5.792578000	11.658558000

5•6$_{O,ac}$···Na^+

C	0.164220000	-0.346797000	-3.235965000
C	-0.530270000	-0.158814000	-1.902932000
O	0.105616000	-0.194594000	-0.831490000
N	-1.871389000	0.052943000	-1.921660000
H	0.944525000	0.416848000	-3.337846000
H	-0.514200000	-0.285846000	-4.093003000
H	0.661591000	-1.324105000	-3.239296000
H	-2.395736000	0.059271000	-2.787572000
H	-2.363612000	0.165745000	-1.034640000
C	-3.582541000	-0.778907000	1.443234000
O	-2.735350000	0.326492000	1.044322000
C	-3.240162000	1.606244000	1.501670000
C	-2.273420000	2.695069000	1.051100000
O	-0.944284000	2.501420000	1.577512000
C	-0.747705000	3.003182000	2.920067000
C	0.608084000	2.521518000	3.399955000
O	0.575618000	1.081252000	3.409815000
C	1.823424000	0.455311000	3.777861000
C	1.645806000	-1.042490000	3.561598000
O	1.315634000	-1.342928000	2.186534000
C	2.467582000	-1.579629000	1.342125000
H	-3.117832000	-1.689888000	1.054361000
H	-4.590407000	-0.668467000	1.018569000

H	-3.653409000	-0.835795000	2.539071000
H	-3.343583000	1.582003000	2.598114000
H	-4.236271000	1.793629000	1.068802000
H	-2.667983000	3.681240000	1.339205000
H	-2.163040000	2.673501000	-0.039775000
H	-1.534371000	2.628655000	3.593075000
H	-0.777723000	4.103871000	2.917404000
H	0.804128000	2.912565000	4.411054000
H	1.404876000	2.875427000	2.724942000
H	2.060623000	0.661571000	4.834220000
H	2.639449000	0.856582000	3.153983000
H	0.803405000	-1.405116000	4.164039000
H	2.552607000	-1.582345000	3.870149000
H	2.082816000	-1.730719000	0.330325000
H	3.007926000	-2.474469000	1.680909000
H	3.150194000	-0.716749000	1.341791000
Na	-0.284448000	0.184972000	1.339150000

5•6$_{O,c}$···Na^+

C	-2.452074000	-1.722870000	1.057199000
O	-2.194895000	-0.618362000	0.154665000
C	-3.091735000	-0.572436000	-0.976976000
C	-2.880944000	0.748752000	-1.695658000
O	-1.503280000	0.844425000	-2.137622000
C	-1.019607000	2.200462000	-2.296336000
C	0.446587000	2.253014000	-1.891422000
O	0.520423000	1.775288000	-0.528359000
C	1.773829000	1.973885000	0.154746000
C	2.814408000	0.913569000	-0.215359000
O	2.284901000	-0.433105000	-0.130036000
C	2.341187000	-1.092172000	1.152724000
C	-1.325989000	-1.745784000	2.095074000
C	0.018253000	-1.852196000	1.380607000
O	0.153867000	-2.532984000	0.353046000
N	1.054903000	-1.082977000	1.846365000
H	-2.464451000	-2.666267000	0.493285000
H	-3.426202000	-1.582280000	1.547755000
H	-4.139710000	-0.627828000	-0.642633000
H	-2.900703000	-1.431722000	-1.643103000
H	-3.095193000	1.575838000	-1.003690000

H	-3.562933000	0.818281000	-2.556800000
H	-1.596713000	2.870575000	-1.644692000
H	-1.138103000	2.527961000	-3.340053000
H	0.808479000	3.290485000	-1.959841000
H	1.066596000	1.632485000	-2.561733000
H	1.524993000	1.918797000	1.221689000
H	2.176345000	2.978745000	-0.049995000
H	3.703085000	1.019599000	0.424797000
H	3.137984000	1.023461000	-1.257228000
H	3.090609000	-0.621217000	1.797976000
H	2.612878000	-2.128882000	0.938465000
H	-1.453638000	-2.627439000	2.739119000
H	-1.368662000	-0.856105000	2.735958000
H	0.914330000	-0.480560000	2.650915000
Na	-0.018900000	-0.552438000	-0.974247000

5•6$_{O\cdots}$Na$^+$

Na	-8.156831000	-11.877933000	-4.594707000
O	-10.468802000	-11.545116000	-4.237862000
O	-8.347355000	-9.987009000	-3.085275000
O	-7.064789000	-12.482192000	-2.539786000
O	-3.581987000	-12.826913000	-3.462696000
O	-7.503761000	-13.684130000	-5.686354000
N	-10.610502000	-14.643288000	-10.546280000
N	-5.172014000	-12.848975000	-10.225472000
N	-6.964862000	-13.120066000	-7.826316000
H	-6.441941000	-13.335830000	-8.694374000
C	-7.165981000	-14.035333000	-6.843294000
C	-8.167249000	-13.137934000	-11.240619000
H	-8.074976000	-13.933228000	-10.508128000
C	-7.031196000	-12.638712000	-11.891612000
C	-7.189848000	-11.615722000	-12.838365000
H	-6.315505000	-11.218951000	-13.352513000
C	-8.461912000	-11.114174000	-13.131087000
H	-8.578361000	-10.326918000	-13.873562000
C	-9.586986000	-11.638326000	-12.487427000
H	-10.579374000	-11.262987000	-12.734855000
C	-9.449562000	-12.656598000	-11.532220000
C	-10.664583000	-13.195616000	-10.802794000
H	-11.581245000	-12.915071000	-11.363656000

H	-10.738550000	-12.699662000	-9.828130000
C	-10.709860000	-15.413169000	-11.795368000
H	-9.906806000	-15.118595000	-12.478242000
H	-10.605807000	-16.482203000	-11.577163000
H	-11.678440000	-15.254814000	-12.311996000
C	-11.675198000	-15.061109000	-9.609094000
H	-12.680090000	-14.848384000	-10.030021000
H	-11.598718000	-16.151574000	-9.504284000
C	-11.568581000	-14.417249000	-8.241087000
C	-12.460437000	-13.404041000	-7.855763000
H	-13.223447000	-13.068884000	-8.557012000
C	-12.389628000	-12.832458000	-6.583855000
H	-13.094410000	-12.050916000	-6.302199000
C	-11.435469000	-13.276081000	-5.655312000
C	-10.541120000	-14.289276000	-6.037549000
H	-9.794548000	-14.650583000	-5.334192000
C	-10.596460000	-14.839774000	-7.320813000
H	-9.891035000	-15.618372000	-7.604294000
C	-11.400985000	-12.680359000	-4.274481000
H	-11.076596000	-13.417337000	-3.523228000
H	-12.397268000	-12.311875000	-3.992827000
C	-10.741954000	-10.633818000	-3.155304000
H	-11.746828000	-10.195864000	-3.278441000
H	-10.724642000	-11.168432000	-2.190531000
C	-9.707727000	-9.514755000	-3.191937000
H	-9.924335000	-8.786721000	-2.395496000
H	-9.754610000	-9.000806000	-4.157640000
C	-7.873284000	-10.292031000	-1.751057000
H	-6.874247000	-9.845575000	-1.672575000
H	-8.515453000	-9.818921000	-0.993729000
C	-7.808610000	-11.802789000	-1.509681000
H	-8.812250000	-12.244824000	-1.532805000
H	-7.368904000	-12.002959000	-0.519086000
C	-5.627600000	-12.408154000	-2.367239000
H	-5.282022000	-11.366948000	-2.419324000
H	-5.359271000	-12.816670000	-1.381550000
C	-4.962825000	-13.203651000	-3.486006000
H	-5.429633000	-12.972688000	-4.455095000
H	-5.078464000	-14.287603000	-3.317401000
C	-2.721021000	-13.707162000	-4.224049000

H	-2.832812000	-14.739074000	-3.852367000
H	-1.707762000	-13.359477000	-3.993403000
C	-2.980736000	-13.664512000	-5.715221000
C	-2.579558000	-12.558180000	-6.483474000
H	-2.055122000	-11.733554000	-6.002349000
C	-2.831666000	-12.513994000	-7.856858000
H	-2.486860000	-11.660339000	-8.438209000
C	-3.508767000	-13.565802000	-8.498980000
C	-3.883737000	-14.678049000	-7.733558000
H	-4.391351000	-15.511710000	-8.212315000
C	-3.626508000	-14.726583000	-6.361812000
H	-3.931605000	-15.601414000	-5.791305000
C	-3.861076000	-13.517435000	-9.971175000
H	-3.944510000	-14.542325000	-10.351261000
H	-3.059222000	-13.017700000	-10.547264000
C	-5.046748000	-11.382572000	-10.097583000
H	-6.030237000	-10.916108000	-10.198748000
H	-4.648627000	-11.130009000	-9.114021000
H	-4.373888000	-10.963609000	-10.870705000
C	-5.660227000	-13.203910000	-11.586484000
H	-4.942089000	-12.856895000	-12.354120000
H	-5.689383000	-14.299029000	-11.639871000
C	-7.007389000	-15.481790000	-7.166194000
C	-7.098336000	-15.984251000	-8.476218000
H	-7.298797000	-15.300806000	-9.292221000
C	-6.978463000	-17.355400000	-8.722896000
C	-6.744224000	-18.205621000	-7.621936000
H	-6.650391000	-19.270263000	-7.806730000
C	-6.644676000	-17.735375000	-6.305966000
C	-6.795481000	-16.356461000	-6.097015000
H	-6.745176000	-15.931657000	-5.099373000
C	-7.138834000	-17.965086000	-10.128069000
C	-8.421948000	-18.839782000	-10.142485000
H	-8.565753000	-19.289054000	-11.133633000
H	-8.364389000	-19.650553000	-9.407006000
H	-9.305477000	-18.232390000	-9.908054000
C	-7.275851000	-16.882204000	-11.217208000
H	-6.372472000	-16.262380000	-11.277605000
H	-7.416559000	-17.356197000	-12.195964000
H	-8.134171000	-16.227647000	-11.035948000

C	-5.907180000	-18.843859000	-10.467799000
H	-4.985811000	-18.247569000	-10.446300000
H	-5.786792000	-19.676368000	-9.765226000
H	-6.015219000	-19.270600000	-11.473030000
C	-6.380653000	-18.659588000	-5.101980000
C	-6.264641000	-20.143029000	-5.512166000
H	-7.187845000	-20.506777000	-5.980768000
H	-5.433542000	-20.310484000	-6.208852000
H	-6.080526000	-20.757255000	-4.622637000
C	-7.547537000	-18.522042000	-4.088409000
H	-7.648535000	-17.492957000	-3.723688000
H	-8.499233000	-18.813204000	-4.550737000
H	-7.375039000	-19.171743000	-3.220652000
C	-5.052433000	-18.233042000	-4.421464000
H	-5.101105000	-17.200454000	-4.054043000
H	-4.837701000	-18.884436000	-3.564671000
H	-4.212889000	-18.305211000	-5.124893000
C	-7.182813000	-11.721870000	-7.561782000
C	-6.208974000	-10.976042000	-6.894266000
H	-5.283772000	-11.467486000	-6.606136000
C	-6.428318000	-9.613184000	-6.611907000
C	-7.646126000	-9.048924000	-7.020739000
H	-7.828462000	-8.000050000	-6.814289000
C	-8.639162000	-9.781183000	-7.703912000
C	-8.391056000	-11.136968000	-7.962056000
H	-9.117321000	-11.762921000	-8.467661000
C	-5.329905000	-8.799968000	-5.900129000
C	-4.067234000	-8.771590000	-6.801711000
H	-3.271738000	-8.185077000	-6.323989000
H	-3.682494000	-9.782041000	-6.977451000
H	-4.292670000	-8.315768000	-7.774022000
C	-5.766500000	-7.348338000	-5.612689000
H	-4.960175000	-6.820911000	-5.089062000
H	-5.978512000	-6.794552000	-6.535687000
H	-6.658495000	-7.311894000	-4.973513000
C	-4.982567000	-9.479600000	-4.550765000
H	-4.221512000	-8.897781000	-4.015290000
H	-5.878578000	-9.534006000	-3.920009000
H	-4.584442000	-10.491159000	-4.686867000
C	-9.949935000	-9.087591000	-8.123915000

C	-10.751435000	-8.717255000	-6.847939000
H	-10.192977000	-8.015772000	-6.215095000
H	-11.701065000	-8.238159000	-7.119377000
H	-10.972334000	-9.616779000	-6.259489000
C	-9.629740000	-7.802255000	-8.930033000
H	-9.067994000	-7.072485000	-8.336393000
H	-9.039990000	-8.040561000	-9.823998000
H	-10.561450000	-7.320736000	-9.253166000
C	-10.830939000	-9.998671000	-9.003423000
H	-10.299996000	-10.311829000	-9.910064000
H	-11.157824000	-10.892129000	-8.459525000
H	-11.730280000	-9.451837000	-9.312119000

5•6$_N$···Li^+

O	9.126868000	10.373127000	7.711881000
O	9.655363000	11.765290000	11.047802000
O	9.121221000	11.901635000	13.762390000
O	6.950418000	11.001351000	15.383449000
O	9.179129000	7.123839000	13.110984000
N	11.842267000	4.323405000	9.883443000
N	8.085545000	4.300096000	15.384714000
N	9.968155000	9.149285000	12.390456000
H	9.710026000	10.033272000	11.936220000
C	9.739626000	3.735346000	12.810733000
H	10.535044000	3.891494000	13.541442000
C	8.518099000	3.165242000	13.226136000
C	7.522171000	2.935000000	12.267810000
H	6.575996000	2.484774000	12.564770000
C	7.745727000	3.279217000	10.925743000
H	6.971288000	3.088170000	10.186161000
C	8.940243000	3.889867000	10.540236000
H	9.111439000	4.175013000	9.504696000
C	9.952242000	4.133649000	11.486433000
C	11.218894000	4.864006000	11.096118000
H	10.966716000	5.915927000	10.904986000
H	11.923815000	4.866173000	11.952975000
C	12.395805000	2.982514000	10.107418000
H	12.810889000	2.596833000	9.169540000
H	11.601026000	2.304049000	10.437378000
H	13.197728000	2.973582000	10.874542000

C	12.856168000	5.251961000	9.348682000
H	13.364638000	4.733235000	8.524108000
H	13.634013000	5.489998000	10.103325000
C	12.241874000	6.536847000	8.832892000
C	11.243492000	6.492043000	7.842351000
H	10.908901000	5.523741000	7.474133000
C	10.690241000	7.666203000	7.334807000
H	9.916972000	7.618405000	6.569187000
C	11.124318000	8.920904000	7.799032000
C	12.107233000	8.961217000	8.795222000
H	12.440384000	9.923628000	9.177331000
C	12.657365000	7.783441000	9.313588000
H	13.406771000	7.835561000	10.101424000
C	10.500519000	10.194104000	7.271870000
H	11.117549000	11.059872000	7.563247000
H	10.428599000	10.180258000	6.178719000
C	8.977293000	10.466893000	9.132848000
H	9.632029000	9.744485000	9.641925000
H	7.939871000	10.193595000	9.349541000
C	9.289543000	11.878299000	9.655400000
H	10.122527000	12.326759000	9.090907000
H	8.415439000	12.539975000	9.553030000
C	9.739510000	13.028931000	11.729541000
H	8.777892000	13.557191000	11.642889000
H	10.522520000	13.656380000	11.270444000
C	10.081002000	12.798032000	13.192379000
H	11.093923000	12.376409000	13.302927000
H	10.059482000	13.775234000	13.706467000
C	9.218152000	11.809274000	15.188669000
H	8.928004000	12.770694000	15.646344000
H	10.257014000	11.580147000	15.488055000
C	8.317868000	10.696443000	15.695614000
H	8.609934000	9.734055000	15.244498000
H	8.455980000	10.617561000	16.788109000
C	5.989161000	10.321288000	16.215803000
H	5.016144000	10.676833000	15.856435000
H	6.109861000	10.644393000	17.263587000
C	6.073896000	8.808231000	16.135731000
C	5.816659000	8.137559000	14.928872000
H	5.517953000	8.701417000	14.049631000

C	5.959707000	6.750764000	14.846523000
H	5.745505000	6.249400000	13.902177000
C	6.361703000	5.994007000	15.965073000
C	6.568428000	6.661434000	17.181768000
H	6.840426000	6.094639000	18.070783000
C	6.433151000	8.051333000	17.260612000
H	6.612441000	8.554628000	18.209910000
C	6.656164000	4.516128000	15.806899000
H	6.444093000	3.960745000	16.736082000
H	6.022244000	4.101842000	15.015003000
C	9.019562000	4.468283000	16.525181000
H	8.909232000	5.470314000	16.951931000
H	10.050338000	4.359061000	16.171416000
H	8.837227000	3.726889000	17.321935000
C	8.277825000	2.956189000	14.707428000
H	7.402638000	2.318429000	14.882942000
H	9.139199000	2.457849000	15.166309000
C	8.982136000	8.242812000	12.554134000
C	7.633021000	8.568111000	12.004945000
C	6.909828000	7.511145000	11.436091000
H	7.380764000	6.531890000	11.380169000
C	5.621770000	7.713950000	10.917606000
C	5.079510000	9.000844000	11.021930000
H	4.077098000	9.177201000	10.647358000
C	5.776120000	10.082854000	11.596332000
C	7.071196000	9.856320000	12.077528000
H	7.631247000	10.654903000	12.550110000
C	4.864082000	6.541863000	10.264189000
C	3.474631000	6.956354000	9.736685000
H	2.823157000	7.318259000	10.542363000
H	2.983980000	6.089706000	9.277435000
H	3.547467000	7.739492000	8.972086000
C	4.670007000	5.418072000	11.315816000
H	4.059257000	5.775743000	12.154757000
H	5.630658000	5.067960000	11.709376000
H	4.157853000	4.556894000	10.866859000
C	5.699960000	6.002965000	9.073562000
H	5.840660000	6.781680000	8.313687000
H	5.186196000	5.154690000	8.602576000
H	6.690489000	5.664502000	9.395501000

C	5.080953000	11.454689000	11.682227000
H	3.096243000	10.591170000	12.123223000
C	4.701421000	11.921986000	10.251773000
H	5.597780000	12.010473000	9.623755000
H	4.214823000	12.904904000	10.292557000
H	4.012299000	11.227031000	9.758219000
C	3.799282000	11.318698000	12.545741000
H	4.054150000	10.995641000	13.563257000
H	3.284242000	12.285201000	12.614656000
C	5.985892000	12.527101000	12.318652000
H	6.279097000	12.267237000	13.341278000
H	5.454534000	13.486526000	12.343999000
H	6.898025000	12.664168000	11.728512000
C	11.355241000	9.023252000	12.683250000
C	11.895703000	7.968305000	13.433456000
H	11.233258000	7.221954000	13.838201000
C	13.280442000	7.886079000	13.634439000
C	14.101069000	8.879567000	13.078970000
H	15.175908000	8.817842000	13.226786000
C	13.580722000	9.956494000	12.340916000
C	12.197472000	10.012239000	12.147661000
H	11.750423000	10.801614000	11.556351000
C	13.910577000	6.732065000	14.440044000
C	14.846858000	5.915519000	13.509733000
H	15.652815000	6.538537000	13.105472000
H	14.288410000	5.497205000	12.662844000
H	15.306462000	5.085592000	14.062356000
C	12.847373000	5.774498000	15.023685000
H	12.158284000	6.300028000	15.697733000
H	13.342079000	4.981811000	15.597929000
H	12.258276000	5.295502000	14.231999000
C	14.734445000	7.312288000	15.619595000
H	14.094535000	7.900157000	16.290084000
H	15.541612000	7.963987000	15.267278000
H	15.188836000	6.500081000	16.201693000
C	14.532156000	11.024386000	11.765262000
C	15.508042000	10.353365000	10.762726000
H	16.096999000	9.563855000	11.242924000
H	16.206805000	11.095864000	10.356765000
H	14.962191000	9.904208000	9.923973000

C	15.338502000	11.666418000	12.924655000
H	15.948563000	10.926792000	13.455779000
H	14.664355000	12.133322000	13.654267000
H	16.013429000	12.440334000	12.536870000
C	13.773479000	12.150024000	11.028766000
H	13.086960000	12.680027000	11.701101000
H	13.196587000	11.766755000	10.177904000
H	14.489403000	12.882196000	10.637068000
Li	8.414144000	5.779317000	13.974118000

5•6$_N$····K^+

O	12.294082000	9.493721000	7.751290000
O	10.788564000	12.175686000	9.749228000
O	8.704853000	11.946355000	11.582308000
O	8.112878000	11.571779000	15.154737000
O	10.007165000	7.443336000	12.670908000
N	8.769077000	3.795413000	10.747405000
N	8.451891000	4.099368000	16.157084000
N	10.456177000	9.495383000	11.741603000
H	9.990670000	10.312224000	11.334846000
C	7.857330000	2.462803000	13.459001000
H	8.941186000	2.350925000	13.536778000
C	7.108119000	2.614056000	14.635020000
C	5.709915000	2.667518000	14.528080000
H	5.105400000	2.764982000	15.428695000
C	5.091518000	2.587378000	13.275422000
H	4.006264000	2.618586000	13.204898000
C	5.862651000	2.494531000	12.110927000
H	5.375985000	2.464327000	11.137120000
C	7.262230000	2.441571000	12.188253000
C	8.145488000	2.453902000	10.957233000
H	8.963879000	1.733136000	11.082835000
H	7.569732000	2.144772000	10.065034000
C	7.754391000	4.780810000	10.317706000
H	8.210571000	5.768587000	10.215624000
H	6.955794000	4.842433000	11.063332000
H	7.296424000	4.508117000	9.347805000
C	9.868208000	3.685756000	9.741158000
H	9.461950000	3.330916000	8.774677000
H	10.558187000	2.913424000	10.106570000

C	10.618751000	4.982052000	9.514407000
C	10.360165000	5.766791000	8.381856000
H	9.615040000	5.432834000	7.662003000
C	11.053831000	6.958695000	8.152075000
H	10.861749000	7.541375000	7.254342000
C	12.021544000	7.407776000	9.059292000
C	12.286344000	6.627352000	10.195865000
H	13.040700000	6.955169000	10.908274000
C	11.602207000	5.429586000	10.413375000
H	11.854972000	4.820926000	11.283890000
C	12.829504000	8.674442000	8.800212000
H	12.935897000	9.246737000	9.737257000
H	13.840510000	8.402434000	8.467761000
C	11.174549000	10.296032000	8.162467000
H	10.605924000	9.787876000	8.953416000
H	10.529739000	10.392274000	7.280543000
C	11.624179000	11.676006000	8.691924000
H	12.618088000	11.576878000	9.142261000
H	11.689651000	12.412185000	7.874407000
C	9.451581000	12.543154000	9.383080000
H	8.903808000	11.683854000	8.969158000
H	9.459050000	13.348275000	8.627691000
C	8.757803000	13.031624000	10.641960000
H	9.320949000	13.874067000	11.077000000
H	7.740132000	13.380498000	10.396032000
C	8.262643000	12.345489000	12.894643000
H	7.179487000	12.549487000	12.889633000
H	8.787861000	13.262120000	13.207486000
C	8.594234000	11.208280000	13.855090000
H	8.123349000	10.275531000	13.513871000
H	9.684326000	11.058451000	13.881440000
C	8.761548000	10.834371000	16.210773000
H	8.411123000	11.307678000	17.135656000
H	9.853629000	10.970365000	16.138991000
C	8.434227000	9.352041000	16.220188000
C	7.140062000	8.908454000	16.548135000
H	6.364930000	9.639736000	16.772624000
C	6.841543000	7.548692000	16.597144000
H	5.832810000	7.230796000	16.859831000
C	7.822515000	6.574435000	16.323723000

C	9.107058000	7.021573000	15.985655000
H	9.904357000	6.308090000	15.788505000
C	9.406454000	8.390441000	15.925619000
H	10.412658000	8.703634000	15.651848000
C	7.398768000	5.111492000	16.390326000
H	6.905454000	4.933148000	17.368051000
H	6.621358000	4.950521000	15.631906000
C	9.371389000	4.020753000	17.316012000
H	9.795627000	5.004083000	17.531998000
H	10.188873000	3.326509000	17.089388000
H	8.854152000	3.665889000	18.226994000
C	7.823121000	2.752512000	15.962550000
H	7.128150000	2.531016000	16.793931000
H	8.634898000	2.016591000	16.017533000
C	9.666013000	8.407731000	11.954141000
C	8.298481000	8.398883000	11.333450000
C	8.068592000	8.604761000	9.968226000
H	8.913169000	8.815548000	9.319679000
C	6.774934000	8.469166000	9.430746000
C	5.730201000	8.133863000	10.307421000
H	4.730031000	8.025213000	9.909002000
C	5.930298000	7.927837000	11.683617000
C	7.231243000	8.060732000	12.180450000
H	7.436493000	7.914800000	13.238132000
C	6.539544000	8.651094000	7.917024000
C	7.092291000	10.026485000	7.465545000
H	6.608127000	10.842695000	8.016444000
H	6.907216000	10.176715000	6.394427000
H	8.173204000	10.096394000	7.628262000
C	5.044104000	8.586673000	7.538254000
H	4.460694000	9.363528000	8.048355000
H	4.603963000	7.610228000	7.775195000
H	4.935801000	8.742596000	6.458326000
C	7.286070000	7.527384000	7.152223000
H	8.362205000	7.560644000	7.347500000
H	7.131152000	7.634945000	6.070805000
H	6.914904000	6.539613000	7.453822000
C	4.781753000	7.575300000	12.647902000
H	5.335963000	5.449379000	12.779984000
C	3.451815000	7.306018000	11.913711000

H	3.100556000	8.189591000	11.367178000
H	2.676756000	7.042667000	12.643773000
H	3.540257000	6.474208000	11.202733000
C	5.163250000	6.302389000	13.446990000
H	6.066921000	6.465731000	14.042449000
H	4.356672000	6.029394000	14.139404000
C	4.577845000	8.756513000	13.634266000
H	5.493868000	8.974758000	14.193369000
H	3.787223000	8.514005000	14.356684000
H	4.281789000	9.665602000	13.096147000
C	11.684953000	9.789286000	12.403711000
C	12.446131000	8.822530000	13.075509000
H	12.112769000	7.796829000	13.069630000
C	13.604967000	9.193411000	13.773218000
C	13.995137000	10.541846000	13.769212000
H	14.885768000	10.836423000	14.309379000
C	13.257877000	11.522894000	13.083047000
C	12.101081000	11.128102000	12.401443000
H	11.506400000	11.852023000	11.856345000
C	14.390683000	8.097906000	14.524517000
C	14.900532000	7.047273000	13.503540000
H	15.559474000	7.514291000	12.760941000
H	14.067504000	6.575690000	12.967486000
H	15.465729000	6.256516000	14.014251000
C	13.448450000	7.402325000	15.543964000
H	13.060624000	8.123982000	16.274016000
H	13.988830000	6.618897000	16.091588000
H	12.589590000	6.937099000	15.043339000
C	15.603283000	8.661572000	15.294789000
H	15.296481000	9.394915000	16.051060000
H	16.324159000	9.142271000	14.622245000
H	16.124028000	7.846161000	15.811603000
C	13.648198000	13.013879000	13.077615000
C	14.967181000	13.281186000	13.832495000
H	14.896905000	12.997620000	14.890179000
H	15.203666000	14.351257000	13.792252000
H	15.808552000	12.737590000	13.384291000
C	12.515321000	13.826206000	13.761921000
H	12.381273000	13.505964000	14.803157000
H	11.558582000	13.693747000	13.242584000

H	12.757685000	14.896824000	13.759988000
C	13.811026000	13.498635000	11.611938000
H	12.878654000	13.393327000	11.045853000
H	14.592240000	12.924182000	11.097404000
H	14.100008000	14.557496000	11.594552000
K	9.357567000	5.093998000	13.301219000

5•6$_{O}$····Li^{+}

Li	-8.389716000	-12.246937000	-4.256498000
O	-10.412345000	-12.376559000	-4.361668000
O	-8.914517000	-10.665146000	-3.106932000
O	-6.444094000	-11.662223000	-3.507558000
O	-3.647421000	-12.545998000	-4.550975000
O	-7.948709000	-13.760339000	-5.244980000
N	-10.981720000	-14.106003000	-10.935967000
N	-5.280643000	-13.015521000	-9.466371000
N	-7.201245000	-13.040983000	-7.282487000
H	-6.586643000	-13.236812000	-8.106763000
C	-7.477514000	-14.010937000	-6.385363000
C	-8.238427000	-12.916942000	-10.844238000
H	-8.382419000	-13.707944000	-10.113510000
C	-6.955210000	-12.610849000	-11.315567000
C	-6.814091000	-11.596319000	-12.277142000
H	-5.823600000	-11.353069000	-12.659220000
C	-7.933305000	-10.910934000	-12.755563000
H	-7.814231000	-10.131014000	-13.505220000
C	-9.209741000	-11.248400000	-12.293281000
H	-10.085900000	-10.736127000	-12.688591000
C	-9.373672000	-12.257183000	-11.335021000
C	-10.748633000	-12.654309000	-10.840122000
H	-11.522033000	-12.079014000	-11.390492000
H	-10.848442000	-12.380056000	-9.781699000
C	-11.045191000	-14.559118000	-12.330670000
H	-10.117720000	-14.290278000	-12.847095000
H	-11.152166000	-15.649778000	-12.354600000
H	-11.893254000	-14.111310000	-12.887892000
C	-12.178733000	-14.512377000	-10.176708000
H	-13.087378000	-13.970217000	-10.511308000
H	-12.349035000	-15.577158000	-10.388632000
C	-11.982571000	-14.315060000	-8.687962000

C	-12.823137000	-13.479651000	-7.939873000
H	-13.654095000	-12.976409000	-8.432499000
C	-12.596664000	-13.274154000	-6.575211000
H	-13.258585000	-12.615269000	-6.014381000
C	-11.519141000	-13.898321000	-5.931368000
C	-10.699763000	-14.764955000	-6.673234000
H	-9.880786000	-15.278522000	-6.177535000
C	-10.923552000	-14.964641000	-8.033312000
H	-10.266309000	-15.620201000	-8.600251000
C	-11.198037000	-13.615669000	-4.489113000
H	-10.576811000	-14.412186000	-4.065401000
H	-12.105672000	-13.511393000	-3.880991000
C	-11.104910000	-11.273605000	-3.748597000
H	-11.986716000	-10.981592000	-4.339029000
H	-11.430801000	-11.555296000	-2.734895000
C	-10.116414000	-10.120157000	-3.691395000
H	-10.513817000	-9.299189000	-3.075147000
H	-9.893423000	-9.740635000	-4.699299000
C	-7.793586000	-9.760902000	-3.019441000
H	-7.591687000	-9.331498000	-4.009530000
H	-8.002177000	-8.950287000	-2.304364000
C	-6.624199000	-10.602110000	-2.545890000
H	-6.843751000	-11.024485000	-1.552174000
H	-5.712481000	-9.988605000	-2.477140000
C	-5.525107000	-12.696173000	-3.056650000
H	-4.784266000	-12.255693000	-2.375569000
H	-6.101182000	-13.456844000	-2.506690000
C	-4.793675000	-13.345283000	-4.218420000
H	-5.458047000	-13.464030000	-5.080237000
H	-4.477195000	-14.347811000	-3.893143000
C	-1.547029000	-14.902718000	-4.170269000
H	-1.370257000	-15.933332000	-3.865529000
H	-1.004139000	-14.126831000	-3.634030000
C	-2.401462000	-14.603316000	-5.171571000
C	-2.702587000	-13.149712000	-5.519462000
H	-1.806643000	-12.539565000	-5.360415000
C	-3.186530000	-12.968665000	-6.937427000
H	-3.057140000	-11.977712000	-7.362989000
C	-3.713058000	-13.984741000	-7.662023000
C	-3.766481000	-15.326165000	-7.073423000

H	-4.294511000	-16.106080000	-7.618310000
C	-3.115851000	-15.623205000	-5.921628000
H	-3.092635000	-16.645024000	-5.547316000
C	-4.103402000	-13.869984000	-9.132392000
H	-4.336655000	-14.876970000	-9.493929000
H	-3.224963000	-13.523424000	-9.710047000
C	-4.927945000	-11.579585000	-9.438262000
H	-5.808810000	-10.980948000	-9.682965000
H	-4.594532000	-11.298870000	-8.441103000
H	-4.125616000	-11.347947000	-10.164353000
C	-5.745355000	-13.385254000	-10.838299000
H	-4.918512000	-13.243880000	-11.559563000
H	-5.975019000	-14.456266000	-10.817428000
C	-7.182386000	-15.416487000	-6.787942000
C	-7.304365000	-15.846172000	-8.118134000
H	-7.640915000	-15.139613000	-8.867461000
C	-7.054279000	-17.182809000	-8.462277000
C	-6.630172000	-18.047854000	-7.439492000
H	-6.407308000	-19.079460000	-7.695766000
C	-6.497321000	-17.648223000	-6.098236000
C	-6.804582000	-16.320564000	-5.784967000
H	-6.741687000	-15.955420000	-4.766729000
C	-7.198251000	-17.709212000	-9.904343000
C	-8.032427000	-16.761771000	-10.794371000
H	-8.149604000	-17.203955000	-11.790955000
H	-9.034118000	-16.589079000	-10.383001000
H	-7.558341000	-15.785118000	-10.930121000
C	-5.776709000	-17.852371000	-10.510786000
H	-5.168522000	-18.558893000	-9.932853000
H	-5.833411000	-18.215893000	-11.544957000
H	-5.255254000	-16.886429000	-10.514722000
C	-7.899280000	-19.092618000	-9.910327000
H	-7.320195000	-19.856540000	-9.380054000
H	-8.889802000	-19.030857000	-9.442074000
H	-8.029149000	-19.439004000	-10.943193000
C	-6.062653000	-18.669840000	-5.030503000
C	-4.764708000	-19.389503000	-5.481355000
H	-4.910874000	-19.957558000	-6.406805000
H	-3.953409000	-18.671428000	-5.652141000
H	-4.438188000	-20.095502000	-4.707814000

C	-7.193208000	-19.719798000	-4.857466000
H	-8.127444000	-19.238877000	-4.541258000
H	-7.388223000	-20.255220000	-5.794272000
H	-6.911775000	-20.458603000	-4.095937000
C	-5.795794000	-18.002435000	-3.663472000
H	-6.699946000	-17.535928000	-3.252817000
H	-5.461755000	-18.759804000	-2.944306000
H	-5.013910000	-17.235198000	-3.733994000
C	-7.466378000	-11.643535000	-7.054033000
C	-6.436486000	-10.820295000	-6.585596000
H	-5.501727000	-11.282625000	-6.292213000
C	-6.610764000	-9.430577000	-6.529788000
C	-7.863773000	-8.915625000	-6.908462000
H	-8.005961000	-7.840349000	-6.891253000
C	-8.929254000	-9.725933000	-7.335799000
C	-8.708645000	-11.113455000	-7.409580000
H	-9.472700000	-11.795366000	-7.769224000
C	-5.438562000	-8.482183000	-6.198003000
C	-4.917587000	-7.905298000	-7.544088000
H	-4.052330000	-7.252659000	-7.369156000
H	-4.608844000	-8.710908000	-8.221266000
H	-5.694273000	-7.318323000	-8.049756000
C	-5.882820000	-7.302641000	-5.297284000
H	-5.035850000	-6.625279000	-5.132043000
H	-6.688204000	-6.713562000	-5.748382000
H	-6.223924000	-7.651117000	-4.315036000
C	-4.280050000	-9.218481000	-5.489862000
H	-3.491481000	-8.500958000	-5.233357000
H	-4.611168000	-9.711126000	-4.569490000
H	-3.829429000	-9.990156000	-6.122465000
C	-10.249041000	-9.104609000	-7.836107000
C	-10.548696000	-7.750838000	-7.148801000
H	-9.815559000	-6.979504000	-7.409820000
H	-11.530492000	-7.383562000	-7.471147000
H	-10.567060000	-7.848434000	-6.055255000
C	-10.109315000	-8.873117000	-9.365336000
H	-9.281771000	-8.185959000	-9.581239000
H	-9.909265000	-9.813937000	-9.889858000
H	-11.032484000	-8.437950000	-9.771392000
C	-11.447081000	-10.045908000	-7.580782000

H	-11.354481000	-10.994983000	-8.114980000
H	-11.556147000	-10.276245000	-6.514658000
H	-12.374098000	-9.569091000	-7.922486000

5•6$_O$····K$^+$

K	-10.141940000	-15.004583000	-6.622021000
O	-11.755183000	-15.231241000	-4.411133000
O	-9.487439000	-13.520434000	-4.228928000
O	-6.610957000	-12.766591000	-3.867462000
O	-4.156213000	-13.038092000	-5.357142000
O	-8.531530000	-13.258865000	-7.800464000
N	-11.469053000	-13.482325000	-10.861209000
N	-5.149087000	-13.089396000	-10.020748000
N	-6.528987000	-12.505276000	-6.938554000
H	-5.663972000	-12.823854000	-6.486526000
C	-7.390239000	-13.471287000	-7.319211000
C	-8.156572000	-12.908549000	-10.983028000
H	-8.169606000	-13.595648000	-10.142191000
C	-6.970842000	-12.713532000	-11.712822000
C	-6.974334000	-11.798071000	-12.775279000
H	-6.058531000	-11.634029000	-13.341407000
C	-8.131487000	-11.084551000	-13.100078000
H	-8.120788000	-10.372166000	-13.922763000
C	-9.294172000	-11.275101000	-12.353922000
H	-10.189337000	-10.701788000	-12.589124000
C	-9.322526000	-12.189670000	-11.286959000
C	-10.596102000	-12.335010000	-10.476080000
H	-11.163691000	-11.387423000	-10.579558000
H	-10.343617000	-12.449025000	-9.415369000
C	-11.558673000	-13.681511000	-12.319347000
H	-10.562471000	-13.859267000	-12.731587000
H	-12.178315000	-14.563785000	-12.516328000
H	-12.004372000	-12.818598000	-12.853880000
C	-12.829538000	-13.299402000	-10.303857000
H	-13.201969000	-12.273591000	-10.508335000
H	-13.493358000	-13.991555000	-10.834198000
C	-12.939442000	-13.584027000	-8.817780000
C	-12.725490000	-12.585275000	-7.852275000
H	-12.485688000	-11.572950000	-8.171563000
C	-12.811082000	-12.876569000	-6.484924000

H	-12.610971000	-12.094419000	-5.753132000
C	-13.135195000	-14.172441000	-6.047314000
C	-13.408179000	-15.160884000	-7.011987000
H	-13.692182000	-16.162108000	-6.688662000
C	-13.298277000	-14.871449000	-8.375935000
H	-13.502097000	-15.649049000	-9.111073000
C	-13.040401000	-14.547850000	-4.589285000
H	-13.839209000	-15.239564000	-4.285669000
H	-13.086749000	-13.655030000	-3.953053000
C	-11.074311000	-14.992968000	-3.163125000
H	-11.765244000	-15.087004000	-2.310628000
H	-10.327633000	-15.793274000	-3.087702000
C	-10.384557000	-13.633120000	-3.111554000
H	-9.843402000	-13.549543000	-2.154243000
H	-11.122111000	-12.813191000	-3.152910000
C	-8.914883000	-12.199945000	-4.385821000
H	-8.641195000	-12.117860000	-5.439111000
H	-9.684756000	-11.439501000	-4.173672000
C	-7.701017000	-11.908307000	-3.510074000
H	-7.937685000	-12.020194000	-2.436721000
H	-7.422199000	-10.854481000	-3.685891000
C	-5.344750000	-12.298212000	-3.379609000
H	-5.204556000	-11.247923000	-3.676375000
H	-5.313956000	-12.350842000	-2.275932000
C	-4.214476000	-13.158244000	-3.925393000
H	-4.349634000	-14.208178000	-3.631067000
H	-3.270445000	-12.803097000	-3.477565000
C	-2.100669000	-15.550764000	-4.727322000
H	-2.040977000	-16.619970000	-4.530774000
H	-1.557399000	-14.894721000	-4.049733000
C	-2.795277000	-15.067070000	-5.778932000
C	-2.919915000	-13.567952000	-6.005697000
H	-2.076452000	-13.044400000	-5.531800000
C	-3.056908000	-13.194313000	-7.458366000
H	-2.804281000	-12.173744000	-7.737833000
C	-3.550496000	-14.072976000	-8.362507000
C	-3.815565000	-15.454259000	-7.960284000
H	-4.261542000	-16.117747000	-8.694831000
C	-3.444029000	-15.934234000	-6.750827000
H	-3.564252000	-16.988101000	-6.509737000

C	-3.819027000	-13.717729000	-9.816806000
H	-3.789903000	-14.642451000	-10.405768000
H	-3.013859000	-13.066475000	-10.207775000
C	-5.059393000	-11.635041000	-9.824322000
H	-6.052954000	-11.182386000	-9.862664000
H	-4.637697000	-11.426174000	-8.839614000
H	-4.417163000	-11.150100000	-10.588133000
C	-5.682460000	-13.435365000	-11.364200000
H	-4.937525000	-13.204418000	-12.152435000
H	-5.839573000	-14.523165000	-11.378566000
C	-6.983613000	-14.903146000	-7.082722000
C	-7.016771000	-15.788160000	-8.172747000
H	-7.172038000	-15.377544000	-9.163485000
C	-6.841320000	-17.164516000	-7.978509000
C	-6.687971000	-17.624261000	-6.656819000
H	-6.587135000	-18.693796000	-6.490205000
C	-6.674387000	-16.764629000	-5.545774000
C	-6.784073000	-15.382472000	-5.779214000
H	-6.780868000	-14.671466000	-4.961192000
C	-6.835348000	-18.169145000	-9.147655000
C	-8.032437000	-19.143322000	-8.984302000
H	-8.041553000	-19.876838000	-9.800479000
H	-7.978371000	-19.692019000	-8.036913000
H	-8.984356000	-18.595871000	-9.007892000
C	-6.958978000	-17.469089000	-10.518319000
H	-6.132211000	-16.768706000	-10.691911000
H	-6.932931000	-18.217884000	-11.318741000
H	-7.900974000	-16.914504000	-10.613157000
C	-5.511304000	-18.978805000	-9.131575000
H	-4.642326000	-18.317684000	-9.240449000
H	-5.389631000	-19.545116000	-8.201044000
H	-5.498216000	-19.694941000	-9.962835000
C	-6.583023000	-17.350576000	-4.121932000
C	-5.202079000	-18.029129000	-3.930163000
H	-5.035733000	-18.820982000	-4.670344000
H	-4.395454000	-17.293463000	-4.028830000
H	-5.134045000	-18.480516000	-2.931870000
C	-7.707145000	-18.402811000	-3.927162000
H	-8.696706000	-17.949196000	-4.077363000
H	-7.611340000	-19.240881000	-4.626158000

H	-7.671273000	-18.811308000	-2.909208000
C	-6.754038000	-16.266770000	-3.035461000
H	-7.708809000	-15.736227000	-3.137539000
H	-6.723412000	-16.733801000	-2.043509000
H	-5.958095000	-15.516453000	-3.078618000
C	-6.695844000	-11.097799000	-6.989931000
C	-5.655976000	-10.317446000	-6.459038000
H	-4.789010000	-10.826606000	-6.049931000
C	-5.716709000	-8.919786000	-6.492139000
C	-6.860091000	-8.317515000	-7.045114000
H	-6.925414000	-7.237490000	-7.068278000
C	-7.909414000	-9.076882000	-7.585913000
C	-7.813024000	-10.476272000	-7.565754000
H	-8.579700000	-11.091955000	-8.012969000
C	-4.523677000	-8.101101000	-5.959104000
C	-4.758370000	-6.578755000	-6.060995000
H	-3.882538000	-6.046290000	-5.670749000
H	-4.905365000	-6.258897000	-7.099970000
H	-5.630277000	-6.260266000	-5.475833000
C	-4.269751000	-8.455473000	-4.471067000
H	-3.437397000	-7.860711000	-4.073279000
H	-5.159762000	-8.249640000	-3.862298000
H	-4.010503000	-9.513970000	-4.350046000
C	-3.262311000	-8.457376000	-6.791128000
H	-2.398316000	-7.876645000	-6.442880000
H	-3.010654000	-9.521432000	-6.704441000
H	-3.421342000	-8.233038000	-7.853228000
C	-9.134551000	-8.428072000	-8.260576000
C	-10.439240000	-9.081126000	-7.732913000
H	-10.534969000	-8.937143000	-6.648570000
H	-11.311540000	-8.623576000	-8.217817000
H	-10.467147000	-10.157593000	-7.936088000
C	-9.219662000	-6.908790000	-8.000380000
H	-9.268991000	-6.681472000	-6.927627000
H	-8.362845000	-6.373961000	-8.427469000
H	-10.124261000	-6.505162000	-8.471804000
C	-9.024289000	-8.664056000	-9.789632000
H	-8.121789000	-8.185321000	-10.190177000
H	-8.968748000	-9.730222000	-10.029871000
H	-9.895681000	-8.240162000	-10.306914000

5•6$_N$(a)···Na^+

O	-0.052934000	1.875998000	-3.936277000
O	0.183464000	4.833622000	-2.571631000
O	0.454534000	5.422675000	0.531620000
O	-1.382708000	4.515253000	3.489763000
N	3.042553000	-4.462274000	-3.101042000
N	-0.278028000	-2.803158000	2.958945000
C	1.219145000	-4.251807000	0.262428000
C	-0.082979000	-4.092094000	0.772062000
C	-1.150623000	-3.999502000	-0.136946000
C	-0.918549000	-4.068476000	-1.513290000
C	0.381481000	-4.228151000	-2.007814000
C	1.469142000	-4.318414000	-1.124538000
C	2.900507000	-4.513706000	-1.638756000
C	3.422540000	-5.752975000	-3.689390000
C	3.857667000	-3.336068000	-3.583988000
C	3.152548000	-1.994336000	-3.451381000
C	1.868056000	-1.807820000	-3.997091000
C	1.209460000	-0.576572000	-3.901120000
C	1.822106000	0.516759000	-3.260324000
C	3.111655000	0.342247000	-2.724611000
C	3.764475000	-0.897504000	-2.816310000
C	1.112675000	1.846087000	-3.109570000
C	-1.010766000	2.884498000	-3.570210000
C	-0.505191000	4.322560000	-3.732811000
C	-0.677219000	5.557221000	-1.674405000
C	0.173026000	6.254908000	-0.612685000
C	-0.440327000	5.661506000	1.628852000
C	-0.175524000	4.621782000	2.717595000
C	-1.292483000	3.577923000	4.594305000
C	-1.310418000	2.134535000	4.136558000
C	-2.519559000	1.518100000	3.763730000
C	-2.538225000	0.209757000	3.273148000
C	-1.350570000	-0.530974000	3.145304000
C	-0.151735000	0.077459000	3.538645000
C	-0.129254000	1.389346000	4.019198000
C	-1.398219000	-1.932678000	2.559213000
C	-0.277882000	-3.044697000	4.414660000
C	-0.335553000	-4.114529000	2.268885000
H	2.050148000	-4.358179000	0.961094000

H	-2.167346000	-3.892799000	0.235631000
H	-1.757769000	-4.017289000	-2.204556000
H	0.574384000	-4.302977000	-3.073574000
H	3.555872000	-3.760248000	-1.172019000
H	3.271016000	-5.480014000	-1.260186000
H	3.383889000	-5.683629000	-4.782621000
H	2.706574000	-6.520982000	-3.374136000
H	4.438777000	-6.085757000	-3.398197000
H	4.063442000	-3.519261000	-4.647251000
H	4.842410000	-3.284609000	-3.077112000
H	1.383801000	-2.641400000	-4.500393000
H	0.217553000	-0.444977000	-4.323883000
H	3.599255000	1.167165000	-2.210303000
H	4.761233000	-1.009688000	-2.392787000
H	0.828625000	1.998222000	-2.058834000
H	1.802958000	2.666690000	-3.366486000
H	-1.351772000	2.721447000	-2.536587000
H	-1.862326000	2.713744000	-4.238205000
H	0.208224000	4.367486000	-4.566699000
H	-1.354787000	4.981443000	-3.976633000
H	-1.406238000	4.880183000	-1.198683000
H	-1.239309000	6.315398000	-2.249105000
H	1.137296000	6.527206000	-1.058711000
H	-0.326915000	7.179194000	-0.286232000
H	-1.487558000	5.567709000	1.302859000
H	-0.300723000	6.684981000	2.013703000
H	0.085161000	3.660627000	2.252795000
H	0.665114000	4.921154000	3.365924000
H	-2.167130000	3.801239000	5.215450000
H	-0.383323000	3.786302000	5.180851000
H	-3.453503000	2.071169000	3.857413000
H	-3.487491000	-0.244873000	2.991713000
H	0.770775000	-0.487468000	3.473336000
H	0.820738000	1.836925000	4.309647000
H	-2.374160000	-2.394076000	2.823157000
H	-1.373863000	-1.837844000	1.466091000
H	-0.237396000	-2.095469000	4.954799000
H	0.603529000	-3.637161000	4.685749000
H	-1.183279000	-3.590596000	4.748073000
H	-1.314365000	-4.605227000	2.453139000

H	0.426686000	-4.747420000	2.739355000
O	-0.377934000	-0.517951000	-0.292828000
N	0.035865000	1.639857000	0.305568000
C	-0.825933000	0.666563000	-0.169279000
C	-2.164900000	1.062199000	-0.618279000
C	-2.835989000	0.281194000	-1.637805000
C	-3.819269000	0.878928000	-2.479226000
C	-4.136780000	2.236342000	-2.191815000
C	-3.905227000	2.798177000	-0.879572000
C	-2.803401000	2.278600000	-0.197046000
C	-4.467144000	0.048118000	-3.638289000
C	-3.422435000	-0.305244000	-4.737048000
C	-5.641986000	0.728599000	-4.399179000
C	-5.145071000	-1.227984000	-3.036739000
C	-4.895320000	3.849336000	-0.272732000
C	-4.486670000	5.315640000	-0.575477000
C	-6.348964000	3.597955000	-0.783724000
C	-5.032874000	3.693474000	1.280270000
C	1.435601000	1.526660000	0.504010000
C	2.051681000	0.390056000	1.064717000
C	3.448560000	0.341442000	1.225574000
C	4.204276000	1.443578000	0.800331000
C	3.613404000	2.605112000	0.274150000
C	2.220755000	2.633135000	0.136877000
C	4.165625000	-0.883827000	1.835934000
C	4.908950000	-1.641799000	0.704921000
C	3.190660000	-1.860666000	2.523246000
C	5.198236000	-0.426091000	2.899580000
C	4.503622000	3.793304000	-0.140507000
C	5.481588000	3.337901000	-1.255597000
C	5.315783000	4.273227000	1.091595000
C	3.677818000	4.982882000	-0.673916000
Na	1.489212000	-1.446659000	-0.858891000
N	-2.443048000	-1.028103000	-1.808790000
N	-4.598401000	3.098316000	-3.159868000
N	-2.227714000	2.967944000	0.881072000
H	-0.345806000	2.588419000	0.317023000
H	-3.061438000	0.612114000	-5.218049000
H	-3.882593000	-0.935108000	-5.509981000
H	-2.533866000	-0.829300000	-4.369892000

H	-5.308769000	1.478764000	-5.129885000
H	-6.384906000	1.165303000	-3.722684000
H	-6.154199000	-0.038709000	-4.991736000
H	-4.507674000	-1.808562000	-2.370196000
H	-5.483887000	-1.882267000	-3.850701000
H	-6.026157000	-0.928549000	-2.455456000
H	-3.485624000	5.537202000	-0.185483000
H	-5.191694000	6.017907000	-0.110976000
H	-4.465884000	5.536275000	-1.650844000
H	-6.494001000	3.771866000	-1.851313000
H	-6.652461000	2.565420000	-0.571450000
H	-7.034742000	4.269329000	-0.254861000
H	-5.026578000	2.638248000	1.575423000
H	-5.987884000	4.129007000	1.594842000
H	-4.265861000	4.217855000	1.859368000
H	1.424566000	-0.411758000	1.431962000
H	5.284656000	1.409355000	0.902946000
H	1.719116000	3.512042000	-0.251383000
H	5.617957000	-0.988890000	0.182992000
H	4.203229000	-2.029111000	-0.043087000
H	5.462426000	-2.497560000	1.112597000
H	2.686437000	-1.396711000	3.378070000
H	3.744777000	-2.730371000	2.897479000
H	2.408678000	-2.235673000	1.854823000
H	4.711875000	0.169056000	3.682133000
H	6.001018000	0.178625000	2.464579000
H	5.662619000	-1.300512000	3.372791000
H	6.119701000	2.507319000	-0.930029000
H	6.136002000	4.169796000	-1.545684000
H	4.932915000	3.015580000	-2.150180000
H	5.954237000	3.478016000	1.493238000
H	4.644618000	4.606599000	1.893176000
H	5.962196000	5.115439000	0.813034000
H	2.968335000	5.348542000	0.077039000
H	3.111741000	4.716793000	-1.575235000
H	4.352566000	5.806895000	-0.935966000
H	-2.582993000	-1.460831000	-2.708024000
H	-1.593884000	-1.283811000	-1.305822000
H	-5.019151000	2.758063000	-4.009932000
H	-4.857195000	4.033827000	-2.886869000

H	-2.718832000	3.801476000	1.167740000
H	-2.024712000	2.399598000	1.702618000

5•6$_N$(b)···Na$^+$

O	3.664147000	2.089743000	-3.733772000
O	1.174329000	4.679451000	-2.939340000
O	-0.896888000	4.625681000	-0.953215000
O	-1.218938000	3.939960000	1.869026000
N	-0.071646000	-4.039139000	-3.354138000
N	0.382362000	-2.472472000	3.302569000
C	-0.497956000	-3.540057000	0.402357000
C	-1.188152000	-2.711717000	1.301347000
C	-2.113946000	-1.789942000	0.780871000
C	-2.346739000	-1.704393000	-0.593078000
C	-1.626672000	-2.513302000	-1.479985000
C	-0.688833000	-3.437242000	-0.988503000
C	0.181732000	-4.277259000	-1.928656000
C	-1.106477000	-4.936798000	-3.884149000
C	1.157048000	-4.025719000	-4.177863000
C	1.974071000	-2.765543000	-3.948736000
C	1.496384000	-1.519846000	-4.402019000
C	2.212919000	-0.339490000	-4.170149000
C	3.425446000	-0.364180000	-3.460059000
C	3.914037000	-1.606257000	-3.018432000
C	3.201329000	-2.790063000	-3.262171000
C	4.225369000	0.904089000	-3.175109000
C	2.772800000	2.799226000	-2.848512000
C	2.242344000	4.007460000	-3.631816000
C	-0.107026000	4.075346000	-3.165620000
C	-1.168936000	4.793653000	-2.353704000
C	-1.885894000	5.210056000	-0.096133000
C	-1.359030000	5.269594000	1.327201000
C	-0.636231000	3.949653000	3.204152000
C	-0.393977000	2.526837000	3.638962000
C	-1.466307000	1.679586000	3.960836000
C	-1.245189000	0.338246000	4.283499000
C	0.056240000	-0.193101000	4.299422000
C	1.126675000	0.675045000	4.040402000
C	0.908339000	2.012896000	3.705754000
C	0.329277000	-1.662266000	4.560960000

C	1.149874000	-3.712706000	3.526165000
C	-0.995479000	-2.778868000	2.808264000
H	0.213134000	-4.274998000	0.777886000
H	-2.652487000	-1.132719000	1.461785000
H	-3.070533000	-0.991709000	-0.977534000
H	-1.764532000	-2.419727000	-2.553912000
H	1.237227000	-4.046764000	-1.720406000
H	0.068656000	-5.348867000	-1.670665000
H	-1.359486000	-4.645900000	-4.910074000
H	-2.011436000	-4.853167000	-3.271072000
H	-0.791779000	-5.999203000	-3.886796000
H	0.836873000	-4.059604000	-5.226727000
H	1.785049000	-4.921970000	-4.006515000
H	0.546331000	-1.477844000	-4.933058000
H	1.832780000	0.611714000	-4.531489000
H	4.862991000	-1.655422000	-2.485141000
H	3.607357000	-3.741862000	-2.920212000
H	4.358955000	1.013902000	-2.084420000
H	5.226569000	0.798064000	-3.617532000
H	3.320680000	3.126311000	-1.951095000
H	1.950903000	2.149548000	-2.517552000
H	3.035691000	4.751253000	-3.769766000
H	1.910602000	3.677542000	-4.630289000
H	-0.086420000	3.010429000	-2.899865000
H	-0.379909000	4.151096000	-4.234433000
H	-1.180281000	5.868332000	-2.601754000
H	-2.152663000	4.359936000	-2.603068000
H	-2.818483000	4.624155000	-0.146407000
H	-2.106942000	6.241818000	-0.420557000
H	-0.380296000	5.774364000	1.344076000
H	-2.066749000	5.846498000	1.946570000
H	-1.332771000	4.467847000	3.883703000
H	0.309879000	4.510196000	3.174839000
H	-2.479650000	2.073025000	3.957950000
H	-2.093483000	-0.294280000	4.542003000
H	2.146310000	0.298650000	4.073206000
H	1.755855000	2.653969000	3.474892000
H	1.310109000	-1.757692000	5.040572000
H	-0.415015000	-2.080223000	5.263508000
H	2.156496000	-3.467914000	3.879189000

H	1.249219000	-4.267263000	2.587975000
H	0.668735000	-4.377896000	4.268593000
H	-1.681816000	-2.053547000	3.251062000
H	-1.307116000	-3.771105000	3.182870000
O	0.362713000	0.717463000	-1.658468000
N	0.198570000	1.940838000	0.262738000
C	-0.339524000	1.381062000	-0.855364000
C	-1.804355000	1.520451000	-1.108035000
C	-2.235967000	1.279063000	-2.422048000
C	-3.592139000	1.307362000	-2.754422000
C	-4.507053000	1.573576000	-1.721112000
C	-4.114130000	1.806764000	-0.392555000
C	-2.742258000	1.780537000	-0.097863000
C	-4.098169000	1.028501000	-4.181769000
C	-4.915663000	2.245544000	-4.687984000
C	-5.002581000	-0.232554000	-4.159211000
C	-2.939601000	0.778556000	-5.171514000
C	-5.191918000	2.079601000	0.674773000
C	-6.199033000	0.900328000	0.707895000
C	-4.587244000	2.236697000	2.084607000
C	-5.939177000	3.391245000	0.314165000
C	1.583854000	1.816089000	0.627194000
C	2.095382000	0.554780000	0.982557000
C	3.454816000	0.405132000	1.335685000
C	4.165363000	1.629402000	1.592637000
C	3.699429000	2.925315000	1.186812000
C	2.374767000	2.989669000	0.671220000
C	4.172939000	-0.984845000	1.458530000
C	5.580486000	-0.942474000	0.782167000
C	3.468052000	-2.141535000	0.688124000
C	4.276639000	-1.462656000	2.933328000
C	4.637099000	4.170403000	1.385236000
C	4.734289000	4.520579000	2.904177000
C	4.195800000	5.509310000	0.725836000
C	6.034785000	3.917550000	0.730198000
Na	1.199429000	-1.281595000	-1.336687000
N	1.168802000	-0.527790000	0.967323000
N	1.723757000	4.166345000	0.308014000
N	5.387238000	1.521554000	2.273584000
H	-0.370182000	2.617148000	0.790101000

H	-1.480186000	1.048139000	-3.163373000
H	-5.567600000	1.588914000	-1.957634000
H	-2.401396000	1.942249000	0.917465000
H	-5.781146000	2.448647000	-4.047204000
H	-5.287181000	2.057628000	-5.703478000
H	-4.292753000	3.149004000	-4.713653000
H	-5.879855000	-0.091011000	-3.517398000
H	-4.446902000	-1.102211000	-3.784785000
H	-5.359725000	-0.462902000	-5.171206000
H	-2.272048000	1.646912000	-5.241516000
H	-3.346686000	0.589986000	-6.172000000
H	-2.339513000	-0.095330000	-4.886009000
H	-6.709045000	0.769387000	-0.253133000
H	-6.967074000	1.078495000	1.471308000
H	-5.688472000	-0.040978000	0.949287000
H	-4.079679000	1.318505000	2.405405000
H	-3.864906000	3.060538000	2.129303000
H	-5.385730000	2.445714000	2.807054000
H	-5.245912000	4.242297000	0.296190000
H	-6.717909000	3.601234000	1.058387000
H	-6.419868000	3.328029000	-0.668916000
H	6.270905000	-0.224588000	1.220241000
H	5.467694000	-0.678229000	-0.277076000
H	6.039433000	-1.937681000	0.836670000
H	2.523267000	-2.466042000	1.122774000
H	4.138570000	-3.009321000	0.685283000
H	3.319523000	-1.867304000	-0.362999000
H	3.274787000	-1.582966000	3.358288000
H	4.820632000	-0.776763000	3.593239000
H	4.790504000	-2.431633000	2.988173000
H	3.764963000	4.901522000	3.249557000
H	5.483256000	5.306432000	3.068572000
H	4.983290000	3.680446000	3.560676000
H	3.296538000	5.935881000	1.180681000
H	4.067693000	5.417704000	-0.360760000
H	5.000905000	6.237713000	0.880038000
H	5.920073000	3.906418000	-0.361219000
H	6.515756000	2.980594000	1.009912000
H	6.714106000	4.741445000	0.984357000
H	0.219555000	-0.156413000	1.020152000

H	1.253629000	-1.172991000	1.761698000
H	0.893688000	4.061540000	-0.272065000
H	2.296842000	4.920160000	-0.033619000
H	5.462821000	0.730176000	2.896997000
H	5.734580000	2.363611000	2.705870000

5•6$_N$(c)···Na^+

O	1.661685000	2.250421000	-4.691355000
O	0.799878000	4.868461000	-2.222158000
O	-0.395664000	4.272206000	0.303747000
O	0.714515000	5.296622000	3.695601000
N	1.633441000	-3.910642000	-2.111856000
N	0.697499000	-2.214401000	3.874865000
C	0.777482000	-4.253737000	1.212980000
C	-0.197004000	-3.890430000	2.161022000
C	-1.546362000	-3.955172000	1.785971000
C	-1.913411000	-4.425025000	0.520924000
C	-0.934946000	-4.822597000	-0.394455000
C	0.432257000	-4.710494000	-0.073549000
C	1.522105000	-5.013161000	-1.095587000
C	0.678681000	-4.110027000	-3.225611000
C	3.022908000	-3.714094000	-2.642336000
C	3.163195000	-2.324859000	-3.242635000
C	2.879062000	-2.066871000	-4.594262000
C	2.855760000	-0.756787000	-5.079655000
C	3.138113000	0.334128000	-4.240424000
C	3.486410000	0.074954000	-2.905975000
C	3.492180000	-1.234796000	-2.418380000
C	3.021934000	1.748832000	-4.768929000
C	1.292076000	2.787162000	-3.409671000
C	1.160086000	4.312386000	-3.500420000
C	-0.608728000	4.960312000	-1.976915000
C	-0.813823000	5.367276000	-0.530854000
C	-0.450430000	4.515470000	1.733510000
C	0.866512000	5.076303000	2.283772000
C	1.409832000	4.342860000	4.536174000
C	0.958759000	2.907343000	4.355200000
C	-0.353336000	2.512054000	4.671961000
C	-0.772357000	1.194449000	4.477792000
C	0.106256000	0.226491000	3.962949000

C	1.416313000	0.619703000	3.672650000
C	1.837772000	1.938418000	3.855198000
C	-0.362664000	-1.196273000	3.705348000
C	1.239998000	-2.201848000	5.248125000
C	0.189509000	-3.577659000	3.596210000
H	1.827021000	-4.224390000	1.500561000
H	-2.314913000	-3.678641000	2.505969000
H	-2.965755000	-4.510323000	0.257220000
H	-1.239803000	-5.244530000	-1.348816000
H	2.484183000	-5.085988000	-0.576887000
H	1.341984000	-5.979991000	-1.595888000
H	0.740983000	-3.271881000	-3.923148000
H	-0.344575000	-4.141235000	-2.846470000
H	0.884340000	-5.048731000	-3.771938000
H	3.274762000	-4.492528000	-3.383886000
H	3.712064000	-3.820869000	-1.796904000
H	2.669720000	-2.894796000	-5.269727000
H	2.613069000	-0.575889000	-6.126022000
H	3.749657000	0.892518000	-2.237591000
H	3.755266000	-1.409025000	-1.377996000
H	3.701828000	2.425063000	-4.228402000
H	3.276344000	1.784162000	-5.834205000
H	2.024357000	2.524098000	-2.637548000
H	0.339002000	2.332669000	-3.110714000
H	2.125706000	4.756998000	-3.772148000
H	0.430273000	4.591058000	-4.276672000
H	-1.111023000	3.993759000	-2.157767000
H	-1.068336000	5.712650000	-2.641043000
H	-0.223012000	6.268221000	-0.308233000
H	-1.879489000	5.583608000	-0.351683000
H	-0.662632000	3.551224000	2.202002000
H	-1.279566000	5.195003000	1.969315000
H	1.688802000	4.379383000	2.071200000
H	1.105403000	6.043824000	1.818882000
H	1.207213000	4.692889000	5.555343000
H	2.495378000	4.414283000	4.355928000
H	-1.053062000	3.248648000	5.064276000
H	-1.795418000	0.914905000	4.724241000
H	2.108583000	-0.124942000	3.305073000
H	2.862208000	2.212946000	3.605044000

H	-1.227764000	-1.412124000	4.367556000
H	-0.729565000	-1.269096000	2.671128000
H	1.622685000	-1.208915000	5.495230000
H	2.063346000	-2.921908000	5.319923000
H	0.473493000	-2.472124000	6.001806000
H	-0.681118000	-3.801708000	4.246842000
H	0.985944000	-4.272960000	3.891354000
O	-0.481193000	-0.600252000	0.129554000
N	0.271444000	1.581250000	0.005694000
C	-0.640198000	0.601577000	-0.157035000
C	-1.901977000	0.994789000	-0.941092000
C	-1.978543000	0.638223000	-2.299474000
C	-3.035247000	1.022732000	-3.147359000
C	-4.185014000	1.458167000	-2.438329000
C	-4.281177000	1.711843000	-1.048238000
C	-3.033534000	1.594167000	-0.381025000
C	-2.846048000	1.198419000	-4.695114000
C	-2.815254000	2.748424000	-4.911541000
C	-3.947111000	0.554141000	-5.582867000
C	-1.491830000	0.677285000	-5.239301000
C	-5.614539000	2.068588000	-0.292541000
C	-5.835987000	3.606235000	-0.338230000
C	-6.876606000	1.382072000	-0.900878000
C	-5.585728000	1.573791000	1.187000000
C	1.671743000	1.477842000	0.272824000
C	2.310141000	0.317025000	0.739093000
C	3.704928000	0.313423000	0.937786000
C	4.417109000	1.487054000	0.657638000
C	3.799068000	2.661965000	0.196622000
C	2.415091000	2.641025000	0.003297000
C	4.467214000	-0.899927000	1.521596000
C	5.737418000	-1.208826000	0.684323000
C	3.592111000	-2.168901000	1.565849000
C	4.924083000	-0.560984000	2.968321000
C	4.655820000	3.911581000	-0.081295000
C	5.668565000	3.584890000	-1.211084000
C	5.425989000	4.297828000	1.209717000
C	3.807434000	5.123446000	-0.518226000
Na	0.893479000	-1.931724000	-1.009442000
N	-0.988200000	-0.377306000	-2.743302000

O	-1.461156000	-1.446468000	-3.144219000
O	0.228767000	-0.168266000	-2.577399000
N	-5.425546000	1.610361000	-3.266505000
O	-5.758724000	2.752833000	-3.602551000
O	-6.017129000	0.568590000	-3.552197000
N	-2.838784000	2.116796000	0.998989000
O	-2.410933000	1.336729000	1.857472000
O	-3.079714000	3.316593000	1.172770000
H	-0.043190000	2.551956000	-0.157124000
H	-3.730716000	3.241269000	-4.578333000
H	-2.686044000	2.945968000	-5.982174000
H	-1.965178000	3.192848000	-4.380201000
H	-4.891037000	1.100536000	-5.581413000
H	-4.138723000	-0.482971000	-5.289150000
H	-3.586064000	0.552653000	-6.617502000
H	-0.621720000	1.062926000	-4.701312000
H	-1.395131000	1.043840000	-6.266510000
H	-1.445372000	-0.414239000	-5.278711000
H	-5.019716000	4.144382000	0.149439000
H	-6.771171000	3.843124000	0.184516000
H	-5.925856000	3.956787000	-1.371685000
H	-7.221246000	1.839068000	-1.828194000
H	-6.731983000	0.307841000	-1.055310000
H	-7.690819000	1.509797000	-0.181138000
H	-5.257567000	0.529539000	1.256465000
H	-6.606218000	1.622033000	1.578820000
H	-4.979755000	2.186562000	1.853085000
H	1.707174000	-0.534833000	1.022710000
H	5.490491000	1.497064000	0.819915000
H	1.890566000	3.515549000	-0.365684000
H	6.450938000	-0.378474000	0.700542000
H	5.498307000	-1.422619000	-0.363129000
H	6.247256000	-2.085929000	1.101824000
H	2.702936000	-2.049938000	2.197156000
H	4.174392000	-3.004392000	1.974486000
H	3.271130000	-2.464696000	0.556628000
H	4.072018000	-0.373323000	3.628523000
H	5.564915000	0.328155000	2.981229000
H	5.497279000	-1.399058000	3.385403000
H	6.332690000	2.756646000	-0.936621000

H	6.292650000	4.461582000	-1.426337000
H	5.145368000	3.304752000	-2.134989000
H	6.098517000	3.498511000	1.540696000
H	4.726942000	4.513251000	2.027568000
H	6.032152000	5.195170000	1.033384000
H	3.082934000	5.409330000	0.252830000
H	3.251148000	4.932689000	-1.441638000
H	4.464174000	5.983970000	-0.692337000

5•6$_N$(d)···Na$^+$

O	-0.948483000	3.264247000	-4.478256000
O	0.000493000	4.393821000	-1.101642000
O	0.248386000	4.487952000	1.800324000
O	-2.097164000	3.428200000	2.915547000
N	2.075312000	-3.239235000	-4.696982000
N	-1.067872000	-2.940424000	1.433387000
C	0.546127000	-3.818447000	-1.255971000
C	-0.682854000	-4.190902000	-0.682826000
C	-1.759593000	-4.467458000	-1.540708000
C	-1.627913000	-4.335683000	-2.923789000
C	-0.415151000	-3.912221000	-3.478011000
C	0.690463000	-3.663068000	-2.648093000
C	2.025827000	-3.197424000	-3.233538000
C	2.473390000	-4.560523000	-5.197767000
C	2.844585000	-2.141364000	-5.290080000
C	2.155052000	-0.788159000	-5.180149000
C	0.754942000	-0.662696000	-5.293662000
C	0.143231000	0.600509000	-5.284868000
C	0.913612000	1.772006000	-5.176396000
C	2.302937000	1.642142000	-5.057857000
C	2.913802000	0.384004000	-5.054459000
C	0.269664000	3.149401000	-5.235241000
C	-0.770826000	3.227909000	-3.051597000
C	-0.329191000	4.585980000	-2.485987000
C	0.453307000	5.580841000	-0.439615000
C	1.106750000	5.194566000	0.885730000
C	-0.640701000	5.327218000	2.563617000
C	-1.407337000	4.493704000	3.582130000
C	-3.028558000	2.692530000	3.769797000
C	-2.891217000	1.208078000	3.530120000

C	-3.589053000	0.560198000	2.499086000
C	-3.350496000	-0.787728000	2.219004000
C	-2.416848000	-1.528195000	2.959004000
C	-1.780738000	-0.898872000	4.038658000
C	-2.012611000	0.449442000	4.318278000
C	-2.050988000	-2.941736000	2.549174000
C	0.222507000	-2.380336000	1.852029000
C	-0.893331000	-4.279752000	0.820792000
H	1.411629000	-3.655620000	-0.613652000
H	-2.710069000	-4.784062000	-1.115632000
H	-2.471596000	-4.558404000	-3.574245000
H	-0.300319000	-3.803920000	-4.553006000
H	2.222123000	-2.161636000	-2.921071000
H	2.848672000	-3.783703000	-2.777877000
H	2.367293000	-4.590517000	-6.288194000
H	1.818068000	-5.326916000	-4.768565000
H	3.519511000	-4.818336000	-4.938323000
H	2.969804000	-2.376877000	-6.356857000
H	3.869919000	-2.062839000	-4.872471000
H	0.155912000	-1.563010000	-5.422093000
H	-0.938317000	0.685576000	-5.388829000
H	2.915823000	2.536711000	-4.954131000
H	3.996101000	0.311758000	-4.955915000
H	1.005013000	3.909254000	-4.927050000
H	-0.027463000	3.378431000	-6.267243000
H	-0.032839000	2.464025000	-2.770068000
H	-1.740732000	2.944405000	-2.635193000
H	0.553387000	4.964618000	-3.028182000
H	-1.140835000	5.324882000	-2.593951000
H	-0.390236000	6.276225000	-0.290378000
H	1.211815000	6.098921000	-1.052839000
H	1.942696000	4.515437000	0.685027000
H	1.504091000	6.103599000	1.367270000
H	-1.336978000	5.847103000	1.892936000
H	-0.053078000	6.086638000	3.107756000
H	-0.721535000	4.082121000	4.337830000
H	-2.118574000	5.169172000	4.090290000
H	-4.046081000	3.043078000	3.538830000
H	-2.814237000	2.923674000	4.822101000
H	-4.298899000	1.117022000	1.890353000

H	-3.877588000	-1.266812000	1.397372000
H	-1.074967000	-1.458373000	4.649460000
H	-1.480885000	0.924950000	5.138393000
H	-1.665572000	-3.497986000	3.427782000
H	-2.945766000	-3.467401000	2.193339000
H	0.080315000	-1.381109000	2.264424000
H	0.887246000	-2.286238000	0.991336000
H	0.717015000	-3.007828000	2.622656000
H	-1.802620000	-4.862731000	1.004918000
H	-0.058611000	-4.829847000	1.296848000
O	0.169749000	-0.174635000	-0.252330000
N	0.434953000	1.759813000	0.898806000
C	-0.337915000	0.890013000	0.162213000
C	-1.746227000	1.240224000	-0.188177000
C	-2.518258000	0.194980000	-0.738722000
C	-3.818607000	0.423721000	-1.195302000
C	-4.323393000	1.728098000	-1.081199000
C	-3.598780000	2.787104000	-0.517887000
C	-2.296568000	2.526071000	-0.060450000
C	-4.708510000	-0.676298000	-1.800849000
C	-5.040806000	-0.306204000	-3.270294000
C	-6.025080000	-0.786736000	-0.986529000
C	-4.010468000	-2.045675000	-1.785936000
C	-4.266267000	4.175344000	-0.463431000
C	-4.599116000	4.636177000	-1.907202000
C	-5.571617000	4.081760000	0.369649000
C	-3.357942000	5.229745000	0.190470000
C	1.814342000	1.503029000	1.141565000
C	2.315446000	1.355407000	2.445468000
C	3.689605000	1.273173000	2.759734000
C	4.506499000	1.053476000	1.621853000
C	4.083363000	0.987822000	0.269843000
C	2.735416000	1.367530000	0.093048000
C	4.225216000	1.421591000	4.233982000
C	5.572324000	2.207035000	4.312115000
C	4.409910000	0.014102000	4.863474000
C	3.252033000	2.247609000	5.130051000
C	4.928131000	0.461748000	-0.949523000
C	6.353250000	-0.073180000	-0.649617000
C	5.099788000	1.553441000	-2.044552000

C	4.163082000	-0.779791000	-1.501266000
Na	0.166985000	-0.738272000	-2.394117000
N	1.280697000	1.185522000	3.500344000
O	0.516779000	2.123126000	3.737029000
O	1.248720000	0.071584000	4.041076000
N	2.255328000	1.861742000	-1.247007000
O	2.412924000	3.067709000	-1.429907000
O	1.749388000	1.094916000	-2.084639000
N	5.981239000	0.960924000	1.853983000
O	6.427176000	-0.072501000	2.360371000
O	6.642781000	1.949547000	1.522103000
H	0.031302000	2.590671000	1.353447000
H	-2.094870000	-0.805056000	-0.732529000
H	-5.329935000	1.925969000	-1.439444000
H	-1.700609000	3.322235000	0.359964000
H	-5.584037000	0.643458000	-3.336063000
H	-5.667075000	-1.083972000	-3.725900000
H	-4.123233000	-0.209906000	-3.865699000
H	-6.593201000	0.149949000	-1.004377000
H	-5.818425000	-1.036046000	0.061555000
H	-6.663700000	-1.573879000	-1.406918000
H	-3.083922000	-2.041031000	-2.371024000
H	-4.668090000	-2.807288000	-2.222449000
H	-3.756654000	-2.364301000	-0.768250000
H	-3.688686000	4.701995000	-2.516367000
H	-5.069244000	5.627733000	-1.887367000
H	-5.288830000	3.948516000	-2.409968000
H	-6.285310000	3.370654000	-0.061972000
H	-5.350110000	3.761246000	1.395264000
H	-6.061622000	5.062562000	0.415651000
H	-3.143841000	4.960565000	1.228560000
H	-3.854262000	6.207784000	0.180362000
H	-2.409129000	5.322891000	-0.348087000
H	5.536634000	3.137575000	3.734426000
H	6.445258000	1.630069000	4.009911000
H	5.736025000	2.474315000	5.360599000
H	3.461866000	-0.531868000	4.886319000
H	4.767764000	0.130819000	5.894324000
H	5.143479000	-0.579338000	4.310887000
H	2.930594000	3.174282000	4.640835000

H	3.790689000	2.524100000	6.041358000
H	2.374930000	1.689377000	5.457957000
H	7.063540000	0.709390000	-0.375668000
H	6.718255000	-0.526596000	-1.577529000
H	6.355890000	-0.856069000	0.111747000
H	5.479938000	2.483046000	-1.606807000
H	4.187819000	1.774040000	-2.599459000
H	5.837529000	1.199930000	-2.774218000
H	3.134746000	-0.551898000	-1.773640000
H	4.153007000	-1.582697000	-0.754106000
H	4.680699000	-1.150859000	-2.393679000

5•6$_N$(e)···Na^+

O	1.205016000	2.451757000	-4.358092000
O	0.147792000	4.188056000	-2.190831000
O	0.671280000	5.439950000	0.410781000
O	-0.736129000	3.955619000	2.409986000
N	3.346010000	-4.069088000	-2.797390000
N	-1.452514000	-3.210251000	2.271984000
C	0.741182000	-4.125255000	-0.031592000
C	-0.656837000	-4.155357000	0.096245000
C	-1.448159000	-3.980189000	-1.048547000
C	-0.848857000	-3.783777000	-2.296412000
C	0.544761000	-3.776252000	-2.420693000
C	1.359488000	-3.945125000	-1.282928000
C	2.894970000	-3.847316000	-1.399885000
C	3.490396000	-5.493228000	-3.134230000
C	4.473504000	-3.178761000	-3.176333000
C	3.944992000	-1.738831000	-3.269214000
C	2.901522000	-1.426020000	-4.163953000
C	2.405236000	-0.120578000	-4.268766000
C	2.917332000	0.912464000	-3.459335000
C	3.953768000	0.602762000	-2.561151000
C	4.469937000	-0.700085000	-2.481691000
C	2.397499000	2.342118000	-3.575508000
C	-0.019572000	2.250027000	-3.625528000
C	-0.661024000	3.562975000	-3.200247000
C	-0.304476000	5.503482000	-1.854947000
C	0.669322000	6.118647000	-0.859955000
C	-0.117441000	6.075499000	1.427719000

C	-0.100068000	5.216937000	2.677951000
C	-0.990043000	3.193907000	3.628239000
C	-1.387010000	1.790991000	3.255667000
C	-2.697502000	1.479790000	2.867846000
C	-3.026560000	0.202097000	2.405588000
C	-2.049409000	-0.802681000	2.326537000
C	-0.749103000	-0.501292000	2.757849000
C	-0.423302000	0.773377000	3.218173000
C	-2.354388000	-2.169512000	1.707944000
C	-1.678239000	-3.498426000	3.694553000
C	-1.274716000	-4.427225000	1.468705000
N	-3.802553000	-2.440537000	1.733072000
N	-2.549344000	-5.190487000	1.291096000
N	3.535520000	-4.624765000	-0.331665000
N	5.088650000	-3.655523000	-4.418872000
H	1.366834000	-4.270602000	0.845357000
H	-2.529613000	-4.020628000	-0.963436000
H	-1.471588000	-3.657313000	-3.180206000
H	1.020571000	-3.662005000	-3.390644000
H	3.186553000	-2.808324000	-1.174206000
H	3.589839000	-5.616933000	-4.213118000
H	2.580112000	-6.013061000	-2.810114000
H	4.362804000	-5.977459000	-2.661577000
H	5.281813000	-3.204227000	-2.417314000
H	2.469947000	-2.214267000	-4.778594000
H	1.611824000	0.112966000	-4.974949000
H	4.354116000	1.375085000	-1.908060000
H	5.281330000	-0.913017000	-1.788784000
H	2.254380000	2.775282000	-2.576430000
H	3.150584000	2.954240000	-4.091943000
H	0.148042000	1.603817000	-2.754177000
H	-0.714379000	1.731785000	-4.301031000
H	-0.747090000	4.227674000	-4.077015000
H	-1.673769000	3.363037000	-2.807087000
H	-1.323314000	5.463061000	-1.432198000
H	-0.341100000	6.137466000	-2.760189000
H	1.689004000	6.039245000	-1.260741000
H	0.438047000	7.186991000	-0.728211000
H	-1.152412000	6.217102000	1.085138000
H	0.305228000	7.065894000	1.672713000

H	0.937221000	5.047769000	3.013378000
H	-0.639085000	5.754922000	3.476854000
H	-1.783264000	3.704146000	4.198775000
H	-0.074294000	3.183303000	4.240204000
H	-3.463906000	2.251993000	2.894016000
H	-4.035915000	-0.007811000	2.065231000
H	0.001568000	-1.285514000	2.716907000
H	0.599191000	0.993694000	3.521227000
H	-2.076236000	-2.102052000	0.647154000
H	-1.591576000	-2.573918000	4.275390000
H	-0.907330000	-4.194324000	4.046726000
H	-2.667861000	-3.938800000	3.917148000
H	-0.533448000	-5.019805000	2.031182000
H	-4.167400000	-2.391693000	2.686216000
H	-3.959469000	-3.389318000	1.385491000
H	-2.865283000	-5.542777000	2.196722000
H	-2.388118000	-6.009855000	0.700724000
H	4.545579000	-4.481373000	-0.326078000
H	3.358462000	-5.623905000	-0.441333000
H	5.986846000	-3.194088000	-4.561548000
H	4.502855000	-3.436496000	-5.227140000
O	-0.159947000	0.020898000	-0.784657000
N	0.239155000	1.988136000	0.286417000
C	-0.590590000	1.113459000	-0.332805000
C	-2.032137000	1.456449000	-0.503757000
C	-2.844043000	0.438036000	-1.029255000
C	-4.220883000	0.614980000	-1.183586000
C	-4.757256000	1.856016000	-0.807452000
C	-3.978524000	2.903840000	-0.289021000
C	-2.600422000	2.690130000	-0.143885000
C	-5.147408000	-0.489059000	-1.724788000
C	-5.826143000	0.009569000	-3.026886000
C	-6.225145000	-0.816075000	-0.656982000
C	-4.375605000	-1.788551000	-2.032542000
C	-4.678426000	4.219757000	0.105505000
C	-5.341455000	4.842701000	-1.151333000
C	-5.766160000	3.923466000	1.172237000
C	-3.698366000	5.250361000	0.697580000
C	1.634008000	1.777155000	0.517710000
C	2.117700000	0.562922000	1.044108000

C	3.477011000	0.412522000	1.342274000
C	4.341593000	1.497068000	1.083411000
C	3.880363000	2.715717000	0.572001000
C	2.507490000	2.838889000	0.287594000
C	4.030141000	-0.860658000	2.016749000
C	5.160730000	-1.472276000	1.152345000
C	2.941450000	-1.933763000	2.219749000
C	4.600320000	-0.481306000	3.410071000
C	4.801741000	3.930419000	0.348901000
C	6.277901000	3.620414000	0.674487000
C	4.323680000	5.086570000	1.269365000
C	4.718372000	4.388468000	-1.130547000
Na	1.478532000	-1.104080000	-1.481591000
H	-0.132244000	2.869275000	0.644728000
H	-2.365682000	-0.499178000	-1.284450000
H	-5.827431000	2.011557000	-0.914961000
H	-1.978325000	3.469596000	0.279506000
H	-6.426878000	0.909843000	-2.851105000
H	-6.491398000	-0.765208000	-3.429407000
H	-5.075012000	0.248794000	-3.791214000
H	-6.834604000	0.062370000	-0.414016000
H	-5.747587000	-1.178905000	0.261683000
H	-6.900500000	-1.597638000	-1.029893000
H	-3.595625000	-1.634833000	-2.789685000
H	-5.069075000	-2.546794000	-2.416346000
H	-3.910946000	-2.186377000	-1.124149000
H	-4.588230000	5.052719000	-1.921865000
H	-5.839657000	5.785612000	-0.890981000
H	-6.093222000	4.175579000	-1.588617000
H	-6.519741000	3.218424000	0.803848000
H	-5.317643000	3.493398000	2.076875000
H	-6.282356000	4.850272000	1.454053000
H	-3.178302000	4.858371000	1.578455000
H	-4.244440000	6.153674000	0.995345000
H	-2.944640000	5.548783000	-0.040891000
H	1.406976000	-0.219563000	1.275034000
H	5.394937000	1.389300000	1.318807000
H	2.092883000	3.767914000	-0.083403000
H	5.977780000	-0.761554000	0.984198000
H	4.770318000	-1.776565000	0.174814000

H	5.578114000	-2.359813000	1.644278000
H	2.164063000	-1.591876000	2.912668000
H	3.387765000	-2.843860000	2.635881000
H	2.458604000	-2.217939000	1.277699000
H	3.816856000	-0.044074000	4.041549000
H	5.415085000	0.246716000	3.329149000
H	4.991582000	-1.373219000	3.916281000
H	6.416417000	3.352691000	1.729422000
H	6.891371000	4.507407000	0.475527000
H	6.667536000	2.801395000	0.055818000
H	4.372446000	4.784456000	2.323396000
H	3.290511000	5.377603000	1.047653000
H	4.966482000	5.966642000	1.136032000
H	3.692307000	4.636401000	-1.422873000
H	5.083642000	3.605224000	-1.807055000
H	5.339428000	5.279922000	-1.284166000

5•6$_N$(f)····Na$^+$

O	3.550257000	1.963210000	-2.657214000
O	1.662569000	4.983958000	-1.846549000
O	-0.088291000	5.546587000	0.375345000
O	-1.124634000	4.076757000	2.444203000
N	2.003997000	-4.338380000	-3.540896000
N	-1.275538000	-3.202448000	2.553544000
C	0.329255000	-3.956171000	-0.307880000
C	-0.831476000	-4.362260000	0.370150000
C	-1.950890000	-4.781380000	-0.362650000
C	-1.904098000	-4.798949000	-1.758129000
C	-0.754998000	-4.391437000	-2.432513000
C	0.371632000	-3.967738000	-1.706582000
C	1.554683000	-3.485162000	-2.522233000
C	2.047648000	-5.801495000	-3.365074000
C	2.554063000	-3.832103000	-4.753455000
C	2.944100000	-2.369388000	-4.727983000
C	2.025053000	-1.348405000	-5.021693000
C	2.385314000	-0.011399000	-4.848857000
C	3.666699000	0.333897000	-4.385690000
C	4.595071000	-0.688925000	-4.134933000
C	4.238722000	-2.029417000	-4.309351000
C	3.978051000	1.767095000	-4.042024000

C	3.344518000	3.347511000	-2.270123000
C	1.906098000	3.779925000	-2.571488000
C	0.313088000	5.461574000	-1.982257000
C	-0.020838000	6.357777000	-0.805651000
C	-0.581988000	6.242776000	1.526262000
C	-0.471221000	5.326539000	2.733597000
C	-1.348985000	3.268031000	3.633250000
C	-1.586329000	1.840853000	3.212703000
C	-2.824723000	1.424722000	2.701617000
C	-3.022526000	0.105617000	2.290694000
C	-1.980184000	-0.829971000	2.380698000
C	-0.731922000	-0.405091000	2.855177000
C	-0.542667000	0.909920000	3.278070000
C	-2.160568000	-2.272142000	1.929944000
C	-1.201304000	-3.273390000	4.027972000
C	-0.819052000	-4.373557000	1.884764000
N	-3.699721000	-2.679974000	2.159459000
O	-4.347822000	-2.973005000	1.146495000
O	-4.113747000	-2.695627000	3.316447000
N	-1.661703000	-5.693092000	2.338816000
O	-1.027524000	-6.745896000	2.393361000
O	-2.857268000	-5.536621000	2.594589000
N	2.810045000	-3.152412000	-1.493775000
O	3.565587000	-4.059271000	-1.181498000
O	2.954388000	-1.962442000	-1.141541000
N	1.501164000	-4.037461000	-5.974967000
O	1.995319000	-4.148275000	-7.092863000
O	0.298621000	-4.042673000	-5.695508000
H	1.193116000	-3.646498000	0.275978000
H	-2.857495000	-5.091095000	0.148695000
H	-2.773775000	-5.126251000	-2.322113000
H	-0.718869000	-4.401587000	-3.519003000
H	1.366402000	-2.482759000	-2.906524000
H	1.435909000	-6.297020000	-4.130151000
H	1.656610000	-6.063611000	-2.381685000
H	3.082158000	-6.160375000	-3.435713000
H	3.388692000	-4.453999000	-5.093809000
H	1.023876000	-1.593153000	-5.369006000
H	1.659621000	0.771724000	-5.063481000
H	5.596856000	-0.437106000	-3.790862000

H	4.962454000	-2.813573000	-4.094474000
H	5.052937000	1.985544000	-4.115899000
H	3.428250000	2.455148000	-4.697598000
H	4.080252000	3.994159000	-2.766317000
H	3.510855000	3.390899000	-1.189809000
H	1.747479000	3.932301000	-3.654649000
H	1.215771000	2.986622000	-2.237148000
H	-0.388134000	4.611168000	-2.001686000
H	0.201731000	6.026306000	-2.924020000
H	0.744095000	7.143730000	-0.684836000
H	-0.992783000	6.846637000	-0.992560000
H	-1.629118000	6.541865000	1.361165000
H	0.012321000	7.153392000	1.718643000
H	0.585864000	5.133227000	2.980709000
H	-0.946697000	5.821937000	3.596113000
H	-2.211733000	3.680443000	4.180306000
H	-0.464842000	3.326297000	4.286304000
H	-3.642951000	2.136882000	2.622090000
H	-3.996240000	-0.186717000	1.905832000
H	0.090297000	-1.112507000	2.905869000
H	0.432814000	1.217920000	3.649037000
H	-2.069585000	-2.356388000	0.844535000
H	-1.466683000	-2.307602000	4.457112000
H	-0.173870000	-3.527695000	4.319397000
H	-1.891791000	-4.025549000	4.429168000
H	0.173892000	-4.645829000	2.260982000
O	0.401845000	0.508234000	-0.974734000
N	0.270164000	2.266326000	0.485793000
C	-0.281962000	1.397489000	-0.404867000
C	-1.741537000	1.539287000	-0.686010000
C	-2.394632000	0.433467000	-1.254251000
C	-3.765723000	0.471777000	-1.533181000
C	-4.455691000	1.656777000	-1.230065000
C	-3.835934000	2.784423000	-0.668527000
C	-2.462549000	2.711429000	-0.398979000
C	-4.514901000	-0.698381000	-2.197112000
C	-4.888478000	-0.278266000	-3.644392000
C	-5.808927000	-1.027627000	-1.408030000
C	-3.649246000	-1.971921000	-2.257941000
C	-4.677009000	4.043823000	-0.384075000

C	-5.249563000	4.586313000	-1.719910000
C	-5.847585000	3.681280000	0.567631000
C	-3.844621000	5.158259000	0.280060000
C	1.610001000	2.225470000	0.973744000
C	2.206011000	1.014237000	1.364730000
C	3.511983000	1.002142000	1.880584000
C	4.191621000	2.229043000	1.984901000
C	3.605626000	3.453579000	1.623484000
C	2.296585000	3.436383000	1.119333000
C	4.204496000	-0.300505000	2.332113000
C	5.376394000	-0.607047000	1.361104000
C	3.237749000	-1.505616000	2.342533000
C	4.761545000	-0.126203000	3.769688000
C	4.400729000	4.764501000	1.777161000
C	5.715169000	4.671478000	0.957077000
C	4.743894000	4.975104000	3.275239000
C	3.605705000	5.990537000	1.276058000
Na	2.529787000	0.230979000	-1.491325000
H	-0.318006000	2.993631000	0.901589000
H	-1.801406000	-0.449198000	-1.464153000
H	-5.518885000	1.705647000	-1.449484000
H	-1.956226000	3.568320000	0.031289000
H	-5.526211000	0.614045000	-3.651228000
H	-5.433641000	-1.088821000	-4.145400000
H	-3.987527000	-0.055334000	-4.230715000
H	-6.501521000	-0.178877000	-1.386020000
H	-5.581032000	-1.316948000	-0.376021000
H	-6.332527000	-1.866023000	-1.884106000
H	-2.743891000	-1.827206000	-2.860511000
H	-4.223525000	-2.785786000	-2.716687000
H	-3.356241000	-2.301293000	-1.255687000
H	-4.439106000	4.847829000	-2.412675000
H	-5.850153000	5.486963000	-1.537698000
H	-5.891615000	3.848869000	-2.215541000
H	-6.511712000	2.929127000	0.128245000
H	-5.471258000	3.285424000	1.519745000
H	-6.448889000	4.573617000	0.783912000
H	-3.412573000	4.826028000	1.230817000
H	-4.483431000	6.027745000	0.477131000
H	-3.024057000	5.487766000	-0.369320000

H	1.624938000	0.103523000	1.289632000
H	5.205218000	2.229605000	2.375651000
H	1.796012000	4.343179000	0.802325000
H	6.096585000	0.219133000	1.335780000
H	5.008742000	-0.775789000	0.340886000
H	5.908441000	-1.513449000	1.677258000
H	2.399474000	-1.337538000	3.030945000
H	3.772693000	-2.401280000	2.680134000
H	2.835438000	-1.718692000	1.345716000
H	3.958426000	0.132699000	4.471446000
H	5.522808000	0.659551000	3.823227000
H	5.224657000	-1.061374000	4.108713000
H	6.352924000	3.847227000	1.297161000
H	6.287676000	5.602131000	1.057613000
H	5.500903000	4.517663000	-0.108407000
H	5.333065000	4.142301000	3.676294000
H	3.829403000	5.061745000	3.876253000
H	5.327526000	5.895545000	3.404541000
H	2.676452000	6.128289000	1.842289000
H	3.346341000	5.904559000	0.214064000
H	4.210852000	6.895868000	1.405679000

5•6$_{O}$(a)···Na^{+}

Na	-0.868842000	2.151477000	2.405376000
O	-3.151305000	1.904402000	3.081994000
O	-1.375765000	3.998632000	3.811320000
O	0.686206000	2.076201000	4.265242000
O	3.476273000	0.944151000	3.559674000
O	-0.528395000	0.138475000	1.489712000
N	-3.161318000	-0.596857000	-3.460658000
N	2.199865000	1.137178000	-3.232284000
N	0.165265000	0.488585000	-0.633120000
C	-0.149416000	-0.376069000	0.387777000
C	-0.738753000	0.958366000	-4.208055000
C	0.367115000	1.495913000	-4.878275000
C	0.171124000	2.562932000	-5.764706000
C	-1.116490000	3.069278000	-5.978194000
C	-2.213208000	2.506189000	-5.318098000
C	-2.034465000	1.440942000	-4.420718000
C	-3.226414000	0.860536000	-3.682411000

C	-3.174767000	-1.330946000	-4.737830000
C	-4.285974000	-1.052339000	-2.605174000
C	-4.170564000	-0.603159000	-1.162634000
C	-4.816459000	0.557152000	-0.703241000
C	-4.700044000	0.963159000	0.627391000
C	-3.947923000	0.206404000	1.540301000
C	-3.318797000	-0.961465000	1.090532000
C	-3.424684000	-1.357786000	-0.244527000
C	-3.856758000	0.629271000	2.982821000
C	-3.292626000	2.532325000	4.370452000
C	-2.747210000	3.952340000	4.266819000
C	-0.353153000	4.171819000	4.817893000
C	0.184511000	2.854754000	5.365062000
C	1.344131000	0.853934000	4.693662000
C	2.208170000	0.281682000	3.585340000
C	4.479479000	0.215620000	2.804628000
C	4.255004000	0.226471000	1.305176000
C	4.855224000	1.209531000	0.501675000
C	4.626223000	1.247310000	-0.876665000
C	3.778186000	0.312680000	-1.490537000
C	3.206726000	-0.689493000	-0.692452000
C	3.441632000	-0.735949000	0.682926000
C	3.453184000	0.383910000	-2.968683000
C	2.380818000	2.577890000	-2.995639000
C	1.738653000	0.903971000	-4.622390000
C	0.024103000	-1.829078000	0.202702000
C	0.039660000	-2.434314000	-1.097009000
C	0.446668000	-3.767020000	-1.301234000
C	0.532198000	-4.577692000	-0.119829000
C	0.700021000	-4.018594000	1.188224000
C	0.312365000	-2.665323000	1.342118000
C	0.740140000	-4.317333000	-2.740231000
C	-0.556095000	-4.656807000	-3.528585000
C	1.609684000	-3.282337000	-3.526631000
C	1.640234000	-5.596047000	-2.762940000
C	1.260774000	-4.838349000	2.403383000
C	2.027404000	-6.145805000	2.022961000
C	0.137760000	-5.261937000	3.390916000
C	2.371523000	-4.009042000	3.129443000
C	0.110115000	1.912864000	-0.434066000

C	1.197568000	2.584340000	0.140037000
C	1.110425000	3.965417000	0.421680000
C	-0.087870000	4.621302000	0.098974000
C	-1.184831000	3.973282000	-0.501500000
C	-1.068949000	2.599255000	-0.761756000
C	2.303520000	4.728675000	1.035593000
C	3.386469000	4.910893000	-0.060963000
C	1.897697000	6.124992000	1.558100000
C	2.904491000	3.925197000	2.211687000
C	-2.477502000	4.765035000	-0.781176000
C	-3.205752000	4.989217000	0.572408000
C	-2.149600000	6.136360000	-1.425155000
C	-3.421465000	4.008311000	-1.738406000
N	0.206989000	-2.102830000	2.610022000
N	0.300139000	-5.926125000	-0.245308000
N	-0.430808000	-1.670037000	-2.177695000
H	0.642671000	0.159738000	-1.474258000
H	-0.570171000	0.133974000	-3.526174000
H	1.022140000	2.993622000	-6.290733000
H	-1.267711000	3.893606000	-6.672786000
H	-3.215503000	2.888674000	-5.508716000
H	-4.155600000	1.137426000	-4.223359000
H	-3.296689000	1.331326000	-2.695290000
H	-2.329016000	-1.015138000	-5.356438000
H	-3.086394000	-2.406413000	-4.542449000
H	-4.106589000	-1.159987000	-5.312837000
H	-5.256941000	-0.717579000	-3.023923000
H	-4.285532000	-2.148801000	-2.637294000
H	-5.428785000	1.137658000	-1.391408000
H	-5.203768000	1.868243000	0.963451000
H	-2.735302000	-1.562693000	1.783230000
H	-2.927919000	-2.268946000	-0.571951000
H	-3.326927000	-0.127572000	3.581637000
H	-4.861474000	0.767251000	3.413215000
H	-4.357779000	2.580822000	4.653413000
H	-2.763573000	1.945765000	5.141084000
H	-2.845079000	4.460024000	5.237393000
H	-3.327437000	4.507062000	3.519907000
H	0.456313000	4.717187000	4.318780000
H	-0.732919000	4.789490000	5.645033000

H	-0.595736000	2.292605000	5.907631000
H	0.998965000	3.073606000	6.076030000
H	1.969357000	1.068143000	5.572823000
H	0.573563000	0.118297000	4.979127000
H	1.689388000	0.359835000	2.620338000
H	2.347704000	-0.793066000	3.785809000
H	4.519962000	-0.822173000	3.176873000
H	5.423562000	0.711964000	3.053165000
H	5.510369000	1.949371000	0.959449000
H	5.111462000	2.009764000	-1.484325000
H	2.570668000	-1.443201000	-1.150317000
H	2.987351000	-1.529183000	1.272623000
H	3.303692000	-0.632034000	-3.353080000
H	4.304934000	0.825176000	-3.524680000
H	1.422707000	3.093902000	-3.104965000
H	2.740191000	2.744412000	-1.980548000
H	3.107486000	3.025797000	-3.704112000
H	2.463638000	1.313622000	-5.354300000
H	1.702759000	-0.183081000	-4.773817000
H	-0.308145000	-5.095925000	-4.504220000
H	-1.170220000	-5.378414000	-2.975256000
H	-1.190094000	-3.783137000	-3.724705000
H	2.582789000	-3.172033000	-3.031213000
H	1.789935000	-3.656857000	-4.541905000
H	1.169462000	-2.289146000	-3.606506000
H	2.417599000	-5.563369000	-1.992403000
H	1.084948000	-6.537849000	-2.668470000
H	2.135248000	-5.655095000	-3.738627000
H	1.378507000	-7.021121000	1.888745000
H	2.648950000	-6.017452000	1.130720000
H	2.688796000	-6.411424000	2.855558000
H	-0.403929000	-4.418643000	3.836853000
H	-0.609239000	-5.879547000	2.876144000
H	0.555706000	-5.849641000	4.219356000
H	2.062781000	-3.007326000	3.423678000
H	2.696583000	-4.547872000	4.028380000
H	3.240032000	-3.907362000	2.466695000
H	2.089976000	2.012199000	0.377297000
H	-0.176902000	5.677797000	0.321407000
H	-1.888406000	2.031999000	-1.188334000

H	4.240558000	5.472224000	0.339941000
H	3.753175000	3.941125000	-0.412215000
H	2.986004000	5.460401000	-0.921935000
H	2.761583000	6.594643000	2.043395000
H	1.572549000	6.794184000	0.751964000
H	1.088649000	6.062896000	2.298144000
H	3.722856000	4.491105000	2.675052000
H	2.154649000	3.711347000	2.981128000
H	3.310713000	2.964792000	1.889890000
H	-2.561382000	5.524211000	1.281469000
H	-4.119655000	5.579596000	0.423290000
H	-3.485352000	4.028405000	1.023925000
H	-1.561073000	6.778874000	-0.761107000
H	-1.587864000	6.003854000	-2.358132000
H	-3.079671000	6.669336000	-1.658434000
H	-2.930856000	3.804952000	-2.697681000
H	-3.758891000	3.059090000	-1.310631000
H	-4.313346000	4.615990000	-1.935076000
H	-0.295709000	-1.215611000	2.613773000
H	0.007915000	-2.739629000	3.367435000
H	0.295701000	-6.355284000	-1.155254000
H	0.455930000	-6.545344000	0.531956000
H	-0.733417000	-2.227342000	-2.966165000
H	-1.187503000	-1.029532000	-1.942569000

5•6$_{O}$(b)···Na^{+}

Na	-1.812280000	2.087437000	1.781662000
O	-3.409601000	2.914675000	3.276293000
O	-0.773656000	2.428748000	3.890752000
O	1.139342000	0.134547000	3.456376000
O	4.060252000	0.619444000	2.827280000
O	-1.170670000	0.106284000	0.971937000
N	-3.339993000	-0.978299000	-2.141266000
N	1.885677000	1.148761000	-3.519504000
N	0.102269000	0.779153000	-0.799037000
C	-0.399694000	-0.186059000	0.032580000
C	-1.009045000	-0.024264000	-4.253909000
C	-0.260779000	1.019260000	-4.814537000
C	-0.948782000	2.107500000	-5.369221000
C	-2.349307000	2.132586000	-5.370097000

C	-3.077701000	1.070646000	-4.820474000
C	-2.409975000	-0.022121000	-4.244104000
C	-3.139808000	-1.187669000	-3.602093000
C	-3.102992000	-2.203520000	-1.364902000
C	-4.679970000	-0.403161000	-1.872135000
C	-4.793312000	0.312725000	-0.540225000
C	-4.762869000	1.715606000	-0.507864000
C	-4.840011000	2.413132000	0.697254000
C	-4.924337000	1.717446000	1.915765000
C	-4.985892000	0.316071000	1.889102000
C	-4.944994000	-0.375294000	0.673616000
C	-4.798878000	2.473145000	3.211687000
C	-2.945853000	3.361447000	4.560713000
C	-1.442774000	3.597159000	4.414782000
C	-0.463204000	1.418154000	4.871913000
C	-0.103662000	0.115042000	4.162614000
C	2.285734000	-0.183603000	4.264862000
C	3.445250000	-0.563189000	3.362348000
C	5.211116000	0.343121000	1.972862000
C	4.833265000	0.365126000	0.509600000
C	5.157593000	1.474638000	-0.282706000
C	4.699417000	1.580081000	-1.599372000
C	3.891933000	0.580603000	-2.155056000
C	3.606364000	-0.553182000	-1.377326000
C	4.076577000	-0.669201000	-0.070340000
C	3.307603000	0.703915000	-3.545224000
C	1.799783000	2.575211000	-3.152514000
C	1.252259000	0.920857000	-4.845611000
C	0.068416000	-1.595651000	-0.146434000
C	0.565966000	-2.110317000	-1.357393000
C	1.014026000	-3.433084000	-1.439809000
C	0.974339000	-4.217781000	-0.268420000
C	0.488023000	-3.734408000	0.951666000
C	0.022906000	-2.411544000	0.987133000
C	1.553554000	-4.060331000	-2.740528000
C	0.711323000	-5.314192000	-3.096083000
C	1.485057000	-3.086914000	-3.935371000
C	3.035083000	-4.475079000	-2.531979000
C	0.438090000	-4.587143000	2.233735000
C	1.015725000	-6.003059000	2.027475000

C	-1.039266000	-4.716766000	2.690839000
C	1.258216000	-3.880034000	3.345761000
C	0.013880000	2.173080000	-0.437120000
C	0.995621000	2.692972000	0.449617000
C	0.881077000	4.027105000	0.936906000
C	-0.275114000	4.751431000	0.499329000
C	-1.055104000	4.363740000	-0.643528000
C	-0.975396000	2.990388000	-1.021642000
C	2.033351000	4.615777000	1.822245000
C	3.368243000	4.487595000	1.018136000
C	1.953233000	6.134439000	2.141044000
C	2.141044000	3.910876000	3.204716000
C	-1.851936000	5.374915000	-1.537522000
C	-3.388132000	5.180498000	-1.444366000
C	-1.576173000	6.883969000	-1.259436000
C	-1.365193000	5.200621000	-3.014167000
N	2.030151000	1.843895000	0.789357000
N	-1.894867000	2.362220000	-1.853593000
N	-0.785965000	5.793234000	1.297204000
H	0.739703000	0.524225000	-1.562949000
H	-0.484900000	-0.877692000	-3.832364000
H	-0.389108000	2.927959000	-5.815918000
H	-2.874140000	2.973860000	-5.819423000
H	-4.166082000	1.083421000	-4.859583000
H	-2.530861000	-2.089472000	-3.731487000
H	-4.099008000	-1.378822000	-4.115511000
H	-2.136622000	-2.630355000	-1.645763000
H	-3.056738000	-1.964325000	-0.298356000
H	-3.890847000	-2.969618000	-1.522014000
H	-4.891740000	0.322517000	-2.663930000
H	-5.455412000	-1.195203000	-1.940515000
H	-4.674816000	2.264354000	-1.440658000
H	-4.815204000	3.501329000	0.698842000
H	-5.067981000	-0.237624000	2.823703000
H	-5.024941000	-1.459998000	0.675071000
H	-5.016224000	1.827163000	4.075297000
H	-5.455637000	3.355757000	3.254028000
H	-3.444995000	4.298656000	4.858458000
H	-3.169148000	2.595899000	5.322313000
H	-1.006938000	3.901457000	5.378399000

H	-1.262648000	4.390894000	3.677316000
H	0.362259000	1.774828000	5.508624000
H	-1.336896000	1.227177000	5.516802000
H	-0.867692000	-0.115568000	3.408892000
H	-0.104027000	-0.695873000	4.912302000
H	2.572779000	0.666411000	4.905244000
H	2.050110000	-1.044265000	4.914401000
H	3.067136000	-1.214433000	2.559343000
H	4.188251000	-1.130053000	3.950473000
H	5.653775000	-0.620889000	2.265559000
H	5.937862000	1.135024000	2.188486000
H	5.758591000	2.276668000	0.143080000
H	4.956973000	2.457261000	-2.191391000
H	3.010435000	-1.355425000	-1.800417000
H	3.841938000	-1.566532000	0.499030000
H	3.332981000	-0.274384000	-4.040149000
H	3.919858000	1.395351000	-4.156101000
H	0.754487000	2.890149000	-3.149508000
H	2.206499000	2.729766000	-2.150352000
H	2.359907000	3.212024000	-3.866238000
H	1.655389000	1.625418000	-5.598847000
H	1.530790000	-0.089743000	-5.168582000
H	0.586889000	-1.473590000	-2.233761000
H	1.333504000	-5.239770000	-0.321423000
H	-0.371975000	-1.982183000	1.901267000
H	1.071730000	-5.757319000	-4.033078000
H	0.774321000	-6.082163000	-2.317313000
H	-0.345805000	-5.049205000	-3.225818000
H	2.056366000	-2.169846000	-3.749548000
H	1.908313000	-3.566216000	-4.826110000
H	0.450512000	-2.810862000	-4.171705000
H	3.658810000	-3.603053000	-2.298106000
H	3.142439000	-5.196446000	-1.713722000
H	3.428879000	-4.941071000	-3.444498000
H	0.457354000	-6.565093000	1.268415000
H	2.071018000	-5.971481000	1.728087000
H	0.953068000	-6.564885000	2.967180000
H	-1.485758000	-3.734188000	2.885011000
H	-1.641318000	-5.215604000	1.920966000
H	-1.102306000	-5.309057000	3.612947000

H	0.854119000	-2.886699000	3.570107000
H	1.237199000	-4.473415000	4.268948000
H	2.305629000	-3.759321000	3.040206000
H	4.209299000	4.816089000	1.642981000
H	3.572121000	3.476134000	0.670479000
H	3.327446000	5.138951000	0.135437000
H	2.916298000	6.447844000	2.561316000
H	1.786466000	6.740857000	1.241823000
H	1.203355000	6.373577000	2.905685000
H	3.041336000	4.256105000	3.730205000
H	1.270532000	4.163872000	3.819173000
H	2.182278000	2.820407000	3.157661000
H	-3.729979000	5.359996000	-0.416797000
H	-3.907734000	5.892298000	-2.100035000
H	-3.706636000	4.172967000	-1.722685000
H	-2.158160000	7.294734000	-0.424473000
H	-0.513986000	7.086847000	-1.087244000
H	-1.888554000	7.457186000	-2.140514000
H	-0.315557000	5.509375000	-3.094399000
H	-1.427390000	4.182399000	-3.401388000
H	-1.959856000	5.841779000	-3.677553000
H	2.519651000	1.945783000	1.667653000
H	1.920657000	0.872707000	0.529995000
H	-2.353972000	2.893905000	-2.575888000
H	-1.680483000	1.409335000	-2.131468000
H	-1.568088000	6.286477000	0.891728000
H	-0.126064000	6.429908000	1.722807000

5•6$_{O}$(c)···Na^{+}

Na	-1.277902000	1.633943000	2.769587000
O	-3.596255000	2.433530000	2.608079000
O	-1.486866000	3.279205000	4.332208000
O	1.135880000	2.033050000	4.141966000
O	4.107574000	1.197240000	3.386206000
O	-0.910835000	-0.192384000	1.540158000
N	-3.433944000	-0.146294000	-3.873568000
N	2.117568000	1.569535000	-3.082582000
N	0.950463000	0.430912000	0.356544000
C	0.080723000	-0.480657000	0.847620000
C	-0.953199000	1.387367000	-4.342904000

C	0.235488000	2.032710000	-4.727396000
C	0.151377000	3.246727000	-5.425059000
C	-1.094280000	3.797337000	-5.743985000
C	-2.265377000	3.130699000	-5.379896000
C	-2.208219000	1.919538000	-4.670953000
C	-3.516834000	1.272474000	-4.239985000
C	-3.240831000	-0.998206000	-5.053780000
C	-4.622404000	-0.570592000	-3.102818000
C	-4.672085000	0.014332000	-1.702494000
C	-5.569740000	1.036236000	-1.362704000
C	-5.599916000	1.564564000	-0.066976000
C	-4.749275000	1.063628000	0.929456000
C	-3.872640000	0.016987000	0.598978000
C	-3.822469000	-0.484104000	-0.701057000
C	-4.794116000	1.623650000	2.324932000
C	-3.812442000	3.307139000	3.738626000
C	-2.576888000	4.154301000	3.969790000
C	-0.242822000	3.971119000	4.588117000
C	0.751839000	2.980637000	5.154941000
C	2.068122000	1.053528000	4.682520000
C	2.925329000	0.411152000	3.602024000
C	5.114134000	0.518392000	2.591875000
C	4.800559000	0.557147000	1.111863000
C	5.095295000	1.709982000	0.367412000
C	4.700139000	1.825336000	-0.965697000
C	3.986304000	0.796791000	-1.600356000
C	3.763038000	-0.390920000	-0.880544000
C	4.163156000	-0.508388000	0.457707000
C	3.470856000	0.962118000	-3.016229000
C	2.128522000	2.973865000	-2.659858000
C	1.591975000	1.404424000	-4.464458000
C	0.359339000	-1.943359000	0.535713000
C	0.360639000	-2.427416000	-0.778048000
C	0.460771000	-3.794056000	-1.115347000
C	0.812226000	-4.609062000	-0.005826000
C	0.876431000	-4.221555000	1.359605000
C	0.502380000	-2.877892000	1.567734000
C	0.163651000	-4.328233000	-2.566210000
C	-0.401628000	-5.781056000	-2.589394000
C	-0.947793000	-3.479799000	-3.262869000

C	1.458917000	-4.289777000	-3.422344000
C	1.345667000	-5.153176000	2.538573000
C	2.518795000	-6.095510000	2.122387000
C	0.150143000	-6.000186000	3.052167000
C	1.919261000	-4.332073000	3.732970000
C	0.727018000	1.857572000	0.395290000
C	1.684068000	2.695563000	0.973183000
C	1.487959000	4.091371000	0.981726000
C	0.308600000	4.584653000	0.408688000
C	-0.661641000	3.757975000	-0.189218000
C	-0.441199000	2.374206000	-0.187141000
C	2.576245000	5.053796000	1.504172000
C	3.580087000	5.298866000	0.344157000
C	1.986307000	6.419628000	1.930631000
C	3.334447000	4.466859000	2.716958000
C	-1.881186000	4.398817000	-0.876287000
C	-2.678249000	5.242368000	0.150644000
C	-1.386323000	5.305455000	-2.035462000
C	-2.816518000	3.329909000	-1.459457000
N	0.101338000	-2.379832000	2.922435000
O	-0.924545000	-2.880909000	3.387952000
O	0.768498000	-1.484096000	3.449144000
N	1.160087000	-6.030350000	-0.317386000
O	2.231684000	-6.214333000	-0.903927000
O	0.365589000	-6.906686000	0.031226000
N	0.194040000	-1.376114000	-1.818055000
O	-0.842866000	-0.700575000	-1.781234000
O	1.126288000	-1.222876000	-2.618711000
H	1.806810000	0.112715000	-0.103839000
H	-0.905257000	0.458626000	-3.785028000
H	1.064325000	3.752953000	-5.735820000
H	-1.150336000	4.736036000	-6.292225000
H	-3.234219000	3.549616000	-5.651854000
H	-4.271533000	1.431219000	-5.040409000
H	-3.888755000	1.813896000	-3.362154000
H	-2.339405000	-0.686435000	-5.591152000
H	-3.117175000	-2.040500000	-4.739070000
H	-4.095108000	-0.945910000	-5.759762000
H	-5.561367000	-0.322824000	-3.640298000
H	-4.581621000	-1.664964000	-3.028417000

H	-6.249245000	1.426267000	-2.119528000
H	-6.292569000	2.371018000	0.170896000
H	-3.221561000	-0.411254000	1.355989000
H	-3.117978000	-1.274202000	-0.945645000
H	-4.845909000	0.823641000	3.080640000
H	-5.675731000	2.269019000	2.444237000
H	-4.668442000	3.970971000	3.536753000
H	-4.039220000	2.713099000	4.639907000
H	-2.772332000	4.865180000	4.788802000
H	-2.317729000	4.722919000	3.063730000
H	0.133867000	4.414203000	3.653199000
H	-0.411586000	4.775531000	5.321127000
H	0.304652000	2.459793000	6.018974000
H	1.640276000	3.529298000	5.509429000
H	2.735685000	1.551176000	5.401412000
H	1.492580000	0.279125000	5.208929000
H	2.351954000	0.284182000	2.674553000
H	3.215436000	-0.596123000	3.946365000
H	5.219742000	-0.520453000	2.945149000
H	6.045806000	1.054395000	2.804170000
H	5.627569000	2.531103000	0.845183000
H	4.943123000	2.727638000	-1.523550000
H	3.274394000	-1.226490000	-1.378359000
H	3.972710000	-1.435643000	0.998026000
H	3.408982000	-0.020828000	-3.496224000
H	4.194764000	1.568861000	-3.599540000
H	1.116144000	3.379502000	-2.710196000
H	2.471521000	3.047109000	-1.626051000
H	2.790350000	3.602018000	-3.292780000
H	2.314269000	1.829012000	-5.191065000
H	1.537339000	0.325316000	-4.650859000
H	-0.792331000	-5.962634000	-3.595409000
H	0.347940000	-6.552614000	-2.415861000
H	-1.228567000	-5.913030000	-1.883089000
H	-0.615051000	-2.508056000	-3.624081000
H	-1.279440000	-4.032313000	-4.147194000
H	-1.819780000	-3.342117000	-2.612686000
H	1.854704000	-3.274021000	-3.502757000
H	1.223369000	-4.651651000	-4.431091000
H	2.215538000	-6.944529000	1.511695000

H	3.316511000	-5.547874000	1.607490000
H	2.943649000	-6.517607000	3.038326000
H	-0.652158000	-5.359097000	3.430880000
H	-0.255255000	-6.639338000	2.264301000
H	0.497393000	-6.641252000	3.872486000
H	1.154533000	-3.850470000	4.342763000
H	2.440954000	-5.026776000	4.397970000
H	2.647534000	-3.580898000	3.404445000
H	2.579249000	2.263642000	1.401729000
H	0.143589000	5.656689000	0.392686000
H	-1.138963000	1.688065000	-0.653698000
H	4.360568000	6.003489000	0.659552000
H	4.061244000	4.364468000	0.040271000
H	3.070683000	5.719264000	-0.531781000
H	2.781306000	7.040968000	2.360134000
H	1.562217000	6.976009000	1.086792000
H	1.202599000	6.303454000	2.690874000
H	4.129498000	5.159021000	3.021396000
H	2.663060000	4.330850000	3.571590000
H	3.795434000	3.497444000	2.513538000
H	-2.059960000	6.021780000	0.612490000
H	-3.524400000	5.736351000	-0.344051000
H	-3.079028000	4.594126000	0.939971000
H	-0.717675000	6.097884000	-1.678804000
H	-0.852535000	4.715991000	-2.790145000
H	-2.242608000	5.782437000	-2.528820000
H	-2.292732000	2.721959000	-2.200421000
H	-3.211140000	2.672063000	-0.680881000
H	-3.668086000	3.807510000	-1.958480000
H	2.231964000	-4.937590000	-2.997723000

5•6$_{O}$(d)···Na^{+}

Na	-2.077955000	0.536188000	2.211758000
O	-3.650347000	-0.643930000	3.411553000
O	-1.423135000	0.553305000	4.416022000
O	1.432119000	-0.497501000	4.739085000
O	4.677007000	-0.388612000	3.144220000
O	0.071922000	0.790574000	1.631267000
N	-3.309844000	-1.150939000	-3.784591000
N	2.302325000	0.640985000	-3.267242000

N	1.114596000	1.092607000	-0.347597000
C	0.583443000	0.268326000	0.615929000
C	-0.884964000	0.477019000	-4.173145000
C	0.257599000	1.074468000	-4.720353000
C	0.106740000	2.243320000	-5.483863000
C	-1.164499000	2.781583000	-5.706832000
C	-2.296456000	2.149027000	-5.182476000
C	-2.167974000	0.991726000	-4.400600000
C	-3.395047000	0.319637000	-3.810145000
C	-3.255227000	-1.716559000	-5.142582000
C	-4.438363000	-1.720741000	-3.031207000
C	-4.349195000	-1.562688000	-1.522858000
C	-5.470256000	-1.160624000	-0.782623000
C	-5.460011000	-1.196177000	0.616071000
C	-4.327100000	-1.647281000	1.308559000
C	-3.182378000	-2.002596000	0.567179000
C	-3.190355000	-1.944526000	-0.827389000
C	-4.339521000	-1.783035000	2.808640000
C	-3.337523000	-0.848795000	4.806569000
C	-2.533862000	0.339549000	5.310852000
C	-0.289083000	1.254381000	4.992932000
C	0.688967000	0.294291000	5.671802000
C	2.662342000	0.124506000	4.308297000
C	3.316712000	-0.802582000	3.288694000
C	5.424804000	-1.198072000	2.206672000
C	5.000890000	-0.979496000	0.768892000
C	5.455486000	0.142460000	0.059452000
C	5.021529000	0.392174000	-1.244913000
C	4.099966000	-0.461650000	-1.869678000
C	3.683784000	-1.606698000	-1.174758000
C	4.122217000	-1.863776000	0.125073000
C	3.556467000	-0.187548000	-3.254692000
C	2.627201000	2.075482000	-3.133503000
C	1.608425000	0.406138000	-4.570149000
C	0.548927000	-1.210045000	0.434034000
C	0.531957000	-1.837755000	-0.823516000
C	0.399054000	-3.232513000	-0.913107000
C	0.285076000	-3.959614000	0.283471000
C	0.268307000	-3.360117000	1.552875000
C	0.404971000	-1.970204000	1.609345000

C	0.387711000	-4.006289000	-2.248317000
C	-0.908302000	-4.853911000	-2.354277000
C	0.444304000	-3.069242000	-3.468471000
C	1.618983000	-4.951757000	-2.291991000
C	0.126953000	-4.221353000	2.821216000
C	-0.944434000	-5.322095000	2.612007000
C	-0.290483000	-3.370422000	4.040908000
C	1.499209000	-4.883874000	3.115261000
C	0.754414000	2.482679000	-0.291355000
C	1.645993000	3.435348000	0.209881000
C	1.343053000	4.803380000	0.389052000
C	0.064177000	5.151861000	-0.123849000
C	-0.900409000	4.282404000	-0.693887000
C	-0.539036000	2.921952000	-0.623958000
C	2.268922000	5.781818000	1.208244000
C	3.789061000	5.451737000	1.113938000
C	2.182941000	7.277679000	0.779848000
C	1.825525000	5.644639000	2.694490000
C	-2.141180000	4.781153000	-1.514492000
C	-3.049795000	5.792228000	-0.760080000
C	-1.545419000	5.409513000	-2.817725000
C	-3.079441000	3.653026000	-2.010249000
N	2.933041000	2.841980000	0.683256000
O	3.863658000	2.802434000	-0.122687000
O	2.961860000	2.432666000	1.847882000
N	-0.395631000	6.565680000	0.077520000
O	-0.253338000	7.378553000	-0.836739000
O	-0.906303000	6.798699000	1.179252000
N	-1.565421000	1.850150000	-0.600281000
O	-1.460546000	0.872037000	-1.334213000
O	-2.433962000	1.970719000	0.297323000
H	1.442558000	0.721756000	-1.258527000
H	-0.790725000	-0.427657000	-3.582525000
H	0.981690000	2.713776000	-5.930507000
H	-1.275790000	3.678055000	-6.313881000
H	-3.287468000	2.551535000	-5.389482000
H	-4.293154000	0.657326000	-4.369595000
H	-3.534063000	0.648366000	-2.770719000
H	-2.402676000	-1.296065000	-5.684423000
H	-3.124754000	-2.802947000	-5.078190000

H	-4.172895000	-1.506798000	-5.728379000
H	-5.408811000	-1.314064000	-3.384108000
H	-4.460114000	-2.797664000	-3.251646000
H	-6.371872000	-0.844833000	-1.306212000
H	-6.349934000	-0.902489000	1.171309000
H	-2.292295000	-2.365541000	1.079901000
H	-2.303134000	-2.223189000	-1.386336000
H	-3.815547000	-2.701206000	3.116879000
H	-5.367296000	-1.820520000	3.198206000
H	-4.263129000	-0.946111000	5.396929000
H	-2.751661000	-1.774645000	4.913112000
H	-2.185108000	0.114135000	6.329495000
H	-3.152358000	1.251605000	5.351068000
H	0.198715000	1.764503000	4.155038000
H	-0.647454000	2.011237000	5.708460000
H	0.139021000	-0.412667000	6.308662000
H	1.371523000	0.868000000	6.321942000
H	2.468037000	1.107357000	3.857300000
H	3.335552000	0.254995000	5.171374000
H	2.786645000	-0.754811000	2.326967000
H	3.266763000	-1.844798000	3.654306000
H	5.325817000	-2.262991000	2.478933000
H	6.467713000	-0.896151000	2.353643000
H	6.145228000	0.834530000	0.538595000
H	5.399236000	1.261375000	-1.779223000
H	3.009280000	-2.305885000	-1.657640000
H	3.778033000	-2.759398000	0.640966000
H	3.306491000	-1.144134000	-3.727091000
H	4.325549000	0.293471000	-3.886553000
H	1.701126000	2.658104000	-3.082467000
H	3.202476000	2.246883000	-2.222907000
H	3.219728000	2.443660000	-3.993109000
H	2.265346000	0.732663000	-5.399355000
H	1.476911000	-0.676297000	-4.670788000
H	0.611181000	-1.235311000	-1.722565000
H	0.190044000	-5.039246000	0.219618000
H	0.399561000	-1.446959000	2.559335000
H	-0.919177000	-5.402244000	-3.304752000
H	-0.984585000	-5.588133000	-1.544662000
H	-1.799150000	-4.215638000	-2.317622000

H	1.335578000	-2.432048000	-3.448405000
H	0.483248000	-3.663690000	-4.389309000
H	-0.443260000	-2.429594000	-3.525871000
H	2.557279000	-4.386691000	-2.229960000
H	1.605626000	-5.671467000	-1.465662000
H	1.625225000	-5.518028000	-3.231915000
H	-1.912902000	-4.881960000	2.339398000
H	-0.664521000	-6.033817000	1.827393000
H	-1.076127000	-5.894043000	3.538743000
H	0.464713000	-2.626422000	4.311544000
H	-1.229775000	-2.837762000	3.841442000
H	-0.447744000	-4.024434000	4.907405000
H	2.266615000	-4.121024000	3.296975000
H	1.433143000	-5.522034000	4.005951000
H	1.828340000	-5.504704000	2.272999000
H	4.330018000	6.274689000	1.590749000
H	4.085667000	4.551074000	1.650556000
H	4.130719000	5.388391000	0.075825000
H	3.003729000	7.807633000	1.272200000
H	2.312195000	7.400229000	-0.300398000
H	1.270809000	7.782079000	1.095727000
H	2.458890000	6.291421000	3.314487000
H	0.782693000	5.949517000	2.829329000
H	1.941052000	4.612528000	3.044269000
H	-2.626379000	6.792496000	-0.666021000
H	-3.980386000	5.899474000	-1.328440000
H	-3.306577000	5.419163000	0.237576000
H	-1.014900000	4.645266000	-3.398140000
H	-2.372917000	5.785331000	-3.432198000
H	-0.864193000	6.236673000	-2.615576000
H	-2.544911000	2.872918000	-2.557534000
H	-3.674269000	3.209710000	-1.208546000
H	-3.775167000	4.105779000	-2.724306000

5•6$_{O}$(e)···Na^+

Na	-0.665958000	0.380330000	3.694003000
O	-2.939107000	0.620241000	3.105073000
O	-1.949241000	2.828729000	4.319334000
O	0.732853000	2.195019000	4.113142000
O	3.899987000	0.487614000	3.885296000

O	-0.153460000	0.133983000	1.563064000
N	-3.203641000	-0.391623000	-3.825106000
N	2.479858000	0.933833000	-2.947586000
N	0.550745000	0.769569000	-0.500074000
C	0.207029000	-0.177387000	0.411066000
C	-0.486768000	0.959643000	-4.017194000
C	0.695070000	1.360950000	-4.650696000
C	0.634898000	2.399047000	-5.593086000
C	-0.586039000	3.010367000	-5.894604000
C	-1.759816000	2.580408000	-5.266952000
C	-1.720043000	1.547226000	-4.320538000
C	-2.977195000	1.054063000	-3.635498000
C	-3.417217000	-0.717452000	-5.243380000
C	-4.342164000	-0.867896000	-3.006807000
C	-4.059199000	-0.842746000	-1.519988000
C	-4.448748000	0.242413000	-0.719147000
C	-4.166226000	0.265517000	0.646474000
C	-3.512644000	-0.812056000	1.266320000
C	-3.149502000	-1.909366000	0.474333000
C	-3.401688000	-1.912881000	-0.901045000
C	-3.267691000	-0.762342000	2.774251000
C	-3.780745000	1.286891000	4.066506000
C	-3.369874000	2.764977000	3.932851000
C	-1.115669000	3.584880000	3.403225000
C	0.280901000	3.601864000	4.045332000
C	2.145443000	2.035234000	4.175631000
C	2.464370000	0.592086000	3.713565000
C	4.596578000	-0.508431000	3.096461000
C	4.360340000	-0.364493000	1.597988000
C	4.923731000	0.729638000	0.921196000
C	4.736682000	0.890962000	-0.453779000
C	3.973203000	-0.031741000	-1.189694000
C	3.429653000	-1.129316000	-0.510821000
C	3.612296000	-1.292653000	0.863438000
C	3.706100000	0.130873000	-2.671847000
C	2.753221000	2.373932000	-2.777196000
C	2.003370000	0.669181000	-4.332728000
C	0.260149000	-1.616315000	0.000537000
C	0.077239000	-2.032348000	-1.327523000
C	0.088163000	-3.391296000	-1.660479000

C	0.293624000	-4.321947000	-0.622677000
C	0.477850000	-3.941826000	0.713845000
C	0.451266000	-2.569424000	1.006617000
C	-0.114501000	-3.898020000	-3.101277000
C	-1.336079000	-4.853657000	-3.149985000
C	-0.362450000	-2.745653000	-4.094964000
C	1.158537000	-4.666682000	-3.546048000
C	0.720071000	-4.965436000	1.841312000
C	0.610021000	-6.423373000	1.345944000
C	-0.325884000	-4.755088000	2.967362000
C	2.142531000	-4.750595000	2.422313000
C	0.390377000	2.174433000	-0.219216000
C	1.472323000	2.925342000	0.258444000
C	1.364154000	4.316691000	0.379199000
C	0.120535000	4.909131000	0.075144000
C	-0.989625000	4.170703000	-0.355920000
C	-0.829119000	2.780013000	-0.506326000
C	2.577889000	5.216505000	0.691921000
C	2.946078000	5.979937000	-0.612085000
C	2.248296000	6.249729000	1.798728000
C	3.812871000	4.403159000	1.133303000
C	-2.328838000	4.827988000	-0.746401000
C	-3.493572000	4.138227000	0.009854000
C	-2.370406000	6.337260000	-0.424251000
C	-2.528901000	4.652358000	-2.274979000
N	-2.189273000	-1.621593000	3.251516000
N	-3.630136000	0.702375000	5.392049000
N	-4.228645000	3.614270000	4.704632000
N	-1.644774000	4.919386000	3.182008000
N	0.249998000	4.304057000	5.295368000
N	2.639897000	2.407595000	5.507229000
N	1.678182000	-0.400336000	4.447711000
N	4.225337000	-1.849280000	3.577471000
H	1.075686000	0.538534000	-1.357564000
H	-0.464124000	0.156120000	-3.289879000
H	1.546004000	2.724164000	-6.093770000
H	-0.625142000	3.812692000	-6.628942000
H	-2.712895000	3.044745000	-5.516211000
H	-3.848178000	1.643574000	-3.991728000
H	-2.895144000	1.229147000	-2.555211000

H	-2.543243000	-0.413168000	-5.827914000
H	-3.548384000	-1.799983000	-5.352121000
H	-4.309857000	-0.212803000	-5.666033000
H	-5.258719000	-0.279412000	-3.221762000
H	-4.546480000	-1.900159000	-3.317030000
H	-4.981658000	1.079035000	-1.169437000
H	-4.449740000	1.132757000	1.235307000
H	-2.660707000	-2.775167000	0.914307000
H	-3.084815000	-2.764884000	-1.498000000
H	-4.189037000	-1.017268000	3.314966000
H	-4.835285000	1.189394000	3.772336000
H	-3.449235000	3.055287000	2.881512000
H	-1.070405000	3.061758000	2.437852000
H	0.970311000	4.107716000	3.359052000
H	2.635339000	2.708101000	3.456490000
H	2.209899000	0.494378000	2.651570000
H	5.648043000	-0.256850000	3.307076000
H	5.518597000	1.454245000	1.475344000
H	5.203149000	1.732435000	-0.963396000
H	2.843985000	-1.861904000	-1.060713000
H	3.158299000	-2.142623000	1.361232000
H	3.546939000	-0.861528000	-3.109479000
H	4.582524000	0.577056000	-3.179878000
H	1.830036000	2.945137000	-2.904096000
H	3.134966000	2.560178000	-1.773066000
H	3.499571000	2.734617000	-3.512213000
H	2.770181000	0.977757000	-5.069785000
H	1.879611000	-0.417519000	-4.428099000
H	-0.114679000	-1.290845000	-2.092487000
H	0.302366000	-5.377108000	-0.873386000
H	0.597926000	-2.213913000	2.022354000
H	-1.489996000	-5.216218000	-4.174243000
H	-1.199879000	-5.727167000	-2.502171000
H	-2.249812000	-4.336175000	-2.832338000
H	0.499620000	-2.069021000	-4.144336000
H	-0.513817000	-3.155339000	-5.101025000
H	-1.247817000	-2.153006000	-3.834848000
H	2.038404000	-4.010893000	-3.513319000
H	1.357330000	-5.531566000	-2.902766000
H	1.041917000	-5.031035000	-4.574750000

H	-0.382869000	-6.638095000	0.930929000
H	1.362342000	-6.651483000	0.580767000
H	0.776411000	-7.109023000	2.185433000
H	-0.239649000	-3.760893000	3.422419000
H	-1.345670000	-4.871345000	2.578166000
H	-0.179901000	-5.495803000	3.763846000
H	2.258097000	-3.749657000	2.853824000
H	2.342659000	-5.483497000	3.214972000
H	2.903332000	-4.876402000	1.640771000
H	2.399033000	2.406964000	0.479509000
H	0.034524000	5.988408000	0.148106000
H	-1.640689000	2.155162000	-0.868088000
H	3.824728000	6.616411000	-0.444991000
H	3.179255000	5.277028000	-1.421229000
H	2.121023000	6.618151000	-0.948421000
H	3.115236000	6.899515000	1.973100000
H	1.404969000	6.892784000	1.522692000
H	2.000624000	5.760228000	2.748108000
H	4.645210000	5.084210000	1.346721000
H	3.618362000	3.819416000	2.041102000
H	4.143112000	3.708989000	0.352855000
H	-3.409538000	4.312724000	1.089202000
H	-4.455192000	4.548499000	-0.324178000
H	-3.508323000	3.057132000	-0.163288000
H	-2.244473000	6.534201000	0.648105000
H	-1.602433000	6.896253000	-0.972598000
H	-3.345352000	6.744702000	-0.717014000
H	-1.710530000	5.123949000	-2.832951000
H	-2.555227000	3.596723000	-2.557906000
H	-3.473511000	5.114389000	-2.590707000
H	-1.676806000	-2.066993000	2.492986000
H	-2.514246000	-2.327536000	3.907266000
H	-4.483430000	0.771244000	5.943273000
H	-2.858199000	1.125800000	5.908492000
H	-3.994797000	4.591857000	4.531278000
H	-4.140624000	3.429556000	5.704823000
H	-1.383792000	5.256451000	2.256402000
H	-1.279466000	5.562782000	3.888192000
H	-0.433624000	3.880722000	5.924092000
H	1.169743000	4.288483000	5.737033000

H	3.652771000	2.272416000	5.540759000
H	2.223705000	1.808235000	6.224435000
H	1.892661000	-0.334980000	5.447067000
H	2.006180000	-1.325194000	4.155250000
H	4.570626000	-1.969766000	4.531561000
H	4.665744000	-2.570121000	3.002631000

5•6$_O$(f)···Na^+

Na	-1.053715000	-0.438966000	3.942869000
O	-3.473024000	0.062269000	3.219263000
O	-2.325907000	2.609962000	3.918981000
O	0.466680000	1.543044000	4.602265000
O	3.883978000	0.852896000	3.987579000
O	-0.370883000	0.271162000	1.905633000
N	-4.105183000	-0.643614000	-3.634209000
N	1.529795000	1.179542000	-2.815308000
N	-0.050376000	0.915017000	-0.257527000
C	-0.159960000	-0.034453000	0.697153000
C	-1.502873000	0.939458000	-3.822332000
C	-0.357145000	1.423323000	-4.468417000
C	-0.506814000	2.423967000	-5.441281000
C	-1.775404000	2.912313000	-5.767580000
C	-2.908899000	2.395047000	-5.133900000
C	-2.784004000	1.403220000	-4.150466000
C	-4.008764000	0.823968000	-3.469478000
C	-4.210561000	-1.030353000	-5.050168000
C	-5.227335000	-1.195623000	-2.860851000
C	-5.010648000	-1.156919000	-1.360193000
C	-6.081248000	-0.923281000	-0.485772000
C	-5.888397000	-0.934107000	0.899278000
C	-4.609264000	-1.164610000	1.424137000
C	-3.532467000	-1.408147000	0.555464000
C	-3.738021000	-1.408744000	-0.819479000
C	-4.371470000	-1.032625000	2.897347000
C	-3.921850000	1.305486000	2.772617000
C	-2.737427000	2.254208000	2.627288000
C	-1.127838000	3.288312000	4.141017000
C	-0.292720000	2.559464000	5.215362000
C	1.817520000	1.401333000	4.970834000
C	2.547230000	0.542190000	3.926633000

C	4.791348000	0.615572000	2.894436000
C	4.234763000	0.536038000	1.507633000
C	4.516029000	1.595267000	0.628624000
C	4.063849000	1.566205000	-0.690727000
C	3.301969000	0.487148000	-1.164057000
C	3.042065000	-0.577792000	-0.287410000
C	3.501546000	-0.563314000	1.028107000
C	2.800683000	0.430688000	-2.592990000
C	1.759026000	2.637716000	-2.724337000
C	1.007759000	0.839276000	-4.169668000
C	-0.057969000	-1.465719000	0.270442000
C	-0.319331000	-1.872557000	-1.054877000
C	-0.292165000	-3.226203000	-1.407204000
C	0.040232000	-4.158196000	-0.405089000
C	0.336136000	-3.794075000	0.914076000
C	0.268730000	-2.428222000	1.235948000
C	-0.643329000	-3.745798000	-2.814912000
C	-1.906265000	-4.644329000	-2.715262000
C	-0.941452000	-2.608244000	-3.809563000
C	0.545203000	-4.573752000	-3.370177000
C	0.767676000	-4.832275000	1.967194000
C	0.487702000	-6.280567000	1.507579000
C	0.015077000	-4.595626000	3.300843000
C	2.294869000	-4.672610000	2.195234000
C	-0.090567000	2.335486000	-0.026193000
C	0.850731000	2.944950000	0.812675000
C	0.942300000	4.345935000	0.863098000
C	0.053790000	5.088870000	0.067520000
C	-0.911868000	4.500492000	-0.764659000
C	-0.974174000	3.097635000	-0.795321000
C	2.072730000	5.061540000	1.631588000
C	3.231896000	5.315001000	0.627881000
C	1.607447000	6.417820000	2.214358000
C	2.609052000	4.195546000	2.792790000
C	-1.779152000	5.380585000	-1.685248000
C	-2.261640000	6.653616000	-0.945868000
C	-0.900699000	5.790175000	-2.900531000
C	-3.020879000	4.628392000	-2.211310000
N	-3.679870000	-2.308095000	3.457434000
O	-2.479790000	-2.261638000	3.773020000

O	-4.393508000	-3.297207000	3.515119000
N	-4.965190000	1.926815000	3.804683000
O	-5.386094000	1.185555000	4.687896000
O	-5.288660000	3.089895000	3.581238000
N	-3.143426000	3.489011000	1.744776000
O	-3.845401000	3.235452000	0.766387000
O	-2.643087000	4.568455000	2.038867000
N	-1.430675000	4.741168000	4.702553000
O	-0.407204000	5.420063000	4.853181000
O	-2.582120000	5.050284000	4.935148000
N	-1.309948000	1.899022000	6.222049000
O	-1.589798000	0.707759000	6.044732000
O	-1.773361000	2.650047000	7.063205000
N	1.968396000	0.802071000	6.439159000
O	2.890875000	0.022580000	6.634566000
O	1.167919000	1.240251000	7.264782000
N	2.264222000	-1.003189000	4.216896000
O	3.169355000	-1.792953000	4.032762000
O	1.097036000	-1.291117000	4.537143000
N	5.688885000	-0.655028000	3.286582000
O	6.120206000	-1.324914000	2.349212000
O	5.935250000	-0.792760000	4.476846000
H	0.303151000	0.676200000	-1.200988000
H	-1.412416000	0.156963000	-3.076062000
H	0.373318000	2.810920000	-5.952841000
H	-1.881829000	3.685241000	-6.525930000
H	-3.898153000	2.761277000	-5.403845000
H	-4.915626000	1.329938000	-3.858112000
H	-3.968636000	1.031836000	-2.390815000
H	-3.346102000	-0.648815000	-5.601289000
H	-4.217132000	-2.123564000	-5.126110000
H	-5.128483000	-0.639211000	-5.531905000
H	-6.187057000	-0.696064000	-3.106700000
H	-5.342732000	-2.247382000	-3.162869000
H	-7.073324000	-0.724958000	-0.888177000
H	-6.727138000	-0.747342000	1.568329000
H	-2.538777000	-1.596528000	0.953086000
H	-2.908329000	-1.592535000	-1.492378000
H	-5.288969000	-0.950115000	3.485510000
H	-4.476497000	1.253306000	1.830626000

H	-1.933405000	1.775911000	2.047061000
H	-0.530803000	3.451679000	3.238862000
H	0.305199000	3.250959000	5.809566000
H	2.327145000	2.368165000	5.046951000
H	2.078277000	0.675437000	2.940904000
H	5.517934000	1.428211000	2.978663000
H	5.113215000	2.437237000	0.973448000
H	4.325559000	2.381100000	-1.361367000
H	2.493309000	-1.443889000	-0.646492000
H	3.335968000	-1.432505000	1.656208000
H	2.609120000	-0.615391000	-2.858553000
H	3.585158000	0.807423000	-3.277519000
H	0.835157000	3.167171000	-2.959879000
H	2.056133000	2.913537000	-1.711242000
H	2.543771000	2.965825000	-3.433029000
H	1.723080000	1.171011000	-4.946130000
H	0.957637000	-0.254806000	-4.227728000
H	-0.582062000	-1.129069000	-1.798360000
H	0.062740000	-5.208950000	-0.669791000
H	0.488496000	-2.102083000	2.246359000
H	-2.186828000	-5.009627000	-3.711347000
H	-1.738322000	-5.516227000	-2.073093000
H	-2.752705000	-4.079870000	-2.304065000
H	-0.081844000	-1.935658000	-3.913180000
H	-1.144912000	-3.033089000	-4.799685000
H	-1.818166000	-2.015336000	-3.524062000
H	1.448748000	-3.954601000	-3.444307000
H	0.778199000	-5.437796000	-2.738513000
H	0.305207000	-4.948746000	-4.373044000
H	-0.574915000	-6.435462000	1.280400000
H	1.077329000	-6.557580000	0.624935000
H	0.761269000	-6.974553000	2.310623000
H	0.237227000	-3.614774000	3.731533000
H	-1.069952000	-4.671244000	3.162848000
H	0.318891000	-5.349475000	4.037240000
H	2.537755000	-3.679178000	2.586619000
H	2.649026000	-5.410777000	2.925649000
H	2.849603000	-4.821617000	1.259982000
H	1.545666000	2.312498000	1.353981000
H	0.130412000	6.169889000	0.080259000

H	-1.671380000	2.582985000	-1.447765000
H	4.073284000	5.810114000	1.130615000
H	3.589531000	4.372202000	0.197557000
H	2.898576000	5.959031000	-0.194118000
H	2.422638000	6.867737000	2.794539000
H	1.339363000	7.133174000	1.429786000
H	0.746009000	6.296513000	2.879345000
H	3.384735000	4.742513000	3.341541000
H	1.806602000	3.957327000	3.502576000
H	3.056029000	3.263542000	2.431547000
H	-1.433902000	7.316348000	-0.668349000
H	-2.927167000	7.226966000	-1.602661000
H	-2.814464000	6.394846000	-0.035876000
H	0.005042000	6.317858000	-2.577581000
H	-0.599817000	4.907920000	-3.479293000
H	-1.464891000	6.455452000	-3.566632000
H	-2.747514000	3.758312000	-2.815048000
H	-3.666583000	4.301827000	-1.390002000
H	-3.602122000	5.297057000	-2.857814000

5•6$_{N^-}\cdots Na^+$

O	10.207444000	8.870107000	9.167908000
O	9.358523000	11.486519000	10.267123000
O	9.101713000	12.232401000	12.966880000
O	8.144145000	11.526434000	15.713034000
O	8.970313000	7.057714000	11.887346000
N	13.117118000	3.587153000	10.301835000
N	7.722433000	4.227287000	14.457480000
N	9.460361000	9.255350000	12.430974000
H	9.180597000	10.213175000	12.225320000
C	9.994304000	3.574438000	12.384641000
H	10.438158000	3.517327000	13.378038000
C	8.604370000	3.424355000	12.257001000
C	8.029287000	3.520764000	10.983006000
H	6.951176000	3.420177000	10.872183000
C	8.831229000	3.741477000	9.857538000
H	8.374553000	3.803510000	8.870643000
C	10.218203000	3.868197000	9.994039000
H	10.859840000	3.999812000	9.124405000
C	10.812758000	3.799511000	11.269197000

C	12.307774000	4.051815000	11.435678000
H	12.467504000	5.134918000	11.536509000
H	12.652525000	3.615594000	12.392157000
C	13.493569000	2.180445000	10.434387000
H	13.996848000	1.843013000	9.519983000
H	12.589434000	1.574766000	10.572705000
H	14.168889000	1.990341000	11.295523000
C	14.249709000	4.483852000	9.973731000
H	14.846275000	3.965144000	9.209062000
H	14.918840000	4.659376000	10.839834000
C	13.731566000	5.796815000	9.432956000
C	12.989993000	5.799840000	8.222407000
H	12.911457000	4.878562000	7.647615000
C	12.318234000	6.943651000	7.800744000
H	11.709166000	6.911863000	6.896384000
C	12.370058000	8.137757000	8.558744000
C	13.192539000	8.164486000	9.712831000
H	13.234394000	9.060792000	10.323901000
C	13.850746000	7.006414000	10.141207000
H	14.424705000	7.039519000	11.065900000
C	11.363130000	9.223654000	8.325458000
H	11.732153000	10.211794000	8.633200000
H	11.037658000	9.266098000	7.273960000
C	8.960191000	9.524954000	8.867545000
H	8.246598000	9.110760000	9.588363000
H	8.634983000	9.255295000	7.845837000
C	8.951231000	11.047931000	8.964954000
H	9.598414000	11.511772000	8.199378000
H	7.913641000	11.365181000	8.760716000
C	8.795375000	12.753014000	10.637549000
H	7.700230000	12.658146000	10.723032000
H	9.020935000	13.520290000	9.874834000
C	9.396119000	13.199441000	11.958967000
H	10.488614000	13.316551000	11.853367000
H	8.973189000	14.184510000	12.229379000
C	9.759617000	12.507699000	14.216426000
H	9.410279000	13.474532000	14.617724000
H	10.848904000	12.565212000	14.048605000
C	9.479371000	11.396931000	15.210438000
H	9.631896000	10.420358000	14.731745000

H	10.203320000	11.484933000	16.043300000
C	7.800272000	10.499613000	16.680358000
H	6.877669000	10.865099000	17.146178000
H	8.588123000	10.453022000	17.452775000
C	7.582361000	9.137937000	16.060969000
C	6.351004000	8.823051000	15.466265000
H	5.544058000	9.553177000	15.489573000
C	6.157082000	7.600987000	14.821825000
H	5.206966000	7.395546000	14.331722000
C	7.193798000	6.660539000	14.744473000
C	8.414309000	6.957416000	15.367697000
H	9.211569000	6.221275000	15.321832000
C	8.606949000	8.178615000	16.015431000
H	9.569173000	8.396604000	16.479228000
C	7.035802000	5.409496000	13.908164000
H	5.955633000	5.205388000	13.746841000
H	7.480582000	5.623584000	12.927524000
C	7.115306000	3.784011000	15.717072000
H	7.131735000	4.605796000	16.440466000
H	7.688113000	2.943514000	16.128577000
H	6.060262000	3.458855000	15.590000000
C	7.751153000	3.129167000	13.476896000
H	6.725903000	2.857912000	13.143482000
H	8.157702000	2.246935000	13.991751000
C	8.548045000	8.252356000	12.096322000
C	7.160027000	8.623595000	11.908186000
C	6.297395000	7.659237000	11.312891000
H	6.743490000	6.716952000	11.018040000
C	4.940771000	7.904809000	11.137943000
C	4.425390000	9.153704000	11.552631000
H	3.365609000	9.356840000	11.424384000
C	5.247310000	10.141118000	12.141430000
C	6.604906000	9.871839000	12.312190000
H	7.233598000	10.594275000	12.819234000
C	3.989396000	6.855931000	10.528096000
C	3.325767000	7.434861000	9.251528000
H	2.754998000	8.344026000	9.473198000
H	2.638856000	6.701381000	8.806365000
H	4.088438000	7.691154000	8.504392000
C	2.889651000	6.499635000	11.563364000

H	2.309402000	7.382967000	11.853387000
H	3.339637000	6.079409000	12.472215000
H	2.194865000	5.756135000	11.148134000
C	4.722004000	5.551887000	10.144777000
H	5.508131000	5.732507000	9.400691000
H	4.007191000	4.837133000	9.716174000
H	5.185852000	5.083578000	11.021093000
C	4.606308000	11.471110000	12.591860000
H	2.669103000	10.561681000	13.136889000
C	4.035965000	12.204330000	11.348520000
H	4.837819000	12.422637000	10.630390000
H	3.568773000	13.154994000	11.641857000
H	3.281897000	11.597762000	10.833144000
C	3.449440000	11.186363000	13.586056000
H	3.824204000	10.665845000	14.476081000
H	2.985466000	12.128106000	13.910622000
C	5.611720000	12.410926000	13.289355000
H	6.067867000	11.954082000	14.174195000
H	5.097320000	13.328737000	13.604472000
H	6.428938000	12.696898000	12.618731000
C	10.820376000	9.102158000	12.722811000
C	11.325763000	7.982588000	13.426502000
H	10.633867000	7.195895000	13.693551000
C	12.669347000	7.939623000	13.819572000
C	13.518566000	9.005980000	13.466398000
H	14.556016000	8.980875000	13.785896000
C	13.054979000	10.108739000	12.726022000
C	11.706453000	10.143127000	12.358771000
H	11.303352000	10.957606000	11.769224000
C	13.229221000	6.802976000	14.702629000
C	12.180056000	5.709948000	14.992362000
H	11.766143000	5.287464000	14.071601000
H	11.346755000	6.100968000	15.586287000
H	12.641785000	4.893597000	15.562653000
C	13.679231000	7.404912000	16.061313000
H	14.471325000	8.150736000	15.928420000
H	14.060920000	6.614501000	16.722320000
H	12.834612000	7.895106000	16.562037000
C	14.449854000	6.138164000	14.015843000
H	15.242414000	6.864483000	13.802128000

H	14.157812000	5.669872000	13.069725000
H	14.872693000	5.357833000	14.663196000
C	14.024532000	11.260635000	12.383744000
C	15.362260000	10.711530000	11.822924000
H	15.870166000	10.057472000	12.539813000
H	16.041313000	11.543512000	11.592845000
H	15.201366000	10.139405000	10.901000000
C	14.312229000	12.059610000	13.683192000
H	14.765164000	11.417605000	14.448016000
H	13.382312000	12.470213000	14.097445000
H	14.998716000	12.893939000	13.482131000
C	13.437506000	12.229469000	11.333687000
H	12.543393000	12.740379000	11.707637000
H	13.165167000	11.707189000	10.407614000
H	14.178864000	12.999372000	11.084607000
Na	10.526364000	6.832563000	10.404131000

5•6$_{O^{-}\cdots}$$Na^+$

Na	-8.346931000	-11.916780000	-4.658615000
O	-10.652524000	-12.252145000	-4.184285000
O	-9.148153000	-9.974690000	-3.382526000
O	-7.141954000	-11.879555000	-2.535471000
O	-3.681566000	-12.547740000	-3.423069000
O	-7.437886000	-13.632634000	-5.611397000
N	-10.479678000	-14.465315000	-10.780987000
N	-4.908924000	-12.746788000	-10.221799000
N	-6.977761000	-13.130004000	-7.832569000
H	-6.248916000	-13.313524000	-8.531011000
C	-7.159058000	-14.060388000	-6.787491000
C	-7.926335000	-12.976417000	-11.172378000
H	-7.838324000	-13.807998000	-10.479634000
C	-6.790272000	-12.481640000	-11.828917000
C	-6.935418000	-11.402955000	-12.714638000
H	-6.058496000	-11.010661000	-13.228885000
C	-8.192196000	-10.831635000	-12.937290000
H	-8.296579000	-9.995311000	-13.626724000
C	-9.318690000	-11.347462000	-12.286565000
H	-10.302466000	-10.919197000	-12.477277000
C	-9.196802000	-12.426833000	-11.400798000
C	-10.414347000	-12.997079000	-10.703332000

H	-11.330069000	-12.525604000	-11.123051000
H	-10.385958000	-12.734397000	-9.637997000
C	-10.608943000	-14.932673000	-12.164939000
H	-9.760268000	-14.578798000	-12.758942000
H	-10.610590000	-16.028584000	-12.179700000
H	-11.543951000	-14.575162000	-12.647597000
C	-11.572700000	-15.013591000	-9.951769000
H	-12.562361000	-14.676797000	-10.330796000
H	-11.539956000	-16.105654000	-10.075606000
C	-11.464352000	-14.664573000	-8.483895000
C	-12.433975000	-13.851743000	-7.869402000
H	-13.260947000	-13.465764000	-8.465347000
C	-12.345918000	-13.530262000	-6.516902000
H	-13.100195000	-12.888751000	-6.061555000
C	-11.277459000	-14.013740000	-5.736402000
C	-10.316030000	-14.840407000	-6.344270000
H	-9.480468000	-15.217261000	-5.761015000
C	-10.406684000	-15.159712000	-7.699266000
H	-9.640869000	-15.783772000	-8.153098000
C	-11.177797000	-13.630782000	-4.292836000
H	-10.505369000	-14.302121000	-3.738656000
H	-12.168123000	-13.636448000	-3.810956000
C	-10.982487000	-11.597524000	-2.953045000
H	-12.073939000	-11.628790000	-2.784528000
H	-10.497467000	-12.101623000	-2.099582000
C	-10.551288000	-10.138398000	-3.071535000
H	-10.800423000	-9.596002000	-2.146235000
H	-11.091674000	-9.676017000	-3.906468000
C	-8.266984000	-9.723896000	-2.268490000
H	-7.409938000	-9.186989000	-2.693934000
H	-8.760423000	-9.066255000	-1.533833000
C	-7.791980000	-11.009008000	-1.589257000
H	-8.639616000	-11.564934000	-1.168390000
H	-7.111465000	-10.758300000	-0.760867000
C	-5.693976000	-11.837639000	-2.447759000
H	-5.332390000	-10.813261000	-2.617968000
H	-5.385088000	-12.153987000	-1.439679000
C	-5.096745000	-12.767058000	-3.496499000
H	-5.495166000	-12.551675000	-4.498807000
H	-5.347552000	-13.816730000	-3.270729000

C	-2.896129000	-13.561581000	-4.104923000
H	-3.160248000	-14.552739000	-3.699295000
H	-1.863241000	-13.328740000	-3.818157000
C	-3.052456000	-13.562317000	-5.606323000
C	-2.441964000	-12.566598000	-6.394346000
H	-1.837667000	-11.798052000	-5.912646000
C	-2.600047000	-12.557724000	-7.780846000
H	-2.108174000	-11.788162000	-8.375074000
C	-3.384131000	-13.535998000	-8.423317000
C	-3.962635000	-14.545032000	-7.637364000
H	-4.561265000	-15.319724000	-8.110498000
C	-3.807743000	-14.554321000	-6.251534000
H	-4.289199000	-15.338739000	-5.669391000
C	-3.661848000	-13.498402000	-9.910978000
H	-3.805251000	-14.523131000	-10.274470000
H	-2.799851000	-13.067135000	-10.459764000
C	-4.696816000	-11.297088000	-10.109187000
H	-5.646168000	-10.769633000	-10.233986000
H	-4.304190000	-11.058200000	-9.121958000
H	-3.979706000	-10.926809000	-10.872031000
C	-5.428865000	-13.092018000	-11.566591000
H	-4.721771000	-12.766793000	-12.357716000
H	-5.493770000	-14.185498000	-11.618433000
C	-7.047766000	-15.466471000	-7.091413000
C	-7.011352000	-15.984274000	-8.415798000
H	-7.115518000	-15.293675000	-9.246894000
C	-6.911475000	-17.356307000	-8.662658000
C	-6.825875000	-18.237002000	-7.567297000
H	-6.744487000	-19.303589000	-7.742385000
C	-6.860152000	-17.760556000	-6.229441000
C	-6.978433000	-16.395650000	-6.009390000
H	-7.012632000	-15.982055000	-5.007544000
C	-6.945214000	-17.863325000	-10.121170000
C	-8.319581000	-17.504850000	-10.745414000
H	-8.365269000	-17.835260000	-11.793257000
H	-9.129218000	-18.000822000	-10.193661000
H	-8.509470000	-16.426858000	-10.715041000
C	-5.820202000	-17.178614000	-10.937716000
H	-4.834757000	-17.414759000	-10.515322000
H	-5.838983000	-17.516714000	-11.983342000

H	-5.937401000	-16.090422000	-10.932573000
C	-6.747093000	-19.390789000	-10.226529000
H	-5.784232000	-19.703387000	-9.802567000
H	-7.544488000	-19.938435000	-9.708889000
H	-6.765436000	-19.692766000	-11.282038000
C	-6.770161000	-18.776508000	-5.073115000
C	-5.440469000	-19.568657000	-5.183442000
H	-5.371861000	-20.108234000	-6.134867000
H	-4.581187000	-18.888214000	-5.121242000
H	-5.360506000	-20.302364000	-4.368897000
C	-7.964454000	-19.763407000	-5.161211000
H	-8.916341000	-19.222200000	-5.084030000
H	-7.965339000	-20.308221000	-6.112243000
H	-7.917289000	-20.500037000	-4.346644000
C	-6.809232000	-18.098933000	-3.686249000
H	-7.741188000	-17.539310000	-3.537025000
H	-6.743269000	-18.862018000	-2.899472000
H	-5.969637000	-17.404626000	-3.553896000
C	-7.176661000	-11.746542000	-7.551810000
C	-6.120946000	-10.933976000	-7.113626000
H	-5.143400000	-11.390242000	-6.982794000
C	-6.330761000	-9.574987000	-6.822018000
C	-7.631150000	-9.061684000	-6.958891000
H	-7.808338000	-8.017000000	-6.736376000
C	-8.718462000	-9.859918000	-7.371222000
C	-8.468908000	-11.208041000	-7.667577000
H	-9.259434000	-11.875494000	-7.994928000
C	-5.157603000	-8.712663000	-6.311045000
C	-3.956032000	-8.818638000	-7.283911000
H	-3.127581000	-8.189346000	-6.931649000
H	-3.586636000	-9.846417000	-7.354553000
H	-4.237607000	-8.484615000	-8.290451000
C	-5.535401000	-7.221921000	-6.172186000
H	-4.663690000	-6.653678000	-5.823511000
H	-5.854105000	-6.794946000	-7.131610000
H	-6.342788000	-7.070334000	-5.444397000
C	-4.728001000	-9.240770000	-4.918289000
H	-3.896715000	-8.645299000	-4.516648000
H	-5.568563000	-9.169874000	-4.215096000
H	-4.403922000	-10.285626000	-4.961439000

C	-10.117999000	-9.248740000	-7.585744000
C	-10.357539000	-8.033942000	-6.656822000
H	-9.702920000	-7.189498000	-6.902349000
H	-11.392788000	-7.684790000	-6.762309000
H	-10.185724000	-8.299513000	-5.605724000
C	-10.213280000	-8.783762000	-9.064609000
H	-9.451564000	-8.024789000	-9.284287000
H	-10.054589000	-9.626271000	-9.748524000
H	-11.202560000	-8.350656000	-9.269486000
C	-11.230476000	-10.287650000	-7.313666000
H	-11.206293000	-11.117537000	-8.025770000
H	-11.145192000	-10.718059000	-6.309209000
H	-12.215508000	-9.810909000	-7.405212000
